# Global, regional, and national burden of knee osteoarthritis attributable to high BMI: a systematic analysis from 1990 to 2021 and projections to 2050

**DOI:** 10.3389/fpubh.2025.1668257

**Published:** 2025-10-15

**Authors:** Jiaxian Xu, Mingming Lei, Dandan Xu

**Affiliations:** ^1^College of Sports Medicine and Health, Chengdu Sport University, Chengdu, China; ^2^Department of Sports Injury, Affiliated Sports Hospital of Chengdu Sport University, Chengdu Sport University, Chengdu, China; ^3^Department of Intensive Care Unit, The Affiliated Hospital of Xuzhou Medical University, Xuzhou, Jiangsu, China

**Keywords:** knee osteoarthritis, high body mass index, global burden, projections, public health

## Abstract

**Background:**

Knee osteoarthritis (KOA) is one of the most prevalent joint diseases globally, with high body mass index (BMI) being a major risk factor. We aimed to assess the burden of KOA attributable to high BMI across global, regional, and national levels from 1990 to 2021 and to project trends to 2050.

**Methods:**

Data were obtained from the Global Health Data Exchange (GHDx) and identified using the M17 code from the International Classification of Diseases, 10th Revision (ICD-10). The analysis focused on disability-adjusted life years (DALYs) and years lived with disability (YLDs). Using R software, we calculated age-standardized rates and generated world maps to illustrate the distribution and trends. Future burden was projected using a combination of log-linear models and Bayesian inference, in line with the Global Burden of Disease (GBD) research standards. Parameters were calibrated using demographic projections from the GBD 2021 study.

**Findings:**

Between 1990 and 2021, there was a significant increase in global DALYs and YLDs of KOA due to high BMI, with a greater burden observed in females compared with males. The most rapid increase was seen in low-middle Socio-demographic Index (SDI) countries. In 2021, the highest burden was in China, the United States, and India. Projections indicate that by 2050, global DALYs and YLDs will nearly double, with females continuing to have a higher burden than males and the highest age-standardized DALY rates in high SDI regions.

**Interpretation:**

The impact of high BMI on the burden of KOA is substantial. Future efforts should focus on individuals aged 50 years and older, females, and low-middle SDI regions for intensified health education and interventions. Promoting a healthy lifestyle, including balanced diets and increased physical activity, is essential to mitigate the future burden of KOA attributable to high BMI. Region-specific interventions are needed. For example, low-middle SDI regions, where obesity rates are rising alongside limited healthcare resources, would benefit from targeted health education programs, including weight management, physical activity promotion, and access to affordable healthcare. High SDI regions should focus on obesity prevention through policy interventions that regulate food quality and encourage physical activity.

## 1 Introduction

Knee osteoarthritis (KOA), the most prevalent form of osteoarthritis (OA), is a widespread chronic joint disorder characterized by articular cartilage degeneration, joint space narrowing, and osteophyte formation, resulting in pain, stiffness, and disability (1, 2). The pathogenesis of KOA is intricate and encompasses various joint structures such as articular cartilage, subchondral bone, synovial tissue, and meniscus (3). As a critical public health concern, KOA has impacted more than 250 million individuals globally and is the 11th leading cause of disability worldwide (4, 5). Knee osteoarthritis (KOA) accounts for approximately 80% of the global osteoarthritis (OA) burden (6), as life expectancy increases and obesity rates rise, the prevalence of KOA is on the rise, and the age of onset is becoming younger (7, 8). Obesity [body mass index (BMI) ≥ 30 kg/m^2^] is among the most significant risk factors for KOA (9), it may serve as a mediating factor between diet and disease incidence (10). Obesity not only heightens the risk of developing radiographic knee osteoarthritis (RKOA) but also hastens its progression to symptomatic knee osteoarthritis (SxKOA) (11). Both generalized obesity and central obesity are linked to an elevated risk of knee osteoarthritis (KOA) (12). In addition to having a higher risk of developing KOA, women typically exhibit more severe pain and functional limitations compared to men (9, 13). The pathogenesis of KOA remains incompletely understood, and no curative drugs are currently available (14). The current treatment strategies for KOA encompass conservative approaches, including weight loss, exercise, and the use of non-steroidal anti-inflammatory drugs (NSAIDs) (15). However, these methods have limited efficacy. Although knee replacement surgery is an effective treatment for late-stage KOA, it is costly and cannot provide a complete cure (6). Considering the aging population and increasing obesity rates, the prevalence of KOA is projected to continue rising (13). Analyzing the global KOA burden attributable to high BMI from 1990 to 2021 and projecting the trends to 2050 is essential for developing public health strategies and optimizing resource allocation.

## 2 Methods

### 2.1 Data source and acquisition

This study utilized data from the Global Burden of Disease (GBD) 2021 study, accessed via the Global Health Data Exchange (GHDx) on 2024/09/26. The GBD 2021 study provides comprehensive health loss estimates for 371 diseases and injuries across 204 countries and regions, categorized by age and sex. It also evaluates 88 risk factors and 288 causes of death, measuring outcomes such as incidence, prevalence, mortality, years of life lost (YLL), years lived with disability (YLD), and disability-adjusted life years (DALYs). For this analysis, we focused on the burden of knee osteoarthritis (KOA) attributable to high body mass index (BMI) using the M17 code from the International Classification of Diseases, 10th Revision (ICD-10). Data were extracted for the period from 1990 to 2021, with projections made to 2050.

### 2.2 Case definition and risk attribution

KOA estimates were defined according to the ICD-10 code M17, representing symptomatic osteoarthritis confirmed radiographically by Kellgren–Lawrence (KL) grades II–IV. A KL grade of II indicates the presence of a definite osteophyte; grade III indicates multiple osteophytes and joint space narrowing; grade IV indicates joint deformity in addition to the findings of grade III. Symptomatic OA requires the presence of pain for at least 1 month within the last 12 months. High BMI is a GBD risk factor, identified as a BMI exceeding the ideal range of 20–25 kg/m^2^ for adults aged 20 and above (16). Population-attributable risk (PAR) for KOA due to high BMI was calculated using the GBD methodology, which incorporates age, sex, location, and year to estimate the disease burden attributable to high BMI.

### 2.3 Socio-demographic index (SDI) stratification

In this study, we utilized the Socio-demographic Index (SDI) to categorize countries and regions based on their developmental status. Developed by the Institute for Health Metrics and Evaluation (IHME), the SDI is a composite metric that integrates per capita income, average years of schooling, and total fertility rate (TFR) into a unified measure. This index serves as an integrated developmental indicator, reflecting a country’s fertility rates among women under 25 years, educational attainment levels, and lag-distributed per capita income. The SDI ranges from 0 to 1, with higher values indicating more advanced levels of development. According to the 2021 SDI data, 204 countries and regions were stratified into five distinct categories: low SDI (<0.466), low-middle SDI (0.466–0.619), middle SDI (0.619–0.720), high-middle SDI (0.720–0.810), and high SDI (≥0.810) (17, 18). These quartiles reflect both economic and healthcare infrastructure disparities, which contribute to varying disease burdens, particularly in low-middle SDI regions where medical resource shortages are prevalent.

### 2.4 Statistical analysis

#### 2.4.1 Trend analysis

The estimated annual percentage change (EAPC) was calculated to evaluate trends in the disease burden. Age-standardized rates (ASRs) per 100,000 individuals were used for population-wide comparisons. A linear regression model (y = *α* + *β*x), where y = ln (ASR) and x = year, was applied. EAPC and its 95% confidence intervals (CIs) were calculated using the formula EAPC = (exp(β) − 1) × 100%.

#### 2.4.2 Forecasting methodology

Future projections of DALYs and YLDs attributable to high BMI were based on a combination of log-linear models and Bayesian inference. The assumptions made for these projections included:

- BMI Trends: Future BMI trends were projected based on historical data and current trajectories, assuming a continuation of recent trends unless significant public health interventions are implemented.
- Aging Structure: Population aging was accounted for using demographic projections from the GBD 2021 study, which incorporate fertility rates, mortality rates, and migration patterns.
- Medical Interventions: The impact of medical interventions was modeled based on current trends in healthcare access and quality (HAQ) indices, with adjustments for regional differences in healthcare infrastructure. Model fit was assessed using cross-validation techniques, where the model was trained on data from 1990 to 2010 and validated against data from 2011 to 2021. Out-of-sample forecasts were also performed to evaluate the model’s predictive accuracy. Additionally, we compared linear and non-linear trend forecasts, particularly for regions experiencing epidemiological transitions, to ensure robustness in our projections.

#### 2.4.3 Sensitivity analysis

To address potential limitations in the use of the ICD-10 M17 code, we conducted sensitivity analyses considering central obesity (waist-to-hip ratio) as an additional risk factor. This approach allowed us to explore the impact of different definitions of obesity on the burden of KOA.

#### 2.4.4 Geographical and gender-specific analysis

The data were analyzed at the global, regional, and national levels, with separate analyses conducted for males and females. ASRs per 100,000 individuals were calculated for each age group, and percentage changes in ASRs from 1990 to 2021 were reported. The burden of disease was also mapped globally to visualize regional differences.

#### 2.4.5 Age-period-cohort analysis

An age-period-cohort (APC) model was used to estimate the independent effects of age, period, and cohort on KOA attributable to high BMI. Data from 1992 to 2021 were grouped into 5-year intervals, and the intrinsic estimator (IE) was used to determine the rate ratio of specific ages, periods, and birth cohorts relative to the average combined level across all ages, periods, and cohorts.

#### 2.4.6 DALY calculation and uncertainty estimation

DALYs were calculated following the standard GBD methodology, which combines years of life lost (YLL) due to premature mortality and years lived with disability (YLD). For KOA, which primarily contributes to YLD rather than YLL, disability weights were applied based on the severity distribution of the condition. The GBD 2021 study used no age-weighting or time discounting in DALY calculations, ensuring comparability across different time periods and age groups. Uncertainty intervals were directly obtained from the GBD 2021 database, which computes them through a sampling process of 1,000 draws from the posterior distribution of each estimation step, accounting for sampling error, non-sampling error, and model uncertainty.

### 2.5 Software and packages

All analyses were conducted using R software (version 4.5.0). The following R packages were utilized:

- Data manipulation and analysis: dplyr, tidyr, stringr, arrow.
- Data visualization: ggplot2, ggmap, rgdal, RColorBrewer, patchwork, ggrepel.
- Geospatial analysis: rgdal.
- Statistical analysis: stats.
- File output: writexl.

### 2.6 Abbreviations

- ASR: Age-standardized rates (ASRs) are rates that have been adjusted to account for differences in age distribution across populations, allowing for more accurate comparisons between different regions and time periods.
- AS YLD rate: Age-standardized years lived with disability (YLD) rate is a measure of the average number of years lost due to disability per 100,000 individuals, adjusted for age distribution.
- ASDR: Age-standardized disability-adjusted life years (DALY) rate is a measure of the average number of DALYs per 100,000 individuals, adjusted for age distribution.
- DALY: Disability-adjusted life years (DALYs) are a measure of overall disease burden, expressed as the number of years lost due to ill-health, disability, or early death.
- EAPC: Estimated annual percentage change (EAPC) is a measure used to describe the average annual rate of change in a given metric over a specified period, calculated using the formula EAPC = (exp(*β*) − 1) × 100%, where β is the slope of the linear regression line.
- HAQ: Healthcare access and quality (HAQ) indices are composite measures that reflect the performance of healthcare systems in terms of access to care and the quality of care provided.
- ICD-10: International Classification of Diseases, 10th Revision (ICD-10) is a system of codes used to classify diseases and related health conditions.
- IE: Intrinsic estimator (IE) is a statistical method used in age-period-cohort (APC) models to estimate the independent effects of age, period, and cohort.
- KOA: Knee osteoarthritis (KOA) is a degenerative joint disease characterized by the breakdown of cartilage in the knee joint.
- M17: ICD-10 code for knee osteoarthritis.
- PAR: Population-attributable risk (PAR) is a measure used to estimate the proportion of disease burden in a population that can be attributed to a specific risk factor.
- SDI: Socio-demographic Index (SDI) is a composite measure that reflects the level of development of a region based on factors such as per capita income, educational attainment, and fertility rates.
- UI: Uncertainty intervals (UI) are ranges that provide an estimate of the precision of a given metric, typically representing the 95% confidence intervals around the point estimate.
- YLD: Years lived with disability (YLDs) are a measure of the number of years lost due to disability from a specific disease or condition.
- YLL: Years of life lost (YLLs) are a measure of the number of years lost due to premature mortality from a specific disease or condition.
- High BMI: High body mass index (High BMI) is defined as a BMI exceeding the ideal range of 20–25 kg/m^2^ for adults aged 20 and above, which is identified as a significant risk factor for various health conditions, including knee osteoarthritis (KOA).

## 3 Results

### 3.1 The global burden of knee osteoarthritis (KOA) attributable to high BMI from 1990 to 2021 and projections to 2050

**Figure 1:**
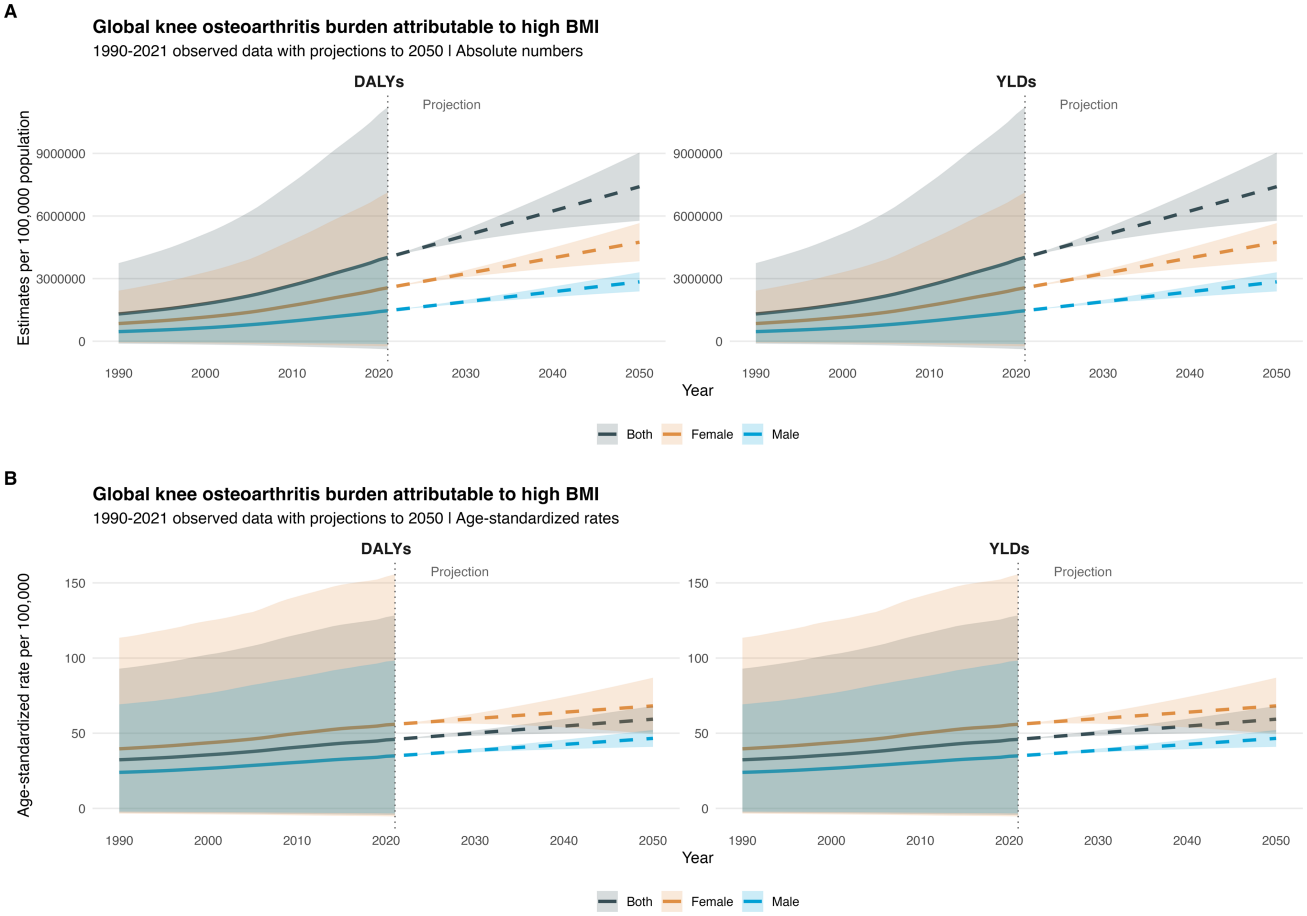
Global burden of knee osteoarthritis (KOA) attributable to high body mass index (BMI) from 1990 to 2021: Time trends, gender differences, and projections to 2050: **(A)** estimates of knee osteoarthritis (KOA) attributable to high BMI **(B)** age-standardized rate of knee osteoarthritis (KOA) Attributable to High BMI.

From 1990 to 2021, there was a significant increase in the global burden of KOA attributable to high body mass index (BMI), with disability-adjusted life years (DALYs) and years lived with disability (YLDs) nearly doubling. Specifically, DALYs increased from 1,306,589.34 years in 1990 (95% uncertainty interval [UI] −114,326.79–3,742,191) to 4,019,554.91 years in 2021 (−382,558.22–11,222,318). Among females, DALYs attributable to high BMI for KOA also saw a significant rise, increasing from 847,154.06 years in 1990 (95% UI −73,596.49–2,424,497.98) to 2,559,536.58 years in 2021 (95% UI −243,349.42–7,130,008.32) (Figure 1A; Appendix, pp. 1–2).

During this period, the global age-standardized disability-adjusted life years (ASDR) and age-standardized YLD rates for KOA attributable to high BMI also increased. The ASDR rose from 32.35 per 100,000 population in 1990 (95% UI −2.815–92.951) to 45.93 per 100,000 population in 2021 (95% UI −4.36–128.33). Females consistently had a higher burden of KOA attributable to high BMI than males. In 2021, females had 1.8 times the DALYs and YLDs of males: 2,559,536.58 years (95% UI −243,349.424–7,130,008.32) vs. males with 1,460,018.33 years (95% UI −139,208.8–4,092,309.6). The ASDR and age-standardized YLD rates for KOA attributable to high BMI in both females and males increased, similar to the overall population trend. In 2021, females had 1.6 times the rate of males: 56 per 100,000 population (95% UI −5.3–155.7) vs. males with 35 per 100,000 population (95% UI −3.3–98.3) (Figure 1B; Appendix, pp. 2–3).

By 2050, the global DALYs and YLDs attributable to high BMI for KOA are projected to nearly double from 2021 levels, reaching 7,406,988.2 years (95% UI 5,772,089.6–9,041,886.8). Among females, DALYs and YLDs are projected to nearly double compared to 2021, reaching 4,745,401.6 years (95% UI 3,832,735.4–5,658,067.9), remaining significantly higher than those of males, which are projected to be 2,845,449.4 years (95% UI 2,388,332.8–3,302,566.0). In 2050, females are projected to have nearly 1.7 times the DALYs and YLDs of males (Figure 1A; Appendix, pp. 3–4).

Similarly, the global ASDR and age-standardized YLD rates for KOA attributable to high BMI are projected to increase to 59.3 per 100,000 population by 2050 (95% UI 50.6–68.1), 1.3 times that of 2021. In 2050, females are projected to have 68.2 per 100,000 population (95% UI 49.4–86.9) vs. males with 46.6 per 100,000 population (95% UI 40.9–52.2) (Figure 1B; Appendix, pp. 4–5).

These trends highlight the multifactorial nature of gender differences in the KOA burden, driven by differences in adipose tissue distribution, hormonal fluctuations (particularly during menopause), and muscle mass changes. Women typically have a higher body fat percentage, particularly subcutaneous fat, and are more prone to obesity-related joint inflammation. Additionally, estrogen plays a key role in cartilage protection, and its decline in postmenopausal women accelerates the pathological process of KOA, contributing to a higher burden in females compared to males.

### 3.2 Regional and sex-specific trends of knee osteoarthritis (KOA) attributable to high body mass index (BMI) from 1990 to 2021

Across all global regions, stratified by SDI quintile, DALYs and YLDs due to KOA attributable to high BMI have shown an upward trend. The most pronounced increase in age-standardized DALY rate and age-standardized YLD rate was observed in low-middle SDI countries, with an EAPC of ASDR and EAPC of AS YLD rate of 2 (1.96–2.04). The age-standardized DALY rate and age-standardized YLD rate rose from 18 (−1.5–53.7) per 100,000 in 1990 to 32 (−2.8–90.8) per 100,000 in 2021. In contrast, the smallest increase was seen in high SDI countries, with an EAPC of ASDR and EAPC of AS YLD rate of 0.67 (0.62–0.73), where the rates increased from 45.8 (−4.2–128) per 100,000 in 1990 to 57.8 (−5.9–155.8) per 100,000 in 2021. In 2021, regions with middle SDI had higher DALYs and YLDs due to KOA attributable to high BMI. At the regional level, from 1990 to 2021, the global age-standardized DALY rate and YLD rate due to KOA attributable to high BMI showed an upward trend. The largest increase was observed in South Asia, where the rate rose from 12.3 (−0.9–36.8) per 100,000 in 1990 to 25.8 (−2–75.3) per 100,000 in 2021, with an EAPC of 2.61 (2.54–2.69). Similar significant increases were seen in East Asia, where the rate increased from 26.4 (−2.2–78.7) per 100,000 to 50.4 (−4.5–144) per 100,000, with an EAPC of 2.45 (2.32–2.58), and in Southeast Asia, where the rate increased from 13.7 (−1–41.4) per 100,000 to 25.8 (−2–74.3) per 100,000, with an EAPC of 2.19 (2.12–2.25). The smallest increases were observed in High-income North America, with an EAPC of 0.34 (0.17–0.51), Central Asia, with an EAPC of 0.6 (0.59–0.6), and Central Europe, with an EAPC of 0.63 (0.61–0.64). Across all regions, females consistently had higher rates than males (Table 1).

**Table 1:** Regional age-standardized DALY rate and age-standardized YLD rate of knee osteoarthritis (KOA) attributable to high body mass index (BMI) from 1990 to 2021, and associated EAPC.

Location	Sex	DALY (95%UI)		DALY (95%UI)			YLDs (95%UI)		YLDs (95%UI)		
		Case, 1990	ASDR (per100000)	Case, 2021	ASDR (per100000)	EAPC of ASDR (95%CI)	Case, 1990	AS YLD rate(per1 00000)	Case, 2021	AS YLD rate (per100000)	EAPC of AS YLD rate (95%CI)
			1990		2021	1990-2021		1990		2021	1990-2021
Global	Both	1306589.3 (−114326.8–3742191)	32.3 (−2.8–93)	4019554.9 (−382558.2–11222318)	45.9 (−4.4–128.3)	1.21 (1.18–1.23)	1306589.3 (−114326.8–3742191)	32.3 (−2.8–93)	4019554.9 (−382558.2–11222318)	45.9 (−4.4–128.3)	1.21 (1.18–1.23)
	Male	459435.3 (−40730.3–1318800.5)	24 (−2.1–69.2)	1460018.3 (−139208.8–4092309.6)	35 (−3.3–98.3)	1.29 (1.27–1.31)	459435.3 (−40730.3–1318800.5)	24 (−2.1–69.2)	1460018.3 (−139208.8–4092309.6)	35 (−3.3–98.3)	1.29 (1.27–1.31)
	Female	847154.1 (−73596.5–2424498)	39.6 (−3.4–113.5)	2559536.6 (−243349.4–7130008.3)	56 (−5.3–155.7)	1.19 (1.16–1.22)	847154.1 (−73596.5–2424498)	39.6 (−3.4–113.5)	2559536.6 (−243349.4–7130008.3)	56 (−5.3–155.7)	1.19 (1.16–1.22)
High SDI	Both	495250.8 (−45671.8–1387650)	45.8 (−4.2–128)	1100287.8 (−110415.5–2997645.5)	57.8 (−5.9–155.8)	0.67 (0.62–0.73)	495250.8 (−45671.8–1387650)	45.8 (−4.2–128)	1100287.8 (−110415.5–2997645.5)	57.8 (−5.9–155.8)	0.67 (0.62–0.73)
	Male	173456.1 (−17007.7–477329.5)	36.2 (−3.5–99.9)	418220.9 (−43867.4–1128251.7)	46.9 (−5–125.7)	0.77 (0.71–0.83)	173456.1 (−17007.7–477329.5)	36.2 (−3.5–99.9)	418220.9 (−43867.4–1128251.7)	46.9 (−5–125.7)	0.77 (0.71–0.83)
	Female	321794.7 (−28664.1–909185.5)	53.4 (−4.8–150.2)	682066.9 (−66548.1–1862294.5)	67.8 (−6.7–183.5)	0.69 (0.64–0.74)	321794.7 (−28664.1–909185.5)	53.4 (−4.8–150.2)	682066.9 (−66548.1–1862294.5)	67.8 (−6.7–183.5)	0.69 (0.64–0.74)
High–middle SDI	Both	354301 (−31810.5–1001324.8)	35 (−3.1–99.3)	1006830.2 (−98796.7–2771002.4)	50.8 (−5–140)	1.32 (1.27–1.36)	354301 (−31810.5–1001324.8)	35 (−3.1–99.3)	1006830.2 (−98796.7–2771002.4)	50.8 (−5–140)	1.32 (1.27–1.36)
	Male	119784.7 (−10472.9–341816.5)	26.4 (−2.3–76.1)	348908 (−33701.7–964222.1)	37.8 (−3.6–104.9)	1.26 (1.22–1.31)	119784.7 (−10472.9–341816.5)	26.4 (−2.3–76.1)	348908 (−33701.7–964222.1)	37.8 (−3.6–104.9)	1.26 (1.22–1.31)
	Female	234516.3 (−21337.6–659911.7)	41.9 (−3.8–117.8)	657922.2 (−65095–1802541)	62.4 (−6.2–170.4)	1.4 (1.36–1.45)	234516.3 (−21337.6–659911.7)	41.9 (−3.8–117.8)	657922.2 (−65095–1802541)	62.4 (−6.2–170.4)	1.4 (1.36–1.45)
Middle SDI	Both	299842.4 (−24685.9–873540.6)	27 (−2.2–79.2)	1281261.4 (−118656.9–3627905.7)	45.1 (−4.1–128.3)	1.85 (1.78–1.92)	299842.4 (−24685.9–873540.6)	27 (−2.2–79.2)	1281261.4 (−118656.9–3627905.7)	45.1 (−4.1–128.3)	1.85 (1.78–1.92)
	Male	108613.2 (−8885.9–319323.8)	19.7 (−1.6–58.2)	453644.2 (−41535.4–1287475.8)	33.1 (−3–94.4)	1.84 (1.77–1.91)	108613.2 (−8885.9–319323.8)	19.7 (−1.6–58.2)	453644.2 (−41535.4–1287475.8)	33.1 (−3–94.4)	1.84 (1.77–1.91)
	Female	191229.2 (−15806.5–552124.7)	34.1 (−2.8–98.9)	827617.3 (−77121.5–2337235.8)	56.3 (−5.2–159.6)	1.82 (1.75–1.89)	191229.2 (−15806.5–552124.7)	34.1 (−2.8–98.9)	827617.3 (−77121.5–2337235.8)	56.3 (−5.2–159.6)	1.82 (1.75–1.89)
Low–middle SDI	Both	117440.1 (−9621.4–345909.8)	18 (−1.5–53.7)	489047.8 (−43506–1379407.2)	32 (−2.8–90.8)	2 (1.96–2.04)	117440.1 (−9621.4–345909.8)	18 (−1.5–53.7)	489047.8 (−43506–1379407.2)	32 (−2.8–90.8)	2 (1.96–2.04)
	Male	42942.3 (−3426.4–126191.4)	13 (−1–38.7)	182079 (−15680.8–523712.8)	24.6 (−2.1–71.5)	2.16 (2.13–2.2)	42942.3 (−3426.4–126191.4)	13 (−1–38.7)	182079 (−15680.8–523712.8)	24.6 (−2.1–71.5)	2.16 (2.13–2.2)
	Female	74497.8 (−6202.2–219718.4)	23.2 (−1.9–68.7)	306968.8 (−27825.1–856687.6)	38.9 (−3.5–109)	1.83 (1.78–1.88)	74497.8 (−6202.2–219718.4)	23.2 (−1.9–68.7)	306968.8 (−27825.1–856687.6)	38.9 (−3.5–109)	1.83 (1.78–1.88)
Low SDI	Both	38129.3 (−2840.5–115086.4)	15.6 (−1.1–47.7)	138626 (−11126.9–407357.8)	24.6 (−1.9–73.6)	1.54 (1.5–1.58)	38129.3 (−2840.5–115086.4)	15.6 (−1.1–47.7)	138626 (−11126.9–407357.8)	24.6 (−1.9–73.6)	1.54 (1.5–1.58)
	Male	14018.7 (−1053.4–43012.9)	11.3 (−0.8–34.9)	55807 (−4395.3–166681)	20.2 (−1.6–60.8)	1.92 (1.89–1.95)	14018.7 (−1053.4–43012.9)	11.3 (−0.8–34.9)	55807 (−4395.3–166681)	20.2 (−1.6–60.8)	1.92 (1.89–1.95)
	Female	24110.5 (−1806.7–71641.3)	20 (−1.5–60.6)	82819.1 (−6731.6–240036.3)	29 (−2.3–85.6)	1.25 (1.21–1.3)	24110.5 (−1806.7–71641.3)	20 (−1.5–60.6)	82819.1 (−6731.6–240036.3)	29 (−2.3–85.6)	1.25 (1.21–1.3)
Andean Latin America	Both	9232.1 (−859.7–25388.5)	43.2 (−4–119.3)	36929.3 (−3943.9–98014.6)	61 (−6.5–162.8)	1.16 (1.13–1.19)	9232.1 (−859.7–25388.5)	43.2 (−4–119.3)	36929.3 (−3943.9–98014.6)	61 (−6.5–162.8)	1.16 (1.13–1.19)
	Male	3463.1 (−304.6–9531)	33.2 (−2.9–92.9)	14064.6 (−1489.7–37249.6)	48.3 (−5.1–128.9)	1.19 (1.14–1.23)	3463.1 (−304.6–9531)	33.2 (−2.9–92.9)	14064.6 (−1489.7–37249.6)	48.3 (−5.1–128.9)	1.19 (1.14–1.23)
	Female	5769.1 (−555.2–15736.9)	52.8 (−5–144.4)	22864.7 (−2454.2–60947.1)	72.8 (−7.8–194.7)	1.14 (1.1–1.18)	5769.1 (−555.2–15736.9)	52.8 (−5–144.4)	22864.7 (−2454.2–60947.1)	72.8 (−7.8–194.7)	1.14 (1.1–1.18)
Australasia	Both	11502.7 (−1100.9–31559.1)	49.8 (−4.8–136.4)	33628.7 (−3413.9–91300.3)	68.4 (−7.1–183.9)	1.02 (0.97–1.07)	11502.7 (−1100.9–31559.1)	49.8 (−4.8–136.4)	33628.7 (−3413.9–91300.3)	68.4 (−7.1–183.9)	1.02 (0.97–1.07)
	Male	4259 (−430.5–11704.6)	39.6 (−4–108.7)	13312 (−1399.9–36046.3)	56.8 (−6.1–153.1)	1.17 (1.11–1.22)	4259 (−430.5–11704.6)	39.6 (−4–108.7)	13312 (−1399.9–36046.3)	56.8 (−6.1–153.1)	1.17 (1.11–1.22)
	Female	7243.7 (−670.4–20033.7)	58.8 (−5.5–161.6)	20316.7 (−2014–54976.1)	79.1 (−8–212.6)	0.96 (0.91–1.01)	7243.7 (−670.4–20033.7)	58.8 (−5.5–161.6)	20316.7 (−2014–54976.1)	79.1 (−8–212.6)	0.96 (0.91–1.01)
Caribbean	Both	10470.6 (−913.1–29732.8)	39.8 (−3.5–113.3)	29874.4 (−2979.9–80681.6)	55.3 (−5.5–149.5)	1.12 (1.08–1.16)	10470.6 (−913.1–29732.8)	39.8 (−3.5–113.3)	29874.4 (−2979.9–80681.6)	55.3 (−5.5–149.5)	1.12 (1.08–1.16)
	Male	3898.8 (−339.2–11174.3)	30.5 (−2.6–87.9)	11548.8 (−1099.4–31909.5)	45.1 (−4.3–124.6)	1.33 (1.28–1.37)	3898.8 (−339.2–11174.3)	30.5 (−2.6–87.9)	11548.8 (−1099.4–31909.5)	45.1 (−4.3–124.6)	1.33 (1.28–1.37)
	Female	6571.8 (−573.9–18557.4)	48.6 (−4.2–137.3)	18325.6 (−1880.5–49380.3)	64.6 (−6.6–173.9)	0.98 (0.95–1.01)	6571.8 (−573.9–18557.4)	48.6 (−4.2–137.3)	18325.6 (−1880.5–49380.3)	64.6 (−6.6–173.9)	0.98 (0.95–1.01)
Central Asia	Both	14277.4 (−1338.7–39307.3)	30 (−2.8–83.3)	31164.5 (−3342.2–82425.6)	36 (−3.8–96)	0.6 (0.59–0.6)	14277.4 (−1338.7–39307.3)	30 (−2.8–83.3)	31164.5 (−3342.2–82425.6)	36 (−3.8–96)	0.6 (0.59–0.6)
	Male	4936.6 (−450.4–14081.7)	24.4 (−2.2–71.1)	11827.6 (−1232.6–31808.3)	30.2 (−3–82.5)	0.7 (0.69–0.71)	4936.6 (−450.4–14081.7)	24.4 (−2.2–71.1)	11827.6 (−1232.6–31808.3)	30.2 (−3–82.5)	0.7 (0.69–0.71)
	Female	9340.8 (−888.2–25181.6)	33.8 (−3.2–91.2)	19336.9 (−2109.7–50696.6)	40.5 (−4.3–106.2)	0.59 (0.58–0.6)	9340.8 (−888.2–25181.6)	33.8 (−3.2–91.2)	19336.9 (−2109.7–50696.6)	40.5 (−4.3–106.2)	0.59 (0.58–0.6)
Central Europe	Both	55300.2 (−5158.1–151934.6)	36.6 (−3.4–100.8)	92400.8 (−9580.7–250858.8)	44.2 (−4.6–118.5)	0.63 (0.61–0.64)	55300.2 (−5158.1–151934.6)	36.6 (−3.4–100.8)	92400.8 (−9580.7–250858.8)	44.2 (−4.6–118.5)	0.63 (0.61–0.64)
	Male	22037.4 (−2149.3–59747.7)	33.1 (−3.2–90.5)	36748.6 (−3935.9–98522.1)	39.7 (−4.3–106.2)	0.59 (0.58–0.6)	22037.4 (−2149.3–59747.7)	33.1 (−3.2–90.5)	36748.6 (−3935.9–98522.1)	39.7 (−4.3–106.2)	0.59 (0.58–0.6)
	Female	33262.8 (−3008.8–90856.1)	39.2 (−3.6–107.1)	55652.1 (−5644.8–152570.2)	48 (−4.9–129)	0.67 (0.66–0.69)	33262.8 (−3008.8–90856.1)	39.2 (−3.6–107.1)	55652.1 (−5644.8–152570.2)	48 (−4.9–129)	0.67 (0.66–0.69)
Central Latin America	Both	41258.5 (−3993.1–113395.4)	47.4 (−4.5–131.2)	162075.5 (−17703.4–428799.1)	62.9 (−6.8–167.2)	0.91 (0.9–0.93)	41258.5 (−3993.1–113395.4)	47.4 (−4.5–131.2)	162075.5 (−17703.4–428799.1)	62.9 (−6.8–167.2)	0.91 (0.9–0.93)
	Male	16068.3 (−1537.4–44306.3)	38.1 (−3.6–105.7)	64070.9 (−6822–170685.2)	53.7 (−5.7–144.1)	1.1 (1.08–1.12)	16068.3 (−1537.4–44306.3)	38.1 (−3.6–105.7)	64070.9 (−6822–170685.2)	53.7 (−5.7–144.1)	1.1 (1.08–1.12)
	Female	25190.2 (−2455.8–69099.8)	56.1 (−5.4–154.8)	98004.6 (−10881.4–257343.3)	71 (−7.8–186.9)	0.76 (0.75–0.77)	25190.2 (−2455.8–69099.8)	56.1 (−5.4–154.8)	98004.6 (−10881.4–257343.3)	71 (−7.8–186.9)	0.76 (0.75–0.77)
Central Sub–Saharan Africa	Both	4411.3 (−313.8–12921.6)	17.9 (−1.3–53.1)	19331.5 (−1520.3–56685.2)	31.2 (−2.4–91.8)	1.77 (1.73–1.81)	4411.3 (−313.8–12921.6)	17.9 (−1.3–53.1)	19331.5 (−1520.3–56685.2)	31.2 (−2.4–91.8)	1.77 (1.73–1.81)
	Male	1651.8 (−122.1–4790.5)	14.4 (−1.1–42.3)	7972.4 (−616.5–23553.8)	27.3 (−2–81.8)	2.07 (2.01–2.12)	1651.8 (−122.1–4790.5)	14.4 (−1.1–42.3)	7972.4 (−616.5–23553.8)	27.3 (−2–81.8)	2.07 (2.01–2.12)
	Female	2759.5 (−191.6–8128.3)	21.1 (−1.4–62.8)	11359.1 (−903.8–32643.3)	34.5 (−2.7–99.4)	1.59 (1.54–1.64)	2759.5 (−191.6–8128.3)	21.1 (−1.4–62.8)	11359.1 (−903.8–32643.3)	34.5 (−2.7–99.4)	1.59 (1.54–1.64)
East Asia	Both	246075.1 (−20248.1–729190.6)	26.4 (−2.2–78.7)	1147086.1 (−103527.9–3261439.2)	50.4 (−4.5–144)	2.45 (2.32–2.58)	246075.1 (−20248.1–729190.6)	26.4 (−2.2–78.7)	1147086.1 (−103527.9–3261439.2)	50.4 (−4.5–144)	2.45 (2.32–2.58)
	Male	84008.9 (−7021.3–248368.5)	17.7 (−1.5–53)	373793.8 (−32971.2–1074522.5)	33.5 (−2.9–96.7)	2.41 (2.26–2.55)	84008.9 (−7021.3–248368.5)	17.7 (−1.5–53)	373793.8 (−32971.2–1074522.5)	33.5 (−2.9–96.7)	2.41 (2.26–2.55)
	Female	162066.2 (−13228.2–478114.1)	34.8 (−2.8–102.9)	773292.2 (−70556.7–2186916.6)	66.6 (−6.1–188.8)	2.44 (2.32–2.57)	162066.2 (−13228.2–478114.1)	34.8 (−2.8–102.9)	773292.2 (−70556.7–2186916.6)	66.6 (−6.1–188.8)	2.44 (2.32–2.57)
Eastern Europe	Both	106117 (−9992.1–292045.7)	37.6 (−3.5–103.7)	164855.9 (−17051.3–438191.7)	47.8 (−5–126.7)	0.84 (0.82–0.86)	106117 (−9992.1–292045.7)	37.6 (−3.5–103.7)	164855.9 (−17051.3–438191.7)	47.8 (−5–126.7)	0.84 (0.82–0.86)
	Male	33206 (−2792.1–95141.8)	31.9 (−2.6–92.6)	57527.4 (−5741.8–153957.9)	42.4 (−4.2–113.5)	0.96 (0.94–0.99)	33206 (−2792.1–95141.8)	31.9 (−2.6–92.6)	57527.4 (−5741.8–153957.9)	42.4 (−4.2–113.5)	0.96 (0.94–0.99)
	Female	72911 (−7199.9–196568)	41.1 (−4–110.4)	107328.5 (−11345.9–284233.8)	51.5 (−5.6–135.4)	0.8 (0.77–0.82)	72911 (−7199.9–196568)	41.1 (−4–110.4)	107328.5 (−11345.9–284233.8)	51.5 (−5.6–135.4)	0.8 (0.77–0.82)
Eastern Sub–Saharan Africa	Both	13222.2 (−983.9–40326.8)	16.4 (−1.2–50.5)	49165.9 (−3898.1–142238.2)	26.1 (−2–76.8)	1.54 (1.52–1.56)	13222.2 (−983.9–40326.8)	16.4 (−1.2–50.5)	49165.9 (−3898.1–142238.2)	26.1 (−2–76.8)	1.54 (1.52–1.56)
	Male	4851.8 (−370.4–14896.2)	12.1 (−0.9–37.4)	19363 (−1516.8–57360.4)	21.4 (−1.6–63.9)	1.84 (1.81–1.87)	4851.8 (−370.4–14896.2)	12.1 (−0.9–37.4)	19363 (−1516.8–57360.4)	21.4 (−1.6–63.9)	1.84 (1.81–1.87)
	Female	8370.4 (−619.7–25124.5)	20.6 (−1.5–62.8)	29802.9 (−2381.3–84893.8)	30.5 (−2.4–88)	1.3 (1.29–1.32)	8370.4 (−619.7–25124.5)	20.6 (−1.5–62.8)	29802.9 (−2381.3–84893.8)	30.5 (−2.4–88)	1.3 (1.29–1.32)
High–income Asia Pacific	Both	76718.5 (−6200.4–227321.7)	37.1 (−3–109.8)	186168.2 (−14599.6–554015.1)	47.5 (−3.8–142)	0.84 (0.82–0.86)	76718.5 (−6200.4–227321.7)	37.1 (−3–109.8)	186168.2 (−14599.6–554015.1)	47.5 (−3.8–142)	0.84 (0.82–0.86)
	Male	23414.4 (−1950.6–68980.3)	24.7 (−2.1–73.3)	61514.6 (−4981.8–180441.4)	34.7 (−2.9–103.2)	1.17 (1.12–1.22)	23414.4 (−1950.6–68980.3)	24.7 (−2.1–73.3)	61514.6 (−4981.8–180441.4)	34.7 (−2.9–103.2)	1.17 (1.12–1.22)
	Female	53304.1 (−4254–157776)	46.7 (−3.7–138.2)	124653.6 (−9666.5–371067.9)	59 (−4.7–176.6)	0.77 (0.76–0.79)	53304.1 (−4254–157776)	46.7 (−3.7–138.2)	124653.6 (−9666.5–371067.9)	59 (−4.7–176.6)	0.77 (0.76–0.79)
High–income North America	Both	190173.5 (−18667.9–515742.2)	57.1 (−5.7–153.7)	421001 (−46372.6–1105960.6)	69 (−7.7–180.5)	0.34 (0.17–0.51)	190173.5 (−18667.9–515742.2)	57.1 (−5.7–153.7)	421001 (−46372.6–1105960.6)	69 (−7.7–180.5)	0.34 (0.17–0.51)
	Male	70042.8 (−7469–185773.1)	47.4 (−5.1–125.5)	167945.3 (−19251.9–440101.1)	58.9 (−6.8–154.1)	0.47 (0.29–0.65)	70042.8 (−7469–185773.1)	47.4 (−5.1–125.5)	167945.3 (−19251.9–440101.1)	58.9 (−6.8–154.1)	0.47 (0.29–0.65)
	Female	120130.7 (−11198.9–326998.7)	64.8 (−6.1–174)	253055.7 (−27120.7–665859.5)	78.1 (−8.5–204.2)	0.32 (0.16–0.48)	120130.7 (−11198.9–326998.7)	64.8 (−6.1–174)	253055.7 (−27120.7–665859.5)	78.1 (−8.5–204.2)	0.32 (0.16–0.48)
North Africa and Middle East	Both	67634.4 (−6583.3–187015.4)	37.7 (−3.6–105.2)	276033.5 (−32004.4–716880.2)	55.6 (−6.3–145.6)	1.25 (1.24–1.26)	67634.4 (−6583.3–187015.4)	37.7 (−3.6–105.2)	276033.5 (−32004.4–716880.2)	55.6 (−6.3–145.6)	1.25 (1.24–1.26)
	Male	27053.2 (−2443.1–76693.3)	29.5 (−2.6–85.1)	122069.4 (−13538.8–319325.3)	47.7 (−5.2–127.6)	1.54 (1.53–1.55)	27053.2 (−2443.1–76693.3)	29.5 (−2.6–85.1)	122069.4 (−13538.8–319325.3)	47.7 (−5.2–127.6)	1.54 (1.53–1.55)
	Female	40581.2 (−4140.1–109134.7)	46.2 (−4.6–125.2)	153964.1 (−18465.6–391736.6)	63.7 (−7.5–163.3)	1.05 (1.04–1.05)	40581.2 (−4140.1–109134.7)	46.2 (−4.6–125.2)	153964.1 (−18465.6–391736.6)	63.7 (−7.5–163.3)	1.05 (1.04–1.05)
Oceania	Both	1231.8 (−115.4–3433)	36.4 (−3.4–102.4)	4047 (−424.3–10835.1)	45.7 (−4.7–124.9)	0.7 (0.64–0.76)	1231.8 (−115.4–3433)	36.4 (−3.4–102.4)	4047 (−424.3–10835.1)	45.7 (−4.7–124.9)	0.7 (0.64–0.76)
	Male	468.9 (−41.9–1293.2)	26.4 (−2.3–74)	1574 (−162.7–4190)	33.8 (−3.4–92)	0.77 (0.7–0.83)	468.9 (−41.9–1293.2)	26.4 (−2.3–74)	1574 (−162.7–4190)	33.8 (−3.4–92)	0.77 (0.7–0.83)
	Female	762.9 (−73.5–2108.3)	47 (−4.4–131.6)	2473 (−261.6–6601.4)	58.4 (−6–157.9)	0.67 (0.61–0.73)	762.9 (−73.5–2108.3)	47 (−4.4–131.6)	2473 (−261.6–6601.4)	58.4 (−6–157.9)	0.67 (0.61–0.73)
South Asia	Both	78767.8 (−5754.3–232704.2)	12.3 (−0.9–36.8)	407367.7 (−32716.1–1185585)	25.8 (−2–75.3)	2.61 (2.54–2.69)	78767.8 (−5754.3–232704.2)	12.3 (−0.9–36.8)	407367.7 (−32716.1–1185585)	25.8 (−2–75.3)	2.61 (2.54–2.69)
	Male	28861.5 (−2146.9–84829.4)	8.8 (−0.6–26.4)	146255.4 (−11019.4–428663.9)	18.8 (−1.4–55)	2.62 (2.56–2.67)	28861.5 (−2146.9–84829.4)	8.8 (−0.6–26.4)	146255.4 (−11019.4–428663.9)	18.8 (−1.4–55)	2.62 (2.56–2.67)
	Female	49906.3 (−3607.3–147407.7)	16.3 (−1.2–48.7)	261112.3 (−21696.7–756921.2)	32.7 (−2.7–95.1)	2.51 (2.42–2.61)	49906.3 (−3607.3–147407.7)	16.3 (−1.2–48.7)	261112.3 (−21696.7–756921.2)	32.7 (−2.7–95.1)	2.51 (2.42–2.61)
Southeast Asia	Both	38835.3 (−2910.1–116462.7)	13.7 (−1–41.4)	187145.4 (−14924.5–535325.4)	25.8 (−2–74.3)	2.19 (2.12–2.25)	38835.3 (−2910.1–116462.7)	13.7 (−1–41.4)	187145.4 (−14924.5–535325.4)	25.8 (−2–74.3)	2.19 (2.12–2.25)
	Male	12584.7 (−946.7–37814.8)	9.3 (−0.7–28.3)	59822.7 (−4788.9–173452.5)	17.1 (−1.3–50)	2.1 (2.04–2.17)	12584.7 (−946.7–37814.8)	9.3 (−0.7–28.3)	59822.7 (−4788.9–173452.5)	17.1 (−1.3–50)	2.1 (2.04–2.17)
	Female	26250.5 (−1990.9–78498)	17.7 (−1.3–53.1)	127322.7 (−10135.6–362002.3)	33.6 (−2.6–95.8)	2.22 (2.16–2.28)	26250.5 (−1990.9–78498)	17.7 (−1.3–53.1)	127322.7 (−10135.6–362002.3)	33.6 (−2.6–95.8)	2.22 (2.16–2.28)
Southern Latin America	Both	23779 (−2332.1–63872.1)	50.9 (−5–136.9)	56673.8 (−6214.1–147174.2)	66.6 (−7.3–172.5)	0.87 (0.83–0.92)	23779 (−2332.1–63872.1)	50.9 (−5–136.9)	56673.8 (−6214.1–147174.2)	66.6 (−7.3–172.5)	0.87 (0.83–0.92)
	Male	8215.7 (−776–22307.4)	38.7 (−3.6–105.8)	20615.4 (−2295.4–53812.4)	53.5 (−5.9–139.7)	1.04 (0.97–1.1)	8215.7 (−776–22307.4)	38.7 (−3.6–105.8)	20615.4 (−2295.4–53812.4)	53.5 (−5.9–139.7)	1.04 (0.97–1.1)
	Female	15563.3 (−1556.1–41534.5)	61 (−6.1–162.3)	36058.5 (−3926.1–93749.1)	77.6 (−8.5–200.8)	0.8 (0.76–0.84)	15563.3 (−1556.1–41534.5)	61 (−6.1–162.3)	36058.5 (−3926.1–93749.1)	77.6 (−8.5–200.8)	0.8 (0.76–0.84)
Southern Sub–Saharan Africa	Both	10870 (−979.9–29915.9)	38.8 (−3.5–107.3)	32347 (−3314.3–85519.8)	53 (−5.4–142.5)	1.02 (1–1.04)	10870 (−979.9–29915.9)	38.8 (−3.5–107.3)	32347 (−3314.3–85519.8)	53 (−5.4–142.5)	1.02 (1–1.04)
	Male	3953.2 (−324.5–11303.2)	31.9 (−2.6–91.5)	13055.3 (−1184.6–35198.9)	49.7 (−4.5–135.9)	1.46 (1.44–1.49)	3953.2 (−324.5–11303.2)	31.9 (−2.6–91.5)	13055.3 (−1184.6–35198.9)	49.7 (−4.5–135.9)	1.46 (1.44–1.49)
	Female	6916.8 (−664.4–18390.4)	44.3 (−4.2–118.7)	19291.7 (−2129.7–50492.8)	55.6 (−6.1–146.5)	0.74 (0.73–0.76)	6916.8 (−664.4–18390.4)	44.3 (−4.2–118.7)	19291.7 (−2129.7–50492.8)	55.6 (−6.1–146.5)	0.74 (0.73–0.76)
Tropical Latin America	Both	40556.6 (−3638.8–112544.2)	42.3 (−3.7–118.1)	152298.1 (−15479.7–406398.5)	58 (−5.9–155.4)	1.04 (1.02–1.06)	40556.6 (−3638.8–112544.2)	42.3 (−3.7–118.1)	152298.1 (−15479.7–406398.5)	58 (−5.9–155.4)	1.04 (1.02–1.06)
	Male	15354.5 (−1393.7–43047.6)	33.6 (−3–94.8)	59474.8 (−6020.6–160028.5)	49.5 (−4.9–133.9)	1.28 (1.24–1.32)	15354.5 (−1393.7–43047.6)	33.6 (−3–94.8)	59474.8 (−6020.6–160028.5)	49.5 (−4.9–133.9)	1.28 (1.24–1.32)
	Female	25202.1 (−2255.7–69496.6)	50 (−4.4–139.3)	92823.3 (−9459.1–247560)	65.2 (−6.6–173.9)	0.88 (0.87–0.89)	25202.1 (−2255.7–69496.6)	50 (−4.4–139.3)	92823.3 (−9459.1–247560)	65.2 (−6.6–173.9)	0.88 (0.87–0.89)
Western Europe	Both	242629.4 (−22186.8–686592.3)	43.6 (−4–122.7)	445302.7 (−43687–1211417.3)	53.4 (−5.3–145)	0.64 (0.6–0.67)	242629.4 (−22186.8–686592.3)	43.6 (−4–122.7)	445302.7 (−43687–1211417.3)	53.4 (−5.3–145)	0.64 (0.6–0.67)
	Male	81572.4 (−7806.1–227294.3)	34 (−3.2–94.9)	164251.5 (−16658.4–448185.6)	42.8 (−4.4–115.4)	0.75 (0.71–0.79)	81572.4 (−7806.1–227294.3)	34 (−3.2–94.9)	164251.5 (−16658.4–448185.6)	42.8 (−4.4–115.4)	0.75 (0.71–0.79)
	Female	161057.1 (−14380.7–459203)	51 (−4.6–145)	281051.2 (−27028.6–761572.6)	63 (−6.1–171)	0.65 (0.61–0.69)	161057.1 (−14380.7–459203)	51 (−4.6–145)	281051.2 (−27028.6–761572.6)	63 (−6.1–171)	0.65 (0.61–0.69)
Western Sub–Saharan Africa	Both	23526 (−1899.3–69740.4)	25.3 (−2–75.7)	84658.1 (−7464.6–237627.9)	38.8 (−3.3–110)	1.37 (1.32–1.41)	23526 (−1899.3–69740.4)	25.3 (−2–75.7)	84658.1 (−7464.6–237627.9)	38.8 (−3.3–110)	1.37 (1.32–1.41)
	Male	9532.2 (−733.5–28714.1)	19.5 (−1.5–59.8)	33211.1 (−2881.4–94166)	32.2 (−2.7–92.2)	1.59 (1.52–1.66)	9532.2 (−733.5–28714.1)	19.5 (−1.5–59.8)	33211.1 (−2881.4–94166)	32.2 (−2.7–92.2)	1.59 (1.52–1.66)
	Female	13993.8 (−1165.8–40700.8)	31.5 (−2.6–92.3)	51447 (−4600.8–142638.7)	44.6 (−3.9–125.3)	1.11 (1.07–1.15)	13993.8 (−1165.8–40700.8)	31.5 (−2.6–92.3)	51447 (−4600.8–142638.7)	44.6 (−3.9–125.3)	1.11 (1.07–1.15)

### 3.3 Trends in KOA burden attributable to BMI across nations from 1990 to 2021

**Figure 2:**
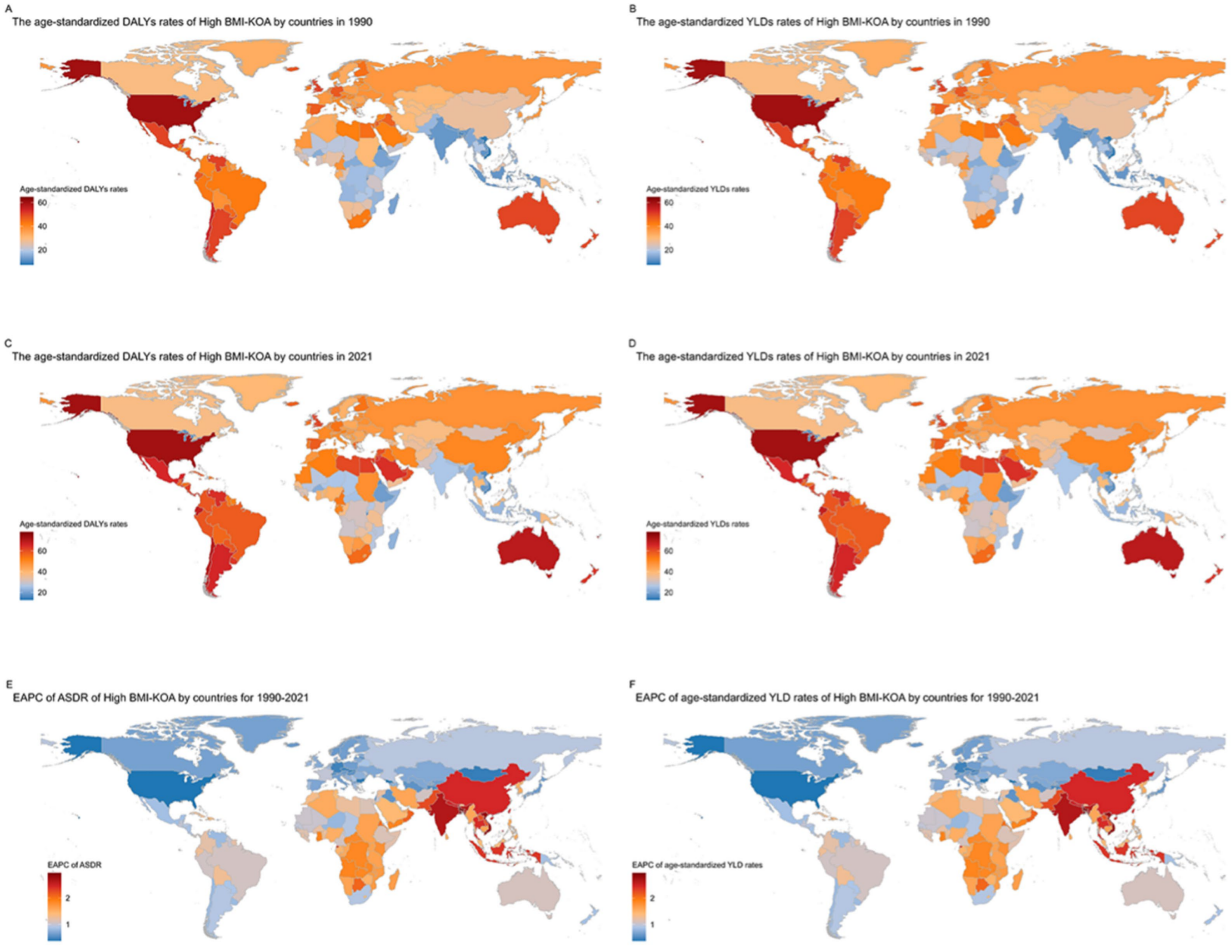
Trends in knee osteoarthritis (KOA) burden attributable to high body mass index (BMI) across nations from 1990 to 2021: **(A)** age-standardized DALY rates of knee osteoarthritis (KOA) attributable to high BMI; **(B)** age-standardized YLD rates of knee osteoarthritis (KOA) attributable to high BMI; **(C)** age-standardized DALY rates of knee osteoarthritis (KOA) attributable to high BMI; **(D)** age-standardized YLD rates; **(E)** EAPC of ASDR; **(F)** EAPC of the age-standardized YLD rates of knee osteoarthritis (KOA) attributable to High BMI.

In 1990, China, the United States of America, and the Russian Federation had the highest DALYs and YLDs due to KOA attributable to high BMI. China had 236,876.755 DALYs (−19,470.525–702,283.279), the United States of America had 180,832.776 DALYs (−17,765.996–489,880.801), and the Russian Federation had 67,931.6872 DALYs (−6,439.7781–187,587.661) (Appendix, pp. 5–53). In 1990, the highest age-standardized DALY rates and age-standardized YLD rates due to KOA attributable to high BMI were in American Samoa at 64.6678824 (−7.5020637–166.980647) per 100,000, Cook Islands at 61.7750792 (−6.8943726–159.510364) per 100,000, and Tonga at 60.7803285 (−6.5414137–158.75239) per 100,000. The lowest age-standardized DALY rates and age-standardized YLD rates were in Timor-Leste at 7.26348776 (−0.6348778–22.04757) per 100,000, Viet Nam at 7.6173424 (−0.605167–23.7718396) per 100,000, and Bangladesh at 9.45585908 (−0.7031136–27.8652144) per 100,000 (Figures 2A,B; Appendix, pp. 53–109).

In 2021, China, the United States of America, and India had the highest DALYs and YLDs due to KOA attributable to high BMI. China had 1,111,618.73 DALYs (−100,286.08–3,158,801.27), the United States of America had 396,242.964 DALYs (−44,029.824–1,040,246.77), and India had 326,778.158 DALYs (−26,038.59–948,113.631) (Appendix, pp. 109–157). In 2021, the highest age-standardized DALY rates and age-standardized YLD rates due to KOA attributable to high BMI were in Cook Islands at 78.2908649 (−9.5627646–198.299761) per 100,000, American Samoa at 77.2854677 (−9.4884426–194.820981) per 100,000, and Tonga at 76.0435441 (−9.6541836–194.212847) per 100,000. The lowest age-standardized DALY rates and age-standardized YLD rates were in Timor-Leste at 12.7570576 (−0.8483256–39.3654477) per 100,000, Viet Nam at 16.288187 (−1.1394476–49.1991348) per 100,000, and Burundi at 16.9653168 (−1.2319123–51.811576) per 100,000 (Figures 2C,D; Appendix, pp. 157–213).

Describing the increase and decrease according to EAPC: From 1990 to 2021, among 204 countries and territories, the age-standardized DALY rate and age-standardized YLD rate for KOA attributable to high BMI increased in 182 countries and territories. The largest increase was in Bangladesh, where the rate rose from 9.45585908 (−0.7031136–27.8652144) per 100,000 in 1990 to 22.0041481 (−1.7225288–64.7094495) per 100,000 in 2021; EAPC was 2.95 (2.36 to 3.55). In Viet Nam, the rate increased from 7.6173424 (−0.605167–23.7718396) per 100,000 in 1990 to 16.288187 (−1.1394476–49.1991348) per 100,000 in 2021; EAPC was 2.80 (2.19 to 3.41). In India, the rate rose from 12.0992959 (−0.8485698–36.008252) per 100,000 in 1990 to 25.8033923 (−2.0363028–75.0653199) per 100,000 in 2021; EAPC was 2.64 (2.06 to 3.22). The smallest increase was in the United States of America, where the rate rose from 60.0564398 (−5.9549506–161.54251) per 100,000 in 1990 to 72.8225921 (−8.1773562–190.230408) per 100,000 in 2021; EAPC was 0.38 (0.06 to 0.70). In Georgia, the rate increased from 31.4053848 (−2.8690556–87.2299779) per 100,000 in 1990 to 34.990576 (−3.5311226–94.2453592) per 100,000 in 2021; EAPC was 0.39 (0.20 to 0.58). In Bulgaria, the rate increased from 37.9405679 (−3.7163697–104.518177) per 100,000 in 1990 to 43.3229879 (−4.3110583–117.699675) per 100,000 in 2021; EAPC was 0.41 (0.26 to 0.57) (Figures 2E,F; Appendix, pp. 213–223).

### 3.4 Trends in KOA burden attributable to BMI across different age groups from 1990 to 2021

From 1990 to 2021, the global burden of knee osteoarthritis (KOA) attributable to high body mass index (BMI) increased significantly across all age groups, with the most pronounced increases observed in individuals aged 50 years and older.

**Figure 3:**
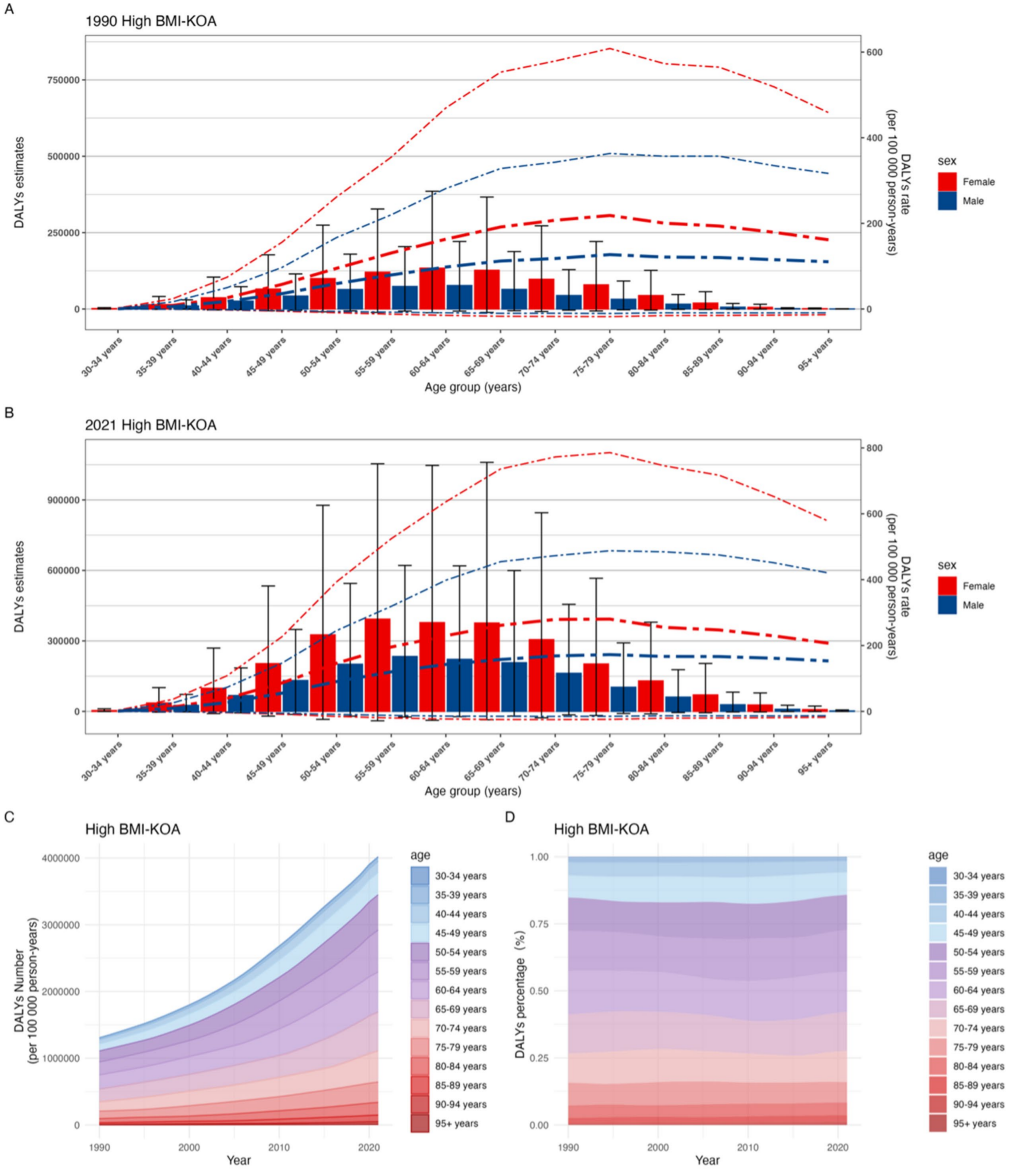
Trends in knee osteoarthritis (KOA) burden attributable to high body mass index (BMI) across different age groups from 1990 to 2021: **(A)** Global DALYs estimates of knee osteoarthritis (KOA) attributable to high BMI in 1990; **(B)** Global DALYs estimates of knee osteoarthritis (KOA) attributable to high BMI in 2021; **(C)** The trend in DALYs (per 100,000 population) of knee osteoarthritis (KOA) attributable to high BMI; **(D)** The change in the proportion of DALYs of knee osteoarthritis (KOA) attributable to high BMI.

In 1990, the disability-adjusted life years (DALYs) rate for KOA attributable to high BMI was lowest in the 30–34 years age group (0.696 per 100,000; 95% uncertainty interval [UI] −0.058 to 2.010) and highest in the 75–79 years age group (181.0 per 100,000, 95% UI −14.7 to 507.5). Females had a higher DALYs rate than males across all age groups, particularly in those aged 50–54 years and older. For instance, in the 60–64 years age group, females had 133,905 DALYs (95% UI −12,141 to 385,433), compared with 77,039 DALYs (95% UI −6,978 to 221,053) in males, with the total DALYs estimate reaching 210,944 (95% UI −19,101 to 606,486) for that year (Figure 3A; Appendix, pp. 223–227).

By 2021, the DALYs rate for KOA attributable to high BMI had significantly increased in age groups of 50–54 years and older. The highest DALYs rate was observed in the 75–79 years age group (231.4 per 100,000; 95% UI −19.9 to 650.5), with females having a higher rate (280.3 per 100,000, 95% UI −24.1 to 785.8) than males (172.5 per 100,000, 95% UI −14.9 to 487.8). The lowest DALYs rate remained in the 30–34 years age group (1.09 per 100,000, 95% UI −0.096 to 3.14), with females (1.28 per 100,000, 95% UI −0.114 to 3.70) having a higher rate than males (0.91 per 100,000, 95% UI −0.078 to 2.61) (Figure 3B; Appendix, pp. 231–234). DALYs estimates were higher across all age groups compared with 1990, with females continuing to have higher values than males. The highest DALYs estimates were in the 55–59 years age group, with females having 392,959 DALYs (95% UI −39,919 to 1,054,043) and males having 233,910 DALYs (95% UI −23,529 to 621,283), and the total estimate reaching 626,869 (95% UI −63,449 to 1,670,761) (Figure 3B; Appendix, pp. 234–237).

From 1990 to 2021, the proportion of DALYs in the 50 + age group increased slightly from approximately 85% to about 86% (Figure 3C). DALYs in the 45–79 years age group accounted for the main proportion across all ages, consistently and stably representing about 86% of the total burden from 1990 to 2021 (Figure 3D) (Appendix, pp. 237–247). The error bars on the bars represent the 95% uncertainty interval for the number of estimates.

**Figure 4:**
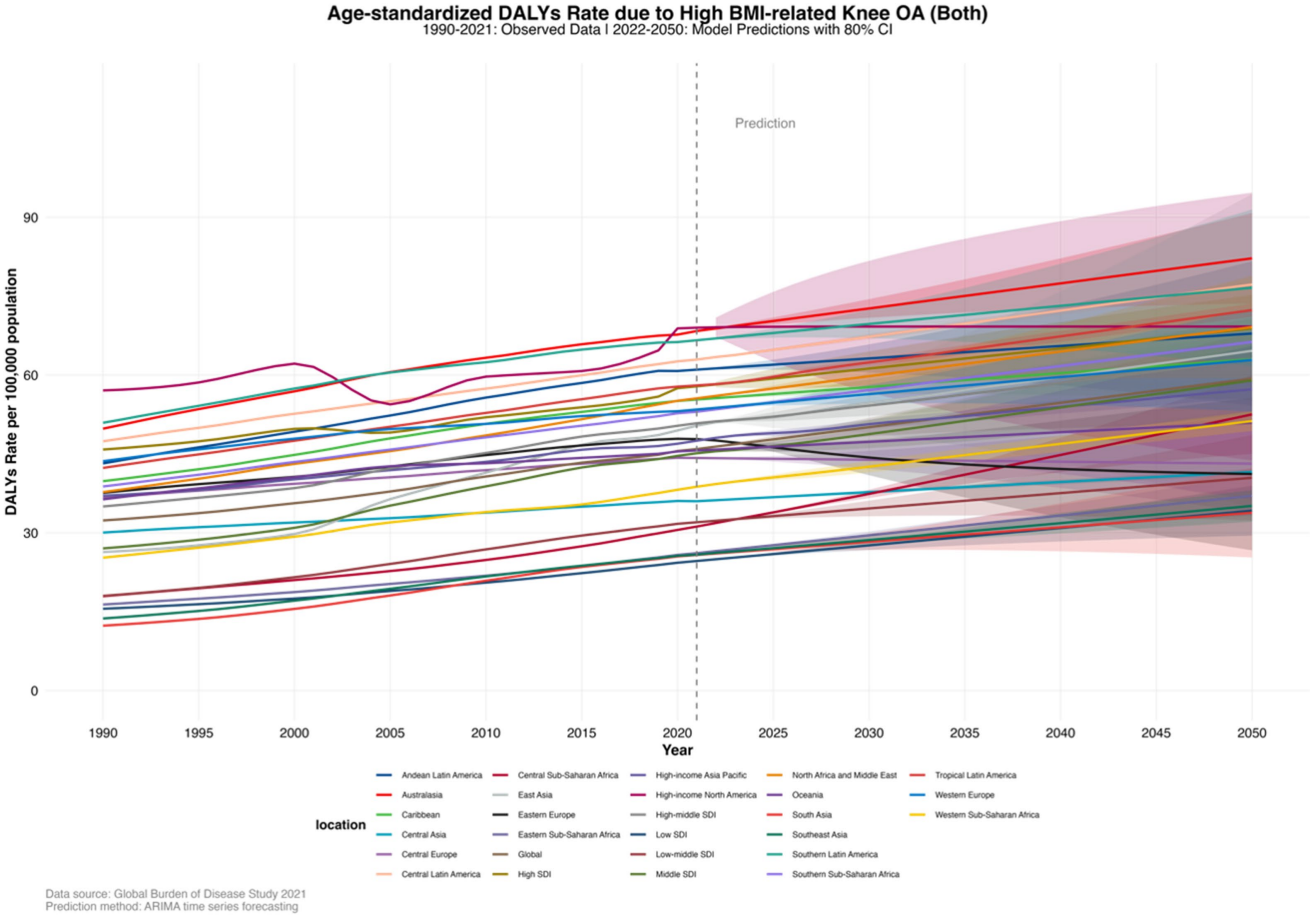
Age-standardized DALY rates of knee osteoarthritis (KOA) burden attributable to high body mass index (BMI) in 27 regions, forecasted to 2050.

In 2021, by SDI quintiles, the highest age-standardized DALYs rate was in High SDI countries at 57.8480556 (−5.9033337–155.753071), and the lowest was in Low SDI countries at 24.6392464 (−1.9357063–73.61697). At the regional level, the highest age-standardized DALYs rates were in High-income North America at 69.0039408 (−7.6861141–180.495099), Australasia at 68.375091 (−7.0705506–183.934162), and Southern Latin America at 66.6194451 (−7.3374808–172.499391). The lowest rates were in South Asia at 25.7856076 (−2.0491443–75.309209), Southeast Asia at 25.8385827 (−2.032275984–74.3322614), and Eastern Sub-Saharan Africa at 26.1305401 (−2.0293549–76.7992493). The age-standardized DALYs rate in most regions showed an upward trend, and this trend is projected to continue in the coming decades (Appendix, pp. 247–250). By 2050, by SDI quintiles, the highest age-standardized DALYs rate is projected to be in High SDI countries at 68.87355332 (62.51924111–75.22786554), and the lowest in Low SDI countries at 34.20333495 (29.45637533–38.95029456). By 2050, at the regional level, the highest age-standardized DALYs rates are projected to be in Australasia at 82.20691821 (73.60359844–90.81023799), Central Latin America at 77.23814059 (76.25639376–78.21988741), and Southern Latin America at 76.61358935 (61.72065656–91.50652215). The lowest rates are projected to be in South Asia at 33.81734541 (25.30984457–42.32484625), Southeast Asia at 35.11642903 (32.11202282–38.12083524), and Eastern Sub-Saharan Africa at 37.0297979 (33.21696842–40.84262739) (Appendix, pp. 251–253; Figure 4).

**Figure 5:**
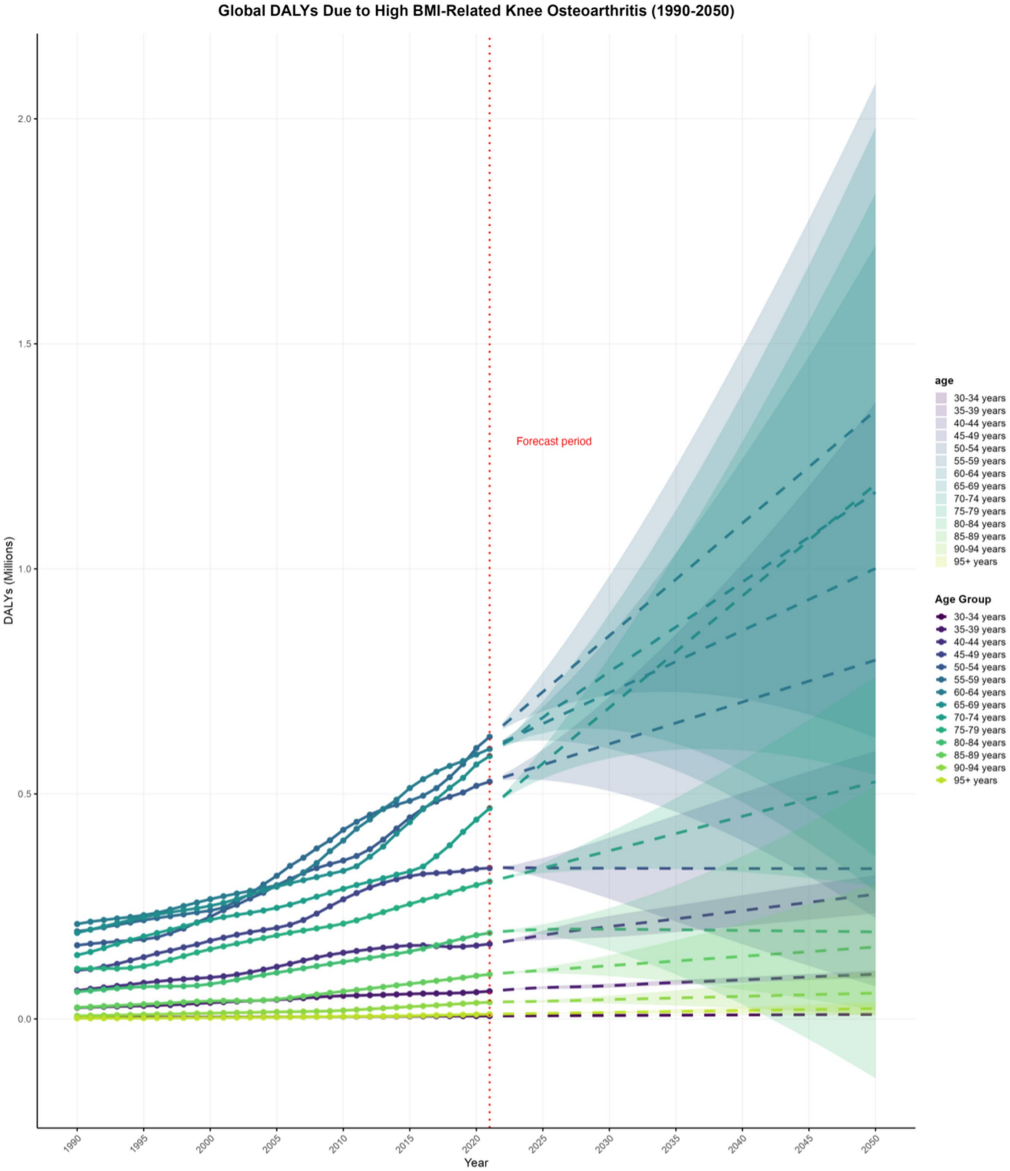
Projections to 2050 of the global knee osteoarthritis (KOA) burden attributable to high body mass index (BMI) across different age groups.

The DALYs due to KOA attributable to high BMI in most age groups globally have shown an upward trend, which is projected to continue in the coming decades. By 2050, globally, the highest DALYs due to KOA attributable to high BMI are projected to be in the 55–59 years age group at 1.351558876 million (0.624402664–2.078715088), and the lowest in the 30–34 years age group at 0.01024589 million (0.009833728–0.010658051) (Appendix, pp. 253–263; Figure 5).

## 4 Discussion

The knee joint is the most frequently affected site in osteoarthritis (16), knee osteoarthritis (KOA) is regarded as a disease that affects the entire joint, encompassing components like articular cartilage, menisci, ligaments, tendons, synovium, and subchondral bone (19). As a heterogeneous disease, it has become increasingly prevalent because of aging and obesity, thereby imposing a significant burden on global health (20). The incidence of knee osteoarthritis rises with increasing age, and females have a higher prevalence than males (21). Obesity-related knee osteoarthritis is a complex biopsychosocial disease that leads to increased morbidity and mortality in patients and imposes a significant economic burden on the healthcare system (22). Thus, conducting a global, regional, and national analysis of the disease burden in patients with knee osteoarthritis attributable to high body mass index (BMI) holds significant importance for clinical practice, epidemiology, and public health.

### 4.1 Analysis of the reasons for the significant increase in global burden and future trends

From 1990 to 2021, global DALYs and YLDs associated with knee osteoarthritis (KOA) due to high body mass index (BMI) saw a marked rise and are anticipated to double by 2050 (23). This significant increase is primarily driven by the continuous rise in global obesity rates. High BMI has emerged as a crucial risk factor for the development of KOA. In obesity, adipose tissue secretes a range of cytokines that exert local effects and influence systemic metabolism and inflammation through endocrine and paracrine mechanisms (24). These cytokines can directly impact joint health, potentially causing joint inflammation and degenerative changes (25). Obesity also changes the stress distribution and contact patterns of knee cartilage, which further increases the mechanical load on the knee joint. Dynamic joint loading, which occurs more frequently during walking, exacerbates the pathological process of KOA (26, 27). Additionally, obesity contributes to chronic metabolic syndrome (28), which can modify the polarization of macrophages in the synovial membrane and adipose tissue, facilitating inflammatory responses in the knee joint (29).

A prospective study has demonstrated that a weight loss exceeding 7.5% can markedly decrease the risk of total knee replacement surgery (30). Obesity is associated with elevated all-cause mortality in patients with KOA (31).

Controlling body weight can reduce the mechanical load on the knee joint and may slow the progression of KOA by improving metabolic and inflammatory status (32).

Furthermore, environmental pollutants, such as air pollution and exposure to endocrine disruptors, may significantly contribute to the exacerbation of knee osteoarthritis (KOA). Recent studies indicate that pollutants can aggravate systemic inflammation, which in turn accelerates joint degeneration. For instance, exposure to heavy metals has been linked to an increased risk of metabolic syndrome (MetS), which includes obesity, diabetes, and hypertension (33). These pollutants may contribute to the development of KOA through mechanisms such as oxidative stress, insulin resistance, and inflammation. Air pollution, particularly fine particulate matter (PM2.5), is a major factor in systemic inflammation. Studies have shown that PM2.5 exposure is associated with endothelial dysfunction and increased levels of pro-inflammatory cytokines such as IL-6 and TNF-*α*, which can exacerbate joint degeneration (34). Additionally, PM2.5 can induce oxidative stress, further contributing to the inflammatory process and accelerating the progression of KOA (35). Endocrine disruptors (EDCs) are another class of environmental pollutants that can interfere with hormonal regulation, leading to metabolic disorders and inflammation. Exposure to EDCs has been linked to metabolic syndrome, type 2 diabetes, and cardiovascular diseases, all of which are associated with increased systemic inflammation (36). These metabolic disorders and inflammatory responses can affect the activity of chondrocytes and the integrity of the extracellular matrix, thereby accelerating the progression of KOA (37). EDCs can also induce oxidative stress and promote the production of pro-inflammatory cytokines, further exacerbating joint inflammation (38). The combined effects of air pollution and EDCs can exacerbate KOA through multiple mechanisms. For example, PM2.5 and EDCs can activate Toll-like receptors (TLRs) and the NF-κB signaling pathway, enhancing inflammatory responses (39). This activation can lead to chondrocyte apoptosis and degradation of the extracellular matrix (35, 37). Additionally, metabolic syndrome and obesity, which are often associated with exposure to these pollutants, can increase joint load and promote inflammation, further worsening KOA (40, 41). Incorporating these factors into the analysis of KOA attributable to high BMI provides a more comprehensive understanding of the disease burden. Addressing these environmental factors through public health strategies and individual interventions is essential to mitigate the progression of KOA and reduce its overall burden (42, 43).

### 4.2 Sex-specific trends in the burden of knee osteoarthritis attributable to high BMI

Our analysis reveals that females consistently experience a higher burden of knee osteoarthritis (KOA) attributable to high body mass index (BMI) compared with males, a disparity that persists across global, regional, and national levels and is projected to continue through 2050. This sex-specific difference is multifactorial, involving adipose tissue distribution, hormonal changes, musculoskeletal aging, and behavioral patterns, all of which collectively contribute to the heightened susceptibility of females to KOA.

#### 4.2.1 Adipose tissue distribution and inflammatory microenvironment

Women typically have a higher overall body fat percentage, with a predominance of subcutaneous fat, whereas men tend to accumulate more visceral fat (44). The area of subcutaneous fat tissue (SCF) is twice that of men, accounting for 50% of total tissue content in women, compared with less than 25% in men (45). Both subcutaneous and intramuscular fat deposits, particularly those located in the patellar region and around the joint, are metabolically active and release adipokines and cytokines that promote chronic low-grade inflammation, cartilage degradation, and synovial pathology. These adipose tissues are not merely energy storage sites but also participate in metabolic regulation and inflammatory responses through the secretion of various bioactive substances. Studies have shown that subcutaneous adipose tissue (SAT) and intramuscular adipose tissue (IMAT) play crucial roles in obesity and metabolic syndrome, especially in inflammation and metabolic dysregulation (46–48).

The infrapatellar fat pad (IPFP), a specialized adipose tissue located within the knee joint and in close proximity to the synovium and cartilage, has been found to secrete a variety of pro-inflammatory cytokines and adipokines, such as interleukin-6 (IL-6) and tumor necrosis factor-*α* (TNF-α), which play important roles in knee joint inflammation and cartilage degradation (49–51). Moreover, the metabolic activity of the IPFP is closely related to knee joint health, and the adipokines and cytokines it secretes may influence the metabolic processes of the synovium and cartilage, thereby promoting the development of OA (52, 53).

Intramuscular adipose tissue (IMAT) also plays a significant role in metabolic disorders and inflammatory responses. Research indicates that PPARγ+ macrophages in IMAT are closely related to the metabolic state of surrounding adipocytes, and these macrophages significantly increase in number in obese states, potentially contributing to metabolic dysregulation through the secretion of pro-inflammatory factors (48, 54). Additionally, abnormal accumulation of IMAT is closely associated with pathological conditions such as muscle atrophy, functional decline, inflammation, and insulin resistance (54, 55).

#### 4.2.2 Menopausal transition and estrogen decline

Menopause marks a sharp decline in estrogen levels, weakening its protective effects on cartilage, bone, and inflammation. Estrogen promotes the synthesis of extracellular matrix, inhibits synovial inflammation, and maintains chondrocyte activity. Its deficiency accelerates cartilage aging, matrix degradation, and the production of pro-inflammatory SASP mediators (e.g., IL-6, MMP-13), which are key drivers of OA pathology (56).

Compared with men, women are more susceptible to OA, especially after menopause (57). The abrupt drop in estrogen levels post-menopause deprives cartilage of its protective effects, leading to degenerative changes and reduced repair capacity, thereby significantly increasing the risk of KOA in women (56). Estrogen has anti-inflammatory properties, inhibiting the release of inflammatory cytokines such as TNF-*α* and IL-1β, thereby alleviating pain caused by inflammatory reactions. The decline in estrogen levels exacerbates the destruction of articular cartilage and subchondral bone, disrupts the balance between osteoblasts and osteoclasts, and triggers cartilage erosion, destruction, and osteophyte formation, thus inducing pain (58).

#### 4.2.3 Postmenopausal sarcopenia, intramuscular fat, and metabolic shifts

Women generally have lower baseline muscle mass and strength than men, and this gap widens with age. Sarcopenia, characterized by loss of lean body mass and intramuscular fat infiltration, is more pronounced in postmenopausal women (59, 60), and is associated with impaired joint stability, altered biomechanics, and increased mechanical load on the knee joint, thereby accelerating KOA progression (61). The decline in muscle mass and function weakens the protective support of the knee joint, further exacerbating obesity-related degenerative changes.

#### 4.2.4 Sociocultural and lifestyle factors

In addition to biological factors, women are more likely to engage in household chores that require prolonged standing, climbing stairs, and caregiving (62, 63), which subject the knee joint to repetitive mechanical stress. Moreover, a relative lack of regular physical exercise may hinder muscle function enhancement and exacerbate joint instability (61). These behavioral patterns interact with hormonal and metabolic susceptibility, collectively explaining why women dominate the burden of KOA attributable to high BMI.

In summary, these endocrine, adipose distribution, musculoskeletal, and behavioral factors constitute a comprehensive mechanistic framework that elucidates why females bear a higher KOA disease burden attributable to high BMI across geographical and temporal dimensions.

### 4.3 The relationship between regional differences and the socio-demographic index (SDI)

The burden of osteoarthritis due to high BMI has seen a marked rise from 1990 to 2021, with low-middle SDI countries experiencing a particularly substantial increase (64). According to Ren, J. L. et al., the incidence and prevalence of knee osteoarthritis (KOA) are rising more quickly in low-middle SDI countries, which is closely associated with rapid urbanization and changes in lifestyle (65). These countries typically encounter problems like limited resources, insufficient health education, and inadequate medical services, which make high BMI one of the key drivers of the rising KOA burden (66). In low SDI regions, where the burden of KOA attributable to high BMI has risen significantly, healthcare resources are limited, which exacerbates the challenges in managing KOA. These countries face critical shortages in both infrastructure and medical professionals. For example, low SDI regions tend to have lower healthcare access, insufficient prevention programs, and limited diagnostic facilities. The lack of adequate medical resources in these regions significantly impacts the management of obesity and its related comorbidities, including KOA. Therefore, targeted interventions and healthcare investments are necessary to address these disparities and reduce the future burden of KOA in these regions.

In contrast, in high SDI countries, the burden of KOA is also on the rise, but the increase is relatively modest. This may be attributed to the more robust healthcare systems and effective public health policies in place, which aid in controlling BMI and reducing the incidence of related diseases (67). High SDI countries typically boast more robust healthcare systems and greater health awareness, enabling more effective weight management and control, and thus slowing the growth of the KOA burden (66). Zhao, G. et al. demonstrated that in high SDI countries, women bear a more significant burden of osteoarthritis due to high BMI, underscoring the importance of gender differences in the KOA burden. These differences are driven by multifactorial factors, including differences in adipose tissue distribution, hormonal fluctuations, particularly during menopause, and muscle mass changes. Women typically have a higher body fat percentage, particularly subcutaneous fat, and are more prone to obesity-related joint inflammation. Additionally, estrogen plays a key role in cartilage protection, and its decline in postmenopausal women accelerates the pathological process of KOA, contributing to a higher burden in females compared to males (68).

### 4.4 Analysis of the reasons for national trends

In 2021, China, the United States, and India had the highest burden of knee osteoarthritis (KOA) attributable to high body mass index (BMI). In the United States, around two-thirds of adults (69%) were overweight or obese, and it is predicted that nearly 50% of American adults will be obese by 2030 (24). Despite improvements in physical activity levels, the high consumption of ultra-processed foods continues to drive the obesity epidemic (69). African Americans and Latinos have higher obesity rates, especially in rural areas, where obesity-related mortality is also elevated (70). Moreover, in the US, the incidence of obesity is particularly high among younger individuals and those with lower educational attainment (71). The rate of disability-adjusted life years (DALYs) for KOA attributable to high BMI has exhibited a marked upward trend in both China and the USA (72). In China, the swift economic growth and rapid urbanization have led to the replacement of traditional diets with high-calorie, high-fat Western fast food, resulting in a rising obesity rate (73, 74). As India’s economy has rapidly developed, an increasing number of people can afford high-calorie and high-fat foods, resulting in a rising obesity rate (75). Higher socioeconomic status and education levels in India are linked to higher obesity rates, likely due to greater access to high-energy-density foods and more sedentary lifestyles (76, 77). Between 1990 and 2021, Bangladesh saw the largest increase in the burden of KOA attributable to high BMI. The rise in obesity rates in Bangladesh is related to urbanization, changes in socioeconomic status, higher education levels, and gender differences in the KOA burden. These differences are driven by multifactorial factors, including differences in adipose tissue distribution, hormonal fluctuations (particularly during menopause), and muscle mass changes. Women typically have a higher body fat percentage, particularly subcutaneous fat, and are more prone to obesity-related joint inflammation. Additionally, estrogen plays a key role in cartilage protection, and its decline in postmenopausal women accelerates the pathological process of KOA, contributing to a higher burden in females compared to males (78, 79).

### 4.5 The influence of age-related factors and targeted intervention populations

Globally, disability-adjusted life years (DALYs) attributable to knee osteoarthritis (KOA) are increasing across most age groups, with a particularly pronounced rise among individuals aged 50 years and older. This trend is underpinned by several age-related physiological changes. As people age, body weight often increases (21), while muscle strength and mass decline (80). This decline in muscle mass, known as sarcopenia, is accompanied by a reduction in muscle size and a rise in intramuscular fat content (81). Sarcopenia is recognized as a potential risk factor for KOA, especially in older adults. Research indicates that lower limb muscle mass and strength are strongly associated with the symptomatic presentation of KOA (82). The reduction in muscle mass not only impacts joint stability but also contributes to gradual cartilage degeneration (83). Among older adult women, a marked decrease in knee muscle strength is strongly associated with reduced knee torque (84). Moreover, in older adults, the combination of sarcopenia and obesity, termed sarcopenic obesity, is associated with a more pronounced deterioration in physical function, particularly in women (85). The combination of age and high body mass index (BMI), especially following an injury, significantly heightens the risk of accelerated KOA (AKOA) (86). Given these findings, targeted health education and weight management interventions should be enhanced for individuals over 50, particularly those with high BMI, to mitigate the progression of KOA and improve overall quality of life.

### 4.6 Analysis of the reasons for the 2050 predictions

By 2050, DALYs and YLDs related to knee osteoarthritis (KOA) due to high BMI are expected to nearly double. This projection is primarily driven by three major factors: the intensification of population aging, the continuous rise in obesity rates, and the uneven distribution of medical resources. The burden of KOA due to high BMI in women has consistently been higher than that in men, and this gender disparity is expected to persist. By 2050, the highest age-standardized DALYs rate is projected to be in high SDI regions, with the highest DALYs in the 55–59 years age group.

To address these challenges, comprehensive strategies are needed to strengthen health education and promotion globally, raise public awareness of the relationship between high BMI and KOA, and advocate for a healthy lifestyle, including balanced diets and increased physical activity. In addition to promoting a healthy lifestyle in general, region-specific interventions are needed. For example, in low-middle SDI regions, where obesity rates are rising alongside limited healthcare resources, targeted health education programs are essential. These programs should include weight management, physical activity promotion, and improved access to affordable healthcare. In contrast, high SDI regions should focus on obesity prevention through policy interventions that regulate food quality and encourage physical activity.

### 4.7 Limitations and future directions

Despite the comprehensive analysis of the global burden of knee osteoarthritis (KOA) due to high BMI from 1990 to 2021 using extensive data from the GBD 2021 study and the prediction of future trends, several limitations should be acknowledged. The accuracy and completeness of the data may be compromised due to variations in data collection and reporting systems across different countries and regions, leading to potential biases or missing data in certain areas. Additionally, while the GBD study provides robust estimates, the causal relationships between high BMI and KOA are complex and may be influenced by unmeasured confounders. In the present study, we employed population-attributable fractions (PAFs) based on a BMI of ≥25 kg/m^2^ to assess the impact of high BMI on the burden of KOA. However, relying solely on this single metric may have limitations. Recent retrospective cohort studies, such as those by Sun et al. (87, 88), have refined risk attribution models by incorporating physiological heterogeneity factors, including AFC, AMH, and blood type. Although Sun et al. (87) concluded that there was no significant association between ABO blood type and ovarian reserve, their approach highlights the importance of considering such physiological characteristics when interpreting large-scale data, thereby aiding in the construction of more precise risk attribution models. Furthermore, Sun et al. (88) underscored the necessity of integrating AFC and AMH into the assessment of ovarian response, revealing that the number of retrieved oocytes was positively correlated with AFC and AMH, but negatively correlated with age and protocol selection, with no significant correlation with BMI. These findings collectively suggest that incorporating physiological heterogeneity factors into risk attribution models can enhance the accuracy of interpreting health impact factors. Future studies should consider including such factors in sensitivity analyses to further refine the assessment of the KOA burden attributable to high BMI.

To address these challenges and mitigate the future burden of KOA due to high BMI, several evidence-based strategies are recommended. Strengthening global health education and public awareness campaigns to highlight the link between high BMI and KOA is essential. Region-specific interventions should be developed based on the socio-demographic index (SDI) to ensure targeted and effective approaches. For instance, in low SDI regions, where healthcare resources are particularly limited, targeted health education programs should focus on basic health literacy, weight management, and promoting physical activity. In low-middle SDI regions, where obesity rates are rising alongside limited healthcare resources, targeted health education programs should include weight management, physical activity promotion, and improved access to affordable healthcare. High SDI regions should prioritize obesity prevention through policy interventions that regulate food quality and encourage physical activity.

Furthermore, governments should develop policies to promote the availability and consumption of healthy foods, limit the excessive intake of high-calorie and high-fat foods, and increase investment in healthcare resources, particularly in low and low-middle SDI countries. These efforts will improve prevention, diagnosis, and treatment capabilities for KOA, thereby alleviating the burden of KOA due to high BMI.

## 5 Conclusions and summary

By analyzing the global burden of knee osteoarthritis (KOA) attributable to high body mass index (BMI) from 1990 to 2021 and projecting the trend to 2050, this study highlights the substantial influence of high BMI on the KOA burden and offers data support for managing the global burden of KOA due to high BMI. The findings underscore the need to focus on individuals aged 50 and older, females, and those in low SDI regions in the future, as these groups experience a more pronounced burden of KOA due to high BMI.

To effectively address this burden, we advocate for comprehensive public health strategies globally, with an emphasis on enhancing health education and awareness. Region-specific interventions should be developed based on the socio-demographic index (SDI) to ensure targeted and effective approaches. For example, in low SDI regions, targeted health education programs should emphasize basic health literacy, weight management, and promoting physical activity. In low-middle SDI regions, these programs should also include strategies to improve access to affordable healthcare. High SDI regions should implement policy interventions to regulate food quality and encourage physical activity, thereby promoting balanced diets and increased physical activity. These tailored interventions, based on regional SDI differences, are expected to be more effective than generalized suggestions for a “healthy lifestyle.”

By implementing these evidence-based, region-specific strategies, we can potentially reduce the incidence of KOA due to high BMI at its root. This approach not only addresses the immediate health concerns but also promotes long-term health benefits and reduces the overall burden of KOA globally.

## Data Availability

The original contributions presented in the study are included in the article/Supplementary material, further inquiries can be directed to the corresponding author.
