# Supplementary material for: Global, regional, and national burden of knee osteoarthritis attributable to high BMI: a systematic analysis from 1990 to 2021 and projections to 2050
Source: Front Public Health. 2025 Oct 15;13:1668257. doi: 10.3389/fpubh.2025.1668257 (PMC12568355; doi:10.3389/fpubh.2025.1668257)
Supplement: Supplementary file 1 [file Table_1.DOCX]

Appendix 1：Global DALYs and YLDs associated with knee osteoarthritis attributable to high body mass index, 1990–2021

| measure | location | sex | age | cause | rei | metric | year | val | upper | lower |
| --- | --- | --- | --- | --- | --- | --- | --- | --- | --- | --- |
| DALYs (Disability-Adjusted Life Years) | Global | Male | All ages | Osteoarthritis knee | High body-mass index | Number | 1990 | 459435.289 | 1318800.49 | -40730.293 |
| DALYs (Disability-Adjusted Life Years) | Global | Female | All ages | Osteoarthritis knee | High body-mass index | Number | 1990 | 847154.055 | 2424497.98 | -73596.493 |
| DALYs (Disability-Adjusted Life Years) | Global | Both | All ages | Osteoarthritis knee | High body-mass index | Number | 1990 | 1306589.34 | 3742191 | -114326.79 |
| DALYs (Disability-Adjusted Life Years) | Global | Male | All ages | Osteoarthritis knee | High body-mass index | Number | 2021 | 1460018.33 | 4092309.63 | -139208.8 |
| DALYs (Disability-Adjusted Life Years) | Global | Female | All ages | Osteoarthritis knee | High body-mass index | Number | 2021 | 2559536.58 | 7130008.32 | -243349.42 |
| DALYs (Disability-Adjusted Life Years) | Global | Both | All ages | Osteoarthritis knee | High body-mass index | Number | 2021 | 4019554.91 | 11222318 | -382558.22 |
| YLDs (Years Lived with Disability) | Global | Male | All ages | Osteoarthritis knee | High body-mass index | Number | 1990 | 459435.289 | 1318800.49 | -40730.293 |
| YLDs (Years Lived with Disability) | Global | Female | All ages | Osteoarthritis knee | High body-mass index | Number | 1990 | 847154.055 | 2424497.98 | -73596.493 |
| YLDs (Years Lived with Disability) | Global | Both | All ages | Osteoarthritis knee | High body-mass index | Number | 1990 | 1306589.34 | 3742191 | -114326.79 |
| YLDs (Years Lived with Disability) | Global | Male | All ages | Osteoarthritis knee | High body-mass index | Number | 2021 | 1460018.33 | 4092309.63 | -139208.8 |
| YLDs (Years Lived with Disability) | Global | Female | All ages | Osteoarthritis knee | High body-mass index | Number | 2021 | 2559536.58 | 7130008.32 | -243349.42 |
| YLDs (Years Lived with Disability) | Global | Both | All ages | Osteoarthritis knee | High body-mass index | Number | 2021 | 4019554.91 | 11222318 | -382558.22 |

Appendix 2：From 1990 to 2021, the global age-standardized disability-adjusted life years (ASDR) and age-standardized years lived with disability (YLD) rates for knee osteoarthritis due to high body mass index (BMI)

| measure | location | sex | age | cause | rei | metric | year | val | upper | lower |
| --- | --- | --- | --- | --- | --- | --- | --- | --- | --- | --- |
| DALYs (Disability-Adjusted Life Years) | Global | Male | Age-standardized | Osteoarthritis knee | High body-mass index | Rate | 1990 | 23.9708742 | 69.2086981 | -2.0998145 |
| DALYs (Disability-Adjusted Life Years) | Global | Female | Age-standardized | Osteoarthritis knee | High body-mass index | Rate | 1990 | 39.6312522 | 113.483067 | -3.4387253 |
| DALYs (Disability-Adjusted Life Years) | Global | Both | Age-standardized | Osteoarthritis knee | High body-mass index | Rate | 1990 | 32.3473099 | 92.9508559 | -2.814638 |
| DALYs (Disability-Adjusted Life Years) | Global | Male | Age-standardized | Osteoarthritis knee | High body-mass index | Rate | 2021 | 34.9535571 | 98.299802 | -3.3125267 |
| DALYs (Disability-Adjusted Life Years) | Global | Female | Age-standardized | Osteoarthritis knee | High body-mass index | Rate | 2021 | 55.9593903 | 155.734909 | -5.3252273 |
| DALYs (Disability-Adjusted Life Years) | Global | Both | Age-standardized | Osteoarthritis knee | High body-mass index | Rate | 2021 | 45.9275427 | 128.3321 | -4.3603188 |
| YLDs (Years Lived with Disability) | Global | Male | Age-standardized | Osteoarthritis knee | High body-mass index | Rate | 1990 | 23.9708742 | 69.2086981 | -2.0998145 |
| YLDs (Years Lived with Disability) | Global | Female | Age-standardized | Osteoarthritis knee | High body-mass index | Rate | 1990 | 39.6312522 | 113.483067 | -3.4387253 |
| YLDs (Years Lived with Disability) | Global | Both | Age-standardized | Osteoarthritis knee | High body-mass index | Rate | 1990 | 32.3473099 | 92.9508559 | -2.814638 |
| YLDs (Years Lived with Disability) | Global | Male | Age-standardized | Osteoarthritis knee | High body-mass index | Rate | 2021 | 34.9535571 | 98.299802 | -3.3125267 |
| YLDs (Years Lived with Disability) | Global | Female | Age-standardized | Osteoarthritis knee | High body-mass index | Rate | 2021 | 55.9593903 | 155.734909 | -5.3252273 |
| YLDs (Years Lived with Disability) | Global | Both | Age-standardized | Osteoarthritis knee | High body-mass index | Rate | 2021 | 45.9275427 | 128.3321 | -4.3603188 |

Appendix 3：By 2050, the global DALYs and YLDs associated with knee osteoarthritis attributable to high BMI

| year | age | val | lower | upper | measure | sex | metric |
| --- | --- | --- | --- | --- | --- | --- | --- |
| 2022 | All ages | 1511195.745 | 1503644.312 | 1518747.178 | DALYs | Male | Number |
| 2022 | All ages | 2634911.235 | 2625043.852 | 2644778.619 | DALYs | Female | Number |
| 2022 | All ages | 4136362.956 | 4118687.085 | 4154038.826 | DALYs | Both | Number |
| 2022 | All ages | 1511195.745 | 1503644.312 | 1518747.178 | YLDs | Male | Number |
| 2022 | All ages | 2634911.235 | 2625043.852 | 2644778.619 | YLDs | Female | Number |
| 2022 | All ages | 4136362.956 | 4118687.085 | 4154038.826 | YLDs | Both | Number |
| 2050 | All ages | 2845449.367 | 2388332.757 | 3302565.977 | DALYs | Male | Number |
| 2050 | All ages | 4745401.64 | 3832735.359 | 5658067.922 | DALYs | Female | Number |
| 2050 | All ages | 7406988.19 | 5772089.605 | 9041886.774 | DALYs | Both | Number |
| 2050 | All ages | 2845449.367 | 2388332.757 | 3302565.977 | YLDs | Male | Number |
| 2050 | All ages | 4745401.64 | 3832735.359 | 5658067.922 | YLDs | Female | Number |
| 2050 | All ages | 7406988.19 | 5772089.605 | 9041886.774 | YLDs | Both | Number |

Appendix 4: By 2050, the global age-standardized disability-adjusted life years (ASDR) and age-standardized years lived with disability (YLD) rates for knee osteoarthritis due to high BMI

| year | age | val | lower | upper | measure | sex | metric |
| --- | --- | --- | --- | --- | --- | --- | --- |
| 2022 | Age-standardized | 35.3541481 | 35.2037622 | 35.5045339 | DALYs | Male | Rate |
| 2022 | Age-standardized | 56.3806857 | 56.178165 | 56.5832064 | DALYs | Female | Rate |
| 2022 | Age-standardized | 46.3903114 | 46.2169015 | 46.5637213 | DALYs | Both | Rate |
| 2022 | Age-standardized | 35.3541481 | 35.2037622 | 35.5045339 | YLDs | Male | Rate |
| 2022 | Age-standardized | 56.3806857 | 56.178165 | 56.5832064 | YLDs | Female | Rate |
| 2022 | Age-standardized | 46.3903114 | 46.2169015 | 46.5637213 | YLDs | Both | Rate |
| 2050 | Age-standardized | 46.5706943 | 40.9659091 | 52.1754795 | DALYs | Male | Rate |
| 2050 | Age-standardized | 68.1769566 | 49.4451616 | 86.9087515 | DALYs | Female | Rate |
| 2050 | Age-standardized | 59.3478355 | 50.5940216 | 68.1016493 | DALYs | Both | Rate |
| 2050 | Age-standardized | 46.5706943 | 40.9659091 | 52.1754795 | YLDs | Male | Rate |
| 2050 | Age-standardized | 68.1769566 | 49.4451616 | 86.9087515 | YLDs | Female | Rate |
| 2050 | Age-standardized | 59.3478355 | 50.5940216 | 68.1016493 | YLDs | Both | Rate |

Appendix 5: In 1990, the DALYs and YLDs associated with knee osteoarthritis due to high BMI across 204 countries

| measure | location | sex | age | cause | rei | metric | year | val | upper | lower |
| --- | --- | --- | --- | --- | --- | --- | --- | --- | --- | --- |
| DALYs (Disability-Adjusted Life Years) | China | Both | All ages | Osteoarthritis knee | High body-mass index | Number | 1990 | 236876.755 | 702283.279 | -19470.525 |
| YLDs (Years Lived with Disability) | China | Both | All ages | Osteoarthritis knee | High body-mass index | Number | 1990 | 236876.755 | 702283.279 | -19470.525 |
| DALYs (Disability-Adjusted Life Years) | United States of America | Both | All ages | Osteoarthritis knee | High body-mass index | Number | 1990 | 180832.776 | 489880.801 | -17765.996 |
| YLDs (Years Lived with Disability) | United States of America | Both | All ages | Osteoarthritis knee | High body-mass index | Number | 1990 | 180832.776 | 489880.801 | -17765.996 |
| DALYs (Disability-Adjusted Life Years) | Russian Federation | Both | All ages | Osteoarthritis knee | High body-mass index | Number | 1990 | 67931.6872 | 187587.661 | -6439.7781 |
| YLDs (Years Lived with Disability) | Russian Federation | Both | All ages | Osteoarthritis knee | High body-mass index | Number | 1990 | 67931.6872 | 187587.661 | -6439.7781 |
| DALYs (Disability-Adjusted Life Years) | Japan | Both | All ages | Osteoarthritis knee | High body-mass index | Number | 1990 | 64233.5732 | 191669.359 | -5129.3977 |
| YLDs (Years Lived with Disability) | Japan | Both | All ages | Osteoarthritis knee | High body-mass index | Number | 1990 | 64233.5732 | 191669.359 | -5129.3977 |
| DALYs (Disability-Adjusted Life Years) | India | Both | All ages | Osteoarthritis knee | High body-mass index | Number | 1990 | 62623.1629 | 184590.063 | -4478.7098 |
| YLDs (Years Lived with Disability) | India | Both | All ages | Osteoarthritis knee | High body-mass index | Number | 1990 | 62623.1629 | 184590.063 | -4478.7098 |
| DALYs (Disability-Adjusted Life Years) | Germany | Both | All ages | Osteoarthritis knee | High body-mass index | Number | 1990 | 57897.7826 | 161585.137 | -5346.7774 |
| YLDs (Years Lived with Disability) | Germany | Both | All ages | Osteoarthritis knee | High body-mass index | Number | 1990 | 57897.7826 | 161585.137 | -5346.7774 |
| DALYs (Disability-Adjusted Life Years) | United Kingdom | Both | All ages | Osteoarthritis knee | High body-mass index | Number | 1990 | 42869.0286 | 117465.654 | -4091.3807 |
| YLDs (Years Lived with Disability) | United Kingdom | Both | All ages | Osteoarthritis knee | High body-mass index | Number | 1990 | 42869.0286 | 117465.654 | -4091.3807 |
| DALYs (Disability-Adjusted Life Years) | Brazil | Both | All ages | Osteoarthritis knee | High body-mass index | Number | 1990 | 39529.7732 | 109688.389 | -3538.4416 |
| YLDs (Years Lived with Disability) | Brazil | Both | All ages | Osteoarthritis knee | High body-mass index | Number | 1990 | 39529.7732 | 109688.389 | -3538.4416 |
| DALYs (Disability-Adjusted Life Years) | Italy | Both | All ages | Osteoarthritis knee | High body-mass index | Number | 1990 | 34588.7257 | 100098.46 | -2930.0187 |
| YLDs (Years Lived with Disability) | Italy | Both | All ages | Osteoarthritis knee | High body-mass index | Number | 1990 | 34588.7257 | 100098.46 | -2930.0187 |
| DALYs (Disability-Adjusted Life Years) | France | Both | All ages | Osteoarthritis knee | High body-mass index | Number | 1990 | 29104.8404 | 85577.4178 | -2548.4865 |
| YLDs (Years Lived with Disability) | France | Both | All ages | Osteoarthritis knee | High body-mass index | Number | 1990 | 29104.8404 | 85577.4178 | -2548.4865 |
| DALYs (Disability-Adjusted Life Years) | Ukraine | Both | All ages | Osteoarthritis knee | High body-mass index | Number | 1990 | 27436.7204 | 74693.474 | -2521.8449 |
| YLDs (Years Lived with Disability) | Ukraine | Both | All ages | Osteoarthritis knee | High body-mass index | Number | 1990 | 27436.7204 | 74693.474 | -2521.8449 |
| DALYs (Disability-Adjusted Life Years) | Spain | Both | All ages | Osteoarthritis knee | High body-mass index | Number | 1990 | 24957.2678 | 70535.3252 | -2483.9122 |
| YLDs (Years Lived with Disability) | Spain | Both | All ages | Osteoarthritis knee | High body-mass index | Number | 1990 | 24957.2678 | 70535.3252 | -2483.9122 |
| DALYs (Disability-Adjusted Life Years) | Mexico | Both | All ages | Osteoarthritis knee | High body-mass index | Number | 1990 | 22380.7075 | 60243.8766 | -2225.6961 |
| YLDs (Years Lived with Disability) | Mexico | Both | All ages | Osteoarthritis knee | High body-mass index | Number | 1990 | 22380.7075 | 60243.8766 | -2225.6961 |
| DALYs (Disability-Adjusted Life Years) | Argentina | Both | All ages | Osteoarthritis knee | High body-mass index | Number | 1990 | 16354.4694 | 44192.0671 | -1574.9236 |
| YLDs (Years Lived with Disability) | Argentina | Both | All ages | Osteoarthritis knee | High body-mass index | Number | 1990 | 16354.4694 | 44192.0671 | -1574.9236 |
| DALYs (Disability-Adjusted Life Years) | T眉rkiye | Both | All ages | Osteoarthritis knee | High body-mass index | Number | 1990 | 16290.0528 | 43989.6629 | -1701.6964 |
| YLDs (Years Lived with Disability) | T眉rkiye | Both | All ages | Osteoarthritis knee | High body-mass index | Number | 1990 | 16290.0528 | 43989.6629 | -1701.6964 |
| DALYs (Disability-Adjusted Life Years) | Poland | Both | All ages | Osteoarthritis knee | High body-mass index | Number | 1990 | 15557.6671 | 42496.1919 | -1457.636 |
| YLDs (Years Lived with Disability) | Poland | Both | All ages | Osteoarthritis knee | High body-mass index | Number | 1990 | 15557.6671 | 42496.1919 | -1457.636 |
| DALYs (Disability-Adjusted Life Years) | Indonesia | Both | All ages | Osteoarthritis knee | High body-mass index | Number | 1990 | 13407.0632 | 39670.7834 | -1010.4708 |
| YLDs (Years Lived with Disability) | Indonesia | Both | All ages | Osteoarthritis knee | High body-mass index | Number | 1990 | 13407.0632 | 39670.7834 | -1010.4708 |
| DALYs (Disability-Adjusted Life Years) | Egypt | Both | All ages | Osteoarthritis knee | High body-mass index | Number | 1990 | 13114.5821 | 35495.4853 | -1334.5995 |
| YLDs (Years Lived with Disability) | Egypt | Both | All ages | Osteoarthritis knee | High body-mass index | Number | 1990 | 13114.5821 | 35495.4853 | -1334.5995 |
| DALYs (Disability-Adjusted Life Years) | Nigeria | Both | All ages | Osteoarthritis knee | High body-mass index | Number | 1990 | 11769.1976 | 34840.7017 | -953.11826 |
| YLDs (Years Lived with Disability) | Nigeria | Both | All ages | Osteoarthritis knee | High body-mass index | Number | 1990 | 11769.1976 | 34840.7017 | -953.11826 |
| DALYs (Disability-Adjusted Life Years) | Republic of Korea | Both | All ages | Osteoarthritis knee | High body-mass index | Number | 1990 | 11599.5268 | 33216.5586 | -1053.6613 |
| YLDs (Years Lived with Disability) | Republic of Korea | Both | All ages | Osteoarthritis knee | High body-mass index | Number | 1990 | 11599.5268 | 33216.5586 | -1053.6613 |
| DALYs (Disability-Adjusted Life Years) | Pakistan | Both | All ages | Osteoarthritis knee | High body-mass index | Number | 1990 | 9935.7785 | 28529.3195 | -819.58849 |
| YLDs (Years Lived with Disability) | Pakistan | Both | All ages | Osteoarthritis knee | High body-mass index | Number | 1990 | 9935.7785 | 28529.3195 | -819.58849 |
| DALYs (Disability-Adjusted Life Years) | Romania | Both | All ages | Osteoarthritis knee | High body-mass index | Number | 1990 | 9899.11891 | 27443.306 | -844.73912 |
| YLDs (Years Lived with Disability) | Romania | Both | All ages | Osteoarthritis knee | High body-mass index | Number | 1990 | 9899.11891 | 27443.306 | -844.73912 |
| DALYs (Disability-Adjusted Life Years) | Australia | Both | All ages | Osteoarthritis knee | High body-mass index | Number | 1990 | 9606.61225 | 26419.8668 | -920.63515 |
| YLDs (Years Lived with Disability) | Australia | Both | All ages | Osteoarthritis knee | High body-mass index | Number | 1990 | 9606.61225 | 26419.8668 | -920.63515 |
| DALYs (Disability-Adjusted Life Years) | Canada | Both | All ages | Osteoarthritis knee | High body-mass index | Number | 1990 | 9324.10716 | 25742.9176 | -900.25363 |
| YLDs (Years Lived with Disability) | Canada | Both | All ages | Osteoarthritis knee | High body-mass index | Number | 1990 | 9324.10716 | 25742.9176 | -900.25363 |
| DALYs (Disability-Adjusted Life Years) | South Africa | Both | All ages | Osteoarthritis knee | High body-mass index | Number | 1990 | 9032.0353 | 24637.5483 | -833.72068 |
| YLDs (Years Lived with Disability) | South Africa | Both | All ages | Osteoarthritis knee | High body-mass index | Number | 1990 | 9032.0353 | 24637.5483 | -833.72068 |
| DALYs (Disability-Adjusted Life Years) | Iran (Islamic Republic of) | Both | All ages | Osteoarthritis knee | High body-mass index | Number | 1990 | 8666.5113 | 24740.339 | -754.0164 |
| YLDs (Years Lived with Disability) | Iran (Islamic Republic of) | Both | All ages | Osteoarthritis knee | High body-mass index | Number | 1990 | 8666.5113 | 24740.339 | -754.0164 |
| DALYs (Disability-Adjusted Life Years) | Netherlands | Both | All ages | Osteoarthritis knee | High body-mass index | Number | 1990 | 8417.38857 | 23540.3289 | -807.74144 |
| YLDs (Years Lived with Disability) | Netherlands | Both | All ages | Osteoarthritis knee | High body-mass index | Number | 1990 | 8417.38857 | 23540.3289 | -807.74144 |
| DALYs (Disability-Adjusted Life Years) | Thailand | Both | All ages | Osteoarthritis knee | High body-mass index | Number | 1990 | 7675.81882 | 22006.7358 | -621.0783 |
| YLDs (Years Lived with Disability) | Thailand | Both | All ages | Osteoarthritis knee | High body-mass index | Number | 1990 | 7675.81882 | 22006.7358 | -621.0783 |
| DALYs (Disability-Adjusted Life Years) | Colombia | Both | All ages | Osteoarthritis knee | High body-mass index | Number | 1990 | 7583.03815 | 21144.5835 | -673.47459 |
| YLDs (Years Lived with Disability) | Colombia | Both | All ages | Osteoarthritis knee | High body-mass index | Number | 1990 | 7583.03815 | 21144.5835 | -673.47459 |
| DALYs (Disability-Adjusted Life Years) | Greece | Both | All ages | Osteoarthritis knee | High body-mass index | Number | 1990 | 6517.4775 | 18137.399 | -614.73256 |
| YLDs (Years Lived with Disability) | Greece | Both | All ages | Osteoarthritis knee | High body-mass index | Number | 1990 | 6517.4775 | 18137.399 | -614.73256 |
| DALYs (Disability-Adjusted Life Years) | Belgium | Both | All ages | Osteoarthritis knee | High body-mass index | Number | 1990 | 5850.53103 | 16740.8034 | -486.75592 |
| YLDs (Years Lived with Disability) | Belgium | Both | All ages | Osteoarthritis knee | High body-mass index | Number | 1990 | 5850.53103 | 16740.8034 | -486.75592 |
| DALYs (Disability-Adjusted Life Years) | Hungary | Both | All ages | Osteoarthritis knee | High body-mass index | Number | 1990 | 5835.10568 | 15978.9659 | -523.29413 |
| YLDs (Years Lived with Disability) | Hungary | Both | All ages | Osteoarthritis knee | High body-mass index | Number | 1990 | 5835.10568 | 15978.9659 | -523.29413 |
| DALYs (Disability-Adjusted Life Years) | Taiwan (Province of China) | Both | All ages | Osteoarthritis knee | High body-mass index | Number | 1990 | 5738.68524 | 16725.6006 | -493.98264 |
| YLDs (Years Lived with Disability) | Taiwan (Province of China) | Both | All ages | Osteoarthritis knee | High body-mass index | Number | 1990 | 5738.68524 | 16725.6006 | -493.98264 |
| DALYs (Disability-Adjusted Life Years) | Portugal | Both | All ages | Osteoarthritis knee | High body-mass index | Number | 1990 | 5647.90252 | 15927.197 | -519.57558 |
| YLDs (Years Lived with Disability) | Portugal | Both | All ages | Osteoarthritis knee | High body-mass index | Number | 1990 | 5647.90252 | 15927.197 | -519.57558 |
| DALYs (Disability-Adjusted Life Years) | Chile | Both | All ages | Osteoarthritis knee | High body-mass index | Number | 1990 | 5542.30019 | 14936.7062 | -578.97921 |
| YLDs (Years Lived with Disability) | Chile | Both | All ages | Osteoarthritis knee | High body-mass index | Number | 1990 | 5542.30019 | 14936.7062 | -578.97921 |
| DALYs (Disability-Adjusted Life Years) | Czechia | Both | All ages | Osteoarthritis knee | High body-mass index | Number | 1990 | 5383.1398 | 14607.1059 | -549.81277 |
| YLDs (Years Lived with Disability) | Czechia | Both | All ages | Osteoarthritis knee | High body-mass index | Number | 1990 | 5383.1398 | 14607.1059 | -549.81277 |
| DALYs (Disability-Adjusted Life Years) | Peru | Both | All ages | Osteoarthritis knee | High body-mass index | Number | 1990 | 5321.52659 | 14560.9155 | -506.91386 |
| YLDs (Years Lived with Disability) | Peru | Both | All ages | Osteoarthritis knee | High body-mass index | Number | 1990 | 5321.52659 | 14560.9155 | -506.91386 |
| DALYs (Disability-Adjusted Life Years) | Philippines | Both | All ages | Osteoarthritis knee | High body-mass index | Number | 1990 | 5083.94713 | 15057.8381 | -375.10015 |
| YLDs (Years Lived with Disability) | Philippines | Both | All ages | Osteoarthritis knee | High body-mass index | Number | 1990 | 5083.94713 | 15057.8381 | -375.10015 |
| DALYs (Disability-Adjusted Life Years) | Venezuela (Bolivarian Republic of) | Both | All ages | Osteoarthritis knee | High body-mass index | Number | 1990 | 5073.17308 | 14081.7196 | -504.07962 |
| YLDs (Years Lived with Disability) | Venezuela (Bolivarian Republic of) | Both | All ages | Osteoarthritis knee | High body-mass index | Number | 1990 | 5073.17308 | 14081.7196 | -504.07962 |
| DALYs (Disability-Adjusted Life Years) | Belarus | Both | All ages | Osteoarthritis knee | High body-mass index | Number | 1990 | 4918.08074 | 13601.4702 | -463.61934 |
| YLDs (Years Lived with Disability) | Belarus | Both | All ages | Osteoarthritis knee | High body-mass index | Number | 1990 | 4918.08074 | 13601.4702 | -463.61934 |
| DALYs (Disability-Adjusted Life Years) | Austria | Both | All ages | Osteoarthritis knee | High body-mass index | Number | 1990 | 4870.87228 | 13448.5172 | -425.11232 |
| YLDs (Years Lived with Disability) | Austria | Both | All ages | Osteoarthritis knee | High body-mass index | Number | 1990 | 4870.87228 | 13448.5172 | -425.11232 |
| DALYs (Disability-Adjusted Life Years) | Bangladesh | Both | All ages | Osteoarthritis knee | High body-mass index | Number | 1990 | 4798.22568 | 14104.4331 | -358.07932 |
| YLDs (Years Lived with Disability) | Bangladesh | Both | All ages | Osteoarthritis knee | High body-mass index | Number | 1990 | 4798.22568 | 14104.4331 | -358.07932 |
| DALYs (Disability-Adjusted Life Years) | Bulgaria | Both | All ages | Osteoarthritis knee | High body-mass index | Number | 1990 | 4775.49021 | 13204.1358 | -467.44102 |
| YLDs (Years Lived with Disability) | Bulgaria | Both | All ages | Osteoarthritis knee | High body-mass index | Number | 1990 | 4775.49021 | 13204.1358 | -467.44102 |
| DALYs (Disability-Adjusted Life Years) | Morocco | Both | All ages | Osteoarthritis knee | High body-mass index | Number | 1990 | 4748.94185 | 13029.8373 | -485.98126 |
| YLDs (Years Lived with Disability) | Morocco | Both | All ages | Osteoarthritis knee | High body-mass index | Number | 1990 | 4748.94185 | 13029.8373 | -485.98126 |
| DALYs (Disability-Adjusted Life Years) | Sweden | Both | All ages | Osteoarthritis knee | High body-mass index | Number | 1990 | 4618.55318 | 13125.4721 | -424.08422 |
| YLDs (Years Lived with Disability) | Sweden | Both | All ages | Osteoarthritis knee | High body-mass index | Number | 1990 | 4618.55318 | 13125.4721 | -424.08422 |
| DALYs (Disability-Adjusted Life Years) | Algeria | Both | All ages | Osteoarthritis knee | High body-mass index | Number | 1990 | 4301.43297 | 12367.0543 | -389.359 |
| YLDs (Years Lived with Disability) | Algeria | Both | All ages | Osteoarthritis knee | High body-mass index | Number | 1990 | 4301.43297 | 12367.0543 | -389.359 |
| DALYs (Disability-Adjusted Life Years) | Serbia | Both | All ages | Osteoarthritis knee | High body-mass index | Number | 1990 | 4218.57328 | 11469.4053 | -415.69931 |
| YLDs (Years Lived with Disability) | Serbia | Both | All ages | Osteoarthritis knee | High body-mass index | Number | 1990 | 4218.57328 | 11469.4053 | -415.69931 |
| DALYs (Disability-Adjusted Life Years) | Cuba | Both | All ages | Osteoarthritis knee | High body-mass index | Number | 1990 | 4045.04065 | 11645.0822 | -333.30654 |
| YLDs (Years Lived with Disability) | Cuba | Both | All ages | Osteoarthritis knee | High body-mass index | Number | 1990 | 4045.04065 | 11645.0822 | -333.30654 |
| DALYs (Disability-Adjusted Life Years) | Kazakhstan | Both | All ages | Osteoarthritis knee | High body-mass index | Number | 1990 | 3979.89395 | 10704.8771 | -360.36334 |
| YLDs (Years Lived with Disability) | Kazakhstan | Both | All ages | Osteoarthritis knee | High body-mass index | Number | 1990 | 3979.89395 | 10704.8771 | -360.36334 |
| DALYs (Disability-Adjusted Life Years) | Switzerland | Both | All ages | Osteoarthritis knee | High body-mass index | Number | 1990 | 3937.63754 | 11183.4995 | -331.12683 |
| YLDs (Years Lived with Disability) | Switzerland | Both | All ages | Osteoarthritis knee | High body-mass index | Number | 1990 | 3937.63754 | 11183.4995 | -331.12683 |
| DALYs (Disability-Adjusted Life Years) | Iraq | Both | All ages | Osteoarthritis knee | High body-mass index | Number | 1990 | 3755.90691 | 9900.8529 | -365.2296 |
| YLDs (Years Lived with Disability) | Iraq | Both | All ages | Osteoarthritis knee | High body-mass index | Number | 1990 | 3755.90691 | 9900.8529 | -365.2296 |
| DALYs (Disability-Adjusted Life Years) | Myanmar | Both | All ages | Osteoarthritis knee | High body-mass index | Number | 1990 | 3650.11874 | 10829.4307 | -271.58045 |
| YLDs (Years Lived with Disability) | Myanmar | Both | All ages | Osteoarthritis knee | High body-mass index | Number | 1990 | 3650.11874 | 10829.4307 | -271.58045 |
| DALYs (Disability-Adjusted Life Years) | Democratic People's Republic of Korea | Both | All ages | Osteoarthritis knee | High body-mass index | Number | 1990 | 3459.61646 | 9989.54885 | -317.50579 |
| YLDs (Years Lived with Disability) | Democratic People's Republic of Korea | Both | All ages | Osteoarthritis knee | High body-mass index | Number | 1990 | 3459.61646 | 9989.54885 | -317.50579 |
| DALYs (Disability-Adjusted Life Years) | Uzbekistan | Both | All ages | Osteoarthritis knee | High body-mass index | Number | 1990 | 3398.47092 | 9470.76653 | -337.09874 |
| YLDs (Years Lived with Disability) | Uzbekistan | Both | All ages | Osteoarthritis knee | High body-mass index | Number | 1990 | 3398.47092 | 9470.76653 | -337.09874 |
| DALYs (Disability-Adjusted Life Years) | Viet Nam | Both | All ages | Osteoarthritis knee | High body-mass index | Number | 1990 | 3099.07481 | 9691.69741 | -246.69565 |
| YLDs (Years Lived with Disability) | Viet Nam | Both | All ages | Osteoarthritis knee | High body-mass index | Number | 1990 | 3099.07481 | 9691.69741 | -246.69565 |
| DALYs (Disability-Adjusted Life Years) | Finland | Both | All ages | Osteoarthritis knee | High body-mass index | Number | 1990 | 3065.5816 | 8571.32677 | -286.88143 |
| YLDs (Years Lived with Disability) | Finland | Both | All ages | Osteoarthritis knee | High body-mass index | Number | 1990 | 3065.5816 | 8571.32677 | -286.88143 |
| DALYs (Disability-Adjusted Life Years) | Democratic Republic of the Congo | Both | All ages | Osteoarthritis knee | High body-mass index | Number | 1990 | 2960.38113 | 8663.3879 | -201.69803 |
| YLDs (Years Lived with Disability) | Democratic Republic of the Congo | Both | All ages | Osteoarthritis knee | High body-mass index | Number | 1990 | 2960.38113 | 8663.3879 | -201.69803 |
| DALYs (Disability-Adjusted Life Years) | Ethiopia | Both | All ages | Osteoarthritis knee | High body-mass index | Number | 1990 | 2958.81149 | 8804.26712 | -220.13032 |
| YLDs (Years Lived with Disability) | Ethiopia | Both | All ages | Osteoarthritis knee | High body-mass index | Number | 1990 | 2958.81149 | 8804.26712 | -220.13032 |
| DALYs (Disability-Adjusted Life Years) | Sudan | Both | All ages | Osteoarthritis knee | High body-mass index | Number | 1990 | 2947.22606 | 8351.47254 | -248.2944 |
| YLDs (Years Lived with Disability) | Sudan | Both | All ages | Osteoarthritis knee | High body-mass index | Number | 1990 | 2947.22606 | 8351.47254 | -248.2944 |
| DALYs (Disability-Adjusted Life Years) | Denmark | Both | All ages | Osteoarthritis knee | High body-mass index | Number | 1990 | 2890.6808 | 8323.69504 | -255.87603 |
| YLDs (Years Lived with Disability) | Denmark | Both | All ages | Osteoarthritis knee | High body-mass index | Number | 1990 | 2890.6808 | 8323.69504 | -255.87603 |
| DALYs (Disability-Adjusted Life Years) | Saudi Arabia | Both | All ages | Osteoarthritis knee | High body-mass index | Number | 1990 | 2690.40537 | 7214.12884 | -274.64639 |
| YLDs (Years Lived with Disability) | Saudi Arabia | Both | All ages | Osteoarthritis knee | High body-mass index | Number | 1990 | 2690.40537 | 7214.12884 | -274.64639 |
| DALYs (Disability-Adjusted Life Years) | Ecuador | Both | All ages | Osteoarthritis knee | High body-mass index | Number | 1990 | 2605.94648 | 7072.67188 | -244.18496 |
| YLDs (Years Lived with Disability) | Ecuador | Both | All ages | Osteoarthritis knee | High body-mass index | Number | 1990 | 2605.94648 | 7072.67188 | -244.18496 |
| DALYs (Disability-Adjusted Life Years) | Malaysia | Both | All ages | Osteoarthritis knee | High body-mass index | Number | 1990 | 2600.6155 | 7356.09633 | -217.25642 |
| YLDs (Years Lived with Disability) | Malaysia | Both | All ages | Osteoarthritis knee | High body-mass index | Number | 1990 | 2600.6155 | 7356.09633 | -217.25642 |
| DALYs (Disability-Adjusted Life Years) | United Republic of Tanzania | Both | All ages | Osteoarthritis knee | High body-mass index | Number | 1990 | 2570.31904 | 7755.61134 | -199.425 |
| YLDs (Years Lived with Disability) | United Republic of Tanzania | Both | All ages | Osteoarthritis knee | High body-mass index | Number | 1990 | 2570.31904 | 7755.61134 | -199.425 |
| DALYs (Disability-Adjusted Life Years) | Norway | Both | All ages | Osteoarthritis knee | High body-mass index | Number | 1990 | 2439.26251 | 6934.42788 | -206.99067 |
| YLDs (Years Lived with Disability) | Norway | Both | All ages | Osteoarthritis knee | High body-mass index | Number | 1990 | 2439.26251 | 6934.42788 | -206.99067 |
| DALYs (Disability-Adjusted Life Years) | Slovakia | Both | All ages | Osteoarthritis knee | High body-mass index | Number | 1990 | 2384.88788 | 6443.20456 | -242.13513 |
| YLDs (Years Lived with Disability) | Slovakia | Both | All ages | Osteoarthritis knee | High body-mass index | Number | 1990 | 2384.88788 | 6443.20456 | -242.13513 |
| DALYs (Disability-Adjusted Life Years) | Croatia | Both | All ages | Osteoarthritis knee | High body-mass index | Number | 1990 | 2304.97896 | 6314.73689 | -228.29931 |
| YLDs (Years Lived with Disability) | Croatia | Both | All ages | Osteoarthritis knee | High body-mass index | Number | 1990 | 2304.97896 | 6314.73689 | -228.29931 |
| DALYs (Disability-Adjusted Life Years) | Syrian Arab Republic | Both | All ages | Osteoarthritis knee | High body-mass index | Number | 1990 | 2259.94803 | 6088.17207 | -225.84483 |
| YLDs (Years Lived with Disability) | Syrian Arab Republic | Both | All ages | Osteoarthritis knee | High body-mass index | Number | 1990 | 2259.94803 | 6088.17207 | -225.84483 |
| DALYs (Disability-Adjusted Life Years) | Israel | Both | All ages | Osteoarthritis knee | High body-mass index | Number | 1990 | 2143.71167 | 5943.96556 | -206.54685 |
| YLDs (Years Lived with Disability) | Israel | Both | All ages | Osteoarthritis knee | High body-mass index | Number | 1990 | 2143.71167 | 5943.96556 | -206.54685 |
| DALYs (Disability-Adjusted Life Years) | Sri Lanka | Both | All ages | Osteoarthritis knee | High body-mass index | Number | 1990 | 2123.804 | 6167.87316 | -151.37896 |
| YLDs (Years Lived with Disability) | Sri Lanka | Both | All ages | Osteoarthritis knee | High body-mass index | Number | 1990 | 2123.804 | 6167.87316 | -151.37896 |
| DALYs (Disability-Adjusted Life Years) | Puerto Rico | Both | All ages | Osteoarthritis knee | High body-mass index | Number | 1990 | 2114.59276 | 5644.84938 | -208.94564 |
| YLDs (Years Lived with Disability) | Puerto Rico | Both | All ages | Osteoarthritis knee | High body-mass index | Number | 1990 | 2114.59276 | 5644.84938 | -208.94564 |
| DALYs (Disability-Adjusted Life Years) | Afghanistan | Both | All ages | Osteoarthritis knee | High body-mass index | Number | 1990 | 2022.16298 | 5730.68881 | -166.66397 |
| YLDs (Years Lived with Disability) | Afghanistan | Both | All ages | Osteoarthritis knee | High body-mass index | Number | 1990 | 2022.16298 | 5730.68881 | -166.66397 |
| DALYs (Disability-Adjusted Life Years) | Georgia | Both | All ages | Osteoarthritis knee | High body-mass index | Number | 1990 | 1992.47604 | 5503.94438 | -183.74042 |
| YLDs (Years Lived with Disability) | Georgia | Both | All ages | Osteoarthritis knee | High body-mass index | Number | 1990 | 1992.47604 | 5503.94438 | -183.74042 |
| DALYs (Disability-Adjusted Life Years) | New Zealand | Both | All ages | Osteoarthritis knee | High body-mass index | Number | 1990 | 1896.1012 | 5177.07393 | -181.36412 |
| YLDs (Years Lived with Disability) | New Zealand | Both | All ages | Osteoarthritis knee | High body-mass index | Number | 1990 | 1896.1012 | 5177.07393 | -181.36412 |
| DALYs (Disability-Adjusted Life Years) | Uruguay | Both | All ages | Osteoarthritis knee | High body-mass index | Number | 1990 | 1881.12422 | 5144.01495 | -178.08935 |
| YLDs (Years Lived with Disability) | Uruguay | Both | All ages | Osteoarthritis knee | High body-mass index | Number | 1990 | 1881.12422 | 5144.01495 | -178.08935 |
| DALYs (Disability-Adjusted Life Years) | Cameroon | Both | All ages | Osteoarthritis knee | High body-mass index | Number | 1990 | 1872.01526 | 5253.47128 | -162.62723 |
| YLDs (Years Lived with Disability) | Cameroon | Both | All ages | Osteoarthritis knee | High body-mass index | Number | 1990 | 1872.01526 | 5253.47128 | -162.62723 |
| DALYs (Disability-Adjusted Life Years) | Tunisia | Both | All ages | Osteoarthritis knee | High body-mass index | Number | 1990 | 1778.74794 | 5076.78401 | -170.83742 |
| YLDs (Years Lived with Disability) | Tunisia | Both | All ages | Osteoarthritis knee | High body-mass index | Number | 1990 | 1778.74794 | 5076.78401 | -170.83742 |
| DALYs (Disability-Adjusted Life Years) | Lithuania | Both | All ages | Osteoarthritis knee | High body-mass index | Number | 1990 | 1764.4739 | 4915.62066 | -168.03602 |
| YLDs (Years Lived with Disability) | Lithuania | Both | All ages | Osteoarthritis knee | High body-mass index | Number | 1990 | 1764.4739 | 4915.62066 | -168.03602 |
| DALYs (Disability-Adjusted Life Years) | Republic of Moldova | Both | All ages | Osteoarthritis knee | High body-mass index | Number | 1990 | 1734.93559 | 4777.97829 | -166.31655 |
| YLDs (Years Lived with Disability) | Republic of Moldova | Both | All ages | Osteoarthritis knee | High body-mass index | Number | 1990 | 1734.93559 | 4777.97829 | -166.31655 |
| DALYs (Disability-Adjusted Life Years) | Ireland | Both | All ages | Osteoarthritis knee | High body-mass index | Number | 1990 | 1712.45716 | 4817.06227 | -153.86067 |
| YLDs (Years Lived with Disability) | Ireland | Both | All ages | Osteoarthritis knee | High body-mass index | Number | 1990 | 1712.45716 | 4817.06227 | -153.86067 |
| DALYs (Disability-Adjusted Life Years) | Kenya | Both | All ages | Osteoarthritis knee | High body-mass index | Number | 1990 | 1676.15455 | 4834.06223 | -132.07401 |
| YLDs (Years Lived with Disability) | Kenya | Both | All ages | Osteoarthritis knee | High body-mass index | Number | 1990 | 1676.15455 | 4834.06223 | -132.07401 |
| DALYs (Disability-Adjusted Life Years) | Ghana | Both | All ages | Osteoarthritis knee | High body-mass index | Number | 1990 | 1600.14139 | 4719.92233 | -124.59126 |
| YLDs (Years Lived with Disability) | Ghana | Both | All ages | Osteoarthritis knee | High body-mass index | Number | 1990 | 1600.14139 | 4719.92233 | -124.59126 |
| DALYs (Disability-Adjusted Life Years) | Guatemala | Both | All ages | Osteoarthritis knee | High body-mass index | Number | 1990 | 1582.67723 | 4399.34982 | -140.48166 |
| YLDs (Years Lived with Disability) | Guatemala | Both | All ages | Osteoarthritis knee | High body-mass index | Number | 1990 | 1582.67723 | 4399.34982 | -140.48166 |
| DALYs (Disability-Adjusted Life Years) | Azerbaijan | Both | All ages | Osteoarthritis knee | High body-mass index | Number | 1990 | 1558.07159 | 4268.1397 | -142.68299 |
| YLDs (Years Lived with Disability) | Azerbaijan | Both | All ages | Osteoarthritis knee | High body-mass index | Number | 1990 | 1558.07159 | 4268.1397 | -142.68299 |
| DALYs (Disability-Adjusted Life Years) | Latvia | Both | All ages | Osteoarthritis knee | High body-mass index | Number | 1990 | 1497.82736 | 4017.725 | -157.37674 |
| YLDs (Years Lived with Disability) | Latvia | Both | All ages | Osteoarthritis knee | High body-mass index | Number | 1990 | 1497.82736 | 4017.725 | -157.37674 |
| DALYs (Disability-Adjusted Life Years) | El Salvador | Both | All ages | Osteoarthritis knee | High body-mass index | Number | 1990 | 1477.54894 | 4053.83106 | -152.78544 |
| YLDs (Years Lived with Disability) | El Salvador | Both | All ages | Osteoarthritis knee | High body-mass index | Number | 1990 | 1477.54894 | 4053.83106 | -152.78544 |
| DALYs (Disability-Adjusted Life Years) | Bosnia and Herzegovina | Both | All ages | Osteoarthritis knee | High body-mass index | Number | 1990 | 1435.8581 | 3969.98504 | -139.68368 |
| YLDs (Years Lived with Disability) | Bosnia and Herzegovina | Both | All ages | Osteoarthritis knee | High body-mass index | Number | 1990 | 1435.8581 | 3969.98504 | -139.68368 |
| DALYs (Disability-Adjusted Life Years) | Dominican Republic | Both | All ages | Osteoarthritis knee | High body-mass index | Number | 1990 | 1398.98919 | 3872.56844 | -119.45487 |
| YLDs (Years Lived with Disability) | Dominican Republic | Both | All ages | Osteoarthritis knee | High body-mass index | Number | 1990 | 1398.98919 | 3872.56844 | -119.45487 |
| DALYs (Disability-Adjusted Life Years) | Nepal | Both | All ages | Osteoarthritis knee | High body-mass index | Number | 1990 | 1331.05199 | 4037.36368 | -93.463846 |
| YLDs (Years Lived with Disability) | Nepal | Both | All ages | Osteoarthritis knee | High body-mass index | Number | 1990 | 1331.05199 | 4037.36368 | -93.463846 |
| DALYs (Disability-Adjusted Life Years) | Bolivia (Plurinational State of) | Both | All ages | Osteoarthritis knee | High body-mass index | Number | 1990 | 1304.65363 | 3643.09922 | -113.73145 |
| YLDs (Years Lived with Disability) | Bolivia (Plurinational State of) | Both | All ages | Osteoarthritis knee | High body-mass index | Number | 1990 | 1304.65363 | 3643.09922 | -113.73145 |
| DALYs (Disability-Adjusted Life Years) | C么te d'Ivoire | Both | All ages | Osteoarthritis knee | High body-mass index | Number | 1990 | 1297.51921 | 3818.09499 | -104.4484 |
| YLDs (Years Lived with Disability) | C么te d'Ivoire | Both | All ages | Osteoarthritis knee | High body-mass index | Number | 1990 | 1297.51921 | 3818.09499 | -104.4484 |
| DALYs (Disability-Adjusted Life Years) | Uganda | Both | All ages | Osteoarthritis knee | High body-mass index | Number | 1990 | 1155.93805 | 3544.11191 | -85.018315 |
| YLDs (Years Lived with Disability) | Uganda | Both | All ages | Osteoarthritis knee | High body-mass index | Number | 1990 | 1155.93805 | 3544.11191 | -85.018315 |
| DALYs (Disability-Adjusted Life Years) | Yemen | Both | All ages | Osteoarthritis knee | High body-mass index | Number | 1990 | 1122.09361 | 3245.99228 | -82.184808 |
| YLDs (Years Lived with Disability) | Yemen | Both | All ages | Osteoarthritis knee | High body-mass index | Number | 1990 | 1122.09361 | 3245.99228 | -82.184808 |
| DALYs (Disability-Adjusted Life Years) | Mozambique | Both | All ages | Osteoarthritis knee | High body-mass index | Number | 1990 | 1087.99939 | 3226.03604 | -78.262717 |
| YLDs (Years Lived with Disability) | Mozambique | Both | All ages | Osteoarthritis knee | High body-mass index | Number | 1990 | 1087.99939 | 3226.03604 | -78.262717 |
| DALYs (Disability-Adjusted Life Years) | Zimbabwe | Both | All ages | Osteoarthritis knee | High body-mass index | Number | 1990 | 1072.82923 | 3173.5377 | -81.673047 |
| YLDs (Years Lived with Disability) | Zimbabwe | Both | All ages | Osteoarthritis knee | High body-mass index | Number | 1990 | 1072.82923 | 3173.5377 | -81.673047 |
| DALYs (Disability-Adjusted Life Years) | Paraguay | Both | All ages | Osteoarthritis knee | High body-mass index | Number | 1990 | 1026.81834 | 2858.08977 | -100.34993 |
| YLDs (Years Lived with Disability) | Paraguay | Both | All ages | Osteoarthritis knee | High body-mass index | Number | 1990 | 1026.81834 | 2858.08977 | -100.34993 |
| DALYs (Disability-Adjusted Life Years) | Slovenia | Both | All ages | Osteoarthritis knee | High body-mass index | Number | 1990 | 943.035559 | 2562.75526 | -91.650136 |
| YLDs (Years Lived with Disability) | Slovenia | Both | All ages | Osteoarthritis knee | High body-mass index | Number | 1990 | 943.035559 | 2562.75526 | -91.650136 |
| DALYs (Disability-Adjusted Life Years) | Lebanon | Both | All ages | Osteoarthritis knee | High body-mass index | Number | 1990 | 941.86996 | 2573.66048 | -93.325523 |
| YLDs (Years Lived with Disability) | Lebanon | Both | All ages | Osteoarthritis knee | High body-mass index | Number | 1990 | 941.86996 | 2573.66048 | -93.325523 |
| DALYs (Disability-Adjusted Life Years) | Senegal | Both | All ages | Osteoarthritis knee | High body-mass index | Number | 1990 | 939.700212 | 2689.15076 | -77.680088 |
| YLDs (Years Lived with Disability) | Senegal | Both | All ages | Osteoarthritis knee | High body-mass index | Number | 1990 | 939.700212 | 2689.15076 | -77.680088 |
| DALYs (Disability-Adjusted Life Years) | Kyrgyzstan | Both | All ages | Osteoarthritis knee | High body-mass index | Number | 1990 | 891.908674 | 2454.90009 | -81.431584 |
| YLDs (Years Lived with Disability) | Kyrgyzstan | Both | All ages | Osteoarthritis knee | High body-mass index | Number | 1990 | 891.908674 | 2454.90009 | -81.431584 |
| DALYs (Disability-Adjusted Life Years) | Honduras | Both | All ages | Osteoarthritis knee | High body-mass index | Number | 1990 | 885.060301 | 2427.6009 | -79.19851 |
| YLDs (Years Lived with Disability) | Honduras | Both | All ages | Osteoarthritis knee | High body-mass index | Number | 1990 | 885.060301 | 2427.6009 | -79.19851 |
| DALYs (Disability-Adjusted Life Years) | Mali | Both | All ages | Osteoarthritis knee | High body-mass index | Number | 1990 | 877.364137 | 2576.84283 | -69.759523 |
| YLDs (Years Lived with Disability) | Mali | Both | All ages | Osteoarthritis knee | High body-mass index | Number | 1990 | 877.364137 | 2576.84283 | -69.759523 |
| DALYs (Disability-Adjusted Life Years) | Armenia | Both | All ages | Osteoarthritis knee | High body-mass index | Number | 1990 | 860.185002 | 2350.67827 | -85.022269 |
| YLDs (Years Lived with Disability) | Armenia | Both | All ages | Osteoarthritis knee | High body-mass index | Number | 1990 | 860.185002 | 2350.67827 | -85.022269 |
| DALYs (Disability-Adjusted Life Years) | Singapore | Both | All ages | Osteoarthritis knee | High body-mass index | Number | 1990 | 841.214222 | 2447.77726 | -66.279115 |
| YLDs (Years Lived with Disability) | Singapore | Both | All ages | Osteoarthritis knee | High body-mass index | Number | 1990 | 841.214222 | 2447.77726 | -66.279115 |
| DALYs (Disability-Adjusted Life Years) | Costa Rica | Both | All ages | Osteoarthritis knee | High body-mass index | Number | 1990 | 839.427575 | 2292.9458 | -85.92578 |
| YLDs (Years Lived with Disability) | Costa Rica | Both | All ages | Osteoarthritis knee | High body-mass index | Number | 1990 | 839.427575 | 2292.9458 | -85.92578 |
| DALYs (Disability-Adjusted Life Years) | Estonia | Both | All ages | Osteoarthritis knee | High body-mass index | Number | 1990 | 833.262786 | 2241.20099 | -75.228365 |
| YLDs (Years Lived with Disability) | Estonia | Both | All ages | Osteoarthritis knee | High body-mass index | Number | 1990 | 833.262786 | 2241.20099 | -75.228365 |
| DALYs (Disability-Adjusted Life Years) | Libya | Both | All ages | Osteoarthritis knee | High body-mass index | Number | 1990 | 827.528074 | 2265.59141 | -79.596502 |
| YLDs (Years Lived with Disability) | Libya | Both | All ages | Osteoarthritis knee | High body-mass index | Number | 1990 | 827.528074 | 2265.59141 | -79.596502 |
| DALYs (Disability-Adjusted Life Years) | Guinea | Both | All ages | Osteoarthritis knee | High body-mass index | Number | 1990 | 780.625389 | 2280.4767 | -62.15996 |
| YLDs (Years Lived with Disability) | Guinea | Both | All ages | Osteoarthritis knee | High body-mass index | Number | 1990 | 780.625389 | 2280.4767 | -62.15996 |
| DALYs (Disability-Adjusted Life Years) | Nicaragua | Both | All ages | Osteoarthritis knee | High body-mass index | Number | 1990 | 765.392509 | 2076.06057 | -75.937822 |
| YLDs (Years Lived with Disability) | Nicaragua | Both | All ages | Osteoarthritis knee | High body-mass index | Number | 1990 | 765.392509 | 2076.06057 | -75.937822 |
| DALYs (Disability-Adjusted Life Years) | Jamaica | Both | All ages | Osteoarthritis knee | High body-mass index | Number | 1990 | 751.934173 | 2141.54758 | -71.244667 |
| YLDs (Years Lived with Disability) | Jamaica | Both | All ages | Osteoarthritis knee | High body-mass index | Number | 1990 | 751.934173 | 2141.54758 | -71.244667 |
| DALYs (Disability-Adjusted Life Years) | Tajikistan | Both | All ages | Osteoarthritis knee | High body-mass index | Number | 1990 | 747.115827 | 2093.29369 | -69.356243 |
| YLDs (Years Lived with Disability) | Tajikistan | Both | All ages | Osteoarthritis knee | High body-mass index | Number | 1990 | 747.115827 | 2093.29369 | -69.356243 |
| DALYs (Disability-Adjusted Life Years) | Angola | Both | All ages | Osteoarthritis knee | High body-mass index | Number | 1990 | 736.999859 | 2165.23584 | -57.021613 |
| YLDs (Years Lived with Disability) | Angola | Both | All ages | Osteoarthritis knee | High body-mass index | Number | 1990 | 736.999859 | 2165.23584 | -57.021613 |
| DALYs (Disability-Adjusted Life Years) | Madagascar | Both | All ages | Osteoarthritis knee | High body-mass index | Number | 1990 | 723.79841 | 2263.83374 | -49.076289 |
| YLDs (Years Lived with Disability) | Madagascar | Both | All ages | Osteoarthritis knee | High body-mass index | Number | 1990 | 723.79841 | 2263.83374 | -49.076289 |
| DALYs (Disability-Adjusted Life Years) | Albania | Both | All ages | Osteoarthritis knee | High body-mass index | Number | 1990 | 718.660868 | 1906.63623 | -70.549387 |
| YLDs (Years Lived with Disability) | Albania | Both | All ages | Osteoarthritis knee | High body-mass index | Number | 1990 | 718.660868 | 1906.63623 | -70.549387 |
| DALYs (Disability-Adjusted Life Years) | Burkina Faso | Both | All ages | Osteoarthritis knee | High body-mass index | Number | 1990 | 710.109088 | 2100.67915 | -61.787568 |
| YLDs (Years Lived with Disability) | Burkina Faso | Both | All ages | Osteoarthritis knee | High body-mass index | Number | 1990 | 710.109088 | 2100.67915 | -61.787568 |
| DALYs (Disability-Adjusted Life Years) | North Macedonia | Both | All ages | Osteoarthritis knee | High body-mass index | Number | 1990 | 704.746798 | 1883.29969 | -69.830281 |
| YLDs (Years Lived with Disability) | North Macedonia | Both | All ages | Osteoarthritis knee | High body-mass index | Number | 1990 | 704.746798 | 1883.29969 | -69.830281 |
| DALYs (Disability-Adjusted Life Years) | Jordan | Both | All ages | Osteoarthritis knee | High body-mass index | Number | 1990 | 691.234682 | 1773.62961 | -72.785487 |
| YLDs (Years Lived with Disability) | Jordan | Both | All ages | Osteoarthritis knee | High body-mass index | Number | 1990 | 691.234682 | 1773.62961 | -72.785487 |
| DALYs (Disability-Adjusted Life Years) | Malawi | Both | All ages | Osteoarthritis knee | High body-mass index | Number | 1990 | 679.295192 | 2029.58591 | -56.94624 |
| YLDs (Years Lived with Disability) | Malawi | Both | All ages | Osteoarthritis knee | High body-mass index | Number | 1990 | 679.295192 | 2029.58591 | -56.94624 |
| DALYs (Disability-Adjusted Life Years) | Panama | Both | All ages | Osteoarthritis knee | High body-mass index | Number | 1990 | 671.431568 | 1863.61939 | -60.216442 |
| YLDs (Years Lived with Disability) | Panama | Both | All ages | Osteoarthritis knee | High body-mass index | Number | 1990 | 671.431568 | 1863.61939 | -60.216442 |
| DALYs (Disability-Adjusted Life Years) | Haiti | Both | All ages | Osteoarthritis knee | High body-mass index | Number | 1990 | 630.414786 | 1903.10542 | -47.396539 |
| YLDs (Years Lived with Disability) | Haiti | Both | All ages | Osteoarthritis knee | High body-mass index | Number | 1990 | 630.414786 | 1903.10542 | -47.396539 |
| DALYs (Disability-Adjusted Life Years) | Niger | Both | All ages | Osteoarthritis knee | High body-mass index | Number | 1990 | 622.720731 | 1794.85422 | -48.049179 |
| YLDs (Years Lived with Disability) | Niger | Both | All ages | Osteoarthritis knee | High body-mass index | Number | 1990 | 622.720731 | 1794.85422 | -48.049179 |
| DALYs (Disability-Adjusted Life Years) | Papua New Guinea | Both | All ages | Osteoarthritis knee | High body-mass index | Number | 1990 | 615.14312 | 1746.52884 | -53.416073 |
| YLDs (Years Lived with Disability) | Papua New Guinea | Both | All ages | Osteoarthritis knee | High body-mass index | Number | 1990 | 615.14312 | 1746.52884 | -53.416073 |
| DALYs (Disability-Adjusted Life Years) | Zambia | Both | All ages | Osteoarthritis knee | High body-mass index | Number | 1990 | 593.97321 | 1738.54474 | -44.93314 |
| YLDs (Years Lived with Disability) | Zambia | Both | All ages | Osteoarthritis knee | High body-mass index | Number | 1990 | 593.97321 | 1738.54474 | -44.93314 |
| DALYs (Disability-Adjusted Life Years) | Benin | Both | All ages | Osteoarthritis knee | High body-mass index | Number | 1990 | 590.479923 | 1690.31118 | -48.498864 |
| YLDs (Years Lived with Disability) | Benin | Both | All ages | Osteoarthritis knee | High body-mass index | Number | 1990 | 590.479923 | 1690.31118 | -48.498864 |
| DALYs (Disability-Adjusted Life Years) | Cambodia | Both | All ages | Osteoarthritis knee | High body-mass index | Number | 1990 | 584.709107 | 1733.46709 | -45.489946 |
| YLDs (Years Lived with Disability) | Cambodia | Both | All ages | Osteoarthritis knee | High body-mass index | Number | 1990 | 584.709107 | 1733.46709 | -45.489946 |
| DALYs (Disability-Adjusted Life Years) | Chad | Both | All ages | Osteoarthritis knee | High body-mass index | Number | 1990 | 580.248058 | 1789.26757 | -44.333167 |
| YLDs (Years Lived with Disability) | Chad | Both | All ages | Osteoarthritis knee | High body-mass index | Number | 1990 | 580.248058 | 1789.26757 | -44.333167 |
| DALYs (Disability-Adjusted Life Years) | Turkmenistan | Both | All ages | Osteoarthritis knee | High body-mass index | Number | 1990 | 571.923253 | 1554.15059 | -56.546852 |
| YLDs (Years Lived with Disability) | Turkmenistan | Both | All ages | Osteoarthritis knee | High body-mass index | Number | 1990 | 571.923253 | 1554.15059 | -56.546852 |
| DALYs (Disability-Adjusted Life Years) | Somalia | Both | All ages | Osteoarthritis knee | High body-mass index | Number | 1990 | 481.255711 | 1346.08933 | -35.80761 |
| YLDs (Years Lived with Disability) | Somalia | Both | All ages | Osteoarthritis knee | High body-mass index | Number | 1990 | 481.255711 | 1346.08933 | -35.80761 |
| DALYs (Disability-Adjusted Life Years) | Sierra Leone | Both | All ages | Osteoarthritis knee | High body-mass index | Number | 1990 | 475.570868 | 1416.98736 | -36.069081 |
| YLDs (Years Lived with Disability) | Sierra Leone | Both | All ages | Osteoarthritis knee | High body-mass index | Number | 1990 | 475.570868 | 1416.98736 | -36.069081 |
| DALYs (Disability-Adjusted Life Years) | Rwanda | Both | All ages | Osteoarthritis knee | High body-mass index | Number | 1990 | 470.643831 | 1378.50939 | -36.427607 |
| YLDs (Years Lived with Disability) | Rwanda | Both | All ages | Osteoarthritis knee | High body-mass index | Number | 1990 | 470.643831 | 1378.50939 | -36.427607 |
| DALYs (Disability-Adjusted Life Years) | Trinidad and Tobago | Both | All ages | Osteoarthritis knee | High body-mass index | Number | 1990 | 408.937276 | 1123.96876 | -36.68934 |
| YLDs (Years Lived with Disability) | Trinidad and Tobago | Both | All ages | Osteoarthritis knee | High body-mass index | Number | 1990 | 408.937276 | 1123.96876 | -36.68934 |
| DALYs (Disability-Adjusted Life Years) | Liberia | Both | All ages | Osteoarthritis knee | High body-mass index | Number | 1990 | 407.304682 | 1146.39211 | -34.372126 |
| YLDs (Years Lived with Disability) | Liberia | Both | All ages | Osteoarthritis knee | High body-mass index | Number | 1990 | 407.304682 | 1146.39211 | -34.372126 |
| DALYs (Disability-Adjusted Life Years) | Palestine | Both | All ages | Osteoarthritis knee | High body-mass index | Number | 1990 | 396.531192 | 1067.52781 | -41.883213 |
| YLDs (Years Lived with Disability) | Palestine | Both | All ages | Osteoarthritis knee | High body-mass index | Number | 1990 | 396.531192 | 1067.52781 | -41.883213 |
| DALYs (Disability-Adjusted Life Years) | Mauritania | Both | All ages | Osteoarthritis knee | High body-mass index | Number | 1990 | 380.723754 | 1054.68064 | -32.775813 |
| YLDs (Years Lived with Disability) | Mauritania | Both | All ages | Osteoarthritis knee | High body-mass index | Number | 1990 | 380.723754 | 1054.68064 | -32.775813 |
| DALYs (Disability-Adjusted Life Years) | Kuwait | Both | All ages | Osteoarthritis knee | High body-mass index | Number | 1990 | 356.507364 | 900.298488 | -39.551591 |
| YLDs (Years Lived with Disability) | Kuwait | Both | All ages | Osteoarthritis knee | High body-mass index | Number | 1990 | 356.507364 | 900.298488 | -39.551591 |
| DALYs (Disability-Adjusted Life Years) | Togo | Both | All ages | Osteoarthritis knee | High body-mass index | Number | 1990 | 330.501369 | 941.26847 | -25.376365 |
| YLDs (Years Lived with Disability) | Togo | Both | All ages | Osteoarthritis knee | High body-mass index | Number | 1990 | 330.501369 | 941.26847 | -25.376365 |
| DALYs (Disability-Adjusted Life Years) | South Sudan | Both | All ages | Osteoarthritis knee | High body-mass index | Number | 1990 | 324.22693 | 976.835256 | -25.728644 |
| YLDs (Years Lived with Disability) | South Sudan | Both | All ages | Osteoarthritis knee | High body-mass index | Number | 1990 | 324.22693 | 976.835256 | -25.728644 |
| DALYs (Disability-Adjusted Life Years) | Cyprus | Both | All ages | Osteoarthritis knee | High body-mass index | Number | 1990 | 299.906557 | 857.946553 | -24.712366 |
| YLDs (Years Lived with Disability) | Cyprus | Both | All ages | Osteoarthritis knee | High body-mass index | Number | 1990 | 299.906557 | 857.946553 | -24.712366 |
| DALYs (Disability-Adjusted Life Years) | Burundi | Both | All ages | Osteoarthritis knee | High body-mass index | Number | 1990 | 298.513504 | 934.50849 | -21.296103 |
| YLDs (Years Lived with Disability) | Burundi | Both | All ages | Osteoarthritis knee | High body-mass index | Number | 1990 | 298.513504 | 934.50849 | -21.296103 |
| DALYs (Disability-Adjusted Life Years) | Lesotho | Both | All ages | Osteoarthritis knee | High body-mass index | Number | 1990 | 293.490176 | 794.255574 | -27.094025 |
| YLDs (Years Lived with Disability) | Lesotho | Both | All ages | Osteoarthritis knee | High body-mass index | Number | 1990 | 293.490176 | 794.255574 | -27.094025 |
| DALYs (Disability-Adjusted Life Years) | Lao People's Democratic Republic | Both | All ages | Osteoarthritis knee | High body-mass index | Number | 1990 | 280.368265 | 847.880141 | -19.94279 |
| YLDs (Years Lived with Disability) | Lao People's Democratic Republic | Both | All ages | Osteoarthritis knee | High body-mass index | Number | 1990 | 280.368265 | 847.880141 | -19.94279 |
| DALYs (Disability-Adjusted Life Years) | Mongolia | Both | All ages | Osteoarthritis knee | High body-mass index | Number | 1990 | 277.358015 | 789.506793 | -25.438715 |
| YLDs (Years Lived with Disability) | Mongolia | Both | All ages | Osteoarthritis knee | High body-mass index | Number | 1990 | 277.358015 | 789.506793 | -25.438715 |
| DALYs (Disability-Adjusted Life Years) | Congo | Both | All ages | Osteoarthritis knee | High body-mass index | Number | 1990 | 272.434871 | 780.95049 | -22.522992 |
| YLDs (Years Lived with Disability) | Congo | Both | All ages | Osteoarthritis knee | High body-mass index | Number | 1990 | 272.434871 | 780.95049 | -22.522992 |
| DALYs (Disability-Adjusted Life Years) | United Arab Emirates | Both | All ages | Osteoarthritis knee | High body-mass index | Number | 1990 | 258.205823 | 693.161132 | -24.545504 |
| YLDs (Years Lived with Disability) | United Arab Emirates | Both | All ages | Osteoarthritis knee | High body-mass index | Number | 1990 | 258.205823 | 693.161132 | -24.545504 |
| DALYs (Disability-Adjusted Life Years) | Montenegro | Both | All ages | Osteoarthritis knee | High body-mass index | Number | 1990 | 254.559235 | 688.034946 | -24.781864 |
| YLDs (Years Lived with Disability) | Montenegro | Both | All ages | Osteoarthritis knee | High body-mass index | Number | 1990 | 254.559235 | 688.034946 | -24.781864 |
| DALYs (Disability-Adjusted Life Years) | Oman | Both | All ages | Osteoarthritis knee | High body-mass index | Number | 1990 | 253.740704 | 700.856303 | -23.293422 |
| YLDs (Years Lived with Disability) | Oman | Both | All ages | Osteoarthritis knee | High body-mass index | Number | 1990 | 253.740704 | 700.856303 | -23.293422 |
| DALYs (Disability-Adjusted Life Years) | Luxembourg | Both | All ages | Osteoarthritis knee | High body-mass index | Number | 1990 | 232.270007 | 655.882268 | -19.517353 |
| YLDs (Years Lived with Disability) | Luxembourg | Both | All ages | Osteoarthritis knee | High body-mass index | Number | 1990 | 232.270007 | 655.882268 | -19.517353 |
| DALYs (Disability-Adjusted Life Years) | Mauritius | Both | All ages | Osteoarthritis knee | High body-mass index | Number | 1990 | 209.167773 | 593.928875 | -16.647885 |
| YLDs (Years Lived with Disability) | Mauritius | Both | All ages | Osteoarthritis knee | High body-mass index | Number | 1990 | 209.167773 | 593.928875 | -16.647885 |
| DALYs (Disability-Adjusted Life Years) | Fiji | Both | All ages | Osteoarthritis knee | High body-mass index | Number | 1990 | 208.18411 | 559.373398 | -20.494066 |
| YLDs (Years Lived with Disability) | Fiji | Both | All ages | Osteoarthritis knee | High body-mass index | Number | 1990 | 208.18411 | 559.373398 | -20.494066 |
| DALYs (Disability-Adjusted Life Years) | Gabon | Both | All ages | Osteoarthritis knee | High body-mass index | Number | 1990 | 197.38703 | 547.927236 | -17.780327 |
| YLDs (Years Lived with Disability) | Gabon | Both | All ages | Osteoarthritis knee | High body-mass index | Number | 1990 | 197.38703 | 547.927236 | -17.780327 |
| DALYs (Disability-Adjusted Life Years) | Central African Republic | Both | All ages | Osteoarthritis knee | High body-mass index | Number | 1990 | 193.476058 | 599.715298 | -13.961004 |
| YLDs (Years Lived with Disability) | Central African Republic | Both | All ages | Osteoarthritis knee | High body-mass index | Number | 1990 | 193.476058 | 599.715298 | -13.961004 |
| DALYs (Disability-Adjusted Life Years) | Namibia | Both | All ages | Osteoarthritis knee | High body-mass index | Number | 1990 | 185.347032 | 559.746097 | -14.16497 |
| YLDs (Years Lived with Disability) | Namibia | Both | All ages | Osteoarthritis knee | High body-mass index | Number | 1990 | 185.347032 | 559.746097 | -14.16497 |
| DALYs (Disability-Adjusted Life Years) | Malta | Both | All ages | Osteoarthritis knee | High body-mass index | Number | 1990 | 166.105275 | 481.299929 | -14.125291 |
| YLDs (Years Lived with Disability) | Malta | Both | All ages | Osteoarthritis knee | High body-mass index | Number | 1990 | 166.105275 | 481.299929 | -14.125291 |
| DALYs (Disability-Adjusted Life Years) | Botswana | Both | All ages | Osteoarthritis knee | High body-mass index | Number | 1990 | 159.066551 | 432.635518 | -12.843289 |
| YLDs (Years Lived with Disability) | Botswana | Both | All ages | Osteoarthritis knee | High body-mass index | Number | 1990 | 159.066551 | 432.635518 | -12.843289 |
| DALYs (Disability-Adjusted Life Years) | Guyana | Both | All ages | Osteoarthritis knee | High body-mass index | Number | 1990 | 157.606906 | 444.960227 | -13.764129 |
| YLDs (Years Lived with Disability) | Guyana | Both | All ages | Osteoarthritis knee | High body-mass index | Number | 1990 | 157.606906 | 444.960227 | -13.764129 |
| DALYs (Disability-Adjusted Life Years) | Barbados | Both | All ages | Osteoarthritis knee | High body-mass index | Number | 1990 | 135.873489 | 378.627615 | -12.964989 |
| YLDs (Years Lived with Disability) | Barbados | Both | All ages | Osteoarthritis knee | High body-mass index | Number | 1990 | 135.873489 | 378.627615 | -12.964989 |
| DALYs (Disability-Adjusted Life Years) | Eritrea | Both | All ages | Osteoarthritis knee | High body-mass index | Number | 1990 | 131.020191 | 383.066014 | -10.801132 |
| YLDs (Years Lived with Disability) | Eritrea | Both | All ages | Osteoarthritis knee | High body-mass index | Number | 1990 | 131.020191 | 383.066014 | -10.801132 |
| DALYs (Disability-Adjusted Life Years) | Iceland | Both | All ages | Osteoarthritis knee | High body-mass index | Number | 1990 | 130.441763 | 358.614739 | -12.763619 |
| YLDs (Years Lived with Disability) | Iceland | Both | All ages | Osteoarthritis knee | High body-mass index | Number | 1990 | 130.441763 | 358.614739 | -12.763619 |
| DALYs (Disability-Adjusted Life Years) | Eswatini | Both | All ages | Osteoarthritis knee | High body-mass index | Number | 1990 | 127.234011 | 346.353207 | -12.964903 |
| YLDs (Years Lived with Disability) | Eswatini | Both | All ages | Osteoarthritis knee | High body-mass index | Number | 1990 | 127.234011 | 346.353207 | -12.964903 |
| DALYs (Disability-Adjusted Life Years) | Gambia | Both | All ages | Osteoarthritis knee | High body-mass index | Number | 1990 | 108.221774 | 307.547607 | -8.9281591 |
| YLDs (Years Lived with Disability) | Gambia | Both | All ages | Osteoarthritis knee | High body-mass index | Number | 1990 | 108.221774 | 307.547607 | -8.9281591 |
| DALYs (Disability-Adjusted Life Years) | Guinea-Bissau | Both | All ages | Osteoarthritis knee | High body-mass index | Number | 1990 | 100.09816 | 287.080777 | -8.0126198 |
| YLDs (Years Lived with Disability) | Guinea-Bissau | Both | All ages | Osteoarthritis knee | High body-mass index | Number | 1990 | 100.09816 | 287.080777 | -8.0126198 |
| DALYs (Disability-Adjusted Life Years) | Suriname | Both | All ages | Osteoarthritis knee | High body-mass index | Number | 1990 | 96.6868971 | 273.525103 | -8.6139121 |
| YLDs (Years Lived with Disability) | Suriname | Both | All ages | Osteoarthritis knee | High body-mass index | Number | 1990 | 96.6868971 | 273.525103 | -8.6139121 |
| DALYs (Disability-Adjusted Life Years) | Bahrain | Both | All ages | Osteoarthritis knee | High body-mass index | Number | 1990 | 92.2149651 | 249.072549 | -9.1786898 |
| YLDs (Years Lived with Disability) | Bahrain | Both | All ages | Osteoarthritis knee | High body-mass index | Number | 1990 | 92.2149651 | 249.072549 | -9.1786898 |
| DALYs (Disability-Adjusted Life Years) | Qatar | Both | All ages | Osteoarthritis knee | High body-mass index | Number | 1990 | 81.5318409 | 208.984662 | -9.3008441 |
| YLDs (Years Lived with Disability) | Qatar | Both | All ages | Osteoarthritis knee | High body-mass index | Number | 1990 | 81.5318409 | 208.984662 | -9.3008441 |
| DALYs (Disability-Adjusted Life Years) | Bahamas | Both | All ages | Osteoarthritis knee | High body-mass index | Number | 1990 | 81.2303858 | 224.524871 | -7.5268453 |
| YLDs (Years Lived with Disability) | Bahamas | Both | All ages | Osteoarthritis knee | High body-mass index | Number | 1990 | 81.2303858 | 224.524871 | -7.5268453 |
| DALYs (Disability-Adjusted Life Years) | Bhutan | Both | All ages | Osteoarthritis knee | High body-mass index | Number | 1990 | 79.54974 | 220.889031 | -7.1802618 |
| YLDs (Years Lived with Disability) | Bhutan | Both | All ages | Osteoarthritis knee | High body-mass index | Number | 1990 | 79.54974 | 220.889031 | -7.1802618 |
| DALYs (Disability-Adjusted Life Years) | Cabo Verde | Both | All ages | Osteoarthritis knee | High body-mass index | Number | 1990 | 61.4550328 | 183.499252 | -4.965125 |
| YLDs (Years Lived with Disability) | Cabo Verde | Both | All ages | Osteoarthritis knee | High body-mass index | Number | 1990 | 61.4550328 | 183.499252 | -4.965125 |
| DALYs (Disability-Adjusted Life Years) | Solomon Islands | Both | All ages | Osteoarthritis knee | High body-mass index | Number | 1990 | 58.1751631 | 165.370983 | -4.9084046 |
| YLDs (Years Lived with Disability) | Solomon Islands | Both | All ages | Osteoarthritis knee | High body-mass index | Number | 1990 | 58.1751631 | 165.370983 | -4.9084046 |
| DALYs (Disability-Adjusted Life Years) | Samoa | Both | All ages | Osteoarthritis knee | High body-mass index | Number | 1990 | 54.6346149 | 136.186895 | -6.5473214 |
| YLDs (Years Lived with Disability) | Samoa | Both | All ages | Osteoarthritis knee | High body-mass index | Number | 1990 | 54.6346149 | 136.186895 | -6.5473214 |
| DALYs (Disability-Adjusted Life Years) | United States Virgin Islands | Both | All ages | Osteoarthritis knee | High body-mass index | Number | 1990 | 54.4061851 | 146.531586 | -6.0575489 |
| YLDs (Years Lived with Disability) | United States Virgin Islands | Both | All ages | Osteoarthritis knee | High body-mass index | Number | 1990 | 54.4061851 | 146.531586 | -6.0575489 |
| DALYs (Disability-Adjusted Life Years) | Equatorial Guinea | Both | All ages | Osteoarthritis knee | High body-mass index | Number | 1990 | 50.6370796 | 147.851774 | -3.8774746 |
| YLDs (Years Lived with Disability) | Equatorial Guinea | Both | All ages | Osteoarthritis knee | High body-mass index | Number | 1990 | 50.6370796 | 147.851774 | -3.8774746 |
| DALYs (Disability-Adjusted Life Years) | Belize | Both | All ages | Osteoarthritis knee | High body-mass index | Number | 1990 | 48.0070554 | 133.0954 | -4.5981733 |
| YLDs (Years Lived with Disability) | Belize | Both | All ages | Osteoarthritis knee | High body-mass index | Number | 1990 | 48.0070554 | 133.0954 | -4.5981733 |
| DALYs (Disability-Adjusted Life Years) | Guam | Both | All ages | Osteoarthritis knee | High body-mass index | Number | 1990 | 46.7813023 | 125.037153 | -4.874342 |
| YLDs (Years Lived with Disability) | Guam | Both | All ages | Osteoarthritis knee | High body-mass index | Number | 1990 | 46.7813023 | 125.037153 | -4.874342 |
| DALYs (Disability-Adjusted Life Years) | Brunei Darussalam | Both | All ages | Osteoarthritis knee | High body-mass index | Number | 1990 | 44.2336523 | 130.64197 | -3.6171841 |
| YLDs (Years Lived with Disability) | Brunei Darussalam | Both | All ages | Osteoarthritis knee | High body-mass index | Number | 1990 | 44.2336523 | 130.64197 | -3.6171841 |
| DALYs (Disability-Adjusted Life Years) | Comoros | Both | All ages | Osteoarthritis knee | High body-mass index | Number | 1990 | 40.2555566 | 124.121955 | -3.0793888 |
| YLDs (Years Lived with Disability) | Comoros | Both | All ages | Osteoarthritis knee | High body-mass index | Number | 1990 | 40.2555566 | 124.121955 | -3.0793888 |
| DALYs (Disability-Adjusted Life Years) | Bermuda | Both | All ages | Osteoarthritis knee | High body-mass index | Number | 1990 | 36.4673179 | 98.7018808 | -3.4335447 |
| YLDs (Years Lived with Disability) | Bermuda | Both | All ages | Osteoarthritis knee | High body-mass index | Number | 1990 | 36.4673179 | 98.7018808 | -3.4335447 |
| DALYs (Disability-Adjusted Life Years) | Tonga | Both | All ages | Osteoarthritis knee | High body-mass index | Number | 1990 | 36.398163 | 94.1765674 | -3.9840351 |
| YLDs (Years Lived with Disability) | Tonga | Both | All ages | Osteoarthritis knee | High body-mass index | Number | 1990 | 36.398163 | 94.1765674 | -3.9840351 |
| DALYs (Disability-Adjusted Life Years) | Saint Lucia | Both | All ages | Osteoarthritis knee | High body-mass index | Number | 1990 | 35.4459784 | 97.6972107 | -3.2485129 |
| YLDs (Years Lived with Disability) | Saint Lucia | Both | All ages | Osteoarthritis knee | High body-mass index | Number | 1990 | 35.4459784 | 97.6972107 | -3.2485129 |
| DALYs (Disability-Adjusted Life Years) | Monaco | Both | All ages | Osteoarthritis knee | High body-mass index | Number | 1990 | 31.3121043 | 86.9789854 | -2.7993662 |
| YLDs (Years Lived with Disability) | Monaco | Both | All ages | Osteoarthritis knee | High body-mass index | Number | 1990 | 31.3121043 | 86.9789854 | -2.7993662 |
| DALYs (Disability-Adjusted Life Years) | Dominica | Both | All ages | Osteoarthritis knee | High body-mass index | Number | 1990 | 31.1624494 | 83.2560548 | -3.0598353 |
| YLDs (Years Lived with Disability) | Dominica | Both | All ages | Osteoarthritis knee | High body-mass index | Number | 1990 | 31.1624494 | 83.2560548 | -3.0598353 |
| DALYs (Disability-Adjusted Life Years) | Micronesia (Federated States of) | Both | All ages | Osteoarthritis knee | High body-mass index | Number | 1990 | 26.559889 | 68.6656045 | -2.8770279 |
| YLDs (Years Lived with Disability) | Micronesia (Federated States of) | Both | All ages | Osteoarthritis knee | High body-mass index | Number | 1990 | 26.559889 | 68.6656045 | -2.8770279 |
| DALYs (Disability-Adjusted Life Years) | Grenada | Both | All ages | Osteoarthritis knee | High body-mass index | Number | 1990 | 26.4931395 | 76.2052717 | -2.3521689 |
| YLDs (Years Lived with Disability) | Grenada | Both | All ages | Osteoarthritis knee | High body-mass index | Number | 1990 | 26.4931395 | 76.2052717 | -2.3521689 |
| DALYs (Disability-Adjusted Life Years) | Vanuatu | Both | All ages | Osteoarthritis knee | High body-mass index | Number | 1990 | 25.9629865 | 70.8763777 | -2.3325968 |
| YLDs (Years Lived with Disability) | Vanuatu | Both | All ages | Osteoarthritis knee | High body-mass index | Number | 1990 | 25.9629865 | 70.8763777 | -2.3325968 |
| DALYs (Disability-Adjusted Life Years) | Timor-Leste | Both | All ages | Osteoarthritis knee | High body-mass index | Number | 1990 | 25.4364942 | 78.0754367 | -2.1766472 |
| YLDs (Years Lived with Disability) | Timor-Leste | Both | All ages | Osteoarthritis knee | High body-mass index | Number | 1990 | 25.4364942 | 78.0754367 | -2.1766472 |
| DALYs (Disability-Adjusted Life Years) | Saint Vincent and the Grenadines | Both | All ages | Osteoarthritis knee | High body-mass index | Number | 1990 | 24.7168427 | 72.6479995 | -2.0229624 |
| YLDs (Years Lived with Disability) | Saint Vincent and the Grenadines | Both | All ages | Osteoarthritis knee | High body-mass index | Number | 1990 | 24.7168427 | 72.6479995 | -2.0229624 |
| DALYs (Disability-Adjusted Life Years) | Andorra | Both | All ages | Osteoarthritis knee | High body-mass index | Number | 1990 | 24.6487245 | 69.054202 | -2.3983718 |
| YLDs (Years Lived with Disability) | Andorra | Both | All ages | Osteoarthritis knee | High body-mass index | Number | 1990 | 24.6487245 | 69.054202 | -2.3983718 |
| DALYs (Disability-Adjusted Life Years) | Antigua and Barbuda | Both | All ages | Osteoarthritis knee | High body-mass index | Number | 1990 | 22.0722843 | 62.861729 | -2.100688 |
| YLDs (Years Lived with Disability) | Antigua and Barbuda | Both | All ages | Osteoarthritis knee | High body-mass index | Number | 1990 | 22.0722843 | 62.861729 | -2.100688 |
| DALYs (Disability-Adjusted Life Years) | Sao Tome and Principe | Both | All ages | Osteoarthritis knee | High body-mass index | Number | 1990 | 21.260941 | 60.4889176 | -1.8894759 |
| YLDs (Years Lived with Disability) | Sao Tome and Principe | Both | All ages | Osteoarthritis knee | High body-mass index | Number | 1990 | 21.260941 | 60.4889176 | -1.8894759 |
| DALYs (Disability-Adjusted Life Years) | Djibouti | Both | All ages | Osteoarthritis knee | High body-mass index | Number | 1990 | 20.5868135 | 61.3254671 | -1.6812715 |
| YLDs (Years Lived with Disability) | Djibouti | Both | All ages | Osteoarthritis knee | High body-mass index | Number | 1990 | 20.5868135 | 61.3254671 | -1.6812715 |
| DALYs (Disability-Adjusted Life Years) | Maldives | Both | All ages | Osteoarthritis knee | High body-mass index | Number | 1990 | 20.4005341 | 59.3990065 | -1.6395217 |
| YLDs (Years Lived with Disability) | Maldives | Both | All ages | Osteoarthritis knee | High body-mass index | Number | 1990 | 20.4005341 | 59.3990065 | -1.6395217 |
| DALYs (Disability-Adjusted Life Years) | Kiribati | Both | All ages | Osteoarthritis knee | High body-mass index | Number | 1990 | 19.9266548 | 53.7902704 | -2.0845447 |
| YLDs (Years Lived with Disability) | Kiribati | Both | All ages | Osteoarthritis knee | High body-mass index | Number | 1990 | 19.9266548 | 53.7902704 | -2.0845447 |
| DALYs (Disability-Adjusted Life Years) | Seychelles | Both | All ages | Osteoarthritis knee | High body-mass index | Number | 1990 | 18.5770427 | 51.9856983 | -1.7583592 |
| YLDs (Years Lived with Disability) | Seychelles | Both | All ages | Osteoarthritis knee | High body-mass index | Number | 1990 | 18.5770427 | 51.9856983 | -1.7583592 |
| DALYs (Disability-Adjusted Life Years) | American Samoa | Both | All ages | Osteoarthritis knee | High body-mass index | Number | 1990 | 16.6891762 | 42.3545876 | -2.0024544 |
| YLDs (Years Lived with Disability) | American Samoa | Both | All ages | Osteoarthritis knee | High body-mass index | Number | 1990 | 16.6891762 | 42.3545876 | -2.0024544 |
| DALYs (Disability-Adjusted Life Years) | Saint Kitts and Nevis | Both | All ages | Osteoarthritis knee | High body-mass index | Number | 1990 | 15.9315445 | 45.8011361 | -1.4203826 |
| YLDs (Years Lived with Disability) | Saint Kitts and Nevis | Both | All ages | Osteoarthritis knee | High body-mass index | Number | 1990 | 15.9315445 | 45.8011361 | -1.4203826 |
| DALYs (Disability-Adjusted Life Years) | San Marino | Both | All ages | Osteoarthritis knee | High body-mass index | Number | 1990 | 15.6316395 | 43.22956 | -1.3954723 |
| YLDs (Years Lived with Disability) | San Marino | Both | All ages | Osteoarthritis knee | High body-mass index | Number | 1990 | 15.6316395 | 43.22956 | -1.3954723 |
| DALYs (Disability-Adjusted Life Years) | Northern Mariana Islands | Both | All ages | Osteoarthritis knee | High body-mass index | Number | 1990 | 13.4680694 | 34.5781309 | -1.4988793 |
| YLDs (Years Lived with Disability) | Northern Mariana Islands | Both | All ages | Osteoarthritis knee | High body-mass index | Number | 1990 | 13.4680694 | 34.5781309 | -1.4988793 |
| DALYs (Disability-Adjusted Life Years) | Greenland | Both | All ages | Osteoarthritis knee | High body-mass index | Number | 1990 | 12.2372592 | 33.1516345 | -1.2057031 |
| YLDs (Years Lived with Disability) | Greenland | Both | All ages | Osteoarthritis knee | High body-mass index | Number | 1990 | 12.2372592 | 33.1516345 | -1.2057031 |
| DALYs (Disability-Adjusted Life Years) | Marshall Islands | Both | All ages | Osteoarthritis knee | High body-mass index | Number | 1990 | 8.61281642 | 22.669198 | -0.9509476 |
| YLDs (Years Lived with Disability) | Marshall Islands | Both | All ages | Osteoarthritis knee | High body-mass index | Number | 1990 | 8.61281642 | 22.669198 | -0.9509476 |
| DALYs (Disability-Adjusted Life Years) | Cook Islands | Both | All ages | Osteoarthritis knee | High body-mass index | Number | 1990 | 8.38848793 | 21.4415366 | -0.9492946 |
| YLDs (Years Lived with Disability) | Cook Islands | Both | All ages | Osteoarthritis knee | High body-mass index | Number | 1990 | 8.38848793 | 21.4415366 | -0.9492946 |
| DALYs (Disability-Adjusted Life Years) | Palau | Both | All ages | Osteoarthritis knee | High body-mass index | Number | 1990 | 5.92858818 | 15.5653719 | -0.6255544 |
| YLDs (Years Lived with Disability) | Palau | Both | All ages | Osteoarthritis knee | High body-mass index | Number | 1990 | 5.92858818 | 15.5653719 | -0.6255544 |
| DALYs (Disability-Adjusted Life Years) | Tuvalu | Both | All ages | Osteoarthritis knee | High body-mass index | Number | 1990 | 3.50012206 | 9.36098216 | -0.3466523 |
| YLDs (Years Lived with Disability) | Tuvalu | Both | All ages | Osteoarthritis knee | High body-mass index | Number | 1990 | 3.50012206 | 9.36098216 | -0.3466523 |
| DALYs (Disability-Adjusted Life Years) | Nauru | Both | All ages | Osteoarthritis knee | High body-mass index | Number | 1990 | 3.02496259 | 7.66728955 | -0.3567271 |
| YLDs (Years Lived with Disability) | Nauru | Both | All ages | Osteoarthritis knee | High body-mass index | Number | 1990 | 3.02496259 | 7.66728955 | -0.3567271 |
| DALYs (Disability-Adjusted Life Years) | Niue | Both | All ages | Osteoarthritis knee | High body-mass index | Number | 1990 | 1.15608167 | 3.02789017 | -0.117784 |
| YLDs (Years Lived with Disability) | Niue | Both | All ages | Osteoarthritis knee | High body-mass index | Number | 1990 | 1.15608167 | 3.02789017 | -0.117784 |
| DALYs (Disability-Adjusted Life Years) | Tokelau | Both | All ages | Osteoarthritis knee | High body-mass index | Number | 1990 | 0.66149644 | 1.74480938 | -0.0654396 |
| YLDs (Years Lived with Disability) | Tokelau | Both | All ages | Osteoarthritis knee | High body-mass index | Number | 1990 | 0.66149644 | 1.74480938 | -0.0654396 |

Appendix 6: In 1990, the age-standardized DALY rates and age-standardized YLD rates for knee osteoarthritis due to high BMI across 204 countries

| measure | location | sex | age | cause | rei | metric | year | val | upper | lower |
| --- | --- | --- | --- | --- | --- | --- | --- | --- | --- | --- |
| DALYs (Disability-Adjusted Life Years) | American Samoa | Both | Age-standardized | Osteoarthritis knee | High body-mass index | Rate | 1990 | 64.6678824 | 166.980647 | -7.5020637 |
| YLDs (Years Lived with Disability) | American Samoa | Both | Age-standardized | Osteoarthritis knee | High body-mass index | Rate | 1990 | 64.6678824 | 166.980647 | -7.5020637 |
| DALYs (Disability-Adjusted Life Years) | Cook Islands | Both | Age-standardized | Osteoarthritis knee | High body-mass index | Rate | 1990 | 61.7750792 | 159.510364 | -6.8943726 |
| YLDs (Years Lived with Disability) | Cook Islands | Both | Age-standardized | Osteoarthritis knee | High body-mass index | Rate | 1990 | 61.7750792 | 159.510364 | -6.8943726 |
| DALYs (Disability-Adjusted Life Years) | Tonga | Both | Age-standardized | Osteoarthritis knee | High body-mass index | Rate | 1990 | 60.7803285 | 158.75239 | -6.5414137 |
| YLDs (Years Lived with Disability) | Tonga | Both | Age-standardized | Osteoarthritis knee | High body-mass index | Rate | 1990 | 60.7803285 | 158.75239 | -6.5414137 |
| DALYs (Disability-Adjusted Life Years) | United States of America | Both | Age-standardized | Osteoarthritis knee | High body-mass index | Rate | 1990 | 60.0564398 | 161.54251 | -5.9549506 |
| YLDs (Years Lived with Disability) | United States of America | Both | Age-standardized | Osteoarthritis knee | High body-mass index | Rate | 1990 | 60.0564398 | 161.54251 | -5.9549506 |
| DALYs (Disability-Adjusted Life Years) | Samoa | Both | Age-standardized | Osteoarthritis knee | High body-mass index | Rate | 1990 | 59.7422718 | 150.484621 | -7.0492041 |
| YLDs (Years Lived with Disability) | Samoa | Both | Age-standardized | Osteoarthritis knee | High body-mass index | Rate | 1990 | 59.7422718 | 150.484621 | -7.0492041 |
| DALYs (Disability-Adjusted Life Years) | United States Virgin Islands | Both | Age-standardized | Osteoarthritis knee | High body-mass index | Rate | 1990 | 59.6099367 | 162.07438 | -6.52813 |
| YLDs (Years Lived with Disability) | United States Virgin Islands | Both | Age-standardized | Osteoarthritis knee | High body-mass index | Rate | 1990 | 59.6099367 | 162.07438 | -6.52813 |
| DALYs (Disability-Adjusted Life Years) | Puerto Rico | Both | Age-standardized | Osteoarthritis knee | High body-mass index | Rate | 1990 | 58.7280869 | 156.570308 | -5.8214879 |
| YLDs (Years Lived with Disability) | Puerto Rico | Both | Age-standardized | Osteoarthritis knee | High body-mass index | Rate | 1990 | 58.7280869 | 156.570308 | -5.8214879 |
| DALYs (Disability-Adjusted Life Years) | Northern Mariana Islands | Both | Age-standardized | Osteoarthritis knee | High body-mass index | Rate | 1990 | 57.6823731 | 153.710798 | -6.3523689 |
| YLDs (Years Lived with Disability) | Northern Mariana Islands | Both | Age-standardized | Osteoarthritis knee | High body-mass index | Rate | 1990 | 57.6823731 | 153.710798 | -6.3523689 |
| DALYs (Disability-Adjusted Life Years) | Palau | Both | Age-standardized | Osteoarthritis knee | High body-mass index | Rate | 1990 | 57.3947157 | 151.718162 | -5.9945176 |
| YLDs (Years Lived with Disability) | Palau | Both | Age-standardized | Osteoarthritis knee | High body-mass index | Rate | 1990 | 57.3947157 | 151.718162 | -5.9945176 |
| DALYs (Disability-Adjusted Life Years) | Bermuda | Both | Age-standardized | Osteoarthritis knee | High body-mass index | Rate | 1990 | 57.3427598 | 155.511258 | -5.3821847 |
| YLDs (Years Lived with Disability) | Bermuda | Both | Age-standardized | Osteoarthritis knee | High body-mass index | Rate | 1990 | 57.3427598 | 155.511258 | -5.3821847 |
| DALYs (Disability-Adjusted Life Years) | Nauru | Both | Age-standardized | Osteoarthritis knee | High body-mass index | Rate | 1990 | 56.0518044 | 146.024659 | -6.3750286 |
| YLDs (Years Lived with Disability) | Nauru | Both | Age-standardized | Osteoarthritis knee | High body-mass index | Rate | 1990 | 56.0518044 | 146.024659 | -6.3750286 |
| DALYs (Disability-Adjusted Life Years) | Niue | Both | Age-standardized | Osteoarthritis knee | High body-mass index | Rate | 1990 | 54.8390117 | 143.532582 | -5.6549256 |
| YLDs (Years Lived with Disability) | Niue | Both | Age-standardized | Osteoarthritis knee | High body-mass index | Rate | 1990 | 54.8390117 | 143.532582 | -5.6549256 |
| DALYs (Disability-Adjusted Life Years) | Dominica | Both | Age-standardized | Osteoarthritis knee | High body-mass index | Rate | 1990 | 54.3961822 | 144.591042 | -5.3737328 |
| YLDs (Years Lived with Disability) | Dominica | Both | Age-standardized | Osteoarthritis knee | High body-mass index | Rate | 1990 | 54.3961822 | 144.591042 | -5.3737328 |
| DALYs (Disability-Adjusted Life Years) | Chile | Both | Age-standardized | Osteoarthritis knee | High body-mass index | Rate | 1990 | 53.9082333 | 145.540448 | -5.59545 |
| YLDs (Years Lived with Disability) | Chile | Both | Age-standardized | Osteoarthritis knee | High body-mass index | Rate | 1990 | 53.9082333 | 145.540448 | -5.59545 |
| DALYs (Disability-Adjusted Life Years) | Guam | Both | Age-standardized | Osteoarthritis knee | High body-mass index | Rate | 1990 | 53.5051176 | 146.00463 | -5.4920315 |
| YLDs (Years Lived with Disability) | Guam | Both | Age-standardized | Osteoarthritis knee | High body-mass index | Rate | 1990 | 53.5051176 | 146.00463 | -5.4920315 |
| DALYs (Disability-Adjusted Life Years) | Micronesia (Federated States of) | Both | Age-standardized | Osteoarthritis knee | High body-mass index | Rate | 1990 | 52.5165978 | 136.705851 | -5.6278505 |
| YLDs (Years Lived with Disability) | Micronesia (Federated States of) | Both | Age-standardized | Osteoarthritis knee | High body-mass index | Rate | 1990 | 52.5165978 | 136.705851 | -5.6278505 |
| DALYs (Disability-Adjusted Life Years) | Belize | Both | Age-standardized | Osteoarthritis knee | High body-mass index | Rate | 1990 | 51.5270483 | 142.584983 | -4.925782 |
| YLDs (Years Lived with Disability) | Belize | Both | Age-standardized | Osteoarthritis knee | High body-mass index | Rate | 1990 | 51.5270483 | 142.584983 | -4.925782 |
| DALYs (Disability-Adjusted Life Years) | Barbados | Both | Age-standardized | Osteoarthritis knee | High body-mass index | Rate | 1990 | 51.456815 | 142.880269 | -5.1308265 |
| YLDs (Years Lived with Disability) | Barbados | Both | Age-standardized | Osteoarthritis knee | High body-mass index | Rate | 1990 | 51.456815 | 142.880269 | -5.1308265 |
| DALYs (Disability-Adjusted Life Years) | Bahamas | Both | Age-standardized | Osteoarthritis knee | High body-mass index | Rate | 1990 | 50.8081718 | 141.821894 | -4.6576135 |
| YLDs (Years Lived with Disability) | Bahamas | Both | Age-standardized | Osteoarthritis knee | High body-mass index | Rate | 1990 | 50.8081718 | 141.821894 | -4.6576135 |
| DALYs (Disability-Adjusted Life Years) | Fiji | Both | Age-standardized | Osteoarthritis knee | High body-mass index | Rate | 1990 | 50.6444653 | 139.074133 | -4.927528 |
| YLDs (Years Lived with Disability) | Fiji | Both | Age-standardized | Osteoarthritis knee | High body-mass index | Rate | 1990 | 50.6444653 | 139.074133 | -4.927528 |
| DALYs (Disability-Adjusted Life Years) | United Kingdom | Both | Age-standardized | Osteoarthritis knee | High body-mass index | Rate | 1990 | 50.365142 | 136.661573 | -4.8804151 |
| YLDs (Years Lived with Disability) | United Kingdom | Both | Age-standardized | Osteoarthritis knee | High body-mass index | Rate | 1990 | 50.365142 | 136.661573 | -4.8804151 |
| DALYs (Disability-Adjusted Life Years) | Venezuela (Bolivarian Republic of) | Both | Age-standardized | Osteoarthritis knee | High body-mass index | Rate | 1990 | 50.3336268 | 140.647477 | -4.9515302 |
| YLDs (Years Lived with Disability) | Venezuela (Bolivarian Republic of) | Both | Age-standardized | Osteoarthritis knee | High body-mass index | Rate | 1990 | 50.3336268 | 140.647477 | -4.9515302 |
| DALYs (Disability-Adjusted Life Years) | Tokelau | Both | Age-standardized | Osteoarthritis knee | High body-mass index | Rate | 1990 | 50.1996065 | 132.018437 | -5.0131613 |
| YLDs (Years Lived with Disability) | Tokelau | Both | Age-standardized | Osteoarthritis knee | High body-mass index | Rate | 1990 | 50.1996065 | 132.018437 | -5.0131613 |
| DALYs (Disability-Adjusted Life Years) | Argentina | Both | Age-standardized | Osteoarthritis knee | High body-mass index | Rate | 1990 | 50.1337132 | 135.582095 | -4.8362873 |
| YLDs (Years Lived with Disability) | Argentina | Both | Age-standardized | Osteoarthritis knee | High body-mass index | Rate | 1990 | 50.1337132 | 135.582095 | -4.8362873 |
| DALYs (Disability-Adjusted Life Years) | Monaco | Both | Age-standardized | Osteoarthritis knee | High body-mass index | Rate | 1990 | 50.1029147 | 137.826576 | -4.5309197 |
| YLDs (Years Lived with Disability) | Monaco | Both | Age-standardized | Osteoarthritis knee | High body-mass index | Rate | 1990 | 50.1029147 | 137.826576 | -4.5309197 |
| DALYs (Disability-Adjusted Life Years) | Mexico | Both | Age-standardized | Osteoarthritis knee | High body-mass index | Rate | 1990 | 49.9975875 | 135.388025 | -4.8979637 |
| YLDs (Years Lived with Disability) | Mexico | Both | Age-standardized | Osteoarthritis knee | High body-mass index | Rate | 1990 | 49.9975875 | 135.388025 | -4.8979637 |
| DALYs (Disability-Adjusted Life Years) | Australia | Both | Age-standardized | Osteoarthritis knee | High body-mass index | Rate | 1990 | 49.8559571 | 136.728326 | -4.7906492 |
| YLDs (Years Lived with Disability) | Australia | Both | Age-standardized | Osteoarthritis knee | High body-mass index | Rate | 1990 | 49.8559571 | 136.728326 | -4.7906492 |
| DALYs (Disability-Adjusted Life Years) | New Zealand | Both | Age-standardized | Osteoarthritis knee | High body-mass index | Rate | 1990 | 49.4804578 | 134.992704 | -4.7564535 |
| YLDs (Years Lived with Disability) | New Zealand | Both | Age-standardized | Osteoarthritis knee | High body-mass index | Rate | 1990 | 49.4804578 | 134.992704 | -4.7564535 |
| DALYs (Disability-Adjusted Life Years) | Marshall Islands | Both | Age-standardized | Osteoarthritis knee | High body-mass index | Rate | 1990 | 49.4470454 | 132.431923 | -5.3592672 |
| YLDs (Years Lived with Disability) | Marshall Islands | Both | Age-standardized | Osteoarthritis knee | High body-mass index | Rate | 1990 | 49.4470454 | 132.431923 | -5.3592672 |
| DALYs (Disability-Adjusted Life Years) | Uruguay | Both | Age-standardized | Osteoarthritis knee | High body-mass index | Rate | 1990 | 49.2908418 | 134.635537 | -4.6927839 |
| YLDs (Years Lived with Disability) | Uruguay | Both | Age-standardized | Osteoarthritis knee | High body-mass index | Rate | 1990 | 49.2908418 | 134.635537 | -4.6927839 |
| DALYs (Disability-Adjusted Life Years) | El Salvador | Both | Age-standardized | Osteoarthritis knee | High body-mass index | Rate | 1990 | 48.9913996 | 134.928646 | -5.0344985 |
| YLDs (Years Lived with Disability) | El Salvador | Both | Age-standardized | Osteoarthritis knee | High body-mass index | Rate | 1990 | 48.9913996 | 134.928646 | -5.0344985 |
| DALYs (Disability-Adjusted Life Years) | Kuwait | Both | Age-standardized | Osteoarthritis knee | High body-mass index | Rate | 1990 | 48.5108057 | 126.754939 | -5.2666384 |
| YLDs (Years Lived with Disability) | Kuwait | Both | Age-standardized | Osteoarthritis knee | High body-mass index | Rate | 1990 | 48.5108057 | 126.754939 | -5.2666384 |
| DALYs (Disability-Adjusted Life Years) | Kiribati | Both | Age-standardized | Osteoarthritis knee | High body-mass index | Rate | 1990 | 48.4885871 | 133.715312 | -4.9532601 |
| YLDs (Years Lived with Disability) | Kiribati | Both | Age-standardized | Osteoarthritis knee | High body-mass index | Rate | 1990 | 48.4885871 | 133.715312 | -4.9532601 |
| DALYs (Disability-Adjusted Life Years) | Qatar | Both | Age-standardized | Osteoarthritis knee | High body-mass index | Rate | 1990 | 48.1470222 | 129.106125 | -5.2044991 |
| YLDs (Years Lived with Disability) | Qatar | Both | Age-standardized | Osteoarthritis knee | High body-mass index | Rate | 1990 | 48.1470222 | 129.106125 | -5.2044991 |
| DALYs (Disability-Adjusted Life Years) | Tuvalu | Both | Age-standardized | Osteoarthritis knee | High body-mass index | Rate | 1990 | 48.0099596 | 129.204882 | -4.7079623 |
| YLDs (Years Lived with Disability) | Tuvalu | Both | Age-standardized | Osteoarthritis knee | High body-mass index | Rate | 1990 | 48.0099596 | 129.204882 | -4.7079623 |
| DALYs (Disability-Adjusted Life Years) | Trinidad and Tobago | Both | Age-standardized | Osteoarthritis knee | High body-mass index | Rate | 1990 | 47.8099787 | 131.968876 | -4.2735541 |
| YLDs (Years Lived with Disability) | Trinidad and Tobago | Both | Age-standardized | Osteoarthritis knee | High body-mass index | Rate | 1990 | 47.8099787 | 131.968876 | -4.2735541 |
| DALYs (Disability-Adjusted Life Years) | Nicaragua | Both | Age-standardized | Osteoarthritis knee | High body-mass index | Rate | 1990 | 47.7995366 | 130.346652 | -4.6762006 |
| YLDs (Years Lived with Disability) | Nicaragua | Both | Age-standardized | Osteoarthritis knee | High body-mass index | Rate | 1990 | 47.7995366 | 130.346652 | -4.6762006 |
| DALYs (Disability-Adjusted Life Years) | Iceland | Both | Age-standardized | Osteoarthritis knee | High body-mass index | Rate | 1990 | 47.7029931 | 130.440907 | -4.7055214 |
| YLDs (Years Lived with Disability) | Iceland | Both | Age-standardized | Osteoarthritis knee | High body-mass index | Rate | 1990 | 47.7029931 | 130.440907 | -4.7055214 |
| DALYs (Disability-Adjusted Life Years) | Germany | Both | Age-standardized | Osteoarthritis knee | High body-mass index | Rate | 1990 | 47.3773288 | 130.694116 | -4.3830816 |
| YLDs (Years Lived with Disability) | Germany | Both | Age-standardized | Osteoarthritis knee | High body-mass index | Rate | 1990 | 47.3773288 | 130.694116 | -4.3830816 |
| DALYs (Disability-Adjusted Life Years) | Costa Rica | Both | Age-standardized | Osteoarthritis knee | High body-mass index | Rate | 1990 | 47.1484688 | 129.132046 | -4.7891595 |
| YLDs (Years Lived with Disability) | Costa Rica | Both | Age-standardized | Osteoarthritis knee | High body-mass index | Rate | 1990 | 47.1484688 | 129.132046 | -4.7891595 |
| DALYs (Disability-Adjusted Life Years) | Ecuador | Both | Age-standardized | Osteoarthritis knee | High body-mass index | Rate | 1990 | 47.1384354 | 129.286484 | -4.366759 |
| YLDs (Years Lived with Disability) | Ecuador | Both | Age-standardized | Osteoarthritis knee | High body-mass index | Rate | 1990 | 47.1384354 | 129.286484 | -4.366759 |
| DALYs (Disability-Adjusted Life Years) | Jordan | Both | Age-standardized | Osteoarthritis knee | High body-mass index | Rate | 1990 | 46.988375 | 124.052202 | -4.7820746 |
| YLDs (Years Lived with Disability) | Jordan | Both | Age-standardized | Osteoarthritis knee | High body-mass index | Rate | 1990 | 46.988375 | 124.052202 | -4.7820746 |
| DALYs (Disability-Adjusted Life Years) | Spain | Both | Age-standardized | Osteoarthritis knee | High body-mass index | Rate | 1990 | 46.7704563 | 131.105419 | -4.6653411 |
| YLDs (Years Lived with Disability) | Spain | Both | Age-standardized | Osteoarthritis knee | High body-mass index | Rate | 1990 | 46.7704563 | 131.105419 | -4.6653411 |
| DALYs (Disability-Adjusted Life Years) | San Marino | Both | Age-standardized | Osteoarthritis knee | High body-mass index | Rate | 1990 | 46.6407593 | 128.295415 | -4.199913 |
| YLDs (Years Lived with Disability) | San Marino | Both | Age-standardized | Osteoarthritis knee | High body-mass index | Rate | 1990 | 46.6407593 | 128.295415 | -4.199913 |
| DALYs (Disability-Adjusted Life Years) | Iraq | Both | Age-standardized | Osteoarthritis knee | High body-mass index | Rate | 1990 | 46.0560199 | 122.964736 | -4.4225225 |
| YLDs (Years Lived with Disability) | Iraq | Both | Age-standardized | Osteoarthritis knee | High body-mass index | Rate | 1990 | 46.0560199 | 122.964736 | -4.4225225 |
| DALYs (Disability-Adjusted Life Years) | Saint Kitts and Nevis | Both | Age-standardized | Osteoarthritis knee | High body-mass index | Rate | 1990 | 45.976144 | 129.166837 | -4.1752832 |
| YLDs (Years Lived with Disability) | Saint Kitts and Nevis | Both | Age-standardized | Osteoarthritis knee | High body-mass index | Rate | 1990 | 45.976144 | 129.166837 | -4.1752832 |
| DALYs (Disability-Adjusted Life Years) | Paraguay | Both | Age-standardized | Osteoarthritis knee | High body-mass index | Rate | 1990 | 45.1678916 | 125.959641 | -4.3800638 |
| YLDs (Years Lived with Disability) | Paraguay | Both | Age-standardized | Osteoarthritis knee | High body-mass index | Rate | 1990 | 45.1678916 | 125.959641 | -4.3800638 |
| DALYs (Disability-Adjusted Life Years) | Israel | Both | Age-standardized | Osteoarthritis knee | High body-mass index | Rate | 1990 | 45.1372405 | 124.651484 | -4.3978194 |
| YLDs (Years Lived with Disability) | Israel | Both | Age-standardized | Osteoarthritis knee | High body-mass index | Rate | 1990 | 45.1372405 | 124.651484 | -4.3978194 |
| DALYs (Disability-Adjusted Life Years) | Panama | Both | Age-standardized | Osteoarthritis knee | High body-mass index | Rate | 1990 | 44.906767 | 124.938189 | -4.0427775 |
| YLDs (Years Lived with Disability) | Panama | Both | Age-standardized | Osteoarthritis knee | High body-mass index | Rate | 1990 | 44.906767 | 124.938189 | -4.0427775 |
| DALYs (Disability-Adjusted Life Years) | T眉rkiye | Both | Age-standardized | Osteoarthritis knee | High body-mass index | Rate | 1990 | 44.5127079 | 121.158175 | -4.564504 |
| YLDs (Years Lived with Disability) | T眉rkiye | Both | Age-standardized | Osteoarthritis knee | High body-mass index | Rate | 1990 | 44.5127079 | 121.158175 | -4.564504 |
| DALYs (Disability-Adjusted Life Years) | Palestine | Both | Age-standardized | Osteoarthritis knee | High body-mass index | Rate | 1990 | 44.5092141 | 120.288212 | -4.6261706 |
| YLDs (Years Lived with Disability) | Palestine | Both | Age-standardized | Osteoarthritis knee | High body-mass index | Rate | 1990 | 44.5092141 | 120.288212 | -4.6261706 |
| DALYs (Disability-Adjusted Life Years) | Jamaica | Both | Age-standardized | Osteoarthritis knee | High body-mass index | Rate | 1990 | 44.2281696 | 125.687285 | -4.2233285 |
| YLDs (Years Lived with Disability) | Jamaica | Both | Age-standardized | Osteoarthritis knee | High body-mass index | Rate | 1990 | 44.2281696 | 125.687285 | -4.2233285 |
| DALYs (Disability-Adjusted Life Years) | Antigua and Barbuda | Both | Age-standardized | Osteoarthritis knee | High body-mass index | Rate | 1990 | 44.172767 | 124.351954 | -4.313565 |
| YLDs (Years Lived with Disability) | Antigua and Barbuda | Both | Age-standardized | Osteoarthritis knee | High body-mass index | Rate | 1990 | 44.172767 | 124.351954 | -4.313565 |
| DALYs (Disability-Adjusted Life Years) | Egypt | Both | Age-standardized | Osteoarthritis knee | High body-mass index | Rate | 1990 | 44.1264302 | 121.366655 | -4.3374642 |
| YLDs (Years Lived with Disability) | Egypt | Both | Age-standardized | Osteoarthritis knee | High body-mass index | Rate | 1990 | 44.1264302 | 121.366655 | -4.3374642 |
| DALYs (Disability-Adjusted Life Years) | Finland | Both | Age-standardized | Osteoarthritis knee | High body-mass index | Rate | 1990 | 44.0695944 | 123.75072 | -4.1661441 |
| YLDs (Years Lived with Disability) | Finland | Both | Age-standardized | Osteoarthritis knee | High body-mass index | Rate | 1990 | 44.0695944 | 123.75072 | -4.1661441 |
| DALYs (Disability-Adjusted Life Years) | Netherlands | Both | Age-standardized | Osteoarthritis knee | High body-mass index | Rate | 1990 | 43.5203226 | 120.607768 | -4.2037788 |
| YLDs (Years Lived with Disability) | Netherlands | Both | Age-standardized | Osteoarthritis knee | High body-mass index | Rate | 1990 | 43.5203226 | 120.607768 | -4.2037788 |
| DALYs (Disability-Adjusted Life Years) | Greece | Both | Age-standardized | Osteoarthritis knee | High body-mass index | Rate | 1990 | 43.5021256 | 120.396139 | -4.1195161 |
| YLDs (Years Lived with Disability) | Greece | Both | Age-standardized | Osteoarthritis knee | High body-mass index | Rate | 1990 | 43.5021256 | 120.396139 | -4.1195161 |
| DALYs (Disability-Adjusted Life Years) | Bahrain | Both | Age-standardized | Osteoarthritis knee | High body-mass index | Rate | 1990 | 43.4204294 | 118.630216 | -4.2038128 |
| YLDs (Years Lived with Disability) | Bahrain | Both | Age-standardized | Osteoarthritis knee | High body-mass index | Rate | 1990 | 43.4204294 | 118.630216 | -4.2038128 |
| DALYs (Disability-Adjusted Life Years) | Luxembourg | Both | Age-standardized | Osteoarthritis knee | High body-mass index | Rate | 1990 | 43.3545075 | 122.171977 | -3.6816381 |
| YLDs (Years Lived with Disability) | Luxembourg | Both | Age-standardized | Osteoarthritis knee | High body-mass index | Rate | 1990 | 43.3545075 | 122.171977 | -3.6816381 |
| DALYs (Disability-Adjusted Life Years) | Ireland | Both | Age-standardized | Osteoarthritis knee | High body-mass index | Rate | 1990 | 43.2672437 | 121.570367 | -3.9756511 |
| YLDs (Years Lived with Disability) | Ireland | Both | Age-standardized | Osteoarthritis knee | High body-mass index | Rate | 1990 | 43.2672437 | 121.570367 | -3.9756511 |
| DALYs (Disability-Adjusted Life Years) | Austria | Both | Age-standardized | Osteoarthritis knee | High body-mass index | Rate | 1990 | 43.0623232 | 118.881487 | -3.8132894 |
| YLDs (Years Lived with Disability) | Austria | Both | Age-standardized | Osteoarthritis knee | High body-mass index | Rate | 1990 | 43.0623232 | 118.881487 | -3.8132894 |
| DALYs (Disability-Adjusted Life Years) | Peru | Both | Age-standardized | Osteoarthritis knee | High body-mass index | Rate | 1990 | 42.7368112 | 118.066102 | -4.029755 |
| YLDs (Years Lived with Disability) | Peru | Both | Age-standardized | Osteoarthritis knee | High body-mass index | Rate | 1990 | 42.7368112 | 118.066102 | -4.029755 |
| DALYs (Disability-Adjusted Life Years) | South Africa | Both | Age-standardized | Osteoarthritis knee | High body-mass index | Rate | 1990 | 42.4981325 | 116.436108 | -3.8880802 |
| YLDs (Years Lived with Disability) | South Africa | Both | Age-standardized | Osteoarthritis knee | High body-mass index | Rate | 1990 | 42.4981325 | 116.436108 | -3.8880802 |
| DALYs (Disability-Adjusted Life Years) | Guatemala | Both | Age-standardized | Osteoarthritis knee | High body-mass index | Rate | 1990 | 42.3694218 | 118.712256 | -3.7108026 |
| YLDs (Years Lived with Disability) | Guatemala | Both | Age-standardized | Osteoarthritis knee | High body-mass index | Rate | 1990 | 42.3694218 | 118.712256 | -3.7108026 |
| DALYs (Disability-Adjusted Life Years) | Brazil | Both | Age-standardized | Osteoarthritis knee | High body-mass index | Rate | 1990 | 42.2661634 | 117.911359 | -3.7296521 |
| YLDs (Years Lived with Disability) | Brazil | Both | Age-standardized | Osteoarthritis knee | High body-mass index | Rate | 1990 | 42.2661634 | 117.911359 | -3.7296521 |
| DALYs (Disability-Adjusted Life Years) | Andorra | Both | Age-standardized | Osteoarthritis knee | High body-mass index | Rate | 1990 | 42.0405421 | 118.0004 | -4.0916945 |
| YLDs (Years Lived with Disability) | Andorra | Both | Age-standardized | Osteoarthritis knee | High body-mass index | Rate | 1990 | 42.0405421 | 118.0004 | -4.0916945 |
| DALYs (Disability-Adjusted Life Years) | Eswatini | Both | Age-standardized | Osteoarthritis knee | High body-mass index | Rate | 1990 | 42.0007925 | 115.06336 | -4.2578077 |
| YLDs (Years Lived with Disability) | Eswatini | Both | Age-standardized | Osteoarthritis knee | High body-mass index | Rate | 1990 | 42.0007925 | 115.06336 | -4.2578077 |
| DALYs (Disability-Adjusted Life Years) | Latvia | Both | Age-standardized | Osteoarthritis knee | High body-mass index | Rate | 1990 | 41.8095779 | 111.82164 | -4.3869222 |
| YLDs (Years Lived with Disability) | Latvia | Both | Age-standardized | Osteoarthritis knee | High body-mass index | Rate | 1990 | 41.8095779 | 111.82164 | -4.3869222 |
| DALYs (Disability-Adjusted Life Years) | Honduras | Both | Age-standardized | Osteoarthritis knee | High body-mass index | Rate | 1990 | 41.6417719 | 115.395606 | -3.7104454 |
| YLDs (Years Lived with Disability) | Honduras | Both | Age-standardized | Osteoarthritis knee | High body-mass index | Rate | 1990 | 41.6417719 | 115.395606 | -3.7104454 |
| DALYs (Disability-Adjusted Life Years) | Libya | Both | Age-standardized | Osteoarthritis knee | High body-mass index | Rate | 1990 | 41.5774063 | 115.774908 | -3.9086368 |
| YLDs (Years Lived with Disability) | Libya | Both | Age-standardized | Osteoarthritis knee | High body-mass index | Rate | 1990 | 41.5774063 | 115.774908 | -3.9086368 |
| DALYs (Disability-Adjusted Life Years) | Saudi Arabia | Both | Age-standardized | Osteoarthritis knee | High body-mass index | Rate | 1990 | 41.3333451 | 113.458974 | -4.2010308 |
| YLDs (Years Lived with Disability) | Saudi Arabia | Both | Age-standardized | Osteoarthritis knee | High body-mass index | Rate | 1990 | 41.3333451 | 113.458974 | -4.2010308 |
| DALYs (Disability-Adjusted Life Years) | Lebanon | Both | Age-standardized | Osteoarthritis knee | High body-mass index | Rate | 1990 | 41.1484154 | 113.679604 | -4.0427039 |
| YLDs (Years Lived with Disability) | Lebanon | Both | Age-standardized | Osteoarthritis knee | High body-mass index | Rate | 1990 | 41.1484154 | 113.679604 | -4.0427039 |
| DALYs (Disability-Adjusted Life Years) | Colombia | Both | Age-standardized | Osteoarthritis knee | High body-mass index | Rate | 1990 | 41.0830679 | 116.584684 | -3.6337697 |
| YLDs (Years Lived with Disability) | Colombia | Both | Age-standardized | Osteoarthritis knee | High body-mass index | Rate | 1990 | 41.0830679 | 116.584684 | -3.6337697 |
| DALYs (Disability-Adjusted Life Years) | Portugal | Both | Age-standardized | Osteoarthritis knee | High body-mass index | Rate | 1990 | 40.930355 | 115.554889 | -3.7799256 |
| YLDs (Years Lived with Disability) | Portugal | Both | Age-standardized | Osteoarthritis knee | High body-mass index | Rate | 1990 | 40.930355 | 115.554889 | -3.7799256 |
| DALYs (Disability-Adjusted Life Years) | Saint Lucia | Both | Age-standardized | Osteoarthritis knee | High body-mass index | Rate | 1990 | 40.86227 | 112.351582 | -3.7795445 |
| YLDs (Years Lived with Disability) | Saint Lucia | Both | Age-standardized | Osteoarthritis knee | High body-mass index | Rate | 1990 | 40.86227 | 112.351582 | -3.7795445 |
| DALYs (Disability-Adjusted Life Years) | Syrian Arab Republic | Both | Age-standardized | Osteoarthritis knee | High body-mass index | Rate | 1990 | 40.8474621 | 111.482949 | -4.0467408 |
| YLDs (Years Lived with Disability) | Syrian Arab Republic | Both | Age-standardized | Osteoarthritis knee | High body-mass index | Rate | 1990 | 40.8474621 | 111.482949 | -4.0467408 |
| DALYs (Disability-Adjusted Life Years) | Estonia | Both | Age-standardized | Osteoarthritis knee | High body-mass index | Rate | 1990 | 40.6589115 | 109.096726 | -3.6894601 |
| YLDs (Years Lived with Disability) | Estonia | Both | Age-standardized | Osteoarthritis knee | High body-mass index | Rate | 1990 | 40.6589115 | 109.096726 | -3.6894601 |
| DALYs (Disability-Adjusted Life Years) | Grenada | Both | Age-standardized | Osteoarthritis knee | High body-mass index | Rate | 1990 | 40.5158453 | 115.27346 | -3.6819732 |
| YLDs (Years Lived with Disability) | Grenada | Both | Age-standardized | Osteoarthritis knee | High body-mass index | Rate | 1990 | 40.5158453 | 115.27346 | -3.6819732 |
| DALYs (Disability-Adjusted Life Years) | Slovakia | Both | Age-standardized | Osteoarthritis knee | High body-mass index | Rate | 1990 | 40.1403137 | 108.057087 | -4.0704525 |
| YLDs (Years Lived with Disability) | Slovakia | Both | Age-standardized | Osteoarthritis knee | High body-mass index | Rate | 1990 | 40.1403137 | 108.057087 | -4.0704525 |
| DALYs (Disability-Adjusted Life Years) | Hungary | Both | Age-standardized | Osteoarthritis knee | High body-mass index | Rate | 1990 | 40.0430444 | 109.058406 | -3.5917605 |
| YLDs (Years Lived with Disability) | Hungary | Both | Age-standardized | Osteoarthritis knee | High body-mass index | Rate | 1990 | 40.0430444 | 109.058406 | -3.5917605 |
| DALYs (Disability-Adjusted Life Years) | Switzerland | Both | Age-standardized | Osteoarthritis knee | High body-mass index | Rate | 1990 | 39.8985692 | 112.718044 | -3.3745342 |
| YLDs (Years Lived with Disability) | Switzerland | Both | Age-standardized | Osteoarthritis knee | High body-mass index | Rate | 1990 | 39.8985692 | 112.718044 | -3.3745342 |
| DALYs (Disability-Adjusted Life Years) | Montenegro | Both | Age-standardized | Osteoarthritis knee | High body-mass index | Rate | 1990 | 39.8823012 | 108.214162 | -3.8853652 |
| YLDs (Years Lived with Disability) | Montenegro | Both | Age-standardized | Osteoarthritis knee | High body-mass index | Rate | 1990 | 39.8823012 | 108.214162 | -3.8853652 |
| DALYs (Disability-Adjusted Life Years) | Italy | Both | Age-standardized | Osteoarthritis knee | High body-mass index | Rate | 1990 | 39.8794653 | 114.089152 | -3.3983875 |
| YLDs (Years Lived with Disability) | Italy | Both | Age-standardized | Osteoarthritis knee | High body-mass index | Rate | 1990 | 39.8794653 | 114.089152 | -3.3983875 |
| DALYs (Disability-Adjusted Life Years) | Czechia | Both | Age-standardized | Osteoarthritis knee | High body-mass index | Rate | 1990 | 39.6966557 | 106.86204 | -4.0428941 |
| YLDs (Years Lived with Disability) | Czechia | Both | Age-standardized | Osteoarthritis knee | High body-mass index | Rate | 1990 | 39.6966557 | 106.86204 | -4.0428941 |
| DALYs (Disability-Adjusted Life Years) | Belgium | Both | Age-standardized | Osteoarthritis knee | High body-mass index | Rate | 1990 | 39.6626763 | 112.41142 | -3.2860321 |
| YLDs (Years Lived with Disability) | Belgium | Both | Age-standardized | Osteoarthritis knee | High body-mass index | Rate | 1990 | 39.6626763 | 112.41142 | -3.2860321 |
| DALYs (Disability-Adjusted Life Years) | United Arab Emirates | Both | Age-standardized | Osteoarthritis knee | High body-mass index | Rate | 1990 | 39.6416256 | 110.872666 | -3.6053187 |
| YLDs (Years Lived with Disability) | United Arab Emirates | Both | Age-standardized | Osteoarthritis knee | High body-mass index | Rate | 1990 | 39.6416256 | 110.872666 | -3.6053187 |
| DALYs (Disability-Adjusted Life Years) | Cuba | Both | Age-standardized | Osteoarthritis knee | High body-mass index | Rate | 1990 | 39.6037033 | 113.822505 | -3.2671539 |
| YLDs (Years Lived with Disability) | Cuba | Both | Age-standardized | Osteoarthritis knee | High body-mass index | Rate | 1990 | 39.6037033 | 113.822505 | -3.2671539 |
| DALYs (Disability-Adjusted Life Years) | Guyana | Both | Age-standardized | Osteoarthritis knee | High body-mass index | Rate | 1990 | 39.4698831 | 112.870134 | -3.4209678 |
| YLDs (Years Lived with Disability) | Guyana | Both | Age-standardized | Osteoarthritis knee | High body-mass index | Rate | 1990 | 39.4698831 | 112.870134 | -3.4209678 |
| DALYs (Disability-Adjusted Life Years) | Norway | Both | Age-standardized | Osteoarthritis knee | High body-mass index | Rate | 1990 | 39.1643195 | 111.210504 | -3.373627 |
| YLDs (Years Lived with Disability) | Norway | Both | Age-standardized | Osteoarthritis knee | High body-mass index | Rate | 1990 | 39.1643195 | 111.210504 | -3.373627 |
| DALYs (Disability-Adjusted Life Years) | Lithuania | Both | Age-standardized | Osteoarthritis knee | High body-mass index | Rate | 1990 | 38.9812129 | 108.659272 | -3.6774896 |
| YLDs (Years Lived with Disability) | Lithuania | Both | Age-standardized | Osteoarthritis knee | High body-mass index | Rate | 1990 | 38.9812129 | 108.659272 | -3.6774896 |
| DALYs (Disability-Adjusted Life Years) | Cameroon | Both | Age-standardized | Osteoarthritis knee | High body-mass index | Rate | 1990 | 38.7465645 | 109.727427 | -3.3191945 |
| YLDs (Years Lived with Disability) | Cameroon | Both | Age-standardized | Osteoarthritis knee | High body-mass index | Rate | 1990 | 38.7465645 | 109.727427 | -3.3191945 |
| DALYs (Disability-Adjusted Life Years) | Malta | Both | Age-standardized | Osteoarthritis knee | High body-mass index | Rate | 1990 | 38.7022646 | 112.258746 | -3.3076416 |
| YLDs (Years Lived with Disability) | Malta | Both | Age-standardized | Osteoarthritis knee | High body-mass index | Rate | 1990 | 38.7022646 | 112.258746 | -3.3076416 |
| DALYs (Disability-Adjusted Life Years) | Republic of Moldova | Both | Age-standardized | Osteoarthritis knee | High body-mass index | Rate | 1990 | 38.5659825 | 106.844232 | -3.6454543 |
| YLDs (Years Lived with Disability) | Republic of Moldova | Both | Age-standardized | Osteoarthritis knee | High body-mass index | Rate | 1990 | 38.5659825 | 106.844232 | -3.6454543 |
| DALYs (Disability-Adjusted Life Years) | Bolivia (Plurinational State of) | Both | Age-standardized | Osteoarthritis knee | High body-mass index | Rate | 1990 | 38.3492778 | 107.725145 | -3.3032271 |
| YLDs (Years Lived with Disability) | Bolivia (Plurinational State of) | Both | Age-standardized | Osteoarthritis knee | High body-mass index | Rate | 1990 | 38.3492778 | 107.725145 | -3.3032271 |
| DALYs (Disability-Adjusted Life Years) | Slovenia | Both | Age-standardized | Osteoarthritis knee | High body-mass index | Rate | 1990 | 38.2652182 | 103.866445 | -3.7111871 |
| YLDs (Years Lived with Disability) | Slovenia | Both | Age-standardized | Osteoarthritis knee | High body-mass index | Rate | 1990 | 38.2652182 | 103.866445 | -3.7111871 |
| DALYs (Disability-Adjusted Life Years) | Ukraine | Both | Age-standardized | Osteoarthritis knee | High body-mass index | Rate | 1990 | 38.1100239 | 104.088424 | -3.446082 |
| YLDs (Years Lived with Disability) | Ukraine | Both | Age-standardized | Osteoarthritis knee | High body-mass index | Rate | 1990 | 38.1100239 | 104.088424 | -3.446082 |
| DALYs (Disability-Adjusted Life Years) | Denmark | Both | Age-standardized | Osteoarthritis knee | High body-mass index | Rate | 1990 | 38.0129451 | 110.064457 | -3.4222515 |
| YLDs (Years Lived with Disability) | Denmark | Both | Age-standardized | Osteoarthritis knee | High body-mass index | Rate | 1990 | 38.0129451 | 110.064457 | -3.4222515 |
| DALYs (Disability-Adjusted Life Years) | Bulgaria | Both | Age-standardized | Osteoarthritis knee | High body-mass index | Rate | 1990 | 37.9405679 | 104.518177 | -3.7163697 |
| YLDs (Years Lived with Disability) | Bulgaria | Both | Age-standardized | Osteoarthritis knee | High body-mass index | Rate | 1990 | 37.9405679 | 104.518177 | -3.7163697 |
| DALYs (Disability-Adjusted Life Years) | Brunei Darussalam | Both | Age-standardized | Osteoarthritis knee | High body-mass index | Rate | 1990 | 37.8488913 | 112.824471 | -3.0311942 |
| YLDs (Years Lived with Disability) | Brunei Darussalam | Both | Age-standardized | Osteoarthritis knee | High body-mass index | Rate | 1990 | 37.8488913 | 112.824471 | -3.0311942 |
| DALYs (Disability-Adjusted Life Years) | Belarus | Both | Age-standardized | Osteoarthritis knee | High body-mass index | Rate | 1990 | 37.6149618 | 103.968043 | -3.541046 |
| YLDs (Years Lived with Disability) | Belarus | Both | Age-standardized | Osteoarthritis knee | High body-mass index | Rate | 1990 | 37.6149618 | 103.968043 | -3.541046 |
| DALYs (Disability-Adjusted Life Years) | Russian Federation | Both | Age-standardized | Osteoarthritis knee | High body-mass index | Rate | 1990 | 37.2102901 | 103.022618 | -3.4979872 |
| YLDs (Years Lived with Disability) | Russian Federation | Both | Age-standardized | Osteoarthritis knee | High body-mass index | Rate | 1990 | 37.2102901 | 103.022618 | -3.4979872 |
| DALYs (Disability-Adjusted Life Years) | Japan | Both | Age-standardized | Osteoarthritis knee | High body-mass index | Rate | 1990 | 37.1424258 | 110.971205 | -2.9644445 |
| YLDs (Years Lived with Disability) | Japan | Both | Age-standardized | Osteoarthritis knee | High body-mass index | Rate | 1990 | 37.1424258 | 110.971205 | -2.9644445 |
| DALYs (Disability-Adjusted Life Years) | Mauritania | Both | Age-standardized | Osteoarthritis knee | High body-mass index | Rate | 1990 | 37.0264888 | 103.073059 | -3.1470899 |
| YLDs (Years Lived with Disability) | Mauritania | Both | Age-standardized | Osteoarthritis knee | High body-mass index | Rate | 1990 | 37.0264888 | 103.073059 | -3.1470899 |
| DALYs (Disability-Adjusted Life Years) | France | Both | Age-standardized | Osteoarthritis knee | High body-mass index | Rate | 1990 | 36.9396643 | 108.316544 | -3.2835107 |
| YLDs (Years Lived with Disability) | France | Both | Age-standardized | Osteoarthritis knee | High body-mass index | Rate | 1990 | 36.9396643 | 108.316544 | -3.2835107 |
| DALYs (Disability-Adjusted Life Years) | Republic of Korea | Both | Age-standardized | Osteoarthritis knee | High body-mass index | Rate | 1990 | 36.6889165 | 105.294889 | -3.3047489 |
| YLDs (Years Lived with Disability) | Republic of Korea | Both | Age-standardized | Osteoarthritis knee | High body-mass index | Rate | 1990 | 36.6889165 | 105.294889 | -3.3047489 |
| DALYs (Disability-Adjusted Life Years) | Croatia | Both | Age-standardized | Osteoarthritis knee | High body-mass index | Rate | 1990 | 36.547001 | 100.919434 | -3.5677834 |
| YLDs (Years Lived with Disability) | Croatia | Both | Age-standardized | Osteoarthritis knee | High body-mass index | Rate | 1990 | 36.547001 | 100.919434 | -3.5677834 |
| DALYs (Disability-Adjusted Life Years) | Vanuatu | Both | Age-standardized | Osteoarthritis knee | High body-mass index | Rate | 1990 | 36.4771179 | 100.738253 | -3.2047511 |
| YLDs (Years Lived with Disability) | Vanuatu | Both | Age-standardized | Osteoarthritis knee | High body-mass index | Rate | 1990 | 36.4771179 | 100.738253 | -3.2047511 |
| DALYs (Disability-Adjusted Life Years) | Solomon Islands | Both | Age-standardized | Osteoarthritis knee | High body-mass index | Rate | 1990 | 36.3957436 | 104.558387 | -3.0498427 |
| YLDs (Years Lived with Disability) | Solomon Islands | Both | Age-standardized | Osteoarthritis knee | High body-mass index | Rate | 1990 | 36.3957436 | 104.558387 | -3.0498427 |
| DALYs (Disability-Adjusted Life Years) | North Macedonia | Both | Age-standardized | Osteoarthritis knee | High body-mass index | Rate | 1990 | 36.2896764 | 97.8310762 | -3.5547112 |
| YLDs (Years Lived with Disability) | North Macedonia | Both | Age-standardized | Osteoarthritis knee | High body-mass index | Rate | 1990 | 36.2896764 | 97.8310762 | -3.5547112 |
| DALYs (Disability-Adjusted Life Years) | Suriname | Both | Age-standardized | Osteoarthritis knee | High body-mass index | Rate | 1990 | 35.9880225 | 102.53938 | -3.1428019 |
| YLDs (Years Lived with Disability) | Suriname | Both | Age-standardized | Osteoarthritis knee | High body-mass index | Rate | 1990 | 35.9880225 | 102.53938 | -3.1428019 |
| DALYs (Disability-Adjusted Life Years) | Cyprus | Both | Age-standardized | Osteoarthritis knee | High body-mass index | Rate | 1990 | 35.8535026 | 103.075 | -2.95867 |
| YLDs (Years Lived with Disability) | Cyprus | Both | Age-standardized | Osteoarthritis knee | High body-mass index | Rate | 1990 | 35.8535026 | 103.075 | -2.95867 |
| DALYs (Disability-Adjusted Life Years) | Dominican Republic | Both | Age-standardized | Osteoarthritis knee | High body-mass index | Rate | 1990 | 35.8146085 | 99.9883695 | -3.0089172 |
| YLDs (Years Lived with Disability) | Dominican Republic | Both | Age-standardized | Osteoarthritis knee | High body-mass index | Rate | 1990 | 35.8146085 | 99.9883695 | -3.0089172 |
| DALYs (Disability-Adjusted Life Years) | Serbia | Both | Age-standardized | Osteoarthritis knee | High body-mass index | Rate | 1990 | 35.5244711 | 97.7540158 | -3.4598721 |
| YLDs (Years Lived with Disability) | Serbia | Both | Age-standardized | Osteoarthritis knee | High body-mass index | Rate | 1990 | 35.5244711 | 97.7540158 | -3.4598721 |
| DALYs (Disability-Adjusted Life Years) | Poland | Both | Age-standardized | Osteoarthritis knee | High body-mass index | Rate | 1990 | 35.510102 | 97.1085704 | -3.335787 |
| YLDs (Years Lived with Disability) | Poland | Both | Age-standardized | Osteoarthritis knee | High body-mass index | Rate | 1990 | 35.510102 | 97.1085704 | -3.335787 |
| DALYs (Disability-Adjusted Life Years) | Saint Vincent and the Grenadines | Both | Age-standardized | Osteoarthritis knee | High body-mass index | Rate | 1990 | 35.4763324 | 104.324464 | -2.9030119 |
| YLDs (Years Lived with Disability) | Saint Vincent and the Grenadines | Both | Age-standardized | Osteoarthritis knee | High body-mass index | Rate | 1990 | 35.4763324 | 104.324464 | -2.9030119 |
| DALYs (Disability-Adjusted Life Years) | Singapore | Both | Age-standardized | Osteoarthritis knee | High body-mass index | Rate | 1990 | 35.0771116 | 101.994329 | -2.7343733 |
| YLDs (Years Lived with Disability) | Singapore | Both | Age-standardized | Osteoarthritis knee | High body-mass index | Rate | 1990 | 35.0771116 | 101.994329 | -2.7343733 |
| DALYs (Disability-Adjusted Life Years) | Romania | Both | Age-standardized | Osteoarthritis knee | High body-mass index | Rate | 1990 | 34.7206294 | 96.318332 | -2.9571835 |
| YLDs (Years Lived with Disability) | Romania | Both | Age-standardized | Osteoarthritis knee | High body-mass index | Rate | 1990 | 34.7206294 | 96.318332 | -2.9571835 |
| DALYs (Disability-Adjusted Life Years) | Liberia | Both | Age-standardized | Osteoarthritis knee | High body-mass index | Rate | 1990 | 34.1049192 | 95.2760862 | -2.869137 |
| YLDs (Years Lived with Disability) | Liberia | Both | Age-standardized | Osteoarthritis knee | High body-mass index | Rate | 1990 | 34.1049192 | 95.2760862 | -2.869137 |
| DALYs (Disability-Adjusted Life Years) | Taiwan (Province of China) | Both | Age-standardized | Osteoarthritis knee | High body-mass index | Rate | 1990 | 34.0337995 | 99.7674697 | -2.8976141 |
| YLDs (Years Lived with Disability) | Taiwan (Province of China) | Both | Age-standardized | Osteoarthritis knee | High body-mass index | Rate | 1990 | 34.0337995 | 99.7674697 | -2.8976141 |
| DALYs (Disability-Adjusted Life Years) | Seychelles | Both | Age-standardized | Osteoarthritis knee | High body-mass index | Rate | 1990 | 33.7861947 | 93.9691965 | -3.2208444 |
| YLDs (Years Lived with Disability) | Seychelles | Both | Age-standardized | Osteoarthritis knee | High body-mass index | Rate | 1990 | 33.7861947 | 93.9691965 | -3.2208444 |
| DALYs (Disability-Adjusted Life Years) | Albania | Both | Age-standardized | Osteoarthritis knee | High body-mass index | Rate | 1990 | 33.7164825 | 90.4619052 | -3.3033448 |
| YLDs (Years Lived with Disability) | Albania | Both | Age-standardized | Osteoarthritis knee | High body-mass index | Rate | 1990 | 33.7164825 | 90.4619052 | -3.3033448 |
| DALYs (Disability-Adjusted Life Years) | Lesotho | Both | Age-standardized | Osteoarthritis knee | High body-mass index | Rate | 1990 | 33.6935033 | 92.0570439 | -3.0852281 |
| YLDs (Years Lived with Disability) | Lesotho | Both | Age-standardized | Osteoarthritis knee | High body-mass index | Rate | 1990 | 33.6935033 | 92.0570439 | -3.0852281 |
| DALYs (Disability-Adjusted Life Years) | Tunisia | Both | Age-standardized | Osteoarthritis knee | High body-mass index | Rate | 1990 | 33.6512746 | 96.6959799 | -3.1846464 |
| YLDs (Years Lived with Disability) | Tunisia | Both | Age-standardized | Osteoarthritis knee | High body-mass index | Rate | 1990 | 33.6512746 | 96.6959799 | -3.1846464 |
| DALYs (Disability-Adjusted Life Years) | Gabon | Both | Age-standardized | Osteoarthritis knee | High body-mass index | Rate | 1990 | 33.5694761 | 94.0956575 | -2.9969213 |
| YLDs (Years Lived with Disability) | Gabon | Both | Age-standardized | Osteoarthritis knee | High body-mass index | Rate | 1990 | 33.5694761 | 94.0956575 | -2.9969213 |
| DALYs (Disability-Adjusted Life Years) | Sweden | Both | Age-standardized | Osteoarthritis knee | High body-mass index | Rate | 1990 | 33.2869004 | 95.7713945 | -3.1500184 |
| YLDs (Years Lived with Disability) | Sweden | Both | Age-standardized | Osteoarthritis knee | High body-mass index | Rate | 1990 | 33.2869004 | 95.7713945 | -3.1500184 |
| DALYs (Disability-Adjusted Life Years) | Algeria | Both | Age-standardized | Osteoarthritis knee | High body-mass index | Rate | 1990 | 33.1272723 | 95.4264302 | -2.9689094 |
| YLDs (Years Lived with Disability) | Algeria | Both | Age-standardized | Osteoarthritis knee | High body-mass index | Rate | 1990 | 33.1272723 | 95.4264302 | -2.9689094 |
| DALYs (Disability-Adjusted Life Years) | Greenland | Both | Age-standardized | Osteoarthritis knee | High body-mass index | Rate | 1990 | 32.9497551 | 90.4181971 | -3.1686918 |
| YLDs (Years Lived with Disability) | Greenland | Both | Age-standardized | Osteoarthritis knee | High body-mass index | Rate | 1990 | 32.9497551 | 90.4181971 | -3.1686918 |
| DALYs (Disability-Adjusted Life Years) | Bosnia and Herzegovina | Both | Age-standardized | Osteoarthritis knee | High body-mass index | Rate | 1990 | 32.8355343 | 91.8227111 | -3.0824935 |
| YLDs (Years Lived with Disability) | Bosnia and Herzegovina | Both | Age-standardized | Osteoarthritis knee | High body-mass index | Rate | 1990 | 32.8355343 | 91.8227111 | -3.0824935 |
| DALYs (Disability-Adjusted Life Years) | Oman | Both | Age-standardized | Osteoarthritis knee | High body-mass index | Rate | 1990 | 32.5021175 | 91.4670773 | -2.8932987 |
| YLDs (Years Lived with Disability) | Oman | Both | Age-standardized | Osteoarthritis knee | High body-mass index | Rate | 1990 | 32.5021175 | 91.4670773 | -2.8932987 |
| DALYs (Disability-Adjusted Life Years) | Morocco | Both | Age-standardized | Osteoarthritis knee | High body-mass index | Rate | 1990 | 32.3108028 | 89.1907734 | -3.2661765 |
| YLDs (Years Lived with Disability) | Morocco | Both | Age-standardized | Osteoarthritis knee | High body-mass index | Rate | 1990 | 32.3108028 | 89.1907734 | -3.2661765 |
| DALYs (Disability-Adjusted Life Years) | Sao Tome and Principe | Both | Age-standardized | Osteoarthritis knee | High body-mass index | Rate | 1990 | 32.1672804 | 92.0791823 | -2.8272552 |
| YLDs (Years Lived with Disability) | Sao Tome and Principe | Both | Age-standardized | Osteoarthritis knee | High body-mass index | Rate | 1990 | 32.1672804 | 92.0791823 | -2.8272552 |
| DALYs (Disability-Adjusted Life Years) | Georgia | Both | Age-standardized | Osteoarthritis knee | High body-mass index | Rate | 1990 | 31.4053848 | 87.2299779 | -2.8690556 |
| YLDs (Years Lived with Disability) | Georgia | Both | Age-standardized | Osteoarthritis knee | High body-mass index | Rate | 1990 | 31.4053848 | 87.2299779 | -2.8690556 |
| DALYs (Disability-Adjusted Life Years) | Kazakhstan | Both | Age-standardized | Osteoarthritis knee | High body-mass index | Rate | 1990 | 30.9425712 | 84.4027261 | -2.7894199 |
| YLDs (Years Lived with Disability) | Kazakhstan | Both | Age-standardized | Osteoarthritis knee | High body-mass index | Rate | 1990 | 30.9425712 | 84.4027261 | -2.7894199 |
| DALYs (Disability-Adjusted Life Years) | Iran (Islamic Republic of) | Both | Age-standardized | Osteoarthritis knee | High body-mass index | Rate | 1990 | 30.5822976 | 87.5073558 | -2.6137203 |
| YLDs (Years Lived with Disability) | Iran (Islamic Republic of) | Both | Age-standardized | Osteoarthritis knee | High body-mass index | Rate | 1990 | 30.5822976 | 87.5073558 | -2.6137203 |
| DALYs (Disability-Adjusted Life Years) | Armenia | Both | Age-standardized | Osteoarthritis knee | High body-mass index | Rate | 1990 | 30.5142436 | 84.5622091 | -2.9628365 |
| YLDs (Years Lived with Disability) | Armenia | Both | Age-standardized | Osteoarthritis knee | High body-mass index | Rate | 1990 | 30.5142436 | 84.5622091 | -2.9628365 |
| DALYs (Disability-Adjusted Life Years) | Sudan | Both | Age-standardized | Osteoarthritis knee | High body-mass index | Rate | 1990 | 30.3852266 | 86.8563288 | -2.5260982 |
| YLDs (Years Lived with Disability) | Sudan | Both | Age-standardized | Osteoarthritis knee | High body-mass index | Rate | 1990 | 30.3852266 | 86.8563288 | -2.5260982 |
| DALYs (Disability-Adjusted Life Years) | Azerbaijan | Both | Age-standardized | Osteoarthritis knee | High body-mass index | Rate | 1990 | 30.2741171 | 83.6958286 | -2.7027694 |
| YLDs (Years Lived with Disability) | Azerbaijan | Both | Age-standardized | Osteoarthritis knee | High body-mass index | Rate | 1990 | 30.2741171 | 83.6958286 | -2.7027694 |
| DALYs (Disability-Adjusted Life Years) | Kyrgyzstan | Both | Age-standardized | Osteoarthritis knee | High body-mass index | Rate | 1990 | 29.7004213 | 82.1666484 | -2.6724492 |
| YLDs (Years Lived with Disability) | Kyrgyzstan | Both | Age-standardized | Osteoarthritis knee | High body-mass index | Rate | 1990 | 29.7004213 | 82.1666484 | -2.6724492 |
| DALYs (Disability-Adjusted Life Years) | Uzbekistan | Both | Age-standardized | Osteoarthritis knee | High body-mass index | Rate | 1990 | 29.4463901 | 82.8872879 | -2.8790418 |
| YLDs (Years Lived with Disability) | Uzbekistan | Both | Age-standardized | Osteoarthritis knee | High body-mass index | Rate | 1990 | 29.4463901 | 82.8872879 | -2.8790418 |
| DALYs (Disability-Adjusted Life Years) | Turkmenistan | Both | Age-standardized | Osteoarthritis knee | High body-mass index | Rate | 1990 | 29.3545717 | 81.3467768 | -2.8619486 |
| YLDs (Years Lived with Disability) | Turkmenistan | Both | Age-standardized | Osteoarthritis knee | High body-mass index | Rate | 1990 | 29.3545717 | 81.3467768 | -2.8619486 |
| DALYs (Disability-Adjusted Life Years) | Canada | Both | Age-standardized | Osteoarthritis knee | High body-mass index | Rate | 1990 | 29.2393938 | 80.473597 | -2.8250963 |
| YLDs (Years Lived with Disability) | Canada | Both | Age-standardized | Osteoarthritis knee | High body-mass index | Rate | 1990 | 29.2393938 | 80.473597 | -2.8250963 |
| DALYs (Disability-Adjusted Life Years) | Papua New Guinea | Both | Age-standardized | Osteoarthritis knee | High body-mass index | Rate | 1990 | 28.8656021 | 82.7541774 | -2.4686686 |
| YLDs (Years Lived with Disability) | Papua New Guinea | Both | Age-standardized | Osteoarthritis knee | High body-mass index | Rate | 1990 | 28.8656021 | 82.7541774 | -2.4686686 |
| DALYs (Disability-Adjusted Life Years) | Cabo Verde | Both | Age-standardized | Osteoarthritis knee | High body-mass index | Rate | 1990 | 28.6768787 | 85.0274709 | -2.33778 |
| YLDs (Years Lived with Disability) | Cabo Verde | Both | Age-standardized | Osteoarthritis knee | High body-mass index | Rate | 1990 | 28.6768787 | 85.0274709 | -2.33778 |
| DALYs (Disability-Adjusted Life Years) | Benin | Both | Age-standardized | Osteoarthritis knee | High body-mass index | Rate | 1990 | 28.5526487 | 82.4097904 | -2.3346101 |
| YLDs (Years Lived with Disability) | Benin | Both | Age-standardized | Osteoarthritis knee | High body-mass index | Rate | 1990 | 28.5526487 | 82.4097904 | -2.3346101 |
| DALYs (Disability-Adjusted Life Years) | Bhutan | Both | Age-standardized | Osteoarthritis knee | High body-mass index | Rate | 1990 | 28.4169107 | 80.6036026 | -2.5405041 |
| YLDs (Years Lived with Disability) | Bhutan | Both | Age-standardized | Osteoarthritis knee | High body-mass index | Rate | 1990 | 28.4169107 | 80.6036026 | -2.5405041 |
| DALYs (Disability-Adjusted Life Years) | Gambia | Both | Age-standardized | Osteoarthritis knee | High body-mass index | Rate | 1990 | 28.3069853 | 81.8864537 | -2.3086827 |
| YLDs (Years Lived with Disability) | Gambia | Both | Age-standardized | Osteoarthritis knee | High body-mass index | Rate | 1990 | 28.3069853 | 81.8864537 | -2.3086827 |
| DALYs (Disability-Adjusted Life Years) | C么te d'Ivoire | Both | Age-standardized | Osteoarthritis knee | High body-mass index | Rate | 1990 | 27.8314372 | 82.0396218 | -2.1701741 |
| YLDs (Years Lived with Disability) | C么te d'Ivoire | Both | Age-standardized | Osteoarthritis knee | High body-mass index | Rate | 1990 | 27.8314372 | 82.0396218 | -2.1701741 |
| DALYs (Disability-Adjusted Life Years) | Senegal | Both | Age-standardized | Osteoarthritis knee | High body-mass index | Rate | 1990 | 27.3948039 | 79.0857062 | -2.2265588 |
| YLDs (Years Lived with Disability) | Senegal | Both | Age-standardized | Osteoarthritis knee | High body-mass index | Rate | 1990 | 27.3948039 | 79.0857062 | -2.2265588 |
| DALYs (Disability-Adjusted Life Years) | Namibia | Both | Age-standardized | Osteoarthritis knee | High body-mass index | Rate | 1990 | 27.275732 | 82.4786306 | -2.0795958 |
| YLDs (Years Lived with Disability) | Namibia | Both | Age-standardized | Osteoarthritis knee | High body-mass index | Rate | 1990 | 27.275732 | 82.4786306 | -2.0795958 |
| DALYs (Disability-Adjusted Life Years) | Afghanistan | Both | Age-standardized | Osteoarthritis knee | High body-mass index | Rate | 1990 | 27.1984362 | 77.3138905 | -2.1985867 |
| YLDs (Years Lived with Disability) | Afghanistan | Both | Age-standardized | Osteoarthritis knee | High body-mass index | Rate | 1990 | 27.1984362 | 77.3138905 | -2.1985867 |
| DALYs (Disability-Adjusted Life Years) | Tajikistan | Both | Age-standardized | Osteoarthritis knee | High body-mass index | Rate | 1990 | 27.0103522 | 76.1839698 | -2.4553475 |
| YLDs (Years Lived with Disability) | Tajikistan | Both | Age-standardized | Osteoarthritis knee | High body-mass index | Rate | 1990 | 27.0103522 | 76.1839698 | -2.4553475 |
| DALYs (Disability-Adjusted Life Years) | Mauritius | Both | Age-standardized | Osteoarthritis knee | High body-mass index | Rate | 1990 | 26.9336046 | 76.7975046 | -2.1304421 |
| YLDs (Years Lived with Disability) | Mauritius | Both | Age-standardized | Osteoarthritis knee | High body-mass index | Rate | 1990 | 26.9336046 | 76.7975046 | -2.1304421 |
| DALYs (Disability-Adjusted Life Years) | Botswana | Both | Age-standardized | Osteoarthritis knee | High body-mass index | Rate | 1990 | 26.9030897 | 73.3962583 | -2.1431498 |
| YLDs (Years Lived with Disability) | Botswana | Both | Age-standardized | Osteoarthritis knee | High body-mass index | Rate | 1990 | 26.9030897 | 73.3962583 | -2.1431498 |
| DALYs (Disability-Adjusted Life Years) | China | Both | Age-standardized | Osteoarthritis knee | High body-mass index | Rate | 1990 | 26.338721 | 78.6970304 | -2.1480243 |
| YLDs (Years Lived with Disability) | China | Both | Age-standardized | Osteoarthritis knee | High body-mass index | Rate | 1990 | 26.338721 | 78.6970304 | -2.1480243 |
| DALYs (Disability-Adjusted Life Years) | Mongolia | Both | Age-standardized | Osteoarthritis knee | High body-mass index | Rate | 1990 | 25.9861103 | 74.7634316 | -2.3457467 |
| YLDs (Years Lived with Disability) | Mongolia | Both | Age-standardized | Osteoarthritis knee | High body-mass index | Rate | 1990 | 25.9861103 | 74.7634316 | -2.3457467 |
| DALYs (Disability-Adjusted Life Years) | Nigeria | Both | Age-standardized | Osteoarthritis knee | High body-mass index | Rate | 1990 | 25.4843867 | 75.1615913 | -2.0403022 |
| YLDs (Years Lived with Disability) | Nigeria | Both | Age-standardized | Osteoarthritis knee | High body-mass index | Rate | 1990 | 25.4843867 | 75.1615913 | -2.0403022 |
| DALYs (Disability-Adjusted Life Years) | Malaysia | Both | Age-standardized | Osteoarthritis knee | High body-mass index | Rate | 1990 | 25.202854 | 72.2184025 | -2.0637901 |
| YLDs (Years Lived with Disability) | Malaysia | Both | Age-standardized | Osteoarthritis knee | High body-mass index | Rate | 1990 | 25.202854 | 72.2184025 | -2.0637901 |
| DALYs (Disability-Adjusted Life Years) | Zimbabwe | Both | Age-standardized | Osteoarthritis knee | High body-mass index | Rate | 1990 | 24.4792133 | 72.5673564 | -1.8390449 |
| YLDs (Years Lived with Disability) | Zimbabwe | Both | Age-standardized | Osteoarthritis knee | High body-mass index | Rate | 1990 | 24.4792133 | 72.5673564 | -1.8390449 |
| DALYs (Disability-Adjusted Life Years) | Togo | Both | Age-standardized | Osteoarthritis knee | High body-mass index | Rate | 1990 | 24.4388249 | 70.3205975 | -1.8590035 |
| YLDs (Years Lived with Disability) | Togo | Both | Age-standardized | Osteoarthritis knee | High body-mass index | Rate | 1990 | 24.4388249 | 70.3205975 | -1.8590035 |
| DALYs (Disability-Adjusted Life Years) | Equatorial Guinea | Both | Age-standardized | Osteoarthritis knee | High body-mass index | Rate | 1990 | 23.655618 | 68.9913348 | -1.8129316 |
| YLDs (Years Lived with Disability) | Equatorial Guinea | Both | Age-standardized | Osteoarthritis knee | High body-mass index | Rate | 1990 | 23.655618 | 68.9913348 | -1.8129316 |
| DALYs (Disability-Adjusted Life Years) | Guinea-Bissau | Both | Age-standardized | Osteoarthritis knee | High body-mass index | Rate | 1990 | 23.4143854 | 68.6563892 | -1.8962429 |
| YLDs (Years Lived with Disability) | Guinea-Bissau | Both | Age-standardized | Osteoarthritis knee | High body-mass index | Rate | 1990 | 23.4143854 | 68.6563892 | -1.8962429 |
| DALYs (Disability-Adjusted Life Years) | Congo | Both | Age-standardized | Osteoarthritis knee | High body-mass index | Rate | 1990 | 23.3258328 | 67.5423555 | -1.9371744 |
| YLDs (Years Lived with Disability) | Congo | Both | Age-standardized | Osteoarthritis knee | High body-mass index | Rate | 1990 | 23.3258328 | 67.5423555 | -1.9371744 |
| DALYs (Disability-Adjusted Life Years) | Ghana | Both | Age-standardized | Osteoarthritis knee | High body-mass index | Rate | 1990 | 23.0392805 | 69.6678976 | -1.7682282 |
| YLDs (Years Lived with Disability) | Ghana | Both | Age-standardized | Osteoarthritis knee | High body-mass index | Rate | 1990 | 23.0392805 | 69.6678976 | -1.7682282 |
| DALYs (Disability-Adjusted Life Years) | Sierra Leone | Both | Age-standardized | Osteoarthritis knee | High body-mass index | Rate | 1990 | 22.7272488 | 67.6090824 | -1.7064104 |
| YLDs (Years Lived with Disability) | Sierra Leone | Both | Age-standardized | Osteoarthritis knee | High body-mass index | Rate | 1990 | 22.7272488 | 67.6090824 | -1.7064104 |
| DALYs (Disability-Adjusted Life Years) | Guinea | Both | Age-standardized | Osteoarthritis knee | High body-mass index | Rate | 1990 | 22.7072446 | 66.8163247 | -1.792867 |
| YLDs (Years Lived with Disability) | Guinea | Both | Age-standardized | Osteoarthritis knee | High body-mass index | Rate | 1990 | 22.7072446 | 66.8163247 | -1.792867 |
| DALYs (Disability-Adjusted Life Years) | United Republic of Tanzania | Both | Age-standardized | Osteoarthritis knee | High body-mass index | Rate | 1990 | 22.2425598 | 66.5237916 | -1.7083625 |
| YLDs (Years Lived with Disability) | United Republic of Tanzania | Both | Age-standardized | Osteoarthritis knee | High body-mass index | Rate | 1990 | 22.2425598 | 66.5237916 | -1.7083625 |
| DALYs (Disability-Adjusted Life Years) | Yemen | Both | Age-standardized | Osteoarthritis knee | High body-mass index | Rate | 1990 | 21.1896781 | 62.2446294 | -1.5252394 |
| YLDs (Years Lived with Disability) | Yemen | Both | Age-standardized | Osteoarthritis knee | High body-mass index | Rate | 1990 | 21.1896781 | 62.2446294 | -1.5252394 |
| DALYs (Disability-Adjusted Life Years) | Mali | Both | Age-standardized | Osteoarthritis knee | High body-mass index | Rate | 1990 | 20.2777017 | 60.2841577 | -1.5625442 |
| YLDs (Years Lived with Disability) | Mali | Both | Age-standardized | Osteoarthritis knee | High body-mass index | Rate | 1990 | 20.2777017 | 60.2841577 | -1.5625442 |
| DALYs (Disability-Adjusted Life Years) | Chad | Both | Age-standardized | Osteoarthritis knee | High body-mass index | Rate | 1990 | 20.2128824 | 62.4088747 | -1.5293866 |
| YLDs (Years Lived with Disability) | Chad | Both | Age-standardized | Osteoarthritis knee | High body-mass index | Rate | 1990 | 20.2128824 | 62.4088747 | -1.5293866 |
| DALYs (Disability-Adjusted Life Years) | Democratic People's Republic of Korea | Both | Age-standardized | Osteoarthritis knee | High body-mass index | Rate | 1990 | 20.1287477 | 58.4732333 | -1.8228466 |
| YLDs (Years Lived with Disability) | Democratic People's Republic of Korea | Both | Age-standardized | Osteoarthritis knee | High body-mass index | Rate | 1990 | 20.1287477 | 58.4732333 | -1.8228466 |
| DALYs (Disability-Adjusted Life Years) | Niger | Both | Age-standardized | Osteoarthritis knee | High body-mass index | Rate | 1990 | 20.0173899 | 59.2494882 | -1.511159 |
| YLDs (Years Lived with Disability) | Niger | Both | Age-standardized | Osteoarthritis knee | High body-mass index | Rate | 1990 | 20.0173899 | 59.2494882 | -1.511159 |
| DALYs (Disability-Adjusted Life Years) | Thailand | Both | Age-standardized | Osteoarthritis knee | High body-mass index | Rate | 1990 | 19.0370611 | 54.8005834 | -1.5243999 |
| YLDs (Years Lived with Disability) | Thailand | Both | Age-standardized | Osteoarthritis knee | High body-mass index | Rate | 1990 | 19.0370611 | 54.8005834 | -1.5243999 |
| DALYs (Disability-Adjusted Life Years) | Comoros | Both | Age-standardized | Osteoarthritis knee | High body-mass index | Rate | 1990 | 19.0175747 | 58.1330549 | -1.4498268 |
| YLDs (Years Lived with Disability) | Comoros | Both | Age-standardized | Osteoarthritis knee | High body-mass index | Rate | 1990 | 19.0175747 | 58.1330549 | -1.4498268 |
| DALYs (Disability-Adjusted Life Years) | Zambia | Both | Age-standardized | Osteoarthritis knee | High body-mass index | Rate | 1990 | 18.9838468 | 56.2084543 | -1.436987 |
| YLDs (Years Lived with Disability) | Zambia | Both | Age-standardized | Osteoarthritis knee | High body-mass index | Rate | 1990 | 18.9838468 | 56.2084543 | -1.436987 |
| DALYs (Disability-Adjusted Life Years) | Kenya | Both | Age-standardized | Osteoarthritis knee | High body-mass index | Rate | 1990 | 18.9654134 | 55.4602569 | -1.4760802 |
| YLDs (Years Lived with Disability) | Kenya | Both | Age-standardized | Osteoarthritis knee | High body-mass index | Rate | 1990 | 18.9654134 | 55.4602569 | -1.4760802 |
| DALYs (Disability-Adjusted Life Years) | Maldives | Both | Age-standardized | Osteoarthritis knee | High body-mass index | Rate | 1990 | 18.3305576 | 54.1403399 | -1.4611692 |
| YLDs (Years Lived with Disability) | Maldives | Both | Age-standardized | Osteoarthritis knee | High body-mass index | Rate | 1990 | 18.3305576 | 54.1403399 | -1.4611692 |
| DALYs (Disability-Adjusted Life Years) | Haiti | Both | Age-standardized | Osteoarthritis knee | High body-mass index | Rate | 1990 | 17.9575962 | 54.0910171 | -1.3429925 |
| YLDs (Years Lived with Disability) | Haiti | Both | Age-standardized | Osteoarthritis knee | High body-mass index | Rate | 1990 | 17.9575962 | 54.0910171 | -1.3429925 |
| DALYs (Disability-Adjusted Life Years) | Sri Lanka | Both | Age-standardized | Osteoarthritis knee | High body-mass index | Rate | 1990 | 17.8844476 | 52.1248762 | -1.2648063 |
| YLDs (Years Lived with Disability) | Sri Lanka | Both | Age-standardized | Osteoarthritis knee | High body-mass index | Rate | 1990 | 17.8844476 | 52.1248762 | -1.2648063 |
| DALYs (Disability-Adjusted Life Years) | Democratic Republic of the Congo | Both | Age-standardized | Osteoarthritis knee | High body-mass index | Rate | 1990 | 17.4354997 | 50.4108904 | -1.1746372 |
| YLDs (Years Lived with Disability) | Democratic Republic of the Congo | Both | Age-standardized | Osteoarthritis knee | High body-mass index | Rate | 1990 | 17.4354997 | 50.4108904 | -1.1746372 |
| DALYs (Disability-Adjusted Life Years) | Uganda | Both | Age-standardized | Osteoarthritis knee | High body-mass index | Rate | 1990 | 16.6274047 | 51.305515 | -1.2223001 |
| YLDs (Years Lived with Disability) | Uganda | Both | Age-standardized | Osteoarthritis knee | High body-mass index | Rate | 1990 | 16.6274047 | 51.305515 | -1.2223001 |
| DALYs (Disability-Adjusted Life Years) | Pakistan | Both | Age-standardized | Osteoarthritis knee | High body-mass index | Rate | 1990 | 16.5971249 | 47.9298292 | -1.3562493 |
| YLDs (Years Lived with Disability) | Pakistan | Both | Age-standardized | Osteoarthritis knee | High body-mass index | Rate | 1990 | 16.5971249 | 47.9298292 | -1.3562493 |
| DALYs (Disability-Adjusted Life Years) | Angola | Both | Age-standardized | Osteoarthritis knee | High body-mass index | Rate | 1990 | 16.569179 | 49.6148573 | -1.2609802 |
| YLDs (Years Lived with Disability) | Angola | Both | Age-standardized | Osteoarthritis knee | High body-mass index | Rate | 1990 | 16.569179 | 49.6148573 | -1.2609802 |
| DALYs (Disability-Adjusted Life Years) | Malawi | Both | Age-standardized | Osteoarthritis knee | High body-mass index | Rate | 1990 | 16.5057424 | 49.7574937 | -1.3800534 |
| YLDs (Years Lived with Disability) | Malawi | Both | Age-standardized | Osteoarthritis knee | High body-mass index | Rate | 1990 | 16.5057424 | 49.7574937 | -1.3800534 |
| DALYs (Disability-Adjusted Life Years) | Mozambique | Both | Age-standardized | Osteoarthritis knee | High body-mass index | Rate | 1990 | 16.3883914 | 49.8319361 | -1.1678014 |
| YLDs (Years Lived with Disability) | Mozambique | Both | Age-standardized | Osteoarthritis knee | High body-mass index | Rate | 1990 | 16.3883914 | 49.8319361 | -1.1678014 |
| DALYs (Disability-Adjusted Life Years) | Somalia | Both | Age-standardized | Osteoarthritis knee | High body-mass index | Rate | 1990 | 16.3630152 | 47.6863132 | -1.1951912 |
| YLDs (Years Lived with Disability) | Somalia | Both | Age-standardized | Osteoarthritis knee | High body-mass index | Rate | 1990 | 16.3630152 | 47.6863132 | -1.1951912 |
| DALYs (Disability-Adjusted Life Years) | Rwanda | Both | Age-standardized | Osteoarthritis knee | High body-mass index | Rate | 1990 | 15.5759795 | 46.3718587 | -1.1955377 |
| YLDs (Years Lived with Disability) | Rwanda | Both | Age-standardized | Osteoarthritis knee | High body-mass index | Rate | 1990 | 15.5759795 | 46.3718587 | -1.1955377 |
| DALYs (Disability-Adjusted Life Years) | Philippines | Both | Age-standardized | Osteoarthritis knee | High body-mass index | Rate | 1990 | 15.3386828 | 46.0603251 | -1.1136731 |
| YLDs (Years Lived with Disability) | Philippines | Both | Age-standardized | Osteoarthritis knee | High body-mass index | Rate | 1990 | 15.3386828 | 46.0603251 | -1.1136731 |
| DALYs (Disability-Adjusted Life Years) | Burkina Faso | Both | Age-standardized | Osteoarthritis knee | High body-mass index | Rate | 1990 | 15.1108733 | 44.919849 | -1.2999173 |
| YLDs (Years Lived with Disability) | Burkina Faso | Both | Age-standardized | Osteoarthritis knee | High body-mass index | Rate | 1990 | 15.1108733 | 44.919849 | -1.2999173 |
| DALYs (Disability-Adjusted Life Years) | Central African Republic | Both | Age-standardized | Osteoarthritis knee | High body-mass index | Rate | 1990 | 15.0038734 | 47.0963675 | -1.0655383 |
| YLDs (Years Lived with Disability) | Central African Republic | Both | Age-standardized | Osteoarthritis knee | High body-mass index | Rate | 1990 | 15.0038734 | 47.0963675 | -1.0655383 |
| DALYs (Disability-Adjusted Life Years) | Myanmar | Both | Age-standardized | Osteoarthritis knee | High body-mass index | Rate | 1990 | 14.3588918 | 43.0337089 | -1.0524987 |
| YLDs (Years Lived with Disability) | Myanmar | Both | Age-standardized | Osteoarthritis knee | High body-mass index | Rate | 1990 | 14.3588918 | 43.0337089 | -1.0524987 |
| DALYs (Disability-Adjusted Life Years) | Madagascar | Both | Age-standardized | Osteoarthritis knee | High body-mass index | Rate | 1990 | 13.6534198 | 42.4017067 | -0.9117239 |
| YLDs (Years Lived with Disability) | Madagascar | Both | Age-standardized | Osteoarthritis knee | High body-mass index | Rate | 1990 | 13.6534198 | 42.4017067 | -0.9117239 |
| DALYs (Disability-Adjusted Life Years) | Ethiopia | Both | Age-standardized | Osteoarthritis knee | High body-mass index | Rate | 1990 | 13.6020825 | 41.3572959 | -1.019886 |
| YLDs (Years Lived with Disability) | Ethiopia | Both | Age-standardized | Osteoarthritis knee | High body-mass index | Rate | 1990 | 13.6020825 | 41.3572959 | -1.019886 |
| DALYs (Disability-Adjusted Life Years) | Djibouti | Both | Age-standardized | Osteoarthritis knee | High body-mass index | Rate | 1990 | 12.8514861 | 39.0348296 | -1.0172172 |
| YLDs (Years Lived with Disability) | Djibouti | Both | Age-standardized | Osteoarthritis knee | High body-mass index | Rate | 1990 | 12.8514861 | 39.0348296 | -1.0172172 |
| DALYs (Disability-Adjusted Life Years) | Lao People's Democratic Republic | Both | Age-standardized | Osteoarthritis knee | High body-mass index | Rate | 1990 | 12.4881635 | 38.1518535 | -0.8812904 |
| YLDs (Years Lived with Disability) | Lao People's Democratic Republic | Both | Age-standardized | Osteoarthritis knee | High body-mass index | Rate | 1990 | 12.4881635 | 38.1518535 | -0.8812904 |
| DALYs (Disability-Adjusted Life Years) | Burundi | Both | Age-standardized | Osteoarthritis knee | High body-mass index | Rate | 1990 | 12.4129531 | 39.1625966 | -0.8734522 |
| YLDs (Years Lived with Disability) | Burundi | Both | Age-standardized | Osteoarthritis knee | High body-mass index | Rate | 1990 | 12.4129531 | 39.1625966 | -0.8734522 |
| DALYs (Disability-Adjusted Life Years) | South Sudan | Both | Age-standardized | Osteoarthritis knee | High body-mass index | Rate | 1990 | 12.1878746 | 36.7819617 | -0.9621942 |
| YLDs (Years Lived with Disability) | South Sudan | Both | Age-standardized | Osteoarthritis knee | High body-mass index | Rate | 1990 | 12.1878746 | 36.7819617 | -0.9621942 |
| DALYs (Disability-Adjusted Life Years) | Nepal | Both | Age-standardized | Osteoarthritis knee | High body-mass index | Rate | 1990 | 12.1358023 | 36.9699157 | -0.8400426 |
| YLDs (Years Lived with Disability) | Nepal | Both | Age-standardized | Osteoarthritis knee | High body-mass index | Rate | 1990 | 12.1358023 | 36.9699157 | -0.8400426 |
| DALYs (Disability-Adjusted Life Years) | India | Both | Age-standardized | Osteoarthritis knee | High body-mass index | Rate | 1990 | 12.0992959 | 36.008252 | -0.8485698 |
| YLDs (Years Lived with Disability) | India | Both | Age-standardized | Osteoarthritis knee | High body-mass index | Rate | 1990 | 12.0992959 | 36.008252 | -0.8485698 |
| DALYs (Disability-Adjusted Life Years) | Indonesia | Both | Age-standardized | Osteoarthritis knee | High body-mass index | Rate | 1990 | 11.9268869 | 35.7967887 | -0.8915872 |
| YLDs (Years Lived with Disability) | Indonesia | Both | Age-standardized | Osteoarthritis knee | High body-mass index | Rate | 1990 | 11.9268869 | 35.7967887 | -0.8915872 |
| DALYs (Disability-Adjusted Life Years) | Cambodia | Both | Age-standardized | Osteoarthritis knee | High body-mass index | Rate | 1990 | 11.6024304 | 34.8389884 | -0.8984193 |
| YLDs (Years Lived with Disability) | Cambodia | Both | Age-standardized | Osteoarthritis knee | High body-mass index | Rate | 1990 | 11.6024304 | 34.8389884 | -0.8984193 |
| DALYs (Disability-Adjusted Life Years) | Eritrea | Both | Age-standardized | Osteoarthritis knee | High body-mass index | Rate | 1990 | 9.96597083 | 28.7611906 | -0.7825407 |
| YLDs (Years Lived with Disability) | Eritrea | Both | Age-standardized | Osteoarthritis knee | High body-mass index | Rate | 1990 | 9.96597083 | 28.7611906 | -0.7825407 |
| DALYs (Disability-Adjusted Life Years) | Bangladesh | Both | Age-standardized | Osteoarthritis knee | High body-mass index | Rate | 1990 | 9.45585908 | 27.8652144 | -0.7031136 |
| YLDs (Years Lived with Disability) | Bangladesh | Both | Age-standardized | Osteoarthritis knee | High body-mass index | Rate | 1990 | 9.45585908 | 27.8652144 | -0.7031136 |
| DALYs (Disability-Adjusted Life Years) | Viet Nam | Both | Age-standardized | Osteoarthritis knee | High body-mass index | Rate | 1990 | 7.6173424 | 23.7718396 | -0.605167 |
| YLDs (Years Lived with Disability) | Viet Nam | Both | Age-standardized | Osteoarthritis knee | High body-mass index | Rate | 1990 | 7.6173424 | 23.7718396 | -0.605167 |
| DALYs (Disability-Adjusted Life Years) | Timor-Leste | Both | Age-standardized | Osteoarthritis knee | High body-mass index | Rate | 1990 | 7.26348776 | 22.04757 | -0.6348778 |
| YLDs (Years Lived with Disability) | Timor-Leste | Both | Age-standardized | Osteoarthritis knee | High body-mass index | Rate | 1990 | 7.26348776 | 22.04757 | -0.6348778 |

Appendix 7: 2021年204个国家因高体重指数导致的膝骨关节炎的相关伤残调整生命年（DALYs）和相关伤残损失生命年（YLDs）

| measure | location | sex | age | cause | rei | metric | year | val | upper | lower |
| --- | --- | --- | --- | --- | --- | --- | --- | --- | --- | --- |
| DALYs (Disability-Adjusted Life Years) | China | Both | All ages | Osteoarthritis knee | High body-mass index | Number | 2021 | 1111618.73 | 3158801.27 | -100286.08 |
| YLDs (Years Lived with Disability) | China | Both | All ages | Osteoarthritis knee | High body-mass index | Number | 2021 | 1111618.73 | 3158801.27 | -100286.08 |
| DALYs (Disability-Adjusted Life Years) | United States of America | Both | All ages | Osteoarthritis knee | High body-mass index | Number | 2021 | 396242.964 | 1040246.77 | -44029.824 |
| YLDs (Years Lived with Disability) | United States of America | Both | All ages | Osteoarthritis knee | High body-mass index | Number | 2021 | 396242.964 | 1040246.77 | -44029.824 |
| DALYs (Disability-Adjusted Life Years) | India | Both | All ages | Osteoarthritis knee | High body-mass index | Number | 2021 | 326778.158 | 948113.631 | -26038.59 |
| YLDs (Years Lived with Disability) | India | Both | All ages | Osteoarthritis knee | High body-mass index | Number | 2021 | 326778.158 | 948113.631 | -26038.59 |
| DALYs (Disability-Adjusted Life Years) | Brazil | Both | All ages | Osteoarthritis knee | High body-mass index | Number | 2021 | 148771.276 | 397036.278 | -15085.995 |
| YLDs (Years Lived with Disability) | Brazil | Both | All ages | Osteoarthritis knee | High body-mass index | Number | 2021 | 148771.276 | 397036.278 | -15085.995 |
| DALYs (Disability-Adjusted Life Years) | Japan | Both | All ages | Osteoarthritis knee | High body-mass index | Number | 2021 | 129094.015 | 384300.555 | -10071.184 |
| YLDs (Years Lived with Disability) | Japan | Both | All ages | Osteoarthritis knee | High body-mass index | Number | 2021 | 129094.015 | 384300.555 | -10071.184 |
| DALYs (Disability-Adjusted Life Years) | Russian Federation | Both | All ages | Osteoarthritis knee | High body-mass index | Number | 2021 | 113723.815 | 301336.243 | -11684.332 |
| YLDs (Years Lived with Disability) | Russian Federation | Both | All ages | Osteoarthritis knee | High body-mass index | Number | 2021 | 113723.815 | 301336.243 | -11684.332 |
| DALYs (Disability-Adjusted Life Years) | Germany | Both | All ages | Osteoarthritis knee | High body-mass index | Number | 2021 | 92814.2678 | 249543.149 | -9203.4174 |
| YLDs (Years Lived with Disability) | Germany | Both | All ages | Osteoarthritis knee | High body-mass index | Number | 2021 | 92814.2678 | 249543.149 | -9203.4174 |
| DALYs (Disability-Adjusted Life Years) | Mexico | Both | All ages | Osteoarthritis knee | High body-mass index | Number | 2021 | 85784.8551 | 225742.597 | -9603.7373 |
| YLDs (Years Lived with Disability) | Mexico | Both | All ages | Osteoarthritis knee | High body-mass index | Number | 2021 | 85784.8551 | 225742.597 | -9603.7373 |
| DALYs (Disability-Adjusted Life Years) | United Kingdom | Both | All ages | Osteoarthritis knee | High body-mass index | Number | 2021 | 72583.1163 | 193692.488 | -7675.6373 |
| YLDs (Years Lived with Disability) | United Kingdom | Both | All ages | Osteoarthritis knee | High body-mass index | Number | 2021 | 72583.1163 | 193692.488 | -7675.6373 |
| DALYs (Disability-Adjusted Life Years) | Indonesia | Both | All ages | Osteoarthritis knee | High body-mass index | Number | 2021 | 66453.3149 | 190475.651 | -5278.9519 |
| YLDs (Years Lived with Disability) | Indonesia | Both | All ages | Osteoarthritis knee | High body-mass index | Number | 2021 | 66453.3149 | 190475.651 | -5278.9519 |
| DALYs (Disability-Adjusted Life Years) | Italy | Both | All ages | Osteoarthritis knee | High body-mass index | Number | 2021 | 63443.0122 | 174191.142 | -5977.0451 |
| YLDs (Years Lived with Disability) | Italy | Both | All ages | Osteoarthritis knee | High body-mass index | Number | 2021 | 63443.0122 | 174191.142 | -5977.0451 |
| DALYs (Disability-Adjusted Life Years) | France | Both | All ages | Osteoarthritis knee | High body-mass index | Number | 2021 | 61463.9407 | 172816.402 | -5415.4318 |
| YLDs (Years Lived with Disability) | France | Both | All ages | Osteoarthritis knee | High body-mass index | Number | 2021 | 61463.9407 | 172816.402 | -5415.4318 |
| DALYs (Disability-Adjusted Life Years) | T眉rkiye | Both | All ages | Osteoarthritis knee | High body-mass index | Number | 2021 | 60248.7891 | 153945.376 | -7172.8989 |
| YLDs (Years Lived with Disability) | T眉rkiye | Both | All ages | Osteoarthritis knee | High body-mass index | Number | 2021 | 60248.7891 | 153945.376 | -7172.8989 |
| DALYs (Disability-Adjusted Life Years) | Republic of Korea | Both | All ages | Osteoarthritis knee | High body-mass index | Number | 2021 | 51405.8899 | 151774.57 | -4308.2804 |
| YLDs (Years Lived with Disability) | Republic of Korea | Both | All ages | Osteoarthritis knee | High body-mass index | Number | 2021 | 51405.8899 | 151774.57 | -4308.2804 |
| DALYs (Disability-Adjusted Life Years) | Spain | Both | All ages | Osteoarthritis knee | High body-mass index | Number | 2021 | 51099.9852 | 137295.866 | -5308.8022 |
| YLDs (Years Lived with Disability) | Spain | Both | All ages | Osteoarthritis knee | High body-mass index | Number | 2021 | 51099.9852 | 137295.866 | -5308.8022 |
| DALYs (Disability-Adjusted Life Years) | Egypt | Both | All ages | Osteoarthritis knee | High body-mass index | Number | 2021 | 44455.5139 | 113119.207 | -5602.1613 |
| YLDs (Years Lived with Disability) | Egypt | Both | All ages | Osteoarthritis knee | High body-mass index | Number | 2021 | 44455.5139 | 113119.207 | -5602.1613 |
| DALYs (Disability-Adjusted Life Years) | Thailand | Both | All ages | Osteoarthritis knee | High body-mass index | Number | 2021 | 42782.2592 | 122581.902 | -3742.4748 |
| YLDs (Years Lived with Disability) | Thailand | Both | All ages | Osteoarthritis knee | High body-mass index | Number | 2021 | 42782.2592 | 122581.902 | -3742.4748 |
| DALYs (Disability-Adjusted Life Years) | Iran (Islamic Republic of) | Both | All ages | Osteoarthritis knee | High body-mass index | Number | 2021 | 41663.7404 | 109939.341 | -4539.5079 |
| YLDs (Years Lived with Disability) | Iran (Islamic Republic of) | Both | All ages | Osteoarthritis knee | High body-mass index | Number | 2021 | 41663.7404 | 109939.341 | -4539.5079 |
| DALYs (Disability-Adjusted Life Years) | Pakistan | Both | All ages | Osteoarthritis knee | High body-mass index | Number | 2021 | 41641.3969 | 120585.657 | -3637.5312 |
| YLDs (Years Lived with Disability) | Pakistan | Both | All ages | Osteoarthritis knee | High body-mass index | Number | 2021 | 41641.3969 | 120585.657 | -3637.5312 |
| DALYs (Disability-Adjusted Life Years) | Nigeria | Both | All ages | Osteoarthritis knee | High body-mass index | Number | 2021 | 41561.972 | 115766.989 | -3731.8089 |
| YLDs (Years Lived with Disability) | Nigeria | Both | All ages | Osteoarthritis knee | High body-mass index | Number | 2021 | 41561.972 | 115766.989 | -3731.8089 |
| DALYs (Disability-Adjusted Life Years) | Argentina | Both | All ages | Osteoarthritis knee | High body-mass index | Number | 2021 | 35885.0974 | 93589.7756 | -3932.9626 |
| YLDs (Years Lived with Disability) | Argentina | Both | All ages | Osteoarthritis knee | High body-mass index | Number | 2021 | 35885.0974 | 93589.7756 | -3932.9626 |
| DALYs (Disability-Adjusted Life Years) | Ukraine | Both | All ages | Osteoarthritis knee | High body-mass index | Number | 2021 | 35041.0907 | 93634.7367 | -3691.5638 |
| YLDs (Years Lived with Disability) | Ukraine | Both | All ages | Osteoarthritis knee | High body-mass index | Number | 2021 | 35041.0907 | 93634.7367 | -3691.5638 |
| DALYs (Disability-Adjusted Life Years) | Colombia | Both | All ages | Osteoarthritis knee | High body-mass index | Number | 2021 | 32708.5619 | 87878.2053 | -3428.8907 |
| YLDs (Years Lived with Disability) | Colombia | Both | All ages | Osteoarthritis knee | High body-mass index | Number | 2021 | 32708.5619 | 87878.2053 | -3428.8907 |
| DALYs (Disability-Adjusted Life Years) | Bangladesh | Both | All ages | Osteoarthritis knee | High body-mass index | Number | 2021 | 32445.1009 | 94834.4008 | -2550.5115 |
| YLDs (Years Lived with Disability) | Bangladesh | Both | All ages | Osteoarthritis knee | High body-mass index | Number | 2021 | 32445.1009 | 94834.4008 | -2550.5115 |
| DALYs (Disability-Adjusted Life Years) | Poland | Both | All ages | Osteoarthritis knee | High body-mass index | Number | 2021 | 29299.8351 | 79186.2696 | -3081.4642 |
| YLDs (Years Lived with Disability) | Poland | Both | All ages | Osteoarthritis knee | High body-mass index | Number | 2021 | 29299.8351 | 79186.2696 | -3081.4642 |
| DALYs (Disability-Adjusted Life Years) | Australia | Both | All ages | Osteoarthritis knee | High body-mass index | Number | 2021 | 28569.3882 | 77094.2215 | -2859.1409 |
| YLDs (Years Lived with Disability) | Australia | Both | All ages | Osteoarthritis knee | High body-mass index | Number | 2021 | 28569.3882 | 77094.2215 | -2859.1409 |
| DALYs (Disability-Adjusted Life Years) | South Africa | Both | All ages | Osteoarthritis knee | High body-mass index | Number | 2021 | 27082.9546 | 71515.1921 | -2849.0521 |
| YLDs (Years Lived with Disability) | South Africa | Both | All ages | Osteoarthritis knee | High body-mass index | Number | 2021 | 27082.9546 | 71515.1921 | -2849.0521 |
| DALYs (Disability-Adjusted Life Years) | Canada | Both | All ages | Osteoarthritis knee | High body-mass index | Number | 2021 | 24722.0736 | 66610.175 | -2339.1094 |
| YLDs (Years Lived with Disability) | Canada | Both | All ages | Osteoarthritis knee | High body-mass index | Number | 2021 | 24722.0736 | 66610.175 | -2339.1094 |
| DALYs (Disability-Adjusted Life Years) | Taiwan (Province of China) | Both | All ages | Osteoarthritis knee | High body-mass index | Number | 2021 | 24466.727 | 70322.4146 | -2408.5977 |
| YLDs (Years Lived with Disability) | Taiwan (Province of China) | Both | All ages | Osteoarthritis knee | High body-mass index | Number | 2021 | 24466.727 | 70322.4146 | -2408.5977 |
| DALYs (Disability-Adjusted Life Years) | Philippines | Both | All ages | Osteoarthritis knee | High body-mass index | Number | 2021 | 22909.5883 | 66139.8359 | -1743.9246 |
| YLDs (Years Lived with Disability) | Philippines | Both | All ages | Osteoarthritis knee | High body-mass index | Number | 2021 | 22909.5883 | 66139.8359 | -1743.9246 |
| DALYs (Disability-Adjusted Life Years) | Peru | Both | All ages | Osteoarthritis knee | High body-mass index | Number | 2021 | 20168.1786 | 53674.5594 | -2210.5516 |
| YLDs (Years Lived with Disability) | Peru | Both | All ages | Osteoarthritis knee | High body-mass index | Number | 2021 | 20168.1786 | 53674.5594 | -2210.5516 |
| DALYs (Disability-Adjusted Life Years) | Venezuela (Bolivarian Republic of) | Both | All ages | Osteoarthritis knee | High body-mass index | Number | 2021 | 20045.2967 | 52716.7634 | -2148.5709 |
| YLDs (Years Lived with Disability) | Venezuela (Bolivarian Republic of) | Both | All ages | Osteoarthritis knee | High body-mass index | Number | 2021 | 20045.2967 | 52716.7634 | -2148.5709 |
| DALYs (Disability-Adjusted Life Years) | Algeria | Both | All ages | Osteoarthritis knee | High body-mass index | Number | 2021 | 19949.7527 | 52275.7754 | -2192.9025 |
| YLDs (Years Lived with Disability) | Algeria | Both | All ages | Osteoarthritis knee | High body-mass index | Number | 2021 | 19949.7527 | 52275.7754 | -2192.9025 |
| DALYs (Disability-Adjusted Life Years) | Viet Nam | Both | All ages | Osteoarthritis knee | High body-mass index | Number | 2021 | 17868.7279 | 53725.2843 | -1266.2074 |
| YLDs (Years Lived with Disability) | Viet Nam | Both | All ages | Osteoarthritis knee | High body-mass index | Number | 2021 | 17868.7279 | 53725.2843 | -1266.2074 |
| DALYs (Disability-Adjusted Life Years) | Chile | Both | All ages | Osteoarthritis knee | High body-mass index | Number | 2021 | 17584.1102 | 45064.5099 | -1966.2512 |
| YLDs (Years Lived with Disability) | Chile | Both | All ages | Osteoarthritis knee | High body-mass index | Number | 2021 | 17584.1102 | 45064.5099 | -1966.2512 |
| DALYs (Disability-Adjusted Life Years) | Netherlands | Both | All ages | Osteoarthritis knee | High body-mass index | Number | 2021 | 17231.0162 | 46722.0772 | -1747.6801 |
| YLDs (Years Lived with Disability) | Netherlands | Both | All ages | Osteoarthritis knee | High body-mass index | Number | 2021 | 17231.0162 | 46722.0772 | -1747.6801 |
| DALYs (Disability-Adjusted Life Years) | Morocco | Both | All ages | Osteoarthritis knee | High body-mass index | Number | 2021 | 17218.0365 | 45048.1028 | -1839.7851 |
| YLDs (Years Lived with Disability) | Morocco | Both | All ages | Osteoarthritis knee | High body-mass index | Number | 2021 | 17218.0365 | 45048.1028 | -1839.7851 |
| DALYs (Disability-Adjusted Life Years) | Saudi Arabia | Both | All ages | Osteoarthritis knee | High body-mass index | Number | 2021 | 16344.8546 | 40687.608 | -1997.5554 |
| YLDs (Years Lived with Disability) | Saudi Arabia | Both | All ages | Osteoarthritis knee | High body-mass index | Number | 2021 | 16344.8546 | 40687.608 | -1997.5554 |
| DALYs (Disability-Adjusted Life Years) | Romania | Both | All ages | Osteoarthritis knee | High body-mass index | Number | 2021 | 15179.9744 | 41140.059 | -1487.25 |
| YLDs (Years Lived with Disability) | Romania | Both | All ages | Osteoarthritis knee | High body-mass index | Number | 2021 | 15179.9744 | 41140.059 | -1487.25 |
| DALYs (Disability-Adjusted Life Years) | Iraq | Both | All ages | Osteoarthritis knee | High body-mass index | Number | 2021 | 14422.3183 | 37318.3688 | -1589.863 |
| YLDs (Years Lived with Disability) | Iraq | Both | All ages | Osteoarthritis knee | High body-mass index | Number | 2021 | 14422.3183 | 37318.3688 | -1589.863 |
| DALYs (Disability-Adjusted Life Years) | Democratic Republic of the Congo | Both | All ages | Osteoarthritis knee | High body-mass index | Number | 2021 | 12533.0635 | 37451.1149 | -956.40227 |
| YLDs (Years Lived with Disability) | Democratic Republic of the Congo | Both | All ages | Osteoarthritis knee | High body-mass index | Number | 2021 | 12533.0635 | 37451.1149 | -956.40227 |
| DALYs (Disability-Adjusted Life Years) | Myanmar | Both | All ages | Osteoarthritis knee | High body-mass index | Number | 2021 | 12286.9269 | 35030.4562 | -989.86945 |
| YLDs (Years Lived with Disability) | Myanmar | Both | All ages | Osteoarthritis knee | High body-mass index | Number | 2021 | 12286.9269 | 35030.4562 | -989.86945 |
| DALYs (Disability-Adjusted Life Years) | Portugal | Both | All ages | Osteoarthritis knee | High body-mass index | Number | 2021 | 11721.7907 | 31252.5619 | -1201.959 |
| YLDs (Years Lived with Disability) | Portugal | Both | All ages | Osteoarthritis knee | High body-mass index | Number | 2021 | 11721.7907 | 31252.5619 | -1201.959 |
| DALYs (Disability-Adjusted Life Years) | Malaysia | Both | All ages | Osteoarthritis knee | High body-mass index | Number | 2021 | 11641.7365 | 31405.2549 | -1080.323 |
| YLDs (Years Lived with Disability) | Malaysia | Both | All ages | Osteoarthritis knee | High body-mass index | Number | 2021 | 11641.7365 | 31405.2549 | -1080.323 |
| DALYs (Disability-Adjusted Life Years) | Greece | Both | All ages | Osteoarthritis knee | High body-mass index | Number | 2021 | 11617.0813 | 31758.1684 | -1160.0823 |
| YLDs (Years Lived with Disability) | Greece | Both | All ages | Osteoarthritis knee | High body-mass index | Number | 2021 | 11617.0813 | 31758.1684 | -1160.0823 |
| DALYs (Disability-Adjusted Life Years) | Ecuador | Both | All ages | Osteoarthritis knee | High body-mass index | Number | 2021 | 11334.8832 | 30291.6407 | -1177.8657 |
| YLDs (Years Lived with Disability) | Ecuador | Both | All ages | Osteoarthritis knee | High body-mass index | Number | 2021 | 11334.8832 | 30291.6407 | -1177.8657 |
| DALYs (Disability-Adjusted Life Years) | Cuba | Both | All ages | Osteoarthritis knee | High body-mass index | Number | 2021 | 11010.4032 | 29897.7182 | -1091.3011 |
| YLDs (Years Lived with Disability) | Cuba | Both | All ages | Osteoarthritis knee | High body-mass index | Number | 2021 | 11010.4032 | 29897.7182 | -1091.3011 |
| DALYs (Disability-Adjusted Life Years) | Democratic People's Republic of Korea | Both | All ages | Osteoarthritis knee | High body-mass index | Number | 2021 | 11000.6263 | 31818.1295 | -936.91796 |
| YLDs (Years Lived with Disability) | Democratic People's Republic of Korea | Both | All ages | Osteoarthritis knee | High body-mass index | Number | 2021 | 11000.6263 | 31818.1295 | -936.91796 |
| DALYs (Disability-Adjusted Life Years) | Belgium | Both | All ages | Osteoarthritis knee | High body-mass index | Number | 2021 | 10640.6776 | 29356.0394 | -1011.4708 |
| YLDs (Years Lived with Disability) | Belgium | Both | All ages | Osteoarthritis knee | High body-mass index | Number | 2021 | 10640.6776 | 29356.0394 | -1011.4708 |
| DALYs (Disability-Adjusted Life Years) | Sudan | Both | All ages | Osteoarthritis knee | High body-mass index | Number | 2021 | 10548.1308 | 27838.4857 | -1120.6274 |
| YLDs (Years Lived with Disability) | Sudan | Both | All ages | Osteoarthritis knee | High body-mass index | Number | 2021 | 10548.1308 | 27838.4857 | -1120.6274 |
| DALYs (Disability-Adjusted Life Years) | Uzbekistan | Both | All ages | Osteoarthritis knee | High body-mass index | Number | 2021 | 10156.142 | 26588.022 | -1141.1271 |
| YLDs (Years Lived with Disability) | Uzbekistan | Both | All ages | Osteoarthritis knee | High body-mass index | Number | 2021 | 10156.142 | 26588.022 | -1141.1271 |
| DALYs (Disability-Adjusted Life Years) | United Republic of Tanzania | Both | All ages | Osteoarthritis knee | High body-mass index | Number | 2021 | 10050.4355 | 27577.4125 | -827.63308 |
| YLDs (Years Lived with Disability) | United Republic of Tanzania | Both | All ages | Osteoarthritis knee | High body-mass index | Number | 2021 | 10050.4355 | 27577.4125 | -827.63308 |
| DALYs (Disability-Adjusted Life Years) | Czechia | Both | All ages | Osteoarthritis knee | High body-mass index | Number | 2021 | 9057.811 | 24310.8708 | -1000.4783 |
| YLDs (Years Lived with Disability) | Czechia | Both | All ages | Osteoarthritis knee | High body-mass index | Number | 2021 | 9057.811 | 24310.8708 | -1000.4783 |
| DALYs (Disability-Adjusted Life Years) | Ethiopia | Both | All ages | Osteoarthritis knee | High body-mass index | Number | 2021 | 9025.38264 | 26688.3882 | -706.19397 |
| YLDs (Years Lived with Disability) | Ethiopia | Both | All ages | Osteoarthritis knee | High body-mass index | Number | 2021 | 9025.38264 | 26688.3882 | -706.19397 |
| DALYs (Disability-Adjusted Life Years) | Syrian Arab Republic | Both | All ages | Osteoarthritis knee | High body-mass index | Number | 2021 | 8775.79541 | 22401.2761 | -1107.7727 |
| YLDs (Years Lived with Disability) | Syrian Arab Republic | Both | All ages | Osteoarthritis knee | High body-mass index | Number | 2021 | 8775.79541 | 22401.2761 | -1107.7727 |
| DALYs (Disability-Adjusted Life Years) | Austria | Both | All ages | Osteoarthritis knee | High body-mass index | Number | 2021 | 8555.70074 | 23998.2934 | -777.86421 |
| YLDs (Years Lived with Disability) | Austria | Both | All ages | Osteoarthritis knee | High body-mass index | Number | 2021 | 8555.70074 | 23998.2934 | -777.86421 |
| DALYs (Disability-Adjusted Life Years) | Hungary | Both | All ages | Osteoarthritis knee | High body-mass index | Number | 2021 | 8554.17756 | 23327.0276 | -858.92426 |
| YLDs (Years Lived with Disability) | Hungary | Both | All ages | Osteoarthritis knee | High body-mass index | Number | 2021 | 8554.17756 | 23327.0276 | -858.92426 |
| DALYs (Disability-Adjusted Life Years) | Kenya | Both | All ages | Osteoarthritis knee | High body-mass index | Number | 2021 | 8278.56846 | 23639.6654 | -695.52351 |
| YLDs (Years Lived with Disability) | Kenya | Both | All ages | Osteoarthritis knee | High body-mass index | Number | 2021 | 8278.56846 | 23639.6654 | -695.52351 |
| DALYs (Disability-Adjusted Life Years) | Sri Lanka | Both | All ages | Osteoarthritis knee | High body-mass index | Number | 2021 | 8104.37024 | 23703.4774 | -610.77915 |
| YLDs (Years Lived with Disability) | Sri Lanka | Both | All ages | Osteoarthritis knee | High body-mass index | Number | 2021 | 8104.37024 | 23703.4774 | -610.77915 |
| DALYs (Disability-Adjusted Life Years) | Ghana | Both | All ages | Osteoarthritis knee | High body-mass index | Number | 2021 | 7787.23649 | 21490.3574 | -656.7804 |
| YLDs (Years Lived with Disability) | Ghana | Both | All ages | Osteoarthritis knee | High body-mass index | Number | 2021 | 7787.23649 | 21490.3574 | -656.7804 |
| DALYs (Disability-Adjusted Life Years) | Sweden | Both | All ages | Osteoarthritis knee | High body-mass index | Number | 2021 | 7728.80593 | 21580.0212 | -756.55927 |
| YLDs (Years Lived with Disability) | Sweden | Both | All ages | Osteoarthritis knee | High body-mass index | Number | 2021 | 7728.80593 | 21580.0212 | -756.55927 |
| DALYs (Disability-Adjusted Life Years) | Belarus | Both | All ages | Osteoarthritis knee | High body-mass index | Number | 2021 | 7642.36063 | 20182.4975 | -846.54138 |
| YLDs (Years Lived with Disability) | Belarus | Both | All ages | Osteoarthritis knee | High body-mass index | Number | 2021 | 7642.36063 | 20182.4975 | -846.54138 |
| DALYs (Disability-Adjusted Life Years) | Switzerland | Both | All ages | Osteoarthritis knee | High body-mass index | Number | 2021 | 7575.7439 | 21272.1247 | -649.80129 |
| YLDs (Years Lived with Disability) | Switzerland | Both | All ages | Osteoarthritis knee | High body-mass index | Number | 2021 | 7575.7439 | 21272.1247 | -649.80129 |
| DALYs (Disability-Adjusted Life Years) | Cameroon | Both | All ages | Osteoarthritis knee | High body-mass index | Number | 2021 | 7450.84359 | 19922.0645 | -801.49789 |
| YLDs (Years Lived with Disability) | Cameroon | Both | All ages | Osteoarthritis knee | High body-mass index | Number | 2021 | 7450.84359 | 19922.0645 | -801.49789 |
| DALYs (Disability-Adjusted Life Years) | Tunisia | Both | All ages | Osteoarthritis knee | High body-mass index | Number | 2021 | 7157.47946 | 18782.3276 | -818.60145 |
| YLDs (Years Lived with Disability) | Tunisia | Both | All ages | Osteoarthritis knee | High body-mass index | Number | 2021 | 7157.47946 | 18782.3276 | -818.60145 |
| DALYs (Disability-Adjusted Life Years) | Serbia | Both | All ages | Osteoarthritis knee | High body-mass index | Number | 2021 | 7128.92782 | 18888.33 | -765.86714 |
| YLDs (Years Lived with Disability) | Serbia | Both | All ages | Osteoarthritis knee | High body-mass index | Number | 2021 | 7128.92782 | 18888.33 | -765.86714 |
| DALYs (Disability-Adjusted Life Years) | Kazakhstan | Both | All ages | Osteoarthritis knee | High body-mass index | Number | 2021 | 7093.87045 | 18956.5262 | -724.06359 |
| YLDs (Years Lived with Disability) | Kazakhstan | Both | All ages | Osteoarthritis knee | High body-mass index | Number | 2021 | 7093.87045 | 18956.5262 | -724.06359 |
| DALYs (Disability-Adjusted Life Years) | Guatemala | Both | All ages | Osteoarthritis knee | High body-mass index | Number | 2021 | 6486.00974 | 17278.3544 | -650.37934 |
| YLDs (Years Lived with Disability) | Guatemala | Both | All ages | Osteoarthritis knee | High body-mass index | Number | 2021 | 6486.00974 | 17278.3544 | -650.37934 |
| DALYs (Disability-Adjusted Life Years) | Israel | Both | All ages | Osteoarthritis knee | High body-mass index | Number | 2021 | 6334.04453 | 17402.4071 | -607.51176 |
| YLDs (Years Lived with Disability) | Israel | Both | All ages | Osteoarthritis knee | High body-mass index | Number | 2021 | 6334.04453 | 17402.4071 | -607.51176 |
| DALYs (Disability-Adjusted Life Years) | Nepal | Both | All ages | Osteoarthritis knee | High body-mass index | Number | 2021 | 6238.59344 | 17801.0703 | -485.34315 |
| YLDs (Years Lived with Disability) | Nepal | Both | All ages | Osteoarthritis knee | High body-mass index | Number | 2021 | 6238.59344 | 17801.0703 | -485.34315 |
| DALYs (Disability-Adjusted Life Years) | Finland | Both | All ages | Osteoarthritis knee | High body-mass index | Number | 2021 | 5982.70947 | 16138.2106 | -610.67949 |
| YLDs (Years Lived with Disability) | Finland | Both | All ages | Osteoarthritis knee | High body-mass index | Number | 2021 | 5982.70947 | 16138.2106 | -610.67949 |
| DALYs (Disability-Adjusted Life Years) | Bulgaria | Both | All ages | Osteoarthritis knee | High body-mass index | Number | 2021 | 5791.96045 | 15940.0763 | -576.49089 |
| YLDs (Years Lived with Disability) | Bulgaria | Both | All ages | Osteoarthritis knee | High body-mass index | Number | 2021 | 5791.96045 | 15940.0763 | -576.49089 |
| DALYs (Disability-Adjusted Life Years) | Yemen | Both | All ages | Osteoarthritis knee | High body-mass index | Number | 2021 | 5574.53392 | 15765.4488 | -471.4893 |
| YLDs (Years Lived with Disability) | Yemen | Both | All ages | Osteoarthritis knee | High body-mass index | Number | 2021 | 5574.53392 | 15765.4488 | -471.4893 |
| DALYs (Disability-Adjusted Life Years) | Dominican Republic | Both | All ages | Osteoarthritis knee | High body-mass index | Number | 2021 | 5512.92032 | 15156.5036 | -550.08297 |
| YLDs (Years Lived with Disability) | Dominican Republic | Both | All ages | Osteoarthritis knee | High body-mass index | Number | 2021 | 5512.92032 | 15156.5036 | -550.08297 |
| DALYs (Disability-Adjusted Life Years) | Jordan | Both | All ages | Osteoarthritis knee | High body-mass index | Number | 2021 | 5452.03084 | 13841.334 | -692.47488 |
| YLDs (Years Lived with Disability) | Jordan | Both | All ages | Osteoarthritis knee | High body-mass index | Number | 2021 | 5452.03084 | 13841.334 | -692.47488 |
| DALYs (Disability-Adjusted Life Years) | Bolivia (Plurinational State of) | Both | All ages | Osteoarthritis knee | High body-mass index | Number | 2021 | 5426.19268 | 14575.5375 | -555.52017 |
| YLDs (Years Lived with Disability) | Bolivia (Plurinational State of) | Both | All ages | Osteoarthritis knee | High body-mass index | Number | 2021 | 5426.19268 | 14575.5375 | -555.52017 |
| DALYs (Disability-Adjusted Life Years) | Singapore | Both | All ages | Osteoarthritis knee | High body-mass index | Number | 2021 | 5415.20998 | 15418.6561 | -435.04896 |
| YLDs (Years Lived with Disability) | Singapore | Both | All ages | Osteoarthritis knee | High body-mass index | Number | 2021 | 5415.20998 | 15418.6561 | -435.04896 |
| DALYs (Disability-Adjusted Life Years) | C么te d'Ivoire | Both | All ages | Osteoarthritis knee | High body-mass index | Number | 2021 | 5238.36576 | 14521.5901 | -452.52761 |
| YLDs (Years Lived with Disability) | C么te d'Ivoire | Both | All ages | Osteoarthritis knee | High body-mass index | Number | 2021 | 5238.36576 | 14521.5901 | -452.52761 |
| DALYs (Disability-Adjusted Life Years) | United Arab Emirates | Both | All ages | Osteoarthritis knee | High body-mass index | Number | 2021 | 5181.80996 | 12690.1998 | -646.89197 |
| YLDs (Years Lived with Disability) | United Arab Emirates | Both | All ages | Osteoarthritis knee | High body-mass index | Number | 2021 | 5181.80996 | 12690.1998 | -646.89197 |
| DALYs (Disability-Adjusted Life Years) | Denmark | Both | All ages | Osteoarthritis knee | High body-mass index | Number | 2021 | 5143.13308 | 14114.5388 | -492.76487 |
| YLDs (Years Lived with Disability) | Denmark | Both | All ages | Osteoarthritis knee | High body-mass index | Number | 2021 | 5143.13308 | 14114.5388 | -492.76487 |
| DALYs (Disability-Adjusted Life Years) | New Zealand | Both | All ages | Osteoarthritis knee | High body-mass index | Number | 2021 | 5059.35091 | 13717.658 | -554.78832 |
| YLDs (Years Lived with Disability) | New Zealand | Both | All ages | Osteoarthritis knee | High body-mass index | Number | 2021 | 5059.35091 | 13717.658 | -554.78832 |
| DALYs (Disability-Adjusted Life Years) | Puerto Rico | Both | All ages | Osteoarthritis knee | High body-mass index | Number | 2021 | 4593.38881 | 12316.003 | -500.76651 |
| YLDs (Years Lived with Disability) | Puerto Rico | Both | All ages | Osteoarthritis knee | High body-mass index | Number | 2021 | 4593.38881 | 12316.003 | -500.76651 |
| DALYs (Disability-Adjusted Life Years) | Uganda | Both | All ages | Osteoarthritis knee | High body-mass index | Number | 2021 | 4512.5852 | 12959.9303 | -360.31953 |
| YLDs (Years Lived with Disability) | Uganda | Both | All ages | Osteoarthritis knee | High body-mass index | Number | 2021 | 4512.5852 | 12959.9303 | -360.31953 |
| DALYs (Disability-Adjusted Life Years) | Norway | Both | All ages | Osteoarthritis knee | High body-mass index | Number | 2021 | 4332.07668 | 12171.5265 | -398.29581 |
| YLDs (Years Lived with Disability) | Norway | Both | All ages | Osteoarthritis knee | High body-mass index | Number | 2021 | 4332.07668 | 12171.5265 | -398.29581 |
| DALYs (Disability-Adjusted Life Years) | Slovakia | Both | All ages | Osteoarthritis knee | High body-mass index | Number | 2021 | 4290.24491 | 11482.0502 | -435.08732 |
| YLDs (Years Lived with Disability) | Slovakia | Both | All ages | Osteoarthritis knee | High body-mass index | Number | 2021 | 4290.24491 | 11482.0502 | -435.08732 |
| DALYs (Disability-Adjusted Life Years) | Azerbaijan | Both | All ages | Osteoarthritis knee | High body-mass index | Number | 2021 | 4196.77997 | 11004.8245 | -448.6389 |
| YLDs (Years Lived with Disability) | Azerbaijan | Both | All ages | Osteoarthritis knee | High body-mass index | Number | 2021 | 4196.77997 | 11004.8245 | -448.6389 |
| DALYs (Disability-Adjusted Life Years) | Afghanistan | Both | All ages | Osteoarthritis knee | High body-mass index | Number | 2021 | 4186.61621 | 11451.8497 | -372.58475 |
| YLDs (Years Lived with Disability) | Afghanistan | Both | All ages | Osteoarthritis knee | High body-mass index | Number | 2021 | 4186.61621 | 11451.8497 | -372.58475 |
| DALYs (Disability-Adjusted Life Years) | Ireland | Both | All ages | Osteoarthritis knee | High body-mass index | Number | 2021 | 4134.99827 | 10982.9529 | -420.59165 |
| YLDs (Years Lived with Disability) | Ireland | Both | All ages | Osteoarthritis knee | High body-mass index | Number | 2021 | 4134.99827 | 10982.9529 | -420.59165 |
| DALYs (Disability-Adjusted Life Years) | Angola | Both | All ages | Osteoarthritis knee | High body-mass index | Number | 2021 | 4083.49261 | 11825.5114 | -324.42013 |
| YLDs (Years Lived with Disability) | Angola | Both | All ages | Osteoarthritis knee | High body-mass index | Number | 2021 | 4083.49261 | 11825.5114 | -324.42013 |
| DALYs (Disability-Adjusted Life Years) | El Salvador | Both | All ages | Osteoarthritis knee | High body-mass index | Number | 2021 | 3958.38069 | 10299.9319 | -430.25421 |
| YLDs (Years Lived with Disability) | El Salvador | Both | All ages | Osteoarthritis knee | High body-mass index | Number | 2021 | 3958.38069 | 10299.9319 | -430.25421 |
| DALYs (Disability-Adjusted Life Years) | Croatia | Both | All ages | Osteoarthritis knee | High body-mass index | Number | 2021 | 3703.46174 | 10206.4914 | -391.83366 |
| YLDs (Years Lived with Disability) | Croatia | Both | All ages | Osteoarthritis knee | High body-mass index | Number | 2021 | 3703.46174 | 10206.4914 | -391.83366 |
| DALYs (Disability-Adjusted Life Years) | Honduras | Both | All ages | Osteoarthritis knee | High body-mass index | Number | 2021 | 3695.6333 | 9696.46789 | -405.65392 |
| YLDs (Years Lived with Disability) | Honduras | Both | All ages | Osteoarthritis knee | High body-mass index | Number | 2021 | 3695.6333 | 9696.46789 | -405.65392 |
| DALYs (Disability-Adjusted Life Years) | Libya | Both | All ages | Osteoarthritis knee | High body-mass index | Number | 2021 | 3624.08445 | 9181.81998 | -455.71988 |
| YLDs (Years Lived with Disability) | Libya | Both | All ages | Osteoarthritis knee | High body-mass index | Number | 2021 | 3624.08445 | 9181.81998 | -455.71988 |
| DALYs (Disability-Adjusted Life Years) | Paraguay | Both | All ages | Osteoarthritis knee | High body-mass index | Number | 2021 | 3526.8271 | 9469.61724 | -393.66299 |
| YLDs (Years Lived with Disability) | Paraguay | Both | All ages | Osteoarthritis knee | High body-mass index | Number | 2021 | 3526.8271 | 9469.61724 | -393.66299 |
| DALYs (Disability-Adjusted Life Years) | Mozambique | Both | All ages | Osteoarthritis knee | High body-mass index | Number | 2021 | 3445.46552 | 9844.68367 | -256.23919 |
| YLDs (Years Lived with Disability) | Mozambique | Both | All ages | Osteoarthritis knee | High body-mass index | Number | 2021 | 3445.46552 | 9844.68367 | -256.23919 |
| DALYs (Disability-Adjusted Life Years) | Costa Rica | Both | All ages | Osteoarthritis knee | High body-mass index | Number | 2021 | 3435.59854 | 9193.64048 | -374.49027 |
| YLDs (Years Lived with Disability) | Costa Rica | Both | All ages | Osteoarthritis knee | High body-mass index | Number | 2021 | 3435.59854 | 9193.64048 | -374.49027 |
| DALYs (Disability-Adjusted Life Years) | Lebanon | Both | All ages | Osteoarthritis knee | High body-mass index | Number | 2021 | 3323.74409 | 8572.54769 | -385.02659 |
| YLDs (Years Lived with Disability) | Lebanon | Both | All ages | Osteoarthritis knee | High body-mass index | Number | 2021 | 3323.74409 | 8572.54769 | -385.02659 |
| DALYs (Disability-Adjusted Life Years) | Uruguay | Both | All ages | Osteoarthritis knee | High body-mass index | Number | 2021 | 3201.47099 | 8544.07029 | -316.57691 |
| YLDs (Years Lived with Disability) | Uruguay | Both | All ages | Osteoarthritis knee | High body-mass index | Number | 2021 | 3201.47099 | 8544.07029 | -316.57691 |
| DALYs (Disability-Adjusted Life Years) | Nicaragua | Both | All ages | Osteoarthritis knee | High body-mass index | Number | 2021 | 3189.29798 | 8429.03507 | -367.49348 |
| YLDs (Years Lived with Disability) | Nicaragua | Both | All ages | Osteoarthritis knee | High body-mass index | Number | 2021 | 3189.29798 | 8429.03507 | -367.49348 |
| DALYs (Disability-Adjusted Life Years) | Senegal | Both | All ages | Osteoarthritis knee | High body-mass index | Number | 2021 | 3136.95266 | 8786.12629 | -269.63533 |
| YLDs (Years Lived with Disability) | Senegal | Both | All ages | Osteoarthritis knee | High body-mass index | Number | 2021 | 3136.95266 | 8786.12629 | -269.63533 |
| DALYs (Disability-Adjusted Life Years) | Zimbabwe | Both | All ages | Osteoarthritis knee | High body-mass index | Number | 2021 | 2997.43169 | 8016.71744 | -261.12231 |
| YLDs (Years Lived with Disability) | Zimbabwe | Both | All ages | Osteoarthritis knee | High body-mass index | Number | 2021 | 2997.43169 | 8016.71744 | -261.12231 |
| DALYs (Disability-Adjusted Life Years) | Republic of Moldova | Both | All ages | Osteoarthritis knee | High body-mass index | Number | 2021 | 2935.42936 | 7874.9954 | -318.30896 |
| YLDs (Years Lived with Disability) | Republic of Moldova | Both | All ages | Osteoarthritis knee | High body-mass index | Number | 2021 | 2935.42936 | 7874.9954 | -318.30896 |
| DALYs (Disability-Adjusted Life Years) | Madagascar | Both | All ages | Osteoarthritis knee | High body-mass index | Number | 2021 | 2875.68031 | 8212.06421 | -212.58191 |
| YLDs (Years Lived with Disability) | Madagascar | Both | All ages | Osteoarthritis knee | High body-mass index | Number | 2021 | 2875.68031 | 8212.06421 | -212.58191 |
| DALYs (Disability-Adjusted Life Years) | Panama | Both | All ages | Osteoarthritis knee | High body-mass index | Number | 2021 | 2771.87412 | 7443.12268 | -293.95268 |
| YLDs (Years Lived with Disability) | Panama | Both | All ages | Osteoarthritis knee | High body-mass index | Number | 2021 | 2771.87412 | 7443.12268 | -293.95268 |
| DALYs (Disability-Adjusted Life Years) | Zambia | Both | All ages | Osteoarthritis knee | High body-mass index | Number | 2021 | 2679.75949 | 7443.16034 | -238.04883 |
| YLDs (Years Lived with Disability) | Zambia | Both | All ages | Osteoarthritis knee | High body-mass index | Number | 2021 | 2679.75949 | 7443.16034 | -238.04883 |
| DALYs (Disability-Adjusted Life Years) | Mali | Both | All ages | Osteoarthritis knee | High body-mass index | Number | 2021 | 2667.73441 | 7727.4433 | -206.8382 |
| YLDs (Years Lived with Disability) | Mali | Both | All ages | Osteoarthritis knee | High body-mass index | Number | 2021 | 2667.73441 | 7727.4433 | -206.8382 |
| DALYs (Disability-Adjusted Life Years) | Kuwait | Both | All ages | Osteoarthritis knee | High body-mass index | Number | 2021 | 2605.39271 | 6308.85804 | -344.71759 |
| YLDs (Years Lived with Disability) | Kuwait | Both | All ages | Osteoarthritis knee | High body-mass index | Number | 2021 | 2605.39271 | 6308.85804 | -344.71759 |
| DALYs (Disability-Adjusted Life Years) | Lithuania | Both | All ages | Osteoarthritis knee | High body-mass index | Number | 2021 | 2552.80684 | 6768.86573 | -263.72023 |
| YLDs (Years Lived with Disability) | Lithuania | Both | All ages | Osteoarthritis knee | High body-mass index | Number | 2021 | 2552.80684 | 6768.86573 | -263.72023 |
| DALYs (Disability-Adjusted Life Years) | Cambodia | Both | All ages | Osteoarthritis knee | High body-mass index | Number | 2021 | 2537.54232 | 7576.2738 | -193.51624 |
| YLDs (Years Lived with Disability) | Cambodia | Both | All ages | Osteoarthritis knee | High body-mass index | Number | 2021 | 2537.54232 | 7576.2738 | -193.51624 |
| DALYs (Disability-Adjusted Life Years) | Bosnia and Herzegovina | Both | All ages | Osteoarthritis knee | High body-mass index | Number | 2021 | 2487.18891 | 6695.42421 | -265.53557 |
| YLDs (Years Lived with Disability) | Bosnia and Herzegovina | Both | All ages | Osteoarthritis knee | High body-mass index | Number | 2021 | 2487.18891 | 6695.42421 | -265.53557 |
| DALYs (Disability-Adjusted Life Years) | Haiti | Both | All ages | Osteoarthritis knee | High body-mass index | Number | 2021 | 2419.38072 | 6866.94514 | -195.68634 |
| YLDs (Years Lived with Disability) | Haiti | Both | All ages | Osteoarthritis knee | High body-mass index | Number | 2021 | 2419.38072 | 6866.94514 | -195.68634 |
| DALYs (Disability-Adjusted Life Years) | Papua New Guinea | Both | All ages | Osteoarthritis knee | High body-mass index | Number | 2021 | 2384.65203 | 6552.158 | -232.24922 |
| YLDs (Years Lived with Disability) | Papua New Guinea | Both | All ages | Osteoarthritis knee | High body-mass index | Number | 2021 | 2384.65203 | 6552.158 | -232.24922 |
| DALYs (Disability-Adjusted Life Years) | Niger | Both | All ages | Osteoarthritis knee | High body-mass index | Number | 2021 | 2373.15788 | 6923.48406 | -178.43193 |
| YLDs (Years Lived with Disability) | Niger | Both | All ages | Osteoarthritis knee | High body-mass index | Number | 2021 | 2373.15788 | 6923.48406 | -178.43193 |
| DALYs (Disability-Adjusted Life Years) | Benin | Both | All ages | Osteoarthritis knee | High body-mass index | Number | 2021 | 2332.17235 | 6602.35842 | -195.3359 |
| YLDs (Years Lived with Disability) | Benin | Both | All ages | Osteoarthritis knee | High body-mass index | Number | 2021 | 2332.17235 | 6602.35842 | -195.3359 |
| DALYs (Disability-Adjusted Life Years) | Burkina Faso | Both | All ages | Osteoarthritis knee | High body-mass index | Number | 2021 | 2261.67523 | 6670.56311 | -184.48831 |
| YLDs (Years Lived with Disability) | Burkina Faso | Both | All ages | Osteoarthritis knee | High body-mass index | Number | 2021 | 2261.67523 | 6670.56311 | -184.48831 |
| DALYs (Disability-Adjusted Life Years) | Malawi | Both | All ages | Osteoarthritis knee | High body-mass index | Number | 2021 | 2230.61933 | 6429.52282 | -178.06584 |
| YLDs (Years Lived with Disability) | Malawi | Both | All ages | Osteoarthritis knee | High body-mass index | Number | 2021 | 2230.61933 | 6429.52282 | -178.06584 |
| DALYs (Disability-Adjusted Life Years) | Georgia | Both | All ages | Osteoarthritis knee | High body-mass index | Number | 2021 | 2008.80502 | 5450.48227 | -200.43598 |
| YLDs (Years Lived with Disability) | Georgia | Both | All ages | Osteoarthritis knee | High body-mass index | Number | 2021 | 2008.80502 | 5450.48227 | -200.43598 |
| DALYs (Disability-Adjusted Life Years) | Tajikistan | Both | All ages | Osteoarthritis knee | High body-mass index | Number | 2021 | 2003.91503 | 5203.7552 | -216.70723 |
| YLDs (Years Lived with Disability) | Tajikistan | Both | All ages | Osteoarthritis knee | High body-mass index | Number | 2021 | 2003.91503 | 5203.7552 | -216.70723 |
| DALYs (Disability-Adjusted Life Years) | Guinea | Both | All ages | Osteoarthritis knee | High body-mass index | Number | 2021 | 1915.19519 | 5463.35268 | -155.13481 |
| YLDs (Years Lived with Disability) | Guinea | Both | All ages | Osteoarthritis knee | High body-mass index | Number | 2021 | 1915.19519 | 5463.35268 | -155.13481 |
| DALYs (Disability-Adjusted Life Years) | Jamaica | Both | All ages | Osteoarthritis knee | High body-mass index | Number | 2021 | 1891.28303 | 4994.134 | -196.28255 |
| YLDs (Years Lived with Disability) | Jamaica | Both | All ages | Osteoarthritis knee | High body-mass index | Number | 2021 | 1891.28303 | 4994.134 | -196.28255 |
| DALYs (Disability-Adjusted Life Years) | Kyrgyzstan | Both | All ages | Osteoarthritis knee | High body-mass index | Number | 2021 | 1841.45766 | 4830.57421 | -198.92553 |
| YLDs (Years Lived with Disability) | Kyrgyzstan | Both | All ages | Osteoarthritis knee | High body-mass index | Number | 2021 | 1841.45766 | 4830.57421 | -198.92553 |
| DALYs (Disability-Adjusted Life Years) | Slovenia | Both | All ages | Osteoarthritis knee | High body-mass index | Number | 2021 | 1838.3425 | 4893.64231 | -186.39846 |
| YLDs (Years Lived with Disability) | Slovenia | Both | All ages | Osteoarthritis knee | High body-mass index | Number | 2021 | 1838.3425 | 4893.64231 | -186.39846 |
| DALYs (Disability-Adjusted Life Years) | Albania | Both | All ages | Osteoarthritis knee | High body-mass index | Number | 2021 | 1788.50956 | 4668.90355 | -190.32226 |
| YLDs (Years Lived with Disability) | Albania | Both | All ages | Osteoarthritis knee | High body-mass index | Number | 2021 | 1788.50956 | 4668.90355 | -190.32226 |
| DALYs (Disability-Adjusted Life Years) | Latvia | Both | All ages | Osteoarthritis knee | High body-mass index | Number | 2021 | 1780.87067 | 4766.28063 | -198.33166 |
| YLDs (Years Lived with Disability) | Latvia | Both | All ages | Osteoarthritis knee | High body-mass index | Number | 2021 | 1780.87067 | 4766.28063 | -198.33166 |
| DALYs (Disability-Adjusted Life Years) | Rwanda | Both | All ages | Osteoarthritis knee | High body-mass index | Number | 2021 | 1718.32486 | 5137.76535 | -146.17051 |
| YLDs (Years Lived with Disability) | Rwanda | Both | All ages | Osteoarthritis knee | High body-mass index | Number | 2021 | 1718.32486 | 5137.76535 | -146.17051 |
| DALYs (Disability-Adjusted Life Years) | Somalia | Both | All ages | Osteoarthritis knee | High body-mass index | Number | 2021 | 1711.84515 | 4737.16209 | -133.44627 |
| YLDs (Years Lived with Disability) | Somalia | Both | All ages | Osteoarthritis knee | High body-mass index | Number | 2021 | 1711.84515 | 4737.16209 | -133.44627 |
| DALYs (Disability-Adjusted Life Years) | Chad | Both | All ages | Osteoarthritis knee | High body-mass index | Number | 2021 | 1657.22821 | 4660.60248 | -130.45495 |
| YLDs (Years Lived with Disability) | Chad | Both | All ages | Osteoarthritis knee | High body-mass index | Number | 2021 | 1657.22821 | 4660.60248 | -130.45495 |
| DALYs (Disability-Adjusted Life Years) | Palestine | Both | All ages | Osteoarthritis knee | High body-mass index | Number | 2021 | 1617.07412 | 4037.38972 | -201.4612 |
| YLDs (Years Lived with Disability) | Palestine | Both | All ages | Osteoarthritis knee | High body-mass index | Number | 2021 | 1617.07412 | 4037.38972 | -201.4612 |
| DALYs (Disability-Adjusted Life Years) | Togo | Both | All ages | Osteoarthritis knee | High body-mass index | Number | 2021 | 1611.05916 | 4550.75157 | -139.25209 |
| YLDs (Years Lived with Disability) | Togo | Both | All ages | Osteoarthritis knee | High body-mass index | Number | 2021 | 1611.05916 | 4550.75157 | -139.25209 |
| DALYs (Disability-Adjusted Life Years) | Armenia | Both | All ages | Osteoarthritis knee | High body-mass index | Number | 2021 | 1584.22392 | 4267.17189 | -169.19597 |
| YLDs (Years Lived with Disability) | Armenia | Both | All ages | Osteoarthritis knee | High body-mass index | Number | 2021 | 1584.22392 | 4267.17189 | -169.19597 |
| DALYs (Disability-Adjusted Life Years) | Oman | Both | All ages | Osteoarthritis knee | High body-mass index | Number | 2021 | 1569.9848 | 3940.27807 | -185.46208 |
| YLDs (Years Lived with Disability) | Oman | Both | All ages | Osteoarthritis knee | High body-mass index | Number | 2021 | 1569.9848 | 3940.27807 | -185.46208 |
| DALYs (Disability-Adjusted Life Years) | Turkmenistan | Both | All ages | Osteoarthritis knee | High body-mass index | Number | 2021 | 1489.04813 | 3965.22623 | -163.01316 |
| YLDs (Years Lived with Disability) | Turkmenistan | Both | All ages | Osteoarthritis knee | High body-mass index | Number | 2021 | 1489.04813 | 3965.22623 | -163.01316 |
| DALYs (Disability-Adjusted Life Years) | North Macedonia | Both | All ages | Osteoarthritis knee | High body-mass index | Number | 2021 | 1473.01991 | 4062.5247 | -152.22009 |
| YLDs (Years Lived with Disability) | North Macedonia | Both | All ages | Osteoarthritis knee | High body-mass index | Number | 2021 | 1473.01991 | 4062.5247 | -152.22009 |
| DALYs (Disability-Adjusted Life Years) | Sierra Leone | Both | All ages | Osteoarthritis knee | High body-mass index | Number | 2021 | 1338.99743 | 3864.4671 | -108.26989 |
| YLDs (Years Lived with Disability) | Sierra Leone | Both | All ages | Osteoarthritis knee | High body-mass index | Number | 2021 | 1338.99743 | 3864.4671 | -108.26989 |
| DALYs (Disability-Adjusted Life Years) | Trinidad and Tobago | Both | All ages | Osteoarthritis knee | High body-mass index | Number | 2021 | 1225.36327 | 3264.91674 | -136.95271 |
| YLDs (Years Lived with Disability) | Trinidad and Tobago | Both | All ages | Osteoarthritis knee | High body-mass index | Number | 2021 | 1225.36327 | 3264.91674 | -136.95271 |
| DALYs (Disability-Adjusted Life Years) | Lao People's Democratic Republic | Both | All ages | Osteoarthritis knee | High body-mass index | Number | 2021 | 1202.10162 | 3485.55479 | -83.698486 |
| YLDs (Years Lived with Disability) | Lao People's Democratic Republic | Both | All ages | Osteoarthritis knee | High body-mass index | Number | 2021 | 1202.10162 | 3485.55479 | -83.698486 |
| DALYs (Disability-Adjusted Life Years) | Congo | Both | All ages | Osteoarthritis knee | High body-mass index | Number | 2021 | 1196.46266 | 3286.80545 | -109.68694 |
| YLDs (Years Lived with Disability) | Congo | Both | All ages | Osteoarthritis knee | High body-mass index | Number | 2021 | 1196.46266 | 3286.80545 | -109.68694 |
| DALYs (Disability-Adjusted Life Years) | Liberia | Both | All ages | Osteoarthritis knee | High body-mass index | Number | 2021 | 1184.24085 | 3258.33 | -116.72451 |
| YLDs (Years Lived with Disability) | Liberia | Both | All ages | Osteoarthritis knee | High body-mass index | Number | 2021 | 1184.24085 | 3258.33 | -116.72451 |
| DALYs (Disability-Adjusted Life Years) | Estonia | Both | All ages | Osteoarthritis knee | High body-mass index | Number | 2021 | 1179.49054 | 3162.13945 | -125.30922 |
| YLDs (Years Lived with Disability) | Estonia | Both | All ages | Osteoarthritis knee | High body-mass index | Number | 2021 | 1179.49054 | 3162.13945 | -125.30922 |
| DALYs (Disability-Adjusted Life Years) | Mauritania | Both | All ages | Osteoarthritis knee | High body-mass index | Number | 2021 | 1168.31432 | 3105.01914 | -116.88901 |
| YLDs (Years Lived with Disability) | Mauritania | Both | All ages | Osteoarthritis knee | High body-mass index | Number | 2021 | 1168.31432 | 3105.01914 | -116.88901 |
| DALYs (Disability-Adjusted Life Years) | Qatar | Both | All ages | Osteoarthritis knee | High body-mass index | Number | 2021 | 1094.8258 | 2729.52388 | -141.75809 |
| YLDs (Years Lived with Disability) | Qatar | Both | All ages | Osteoarthritis knee | High body-mass index | Number | 2021 | 1094.8258 | 2729.52388 | -141.75809 |
| DALYs (Disability-Adjusted Life Years) | Cyprus | Both | All ages | Osteoarthritis knee | High body-mass index | Number | 2021 | 1031.24852 | 2789.51489 | -103.42011 |
| YLDs (Years Lived with Disability) | Cyprus | Both | All ages | Osteoarthritis knee | High body-mass index | Number | 2021 | 1031.24852 | 2789.51489 | -103.42011 |
| DALYs (Disability-Adjusted Life Years) | Burundi | Both | All ages | Osteoarthritis knee | High body-mass index | Number | 2021 | 922.751645 | 2796.94024 | -68.887348 |
| YLDs (Years Lived with Disability) | Burundi | Both | All ages | Osteoarthritis knee | High body-mass index | Number | 2021 | 922.751645 | 2796.94024 | -68.887348 |
| DALYs (Disability-Adjusted Life Years) | Mongolia | Both | All ages | Osteoarthritis knee | High body-mass index | Number | 2021 | 790.271745 | 2140.75947 | -81.394146 |
| YLDs (Years Lived with Disability) | Mongolia | Both | All ages | Osteoarthritis knee | High body-mass index | Number | 2021 | 790.271745 | 2140.75947 | -81.394146 |
| DALYs (Disability-Adjusted Life Years) | South Sudan | Both | All ages | Osteoarthritis knee | High body-mass index | Number | 2021 | 783.279381 | 2295.90999 | -60.295102 |
| YLDs (Years Lived with Disability) | South Sudan | Both | All ages | Osteoarthritis knee | High body-mass index | Number | 2021 | 783.279381 | 2295.90999 | -60.295102 |
| DALYs (Disability-Adjusted Life Years) | Mauritius | Both | All ages | Osteoarthritis knee | High body-mass index | Number | 2021 | 776.387423 | 2072.57701 | -72.562894 |
| YLDs (Years Lived with Disability) | Mauritius | Both | All ages | Osteoarthritis knee | High body-mass index | Number | 2021 | 776.387423 | 2072.57701 | -72.562894 |
| DALYs (Disability-Adjusted Life Years) | Bahrain | Both | All ages | Osteoarthritis knee | High body-mass index | Number | 2021 | 761.51312 | 1925.62269 | -96.407389 |
| YLDs (Years Lived with Disability) | Bahrain | Both | All ages | Osteoarthritis knee | High body-mass index | Number | 2021 | 761.51312 | 1925.62269 | -96.407389 |
| DALYs (Disability-Adjusted Life Years) | Botswana | Both | All ages | Osteoarthritis knee | High body-mass index | Number | 2021 | 748.594334 | 2141.50443 | -62.986437 |
| YLDs (Years Lived with Disability) | Botswana | Both | All ages | Osteoarthritis knee | High body-mass index | Number | 2021 | 748.594334 | 2141.50443 | -62.986437 |
| DALYs (Disability-Adjusted Life Years) | Central African Republic | Both | All ages | Osteoarthritis knee | High body-mass index | Number | 2021 | 662.501006 | 1831.19681 | -51.241127 |
| YLDs (Years Lived with Disability) | Central African Republic | Both | All ages | Osteoarthritis knee | High body-mass index | Number | 2021 | 662.501006 | 1831.19681 | -51.241127 |
| DALYs (Disability-Adjusted Life Years) | Namibia | Both | All ages | Osteoarthritis knee | High body-mass index | Number | 2021 | 650.323028 | 1827.81252 | -53.037859 |
| YLDs (Years Lived with Disability) | Namibia | Both | All ages | Osteoarthritis knee | High body-mass index | Number | 2021 | 650.323028 | 1827.81252 | -53.037859 |
| DALYs (Disability-Adjusted Life Years) | Gabon | Both | All ages | Osteoarthritis knee | High body-mass index | Number | 2021 | 594.858959 | 1580.92489 | -61.97714 |
| YLDs (Years Lived with Disability) | Gabon | Both | All ages | Osteoarthritis knee | High body-mass index | Number | 2021 | 594.858959 | 1580.92489 | -61.97714 |
| DALYs (Disability-Adjusted Life Years) | Fiji | Both | All ages | Osteoarthritis knee | High body-mass index | Number | 2021 | 586.596752 | 1480.09067 | -69.735571 |
| YLDs (Years Lived with Disability) | Fiji | Both | All ages | Osteoarthritis knee | High body-mass index | Number | 2021 | 586.596752 | 1480.09067 | -69.735571 |
| DALYs (Disability-Adjusted Life Years) | Eritrea | Both | All ages | Osteoarthritis knee | High body-mass index | Number | 2021 | 551.817647 | 1652.41572 | -40.527765 |
| YLDs (Years Lived with Disability) | Eritrea | Both | All ages | Osteoarthritis knee | High body-mass index | Number | 2021 | 551.817647 | 1652.41572 | -40.527765 |
| DALYs (Disability-Adjusted Life Years) | Luxembourg | Both | All ages | Osteoarthritis knee | High body-mass index | Number | 2021 | 541.673696 | 1472.96551 | -50.263524 |
| YLDs (Years Lived with Disability) | Luxembourg | Both | All ages | Osteoarthritis knee | High body-mass index | Number | 2021 | 541.673696 | 1472.96551 | -50.263524 |
| DALYs (Disability-Adjusted Life Years) | Lesotho | Both | All ages | Osteoarthritis knee | High body-mass index | Number | 2021 | 519.260765 | 1392.10706 | -51.812989 |
| YLDs (Years Lived with Disability) | Lesotho | Both | All ages | Osteoarthritis knee | High body-mass index | Number | 2021 | 519.260765 | 1392.10706 | -51.812989 |
| DALYs (Disability-Adjusted Life Years) | Montenegro | Both | All ages | Osteoarthritis knee | High body-mass index | Number | 2021 | 462.372028 | 1212.78678 | -49.355287 |
| YLDs (Years Lived with Disability) | Montenegro | Both | All ages | Osteoarthritis knee | High body-mass index | Number | 2021 | 462.372028 | 1212.78678 | -49.355287 |
| DALYs (Disability-Adjusted Life Years) | Malta | Both | All ages | Osteoarthritis knee | High body-mass index | Number | 2021 | 460.959725 | 1254.94904 | -44.12115 |
| YLDs (Years Lived with Disability) | Malta | Both | All ages | Osteoarthritis knee | High body-mass index | Number | 2021 | 460.959725 | 1254.94904 | -44.12115 |
| DALYs (Disability-Adjusted Life Years) | Gambia | Both | All ages | Osteoarthritis knee | High body-mass index | Number | 2021 | 423.919919 | 1184.91056 | -38.776839 |
| YLDs (Years Lived with Disability) | Gambia | Both | All ages | Osteoarthritis knee | High body-mass index | Number | 2021 | 423.919919 | 1184.91056 | -38.776839 |
| DALYs (Disability-Adjusted Life Years) | Guyana | Both | All ages | Osteoarthritis knee | High body-mass index | Number | 2021 | 366.407002 | 1011.05255 | -35.808653 |
| YLDs (Years Lived with Disability) | Guyana | Both | All ages | Osteoarthritis knee | High body-mass index | Number | 2021 | 366.407002 | 1011.05255 | -35.808653 |
| DALYs (Disability-Adjusted Life Years) | Eswatini | Both | All ages | Osteoarthritis knee | High body-mass index | Number | 2021 | 348.396339 | 907.207617 | -36.27291 |
| YLDs (Years Lived with Disability) | Eswatini | Both | All ages | Osteoarthritis knee | High body-mass index | Number | 2021 | 348.396339 | 907.207617 | -36.27291 |
| DALYs (Disability-Adjusted Life Years) | Suriname | Both | All ages | Osteoarthritis knee | High body-mass index | Number | 2021 | 340.867062 | 921.749549 | -32.242637 |
| YLDs (Years Lived with Disability) | Suriname | Both | All ages | Osteoarthritis knee | High body-mass index | Number | 2021 | 340.867062 | 921.749549 | -32.242637 |
| DALYs (Disability-Adjusted Life Years) | Barbados | Both | All ages | Osteoarthritis knee | High body-mass index | Number | 2021 | 332.99212 | 870.217504 | -33.570161 |
| YLDs (Years Lived with Disability) | Barbados | Both | All ages | Osteoarthritis knee | High body-mass index | Number | 2021 | 332.99212 | 870.217504 | -33.570161 |
| DALYs (Disability-Adjusted Life Years) | Iceland | Both | All ages | Osteoarthritis knee | High body-mass index | Number | 2021 | 309.801164 | 830.346859 | -30.646741 |
| YLDs (Years Lived with Disability) | Iceland | Both | All ages | Osteoarthritis knee | High body-mass index | Number | 2021 | 309.801164 | 830.346859 | -30.646741 |
| DALYs (Disability-Adjusted Life Years) | Bahamas | Both | All ages | Osteoarthritis knee | High body-mass index | Number | 2021 | 286.38047 | 741.866254 | -32.157519 |
| YLDs (Years Lived with Disability) | Bahamas | Both | All ages | Osteoarthritis knee | High body-mass index | Number | 2021 | 286.38047 | 741.866254 | -32.157519 |
| DALYs (Disability-Adjusted Life Years) | Guinea-Bissau | Both | All ages | Osteoarthritis knee | High body-mass index | Number | 2021 | 278.898205 | 802.614255 | -23.360419 |
| YLDs (Years Lived with Disability) | Guinea-Bissau | Both | All ages | Osteoarthritis knee | High body-mass index | Number | 2021 | 278.898205 | 802.614255 | -23.360419 |
| DALYs (Disability-Adjusted Life Years) | Bhutan | Both | All ages | Osteoarthritis knee | High body-mass index | Number | 2021 | 264.455015 | 730.544563 | -24.215302 |
| YLDs (Years Lived with Disability) | Bhutan | Both | All ages | Osteoarthritis knee | High body-mass index | Number | 2021 | 264.455015 | 730.544563 | -24.215302 |
| DALYs (Disability-Adjusted Life Years) | Equatorial Guinea | Both | All ages | Osteoarthritis knee | High body-mass index | Number | 2021 | 261.111282 | 716.612713 | -24.167722 |
| YLDs (Years Lived with Disability) | Equatorial Guinea | Both | All ages | Osteoarthritis knee | High body-mass index | Number | 2021 | 261.111282 | 716.612713 | -24.167722 |
| DALYs (Disability-Adjusted Life Years) | Brunei Darussalam | Both | All ages | Osteoarthritis knee | High body-mass index | Number | 2021 | 253.110247 | 713.875852 | -23.442359 |
| YLDs (Years Lived with Disability) | Brunei Darussalam | Both | All ages | Osteoarthritis knee | High body-mass index | Number | 2021 | 253.110247 | 713.875852 | -23.442359 |
| DALYs (Disability-Adjusted Life Years) | Belize | Both | All ages | Osteoarthritis knee | High body-mass index | Number | 2021 | 215.112748 | 572.253535 | -22.538227 |
| YLDs (Years Lived with Disability) | Belize | Both | All ages | Osteoarthritis knee | High body-mass index | Number | 2021 | 215.112748 | 572.253535 | -22.538227 |
| DALYs (Disability-Adjusted Life Years) | Cabo Verde | Both | All ages | Osteoarthritis knee | High body-mass index | Number | 2021 | 211.681201 | 594.310735 | -19.96566 |
| YLDs (Years Lived with Disability) | Cabo Verde | Both | All ages | Osteoarthritis knee | High body-mass index | Number | 2021 | 211.681201 | 594.310735 | -19.96566 |
| DALYs (Disability-Adjusted Life Years) | Solomon Islands | Both | All ages | Osteoarthritis knee | High body-mass index | Number | 2021 | 202.737585 | 544.150824 | -21.134452 |
| YLDs (Years Lived with Disability) | Solomon Islands | Both | All ages | Osteoarthritis knee | High body-mass index | Number | 2021 | 202.737585 | 544.150824 | -21.134452 |
| DALYs (Disability-Adjusted Life Years) | Comoros | Both | All ages | Osteoarthritis knee | High body-mass index | Number | 2021 | 177.920573 | 506.375229 | -14.595995 |
| YLDs (Years Lived with Disability) | Comoros | Both | All ages | Osteoarthritis knee | High body-mass index | Number | 2021 | 177.920573 | 506.375229 | -14.595995 |
| DALYs (Disability-Adjusted Life Years) | Djibouti | Both | All ages | Osteoarthritis knee | High body-mass index | Number | 2021 | 158.62936 | 451.525435 | -11.948466 |
| YLDs (Years Lived with Disability) | Djibouti | Both | All ages | Osteoarthritis knee | High body-mass index | Number | 2021 | 158.62936 | 451.525435 | -11.948466 |
| DALYs (Disability-Adjusted Life Years) | Maldives | Both | All ages | Osteoarthritis knee | High body-mass index | Number | 2021 | 145.283427 | 391.60393 | -13.80038 |
| YLDs (Years Lived with Disability) | Maldives | Both | All ages | Osteoarthritis knee | High body-mass index | Number | 2021 | 145.283427 | 391.60393 | -13.80038 |
| DALYs (Disability-Adjusted Life Years) | Saint Lucia | Both | All ages | Osteoarthritis knee | High body-mass index | Number | 2021 | 140.414251 | 390.603439 | -13.94707 |
| YLDs (Years Lived with Disability) | Saint Lucia | Both | All ages | Osteoarthritis knee | High body-mass index | Number | 2021 | 140.414251 | 390.603439 | -13.94707 |
| DALYs (Disability-Adjusted Life Years) | Guam | Both | All ages | Osteoarthritis knee | High body-mass index | Number | 2021 | 140.327416 | 373.771309 | -15.605769 |
| YLDs (Years Lived with Disability) | Guam | Both | All ages | Osteoarthritis knee | High body-mass index | Number | 2021 | 140.327416 | 373.771309 | -15.605769 |
| DALYs (Disability-Adjusted Life Years) | United States Virgin Islands | Both | All ages | Osteoarthritis knee | High body-mass index | Number | 2021 | 121.470673 | 328.877322 | -12.646324 |
| YLDs (Years Lived with Disability) | United States Virgin Islands | Both | All ages | Osteoarthritis knee | High body-mass index | Number | 2021 | 121.470673 | 328.877322 | -12.646324 |
| DALYs (Disability-Adjusted Life Years) | Timor-Leste | Both | All ages | Osteoarthritis knee | High body-mass index | Number | 2021 | 114.843944 | 353.117008 | -7.6079774 |
| YLDs (Years Lived with Disability) | Timor-Leste | Both | All ages | Osteoarthritis knee | High body-mass index | Number | 2021 | 114.843944 | 353.117008 | -7.6079774 |
| DALYs (Disability-Adjusted Life Years) | Samoa | Both | All ages | Osteoarthritis knee | High body-mass index | Number | 2021 | 111.25561 | 277.534331 | -14.387234 |
| YLDs (Years Lived with Disability) | Samoa | Both | All ages | Osteoarthritis knee | High body-mass index | Number | 2021 | 111.25561 | 277.534331 | -14.387234 |
| DALYs (Disability-Adjusted Life Years) | Vanuatu | Both | All ages | Osteoarthritis knee | High body-mass index | Number | 2021 | 100.396805 | 262.71693 | -10.301815 |
| YLDs (Years Lived with Disability) | Vanuatu | Both | All ages | Osteoarthritis knee | High body-mass index | Number | 2021 | 100.396805 | 262.71693 | -10.301815 |
| DALYs (Disability-Adjusted Life Years) | Bermuda | Both | All ages | Osteoarthritis knee | High body-mass index | Number | 2021 | 91.5878362 | 241.570478 | -10.711013 |
| YLDs (Years Lived with Disability) | Bermuda | Both | All ages | Osteoarthritis knee | High body-mass index | Number | 2021 | 91.5878362 | 241.570478 | -10.711013 |
| DALYs (Disability-Adjusted Life Years) | Andorra | Both | All ages | Osteoarthritis knee | High body-mass index | Number | 2021 | 78.0165529 | 212.569082 | -7.1897903 |
| YLDs (Years Lived with Disability) | Andorra | Both | All ages | Osteoarthritis knee | High body-mass index | Number | 2021 | 78.0165529 | 212.569082 | -7.1897903 |
| DALYs (Disability-Adjusted Life Years) | Saint Vincent and the Grenadines | Both | All ages | Osteoarthritis knee | High body-mass index | Number | 2021 | 74.6942019 | 208.557647 | -6.9702925 |
| YLDs (Years Lived with Disability) | Saint Vincent and the Grenadines | Both | All ages | Osteoarthritis knee | High body-mass index | Number | 2021 | 74.6942019 | 208.557647 | -6.9702925 |
| DALYs (Disability-Adjusted Life Years) | Grenada | Both | All ages | Osteoarthritis knee | High body-mass index | Number | 2021 | 67.9328411 | 185.26509 | -7.1109776 |
| YLDs (Years Lived with Disability) | Grenada | Both | All ages | Osteoarthritis knee | High body-mass index | Number | 2021 | 67.9328411 | 185.26509 | -7.1109776 |
| DALYs (Disability-Adjusted Life Years) | Antigua and Barbuda | Both | All ages | Osteoarthritis knee | High body-mass index | Number | 2021 | 66.6023306 | 175.89478 | -7.2576221 |
| YLDs (Years Lived with Disability) | Antigua and Barbuda | Both | All ages | Osteoarthritis knee | High body-mass index | Number | 2021 | 66.6023306 | 175.89478 | -7.2576221 |
| DALYs (Disability-Adjusted Life Years) | Tonga | Both | All ages | Osteoarthritis knee | High body-mass index | Number | 2021 | 63.3148744 | 160.541439 | -8.1198632 |
| YLDs (Years Lived with Disability) | Tonga | Both | All ages | Osteoarthritis knee | High body-mass index | Number | 2021 | 63.3148744 | 160.541439 | -8.1198632 |
| DALYs (Disability-Adjusted Life Years) | Seychelles | Both | All ages | Osteoarthritis knee | High body-mass index | Number | 2021 | 61.2639601 | 159.583458 | -7.1738515 |
| YLDs (Years Lived with Disability) | Seychelles | Both | All ages | Osteoarthritis knee | High body-mass index | Number | 2021 | 61.2639601 | 159.583458 | -7.1738515 |
| DALYs (Disability-Adjusted Life Years) | Dominica | Both | All ages | Osteoarthritis knee | High body-mass index | Number | 2021 | 57.9028897 | 151.582184 | -6.6829256 |
| YLDs (Years Lived with Disability) | Dominica | Both | All ages | Osteoarthritis knee | High body-mass index | Number | 2021 | 57.9028897 | 151.582184 | -6.6829256 |
| DALYs (Disability-Adjusted Life Years) | Micronesia (Federated States of) | Both | All ages | Osteoarthritis knee | High body-mass index | Number | 2021 | 57.706349 | 143.748686 | -6.8184952 |
| YLDs (Years Lived with Disability) | Micronesia (Federated States of) | Both | All ages | Osteoarthritis knee | High body-mass index | Number | 2021 | 57.706349 | 143.748686 | -6.8184952 |
| DALYs (Disability-Adjusted Life Years) | Sao Tome and Principe | Both | All ages | Osteoarthritis knee | High body-mass index | Number | 2021 | 57.4575254 | 157.755311 | -5.6491934 |
| YLDs (Years Lived with Disability) | Sao Tome and Principe | Both | All ages | Osteoarthritis knee | High body-mass index | Number | 2021 | 57.4575254 | 157.755311 | -5.6491934 |
| DALYs (Disability-Adjusted Life Years) | Kiribati | Both | All ages | Osteoarthritis knee | High body-mass index | Number | 2021 | 53.2019844 | 136.105984 | -6.5398672 |
| YLDs (Years Lived with Disability) | Kiribati | Both | All ages | Osteoarthritis knee | High body-mass index | Number | 2021 | 53.2019844 | 136.105984 | -6.5398672 |
| DALYs (Disability-Adjusted Life Years) | Monaco | Both | All ages | Osteoarthritis knee | High body-mass index | Number | 2021 | 49.9657163 | 135.493312 | -4.8822697 |
| YLDs (Years Lived with Disability) | Monaco | Both | All ages | Osteoarthritis knee | High body-mass index | Number | 2021 | 49.9657163 | 135.493312 | -4.8822697 |
| DALYs (Disability-Adjusted Life Years) | Saint Kitts and Nevis | Both | All ages | Osteoarthritis knee | High body-mass index | Number | 2021 | 48.2665548 | 125.575716 | -5.2737117 |
| YLDs (Years Lived with Disability) | Saint Kitts and Nevis | Both | All ages | Osteoarthritis knee | High body-mass index | Number | 2021 | 48.2665548 | 125.575716 | -5.2737117 |
| DALYs (Disability-Adjusted Life Years) | Northern Mariana Islands | Both | All ages | Osteoarthritis knee | High body-mass index | Number | 2021 | 44.9967903 | 112.551988 | -5.4971305 |
| YLDs (Years Lived with Disability) | Northern Mariana Islands | Both | All ages | Osteoarthritis knee | High body-mass index | Number | 2021 | 44.9967903 | 112.551988 | -5.4971305 |
| DALYs (Disability-Adjusted Life Years) | American Samoa | Both | All ages | Osteoarthritis knee | High body-mass index | Number | 2021 | 41.1340771 | 102.398031 | -5.1718931 |
| YLDs (Years Lived with Disability) | American Samoa | Both | All ages | Osteoarthritis knee | High body-mass index | Number | 2021 | 41.1340771 | 102.398031 | -5.1718931 |
| DALYs (Disability-Adjusted Life Years) | San Marino | Both | All ages | Osteoarthritis knee | High body-mass index | Number | 2021 | 36.911762 | 100.595032 | -3.7567391 |
| YLDs (Years Lived with Disability) | San Marino | Both | All ages | Osteoarthritis knee | High body-mass index | Number | 2021 | 36.911762 | 100.595032 | -3.7567391 |
| DALYs (Disability-Adjusted Life Years) | Greenland | Both | All ages | Osteoarthritis knee | High body-mass index | Number | 2021 | 29.3346478 | 77.9452247 | -2.9717277 |
| YLDs (Years Lived with Disability) | Greenland | Both | All ages | Osteoarthritis knee | High body-mass index | Number | 2021 | 29.3346478 | 77.9452247 | -2.9717277 |
| DALYs (Disability-Adjusted Life Years) | Marshall Islands | Both | All ages | Osteoarthritis knee | High body-mass index | Number | 2021 | 25.646493 | 65.4608427 | -3.1572508 |
| YLDs (Years Lived with Disability) | Marshall Islands | Both | All ages | Osteoarthritis knee | High body-mass index | Number | 2021 | 25.646493 | 65.4608427 | -3.1572508 |
| DALYs (Disability-Adjusted Life Years) | Cook Islands | Both | All ages | Osteoarthritis knee | High body-mass index | Number | 2021 | 20.0096232 | 51.1224298 | -2.4307187 |
| YLDs (Years Lived with Disability) | Cook Islands | Both | All ages | Osteoarthritis knee | High body-mass index | Number | 2021 | 20.0096232 | 51.1224298 | -2.4307187 |
| DALYs (Disability-Adjusted Life Years) | Palau | Both | All ages | Osteoarthritis knee | High body-mass index | Number | 2021 | 18.7995097 | 47.8702998 | -2.3059798 |
| YLDs (Years Lived with Disability) | Palau | Both | All ages | Osteoarthritis knee | High body-mass index | Number | 2021 | 18.7995097 | 47.8702998 | -2.3059798 |
| DALYs (Disability-Adjusted Life Years) | Tuvalu | Both | All ages | Osteoarthritis knee | High body-mass index | Number | 2021 | 7.16657244 | 18.2205967 | -0.845122 |
| YLDs (Years Lived with Disability) | Tuvalu | Both | All ages | Osteoarthritis knee | High body-mass index | Number | 2021 | 7.16657244 | 18.2205967 | -0.845122 |
| DALYs (Disability-Adjusted Life Years) | Nauru | Both | All ages | Osteoarthritis knee | High body-mass index | Number | 2021 | 4.78288623 | 11.776935 | -0.6464941 |
| YLDs (Years Lived with Disability) | Nauru | Both | All ages | Osteoarthritis knee | High body-mass index | Number | 2021 | 4.78288623 | 11.776935 | -0.6464941 |
| DALYs (Disability-Adjusted Life Years) | Niue | Both | All ages | Osteoarthritis knee | High body-mass index | Number | 2021 | 1.59801577 | 4.10262453 | -0.1780908 |
| YLDs (Years Lived with Disability) | Niue | Both | All ages | Osteoarthritis knee | High body-mass index | Number | 2021 | 1.59801577 | 4.10262453 | -0.1780908 |
| DALYs (Disability-Adjusted Life Years) | Tokelau | Both | All ages | Osteoarthritis knee | High body-mass index | Number | 2021 | 0.99608847 | 2.54379577 | -0.1154117 |
| YLDs (Years Lived with Disability) | Tokelau | Both | All ages | Osteoarthritis knee | High body-mass index | Number | 2021 | 0.99608847 | 2.54379577 | -0.1154117 |

Appendix 8: In 2021, the age-standardized DALY rates and age-standardized YLD rates for knee osteoarthritis due to high BMI across 204 countries

| measure | location | sex | age | cause | rei | metric | year | val | upper | lower |
| --- | --- | --- | --- | --- | --- | --- | --- | --- | --- | --- |
| DALYs (Disability-Adjusted Life Years) | Cook Islands | Both | Age-standardized | Osteoarthritis knee | High body-mass index | Rate | 2021 | 78.2908649 | 198.299761 | -9.5627646 |
| YLDs (Years Lived with Disability) | Cook Islands | Both | Age-standardized | Osteoarthritis knee | High body-mass index | Rate | 2021 | 78.2908649 | 198.299761 | -9.5627646 |
| DALYs (Disability-Adjusted Life Years) | American Samoa | Both | Age-standardized | Osteoarthritis knee | High body-mass index | Rate | 2021 | 77.2854677 | 194.820981 | -9.4884426 |
| YLDs (Years Lived with Disability) | American Samoa | Both | Age-standardized | Osteoarthritis knee | High body-mass index | Rate | 2021 | 77.2854677 | 194.820981 | -9.4884426 |
| DALYs (Disability-Adjusted Life Years) | Tonga | Both | Age-standardized | Osteoarthritis knee | High body-mass index | Rate | 2021 | 76.0435441 | 194.212847 | -9.6541836 |
| YLDs (Years Lived with Disability) | Tonga | Both | Age-standardized | Osteoarthritis knee | High body-mass index | Rate | 2021 | 76.0435441 | 194.212847 | -9.6541836 |
| DALYs (Disability-Adjusted Life Years) | Puerto Rico | Both | Age-standardized | Osteoarthritis knee | High body-mass index | Rate | 2021 | 73.8605836 | 195.327036 | -8.2627221 |
| YLDs (Years Lived with Disability) | Puerto Rico | Both | Age-standardized | Osteoarthritis knee | High body-mass index | Rate | 2021 | 73.8605836 | 195.327036 | -8.2627221 |
| DALYs (Disability-Adjusted Life Years) | Northern Mariana Islands | Both | Age-standardized | Osteoarthritis knee | High body-mass index | Rate | 2021 | 73.0790857 | 185.163514 | -8.6237661 |
| YLDs (Years Lived with Disability) | Northern Mariana Islands | Both | Age-standardized | Osteoarthritis knee | High body-mass index | Rate | 2021 | 73.0790857 | 185.163514 | -8.6237661 |
| DALYs (Disability-Adjusted Life Years) | United States of America | Both | Age-standardized | Osteoarthritis knee | High body-mass index | Rate | 2021 | 72.8225921 | 190.230408 | -8.1773562 |
| YLDs (Years Lived with Disability) | United States of America | Both | Age-standardized | Osteoarthritis knee | High body-mass index | Rate | 2021 | 72.8225921 | 190.230408 | -8.1773562 |
| DALYs (Disability-Adjusted Life Years) | Nauru | Both | Age-standardized | Osteoarthritis knee | High body-mass index | Rate | 2021 | 72.6897623 | 183.013404 | -9.4364325 |
| YLDs (Years Lived with Disability) | Nauru | Both | Age-standardized | Osteoarthritis knee | High body-mass index | Rate | 2021 | 72.6897623 | 183.013404 | -9.4364325 |
| DALYs (Disability-Adjusted Life Years) | Bermuda | Both | Age-standardized | Osteoarthritis knee | High body-mass index | Rate | 2021 | 72.6065781 | 190.945915 | -8.5519249 |
| YLDs (Years Lived with Disability) | Bermuda | Both | Age-standardized | Osteoarthritis knee | High body-mass index | Rate | 2021 | 72.6065781 | 190.945915 | -8.5519249 |
| DALYs (Disability-Adjusted Life Years) | Niue | Both | Age-standardized | Osteoarthritis knee | High body-mass index | Rate | 2021 | 72.0517083 | 185.649667 | -7.9997991 |
| YLDs (Years Lived with Disability) | Niue | Both | Age-standardized | Osteoarthritis knee | High body-mass index | Rate | 2021 | 72.0517083 | 185.649667 | -7.9997991 |
| DALYs (Disability-Adjusted Life Years) | Palau | Both | Age-standardized | Osteoarthritis knee | High body-mass index | Rate | 2021 | 71.3628455 | 183.2636 | -8.5042004 |
| YLDs (Years Lived with Disability) | Palau | Both | Age-standardized | Osteoarthritis knee | High body-mass index | Rate | 2021 | 71.3628455 | 183.2636 | -8.5042004 |
| DALYs (Disability-Adjusted Life Years) | Samoa | Both | Age-standardized | Osteoarthritis knee | High body-mass index | Rate | 2021 | 71.2965808 | 178.61947 | -9.0470076 |
| YLDs (Years Lived with Disability) | Samoa | Both | Age-standardized | Osteoarthritis knee | High body-mass index | Rate | 2021 | 71.2965808 | 178.61947 | -9.0470076 |
| DALYs (Disability-Adjusted Life Years) | United States Virgin Islands | Both | Age-standardized | Osteoarthritis knee | High body-mass index | Rate | 2021 | 70.713115 | 186.857213 | -7.4818313 |
| YLDs (Years Lived with Disability) | United States Virgin Islands | Both | Age-standardized | Osteoarthritis knee | High body-mass index | Rate | 2021 | 70.713115 | 186.857213 | -7.4818313 |
| DALYs (Disability-Adjusted Life Years) | Chile | Both | Age-standardized | Osteoarthritis knee | High body-mass index | Rate | 2021 | 69.3333849 | 177.889749 | -7.7765241 |
| YLDs (Years Lived with Disability) | Chile | Both | Age-standardized | Osteoarthritis knee | High body-mass index | Rate | 2021 | 69.3333849 | 177.889749 | -7.7765241 |
| DALYs (Disability-Adjusted Life Years) | Australia | Both | Age-standardized | Osteoarthritis knee | High body-mass index | Rate | 2021 | 69.2945531 | 185.320904 | -7.0831459 |
| YLDs (Years Lived with Disability) | Australia | Both | Age-standardized | Osteoarthritis knee | High body-mass index | Rate | 2021 | 69.2945531 | 185.320904 | -7.0831459 |
| DALYs (Disability-Adjusted Life Years) | Fiji | Both | Age-standardized | Osteoarthritis knee | High body-mass index | Rate | 2021 | 69.127588 | 176.441768 | -8.0792076 |
| YLDs (Years Lived with Disability) | Fiji | Both | Age-standardized | Osteoarthritis knee | High body-mass index | Rate | 2021 | 69.127588 | 176.441768 | -8.0792076 |
| DALYs (Disability-Adjusted Life Years) | Kuwait | Both | Age-standardized | Osteoarthritis knee | High body-mass index | Rate | 2021 | 68.4059843 | 170.548101 | -8.7737046 |
| YLDs (Years Lived with Disability) | Kuwait | Both | Age-standardized | Osteoarthritis knee | High body-mass index | Rate | 2021 | 68.4059843 | 170.548101 | -8.7737046 |
| DALYs (Disability-Adjusted Life Years) | Ecuador | Both | Age-standardized | Osteoarthritis knee | High body-mass index | Rate | 2021 | 68.0117707 | 182.489124 | -7.0466414 |
| YLDs (Years Lived with Disability) | Ecuador | Both | Age-standardized | Osteoarthritis knee | High body-mass index | Rate | 2021 | 68.0117707 | 182.489124 | -7.0466414 |
| DALYs (Disability-Adjusted Life Years) | Tokelau | Both | Age-standardized | Osteoarthritis knee | High body-mass index | Rate | 2021 | 67.7883425 | 172.635564 | -7.8691392 |
| YLDs (Years Lived with Disability) | Tokelau | Both | Age-standardized | Osteoarthritis knee | High body-mass index | Rate | 2021 | 67.7883425 | 172.635564 | -7.8691392 |
| DALYs (Disability-Adjusted Life Years) | Micronesia (Federated States of) | Both | Age-standardized | Osteoarthritis knee | High body-mass index | Rate | 2021 | 67.4258167 | 172.10965 | -7.6984757 |
| YLDs (Years Lived with Disability) | Micronesia (Federated States of) | Both | Age-standardized | Osteoarthritis knee | High body-mass index | Rate | 2021 | 67.4258167 | 172.10965 | -7.6984757 |
| DALYs (Disability-Adjusted Life Years) | Dominica | Both | Age-standardized | Osteoarthritis knee | High body-mass index | Rate | 2021 | 67.4094967 | 177.062687 | -7.7402738 |
| YLDs (Years Lived with Disability) | Dominica | Both | Age-standardized | Osteoarthritis knee | High body-mass index | Rate | 2021 | 67.4094967 | 177.062687 | -7.7402738 |
| DALYs (Disability-Adjusted Life Years) | Belize | Both | Age-standardized | Osteoarthritis knee | High body-mass index | Rate | 2021 | 67.1693432 | 180.875052 | -6.960941 |
| YLDs (Years Lived with Disability) | Belize | Both | Age-standardized | Osteoarthritis knee | High body-mass index | Rate | 2021 | 67.1693432 | 180.875052 | -6.960941 |
| DALYs (Disability-Adjusted Life Years) | Guam | Both | Age-standardized | Osteoarthritis knee | High body-mass index | Rate | 2021 | 66.7169401 | 177.156527 | -7.4100933 |
| YLDs (Years Lived with Disability) | Guam | Both | Age-standardized | Osteoarthritis knee | High body-mass index | Rate | 2021 | 66.7169401 | 177.156527 | -7.4100933 |
| DALYs (Disability-Adjusted Life Years) | Qatar | Both | Age-standardized | Osteoarthritis knee | High body-mass index | Rate | 2021 | 66.476621 | 172.545081 | -8.2121202 |
| YLDs (Years Lived with Disability) | Qatar | Both | Age-standardized | Osteoarthritis knee | High body-mass index | Rate | 2021 | 66.476621 | 172.545081 | -8.2121202 |
| DALYs (Disability-Adjusted Life Years) | Barbados | Both | Age-standardized | Osteoarthritis knee | High body-mass index | Rate | 2021 | 66.4410766 | 173.16663 | -6.7262547 |
| YLDs (Years Lived with Disability) | Barbados | Both | Age-standardized | Osteoarthritis knee | High body-mass index | Rate | 2021 | 66.4410766 | 173.16663 | -6.7262547 |
| DALYs (Disability-Adjusted Life Years) | Argentina | Both | Age-standardized | Osteoarthritis knee | High body-mass index | Rate | 2021 | 65.6558251 | 170.620498 | -7.224961 |
| YLDs (Years Lived with Disability) | Argentina | Both | Age-standardized | Osteoarthritis knee | High body-mass index | Rate | 2021 | 65.6558251 | 170.620498 | -7.224961 |
| DALYs (Disability-Adjusted Life Years) | Bahamas | Both | Age-standardized | Osteoarthritis knee | High body-mass index | Rate | 2021 | 65.6407929 | 171.897542 | -7.2262576 |
| YLDs (Years Lived with Disability) | Bahamas | Both | Age-standardized | Osteoarthritis knee | High body-mass index | Rate | 2021 | 65.6407929 | 171.897542 | -7.2262576 |
| DALYs (Disability-Adjusted Life Years) | El Salvador | Both | Age-standardized | Osteoarthritis knee | High body-mass index | Rate | 2021 | 65.2810296 | 169.559073 | -7.1191168 |
| YLDs (Years Lived with Disability) | El Salvador | Both | Age-standardized | Osteoarthritis knee | High body-mass index | Rate | 2021 | 65.2810296 | 169.559073 | -7.1191168 |
| DALYs (Disability-Adjusted Life Years) | Mexico | Both | Age-standardized | Osteoarthritis knee | High body-mass index | Rate | 2021 | 65.1578815 | 172.415718 | -7.2307813 |
| YLDs (Years Lived with Disability) | Mexico | Both | Age-standardized | Osteoarthritis knee | High body-mass index | Rate | 2021 | 65.1578815 | 172.415718 | -7.2307813 |
| DALYs (Disability-Adjusted Life Years) | Tuvalu | Both | Age-standardized | Osteoarthritis knee | High body-mass index | Rate | 2021 | 64.9754791 | 165.692234 | -7.5566399 |
| YLDs (Years Lived with Disability) | Tuvalu | Both | Age-standardized | Osteoarthritis knee | High body-mass index | Rate | 2021 | 64.9754791 | 165.692234 | -7.5566399 |
| DALYs (Disability-Adjusted Life Years) | Venezuela (Bolivarian Republic of) | Both | Age-standardized | Osteoarthritis knee | High body-mass index | Rate | 2021 | 64.3346573 | 169.82939 | -6.843068 |
| YLDs (Years Lived with Disability) | Venezuela (Bolivarian Republic of) | Both | Age-standardized | Osteoarthritis knee | High body-mass index | Rate | 2021 | 64.3346573 | 169.82939 | -6.843068 |
| DALYs (Disability-Adjusted Life Years) | Saudi Arabia | Both | Age-standardized | Osteoarthritis knee | High body-mass index | Rate | 2021 | 64.2603024 | 163.360032 | -7.6489375 |
| YLDs (Years Lived with Disability) | Saudi Arabia | Both | Age-standardized | Osteoarthritis knee | High body-mass index | Rate | 2021 | 64.2603024 | 163.360032 | -7.6489375 |
| DALYs (Disability-Adjusted Life Years) | Kiribati | Both | Age-standardized | Osteoarthritis knee | High body-mass index | Rate | 2021 | 63.9757746 | 166.591432 | -7.5617062 |
| YLDs (Years Lived with Disability) | Kiribati | Both | Age-standardized | Osteoarthritis knee | High body-mass index | Rate | 2021 | 63.9757746 | 166.591432 | -7.5617062 |
| DALYs (Disability-Adjusted Life Years) | Jordan | Both | Age-standardized | Osteoarthritis knee | High body-mass index | Rate | 2021 | 63.7278573 | 164.052699 | -7.945426 |
| YLDs (Years Lived with Disability) | Jordan | Both | Age-standardized | Osteoarthritis knee | High body-mass index | Rate | 2021 | 63.7278573 | 164.052699 | -7.945426 |
| DALYs (Disability-Adjusted Life Years) | New Zealand | Both | Age-standardized | Osteoarthritis knee | High body-mass index | Rate | 2021 | 63.6062563 | 171.790321 | -7.0099504 |
| YLDs (Years Lived with Disability) | New Zealand | Both | Age-standardized | Osteoarthritis knee | High body-mass index | Rate | 2021 | 63.6062563 | 171.790321 | -7.0099504 |
| DALYs (Disability-Adjusted Life Years) | Uruguay | Both | Age-standardized | Osteoarthritis knee | High body-mass index | Rate | 2021 | 63.2294244 | 167.613038 | -6.3220876 |
| YLDs (Years Lived with Disability) | Uruguay | Both | Age-standardized | Osteoarthritis knee | High body-mass index | Rate | 2021 | 63.2294244 | 167.613038 | -6.3220876 |
| DALYs (Disability-Adjusted Life Years) | Saint Kitts and Nevis | Both | Age-standardized | Osteoarthritis knee | High body-mass index | Rate | 2021 | 63.1598381 | 166.209659 | -6.730002 |
| YLDs (Years Lived with Disability) | Saint Kitts and Nevis | Both | Age-standardized | Osteoarthritis knee | High body-mass index | Rate | 2021 | 63.1598381 | 166.209659 | -6.730002 |
| DALYs (Disability-Adjusted Life Years) | Panama | Both | Age-standardized | Osteoarthritis knee | High body-mass index | Rate | 2021 | 62.7131208 | 168.543149 | -6.6647246 |
| YLDs (Years Lived with Disability) | Panama | Both | Age-standardized | Osteoarthritis knee | High body-mass index | Rate | 2021 | 62.7131208 | 168.543149 | -6.6647246 |
| DALYs (Disability-Adjusted Life Years) | Trinidad and Tobago | Both | Age-standardized | Osteoarthritis knee | High body-mass index | Rate | 2021 | 62.7030799 | 167.233172 | -6.986212 |
| YLDs (Years Lived with Disability) | Trinidad and Tobago | Both | Age-standardized | Osteoarthritis knee | High body-mass index | Rate | 2021 | 62.7030799 | 167.233172 | -6.986212 |
| DALYs (Disability-Adjusted Life Years) | Egypt | Both | Age-standardized | Osteoarthritis knee | High body-mass index | Rate | 2021 | 62.52077 | 160.534346 | -7.7130792 |
| YLDs (Years Lived with Disability) | Egypt | Both | Age-standardized | Osteoarthritis knee | High body-mass index | Rate | 2021 | 62.52077 | 160.534346 | -7.7130792 |
| DALYs (Disability-Adjusted Life Years) | Nicaragua | Both | Age-standardized | Osteoarthritis knee | High body-mass index | Rate | 2021 | 62.3774194 | 165.728091 | -7.1770005 |
| YLDs (Years Lived with Disability) | Nicaragua | Both | Age-standardized | Osteoarthritis knee | High body-mass index | Rate | 2021 | 62.3774194 | 165.728091 | -7.1770005 |
| DALYs (Disability-Adjusted Life Years) | Costa Rica | Both | Age-standardized | Osteoarthritis knee | High body-mass index | Rate | 2021 | 62.0476182 | 166.005528 | -6.7483997 |
| YLDs (Years Lived with Disability) | Costa Rica | Both | Age-standardized | Osteoarthritis knee | High body-mass index | Rate | 2021 | 62.0476182 | 166.005528 | -6.7483997 |
| DALYs (Disability-Adjusted Life Years) | Marshall Islands | Both | Age-standardized | Osteoarthritis knee | High body-mass index | Rate | 2021 | 61.9594468 | 162.35509 | -7.4400989 |
| YLDs (Years Lived with Disability) | Marshall Islands | Both | Age-standardized | Osteoarthritis knee | High body-mass index | Rate | 2021 | 61.9594468 | 162.35509 | -7.4400989 |
| DALYs (Disability-Adjusted Life Years) | United Arab Emirates | Both | Age-standardized | Osteoarthritis knee | High body-mass index | Rate | 2021 | 61.9511388 | 159.684126 | -7.3434258 |
| YLDs (Years Lived with Disability) | United Arab Emirates | Both | Age-standardized | Osteoarthritis knee | High body-mass index | Rate | 2021 | 61.9511388 | 159.684126 | -7.3434258 |
| DALYs (Disability-Adjusted Life Years) | T眉rkiye | Both | Age-standardized | Osteoarthritis knee | High body-mass index | Rate | 2021 | 61.8688682 | 158.314096 | -7.3006712 |
| YLDs (Years Lived with Disability) | T眉rkiye | Both | Age-standardized | Osteoarthritis knee | High body-mass index | Rate | 2021 | 61.8688682 | 158.314096 | -7.3006712 |
| DALYs (Disability-Adjusted Life Years) | United Kingdom | Both | Age-standardized | Osteoarthritis knee | High body-mass index | Rate | 2021 | 61.5019398 | 163.103115 | -6.5877926 |
| YLDs (Years Lived with Disability) | United Kingdom | Both | Age-standardized | Osteoarthritis knee | High body-mass index | Rate | 2021 | 61.5019398 | 163.103115 | -6.5877926 |
| DALYs (Disability-Adjusted Life Years) | Singapore | Both | Age-standardized | Osteoarthritis knee | High body-mass index | Rate | 2021 | 61.2570302 | 174.209716 | -4.912067 |
| YLDs (Years Lived with Disability) | Singapore | Both | Age-standardized | Osteoarthritis knee | High body-mass index | Rate | 2021 | 61.2570302 | 174.209716 | -4.912067 |
| DALYs (Disability-Adjusted Life Years) | Jamaica | Both | Age-standardized | Osteoarthritis knee | High body-mass index | Rate | 2021 | 61.2103239 | 161.708397 | -6.3448202 |
| YLDs (Years Lived with Disability) | Jamaica | Both | Age-standardized | Osteoarthritis knee | High body-mass index | Rate | 2021 | 61.2103239 | 161.708397 | -6.3448202 |
| DALYs (Disability-Adjusted Life Years) | Brunei Darussalam | Both | Age-standardized | Osteoarthritis knee | High body-mass index | Rate | 2021 | 61.197436 | 174.517675 | -5.5644303 |
| YLDs (Years Lived with Disability) | Brunei Darussalam | Both | Age-standardized | Osteoarthritis knee | High body-mass index | Rate | 2021 | 61.197436 | 174.517675 | -5.5644303 |
| DALYs (Disability-Adjusted Life Years) | Bahrain | Both | Age-standardized | Osteoarthritis knee | High body-mass index | Rate | 2021 | 61.1127692 | 156.824219 | -7.4194473 |
| YLDs (Years Lived with Disability) | Bahrain | Both | Age-standardized | Osteoarthritis knee | High body-mass index | Rate | 2021 | 61.1127692 | 156.824219 | -7.4194473 |
| DALYs (Disability-Adjusted Life Years) | Libya | Both | Age-standardized | Osteoarthritis knee | High body-mass index | Rate | 2021 | 60.8406775 | 155.678626 | -7.475808 |
| YLDs (Years Lived with Disability) | Libya | Both | Age-standardized | Osteoarthritis knee | High body-mass index | Rate | 2021 | 60.8406775 | 155.678626 | -7.475808 |
| DALYs (Disability-Adjusted Life Years) | Oman | Both | Age-standardized | Osteoarthritis knee | High body-mass index | Rate | 2021 | 59.8546462 | 153.909956 | -6.9296105 |
| YLDs (Years Lived with Disability) | Oman | Both | Age-standardized | Osteoarthritis knee | High body-mass index | Rate | 2021 | 59.8546462 | 153.909956 | -6.9296105 |
| DALYs (Disability-Adjusted Life Years) | Syrian Arab Republic | Both | Age-standardized | Osteoarthritis knee | High body-mass index | Rate | 2021 | 59.7200483 | 155.230922 | -7.3381248 |
| YLDs (Years Lived with Disability) | Syrian Arab Republic | Both | Age-standardized | Osteoarthritis knee | High body-mass index | Rate | 2021 | 59.7200483 | 155.230922 | -7.3381248 |
| DALYs (Disability-Adjusted Life Years) | Taiwan (Province of China) | Both | Age-standardized | Osteoarthritis knee | High body-mass index | Rate | 2021 | 59.5584851 | 170.685861 | -5.8761961 |
| YLDs (Years Lived with Disability) | Taiwan (Province of China) | Both | Age-standardized | Osteoarthritis knee | High body-mass index | Rate | 2021 | 59.5584851 | 170.685861 | -5.8761961 |
| DALYs (Disability-Adjusted Life Years) | Antigua and Barbuda | Both | Age-standardized | Osteoarthritis knee | High body-mass index | Rate | 2021 | 59.075939 | 156.935751 | -6.3627297 |
| YLDs (Years Lived with Disability) | Antigua and Barbuda | Both | Age-standardized | Osteoarthritis knee | High body-mass index | Rate | 2021 | 59.075939 | 156.935751 | -6.3627297 |
| DALYs (Disability-Adjusted Life Years) | Colombia | Both | Age-standardized | Osteoarthritis knee | High body-mass index | Rate | 2021 | 58.9420529 | 158.371327 | -6.1567937 |
| YLDs (Years Lived with Disability) | Colombia | Both | Age-standardized | Osteoarthritis knee | High body-mass index | Rate | 2021 | 58.9420529 | 158.371327 | -6.1567937 |
| DALYs (Disability-Adjusted Life Years) | Peru | Both | Age-standardized | Osteoarthritis knee | High body-mass index | Rate | 2021 | 58.9396695 | 157.57248 | -6.4283605 |
| YLDs (Years Lived with Disability) | Peru | Both | Age-standardized | Osteoarthritis knee | High body-mass index | Rate | 2021 | 58.9396695 | 157.57248 | -6.4283605 |
| DALYs (Disability-Adjusted Life Years) | Paraguay | Both | Age-standardized | Osteoarthritis knee | High body-mass index | Rate | 2021 | 58.6293552 | 157.995299 | -6.4940144 |
| YLDs (Years Lived with Disability) | Paraguay | Both | Age-standardized | Osteoarthritis knee | High body-mass index | Rate | 2021 | 58.6293552 | 157.995299 | -6.4940144 |
| DALYs (Disability-Adjusted Life Years) | Eswatini | Both | Age-standardized | Osteoarthritis knee | High body-mass index | Rate | 2021 | 58.4719988 | 152.658332 | -6.0043874 |
| YLDs (Years Lived with Disability) | Eswatini | Both | Age-standardized | Osteoarthritis knee | High body-mass index | Rate | 2021 | 58.4719988 | 152.658332 | -6.0043874 |
| DALYs (Disability-Adjusted Life Years) | Monaco | Both | Age-standardized | Osteoarthritis knee | High body-mass index | Rate | 2021 | 58.2330458 | 154.768525 | -5.8631484 |
| YLDs (Years Lived with Disability) | Monaco | Both | Age-standardized | Osteoarthritis knee | High body-mass index | Rate | 2021 | 58.2330458 | 154.768525 | -5.8631484 |
| DALYs (Disability-Adjusted Life Years) | Brazil | Both | Age-standardized | Osteoarthritis knee | High body-mass index | Rate | 2021 | 58.0125876 | 155.3104 | -5.8562999 |
| YLDs (Years Lived with Disability) | Brazil | Both | Age-standardized | Osteoarthritis knee | High body-mass index | Rate | 2021 | 58.0125876 | 155.3104 | -5.8562999 |
| DALYs (Disability-Adjusted Life Years) | Cuba | Both | Age-standardized | Osteoarthritis knee | High body-mass index | Rate | 2021 | 57.8454088 | 156.341359 | -5.7801513 |
| YLDs (Years Lived with Disability) | Cuba | Both | Age-standardized | Osteoarthritis knee | High body-mass index | Rate | 2021 | 57.8454088 | 156.341359 | -5.7801513 |
| DALYs (Disability-Adjusted Life Years) | Spain | Both | Age-standardized | Osteoarthritis knee | High body-mass index | Rate | 2021 | 57.8322094 | 155.359112 | -6.0718498 |
| YLDs (Years Lived with Disability) | Spain | Both | Age-standardized | Osteoarthritis knee | High body-mass index | Rate | 2021 | 57.8322094 | 155.359112 | -6.0718498 |
| DALYs (Disability-Adjusted Life Years) | Iceland | Both | Age-standardized | Osteoarthritis knee | High body-mass index | Rate | 2021 | 57.1657259 | 152.462223 | -5.7233212 |
| YLDs (Years Lived with Disability) | Iceland | Both | Age-standardized | Osteoarthritis knee | High body-mass index | Rate | 2021 | 57.1657259 | 152.462223 | -5.7233212 |
| DALYs (Disability-Adjusted Life Years) | Lebanon | Both | Age-standardized | Osteoarthritis knee | High body-mass index | Rate | 2021 | 57.1195676 | 146.809309 | -6.6974566 |
| YLDs (Years Lived with Disability) | Lebanon | Both | Age-standardized | Osteoarthritis knee | High body-mass index | Rate | 2021 | 57.1195676 | 146.809309 | -6.6974566 |
| DALYs (Disability-Adjusted Life Years) | Guatemala | Both | Age-standardized | Osteoarthritis knee | High body-mass index | Rate | 2021 | 56.9327603 | 152.148583 | -5.6697355 |
| YLDs (Years Lived with Disability) | Guatemala | Both | Age-standardized | Osteoarthritis knee | High body-mass index | Rate | 2021 | 56.9327603 | 152.148583 | -5.6697355 |
| DALYs (Disability-Adjusted Life Years) | Saint Lucia | Both | Age-standardized | Osteoarthritis knee | High body-mass index | Rate | 2021 | 56.9327215 | 158.644107 | -5.6080632 |
| YLDs (Years Lived with Disability) | Saint Lucia | Both | Age-standardized | Osteoarthritis knee | High body-mass index | Rate | 2021 | 56.9327215 | 158.644107 | -5.6080632 |
| DALYs (Disability-Adjusted Life Years) | Palestine | Both | Age-standardized | Osteoarthritis knee | High body-mass index | Rate | 2021 | 56.8414624 | 144.168084 | -6.9219052 |
| YLDs (Years Lived with Disability) | Palestine | Both | Age-standardized | Osteoarthritis knee | High body-mass index | Rate | 2021 | 56.8414624 | 144.168084 | -6.9219052 |
| DALYs (Disability-Adjusted Life Years) | Bolivia (Plurinational State of) | Both | Age-standardized | Osteoarthritis knee | High body-mass index | Rate | 2021 | 56.2261641 | 152.597231 | -5.6747888 |
| YLDs (Years Lived with Disability) | Bolivia (Plurinational State of) | Both | Age-standardized | Osteoarthritis knee | High body-mass index | Rate | 2021 | 56.2261641 | 152.597231 | -5.6747888 |
| DALYs (Disability-Adjusted Life Years) | Grenada | Both | Age-standardized | Osteoarthritis knee | High body-mass index | Rate | 2021 | 56.1941109 | 154.268922 | -5.7767995 |
| YLDs (Years Lived with Disability) | Grenada | Both | Age-standardized | Osteoarthritis knee | High body-mass index | Rate | 2021 | 56.1941109 | 154.268922 | -5.7767995 |
| DALYs (Disability-Adjusted Life Years) | Greece | Both | Age-standardized | Osteoarthritis knee | High body-mass index | Rate | 2021 | 56.1747377 | 151.211566 | -5.638568 |
| YLDs (Years Lived with Disability) | Greece | Both | Age-standardized | Osteoarthritis knee | High body-mass index | Rate | 2021 | 56.1747377 | 151.211566 | -5.638568 |
| DALYs (Disability-Adjusted Life Years) | San Marino | Both | Age-standardized | Osteoarthritis knee | High body-mass index | Rate | 2021 | 56.0122269 | 150.849981 | -5.8294201 |
| YLDs (Years Lived with Disability) | San Marino | Both | Age-standardized | Osteoarthritis knee | High body-mass index | Rate | 2021 | 56.0122269 | 150.849981 | -5.8294201 |
| DALYs (Disability-Adjusted Life Years) | South Africa | Both | Age-standardized | Osteoarthritis knee | High body-mass index | Rate | 2021 | 55.5708994 | 148.714184 | -5.7943294 |
| YLDs (Years Lived with Disability) | South Africa | Both | Age-standardized | Osteoarthritis knee | High body-mass index | Rate | 2021 | 55.5708994 | 148.714184 | -5.7943294 |
| DALYs (Disability-Adjusted Life Years) | Honduras | Both | Age-standardized | Osteoarthritis knee | High body-mass index | Rate | 2021 | 55.1788425 | 145.729314 | -6.0518415 |
| YLDs (Years Lived with Disability) | Honduras | Both | Age-standardized | Osteoarthritis knee | High body-mass index | Rate | 2021 | 55.1788425 | 145.729314 | -6.0518415 |
| DALYs (Disability-Adjusted Life Years) | Ireland | Both | Age-standardized | Osteoarthritis knee | High body-mass index | Rate | 2021 | 54.717101 | 144.600092 | -5.6381418 |
| YLDs (Years Lived with Disability) | Ireland | Both | Age-standardized | Osteoarthritis knee | High body-mass index | Rate | 2021 | 54.717101 | 144.600092 | -5.6381418 |
| DALYs (Disability-Adjusted Life Years) | Republic of Korea | Both | Age-standardized | Osteoarthritis knee | High body-mass index | Rate | 2021 | 54.480267 | 160.778644 | -4.5741148 |
| YLDs (Years Lived with Disability) | Republic of Korea | Both | Age-standardized | Osteoarthritis knee | High body-mass index | Rate | 2021 | 54.480267 | 160.778644 | -4.5741148 |
| DALYs (Disability-Adjusted Life Years) | Iraq | Both | Age-standardized | Osteoarthritis knee | High body-mass index | Rate | 2021 | 54.2636503 | 142.857082 | -5.9488032 |
| YLDs (Years Lived with Disability) | Iraq | Both | Age-standardized | Osteoarthritis knee | High body-mass index | Rate | 2021 | 54.2636503 | 142.857082 | -5.9488032 |
| DALYs (Disability-Adjusted Life Years) | Israel | Both | Age-standardized | Osteoarthritis knee | High body-mass index | Rate | 2021 | 54.2315104 | 147.944149 | -5.2372652 |
| YLDs (Years Lived with Disability) | Israel | Both | Age-standardized | Osteoarthritis knee | High body-mass index | Rate | 2021 | 54.2315104 | 147.944149 | -5.2372652 |
| DALYs (Disability-Adjusted Life Years) | Germany | Both | Age-standardized | Osteoarthritis knee | High body-mass index | Rate | 2021 | 53.9944315 | 144.403059 | -5.4782034 |
| YLDs (Years Lived with Disability) | Germany | Both | Age-standardized | Osteoarthritis knee | High body-mass index | Rate | 2021 | 53.9944315 | 144.403059 | -5.4782034 |
| DALYs (Disability-Adjusted Life Years) | Finland | Both | Age-standardized | Osteoarthritis knee | High body-mass index | Rate | 2021 | 53.9792359 | 145.632851 | -5.5644457 |
| YLDs (Years Lived with Disability) | Finland | Both | Age-standardized | Osteoarthritis knee | High body-mass index | Rate | 2021 | 53.9792359 | 145.632851 | -5.5644457 |
| DALYs (Disability-Adjusted Life Years) | Dominican Republic | Both | Age-standardized | Osteoarthritis knee | High body-mass index | Rate | 2021 | 53.9523037 | 149.193291 | -5.3375254 |
| YLDs (Years Lived with Disability) | Dominican Republic | Both | Age-standardized | Osteoarthritis knee | High body-mass index | Rate | 2021 | 53.9523037 | 149.193291 | -5.3375254 |
| DALYs (Disability-Adjusted Life Years) | Portugal | Both | Age-standardized | Osteoarthritis knee | High body-mass index | Rate | 2021 | 53.7077892 | 141.12324 | -5.5837655 |
| YLDs (Years Lived with Disability) | Portugal | Both | Age-standardized | Osteoarthritis knee | High body-mass index | Rate | 2021 | 53.7077892 | 141.12324 | -5.5837655 |
| DALYs (Disability-Adjusted Life Years) | Cameroon | Both | Age-standardized | Osteoarthritis knee | High body-mass index | Rate | 2021 | 53.4045393 | 143.74576 | -5.6326928 |
| YLDs (Years Lived with Disability) | Cameroon | Both | Age-standardized | Osteoarthritis knee | High body-mass index | Rate | 2021 | 53.4045393 | 143.74576 | -5.6326928 |
| DALYs (Disability-Adjusted Life Years) | Netherlands | Both | Age-standardized | Osteoarthritis knee | High body-mass index | Rate | 2021 | 53.3503782 | 146.800592 | -5.4911769 |
| YLDs (Years Lived with Disability) | Netherlands | Both | Age-standardized | Osteoarthritis knee | High body-mass index | Rate | 2021 | 53.3503782 | 146.800592 | -5.4911769 |
| DALYs (Disability-Adjusted Life Years) | Guyana | Both | Age-standardized | Osteoarthritis knee | High body-mass index | Rate | 2021 | 53.3151876 | 148.168819 | -5.1113059 |
| YLDs (Years Lived with Disability) | Guyana | Both | Age-standardized | Osteoarthritis knee | High body-mass index | Rate | 2021 | 53.3151876 | 148.168819 | -5.1113059 |
| DALYs (Disability-Adjusted Life Years) | Luxembourg | Both | Age-standardized | Osteoarthritis knee | High body-mass index | Rate | 2021 | 53.1456831 | 144.992253 | -4.9455785 |
| YLDs (Years Lived with Disability) | Luxembourg | Both | Age-standardized | Osteoarthritis knee | High body-mass index | Rate | 2021 | 53.1456831 | 144.992253 | -4.9455785 |
| DALYs (Disability-Adjusted Life Years) | Malta | Both | Age-standardized | Osteoarthritis knee | High body-mass index | Rate | 2021 | 52.7182111 | 142.793069 | -5.1484127 |
| YLDs (Years Lived with Disability) | Malta | Both | Age-standardized | Osteoarthritis knee | High body-mass index | Rate | 2021 | 52.7182111 | 142.793069 | -5.1484127 |
| DALYs (Disability-Adjusted Life Years) | Algeria | Both | Age-standardized | Osteoarthritis knee | High body-mass index | Rate | 2021 | 51.8563694 | 137.301379 | -5.6186934 |
| YLDs (Years Lived with Disability) | Algeria | Both | Age-standardized | Osteoarthritis knee | High body-mass index | Rate | 2021 | 51.8563694 | 137.301379 | -5.6186934 |
| DALYs (Disability-Adjusted Life Years) | Tunisia | Both | Age-standardized | Osteoarthritis knee | High body-mass index | Rate | 2021 | 51.6502377 | 136.479381 | -5.845569 |
| YLDs (Years Lived with Disability) | Tunisia | Both | Age-standardized | Osteoarthritis knee | High body-mass index | Rate | 2021 | 51.6502377 | 136.479381 | -5.845569 |
| DALYs (Disability-Adjusted Life Years) | Suriname | Both | Age-standardized | Osteoarthritis knee | High body-mass index | Rate | 2021 | 51.552316 | 140.01529 | -4.8237224 |
| YLDs (Years Lived with Disability) | Suriname | Both | Age-standardized | Osteoarthritis knee | High body-mass index | Rate | 2021 | 51.552316 | 140.01529 | -4.8237224 |
| DALYs (Disability-Adjusted Life Years) | Austria | Both | Age-standardized | Osteoarthritis knee | High body-mass index | Rate | 2021 | 51.5267483 | 143.634081 | -4.7552901 |
| YLDs (Years Lived with Disability) | Austria | Both | Age-standardized | Osteoarthritis knee | High body-mass index | Rate | 2021 | 51.5267483 | 143.634081 | -4.7552901 |
| DALYs (Disability-Adjusted Life Years) | Gabon | Both | Age-standardized | Osteoarthritis knee | High body-mass index | Rate | 2021 | 51.1752752 | 138.406521 | -5.2397859 |
| YLDs (Years Lived with Disability) | Gabon | Both | Age-standardized | Osteoarthritis knee | High body-mass index | Rate | 2021 | 51.1752752 | 138.406521 | -5.2397859 |
| DALYs (Disability-Adjusted Life Years) | Andorra | Both | Age-standardized | Osteoarthritis knee | High body-mass index | Rate | 2021 | 51.1519757 | 139.112269 | -4.7020901 |
| YLDs (Years Lived with Disability) | Andorra | Both | Age-standardized | Osteoarthritis knee | High body-mass index | Rate | 2021 | 51.1519757 | 139.112269 | -4.7020901 |
| DALYs (Disability-Adjusted Life Years) | Saint Vincent and the Grenadines | Both | Age-standardized | Osteoarthritis knee | High body-mass index | Rate | 2021 | 51.0012003 | 142.870548 | -4.7233889 |
| YLDs (Years Lived with Disability) | Saint Vincent and the Grenadines | Both | Age-standardized | Osteoarthritis knee | High body-mass index | Rate | 2021 | 51.0012003 | 142.870548 | -4.7233889 |
| DALYs (Disability-Adjusted Life Years) | Mauritania | Both | Age-standardized | Osteoarthritis knee | High body-mass index | Rate | 2021 | 51.0007745 | 137.775249 | -5.0054629 |
| YLDs (Years Lived with Disability) | Mauritania | Both | Age-standardized | Osteoarthritis knee | High body-mass index | Rate | 2021 | 51.0007745 | 137.775249 | -5.0054629 |
| DALYs (Disability-Adjusted Life Years) | Cyprus | Both | Age-standardized | Osteoarthritis knee | High body-mass index | Rate | 2021 | 50.9372708 | 137.028011 | -5.1353279 |
| YLDs (Years Lived with Disability) | Cyprus | Both | Age-standardized | Osteoarthritis knee | High body-mass index | Rate | 2021 | 50.9372708 | 137.028011 | -5.1353279 |
| DALYs (Disability-Adjusted Life Years) | Belgium | Both | Age-standardized | Osteoarthritis knee | High body-mass index | Rate | 2021 | 50.8321929 | 140.34375 | -4.8788055 |
| YLDs (Years Lived with Disability) | Belgium | Both | Age-standardized | Osteoarthritis knee | High body-mass index | Rate | 2021 | 50.8321929 | 140.34375 | -4.8788055 |
| DALYs (Disability-Adjusted Life Years) | China | Both | Age-standardized | Osteoarthritis knee | High body-mass index | Rate | 2021 | 50.4858286 | 144.228951 | -4.528027 |
| YLDs (Years Lived with Disability) | China | Both | Age-standardized | Osteoarthritis knee | High body-mass index | Rate | 2021 | 50.4858286 | 144.228951 | -4.528027 |
| DALYs (Disability-Adjusted Life Years) | Iran (Islamic Republic of) | Both | Age-standardized | Osteoarthritis knee | High body-mass index | Rate | 2021 | 50.2858064 | 133.850855 | -5.3953467 |
| YLDs (Years Lived with Disability) | Iran (Islamic Republic of) | Both | Age-standardized | Osteoarthritis knee | High body-mass index | Rate | 2021 | 50.2858064 | 133.850855 | -5.3953467 |
| DALYs (Disability-Adjusted Life Years) | Vanuatu | Both | Age-standardized | Osteoarthritis knee | High body-mass index | Rate | 2021 | 50.1557287 | 132.529967 | -5.0373494 |
| YLDs (Years Lived with Disability) | Vanuatu | Both | Age-standardized | Osteoarthritis knee | High body-mass index | Rate | 2021 | 50.1557287 | 132.529967 | -5.0373494 |
| DALYs (Disability-Adjusted Life Years) | Republic of Moldova | Both | Age-standardized | Osteoarthritis knee | High body-mass index | Rate | 2021 | 49.983454 | 133.212622 | -5.440704 |
| YLDs (Years Lived with Disability) | Republic of Moldova | Both | Age-standardized | Osteoarthritis knee | High body-mass index | Rate | 2021 | 49.983454 | 133.212622 | -5.440704 |
| DALYs (Disability-Adjusted Life Years) | France | Both | Age-standardized | Osteoarthritis knee | High body-mass index | Rate | 2021 | 49.9236202 | 140.199595 | -4.4073407 |
| YLDs (Years Lived with Disability) | France | Both | Age-standardized | Osteoarthritis knee | High body-mass index | Rate | 2021 | 49.9236202 | 140.199595 | -4.4073407 |
| DALYs (Disability-Adjusted Life Years) | Solomon Islands | Both | Age-standardized | Osteoarthritis knee | High body-mass index | Rate | 2021 | 49.4351612 | 134.802057 | -5.0294737 |
| YLDs (Years Lived with Disability) | Solomon Islands | Both | Age-standardized | Osteoarthritis knee | High body-mass index | Rate | 2021 | 49.4351612 | 134.802057 | -5.0294737 |
| DALYs (Disability-Adjusted Life Years) | Latvia | Both | Age-standardized | Osteoarthritis knee | High body-mass index | Rate | 2021 | 49.2477121 | 132.172633 | -5.5093665 |
| YLDs (Years Lived with Disability) | Latvia | Both | Age-standardized | Osteoarthritis knee | High body-mass index | Rate | 2021 | 49.2477121 | 132.172633 | -5.5093665 |
| DALYs (Disability-Adjusted Life Years) | Italy | Both | Age-standardized | Osteoarthritis knee | High body-mass index | Rate | 2021 | 49.2057506 | 136.354522 | -4.6937178 |
| YLDs (Years Lived with Disability) | Italy | Both | Age-standardized | Osteoarthritis knee | High body-mass index | Rate | 2021 | 49.2057506 | 136.354522 | -4.6937178 |
| DALYs (Disability-Adjusted Life Years) | Estonia | Both | Age-standardized | Osteoarthritis knee | High body-mass index | Rate | 2021 | 48.8703638 | 129.38336 | -5.3079086 |
| YLDs (Years Lived with Disability) | Estonia | Both | Age-standardized | Osteoarthritis knee | High body-mass index | Rate | 2021 | 48.8703638 | 129.38336 | -5.3079086 |
| DALYs (Disability-Adjusted Life Years) | Sudan | Both | Age-standardized | Osteoarthritis knee | High body-mass index | Rate | 2021 | 48.738526 | 132.063927 | -5.1029698 |
| YLDs (Years Lived with Disability) | Sudan | Both | Age-standardized | Osteoarthritis knee | High body-mass index | Rate | 2021 | 48.738526 | 132.063927 | -5.1029698 |
| DALYs (Disability-Adjusted Life Years) | Belarus | Both | Age-standardized | Osteoarthritis knee | High body-mass index | Rate | 2021 | 48.3870383 | 127.622505 | -5.3646002 |
| YLDs (Years Lived with Disability) | Belarus | Both | Age-standardized | Osteoarthritis knee | High body-mass index | Rate | 2021 | 48.3870383 | 127.622505 | -5.3646002 |
| DALYs (Disability-Adjusted Life Years) | Lithuania | Both | Age-standardized | Osteoarthritis knee | High body-mass index | Rate | 2021 | 48.1956569 | 126.377598 | -5.1126975 |
| YLDs (Years Lived with Disability) | Lithuania | Both | Age-standardized | Osteoarthritis knee | High body-mass index | Rate | 2021 | 48.1956569 | 126.377598 | -5.1126975 |
| DALYs (Disability-Adjusted Life Years) | Russian Federation | Both | Age-standardized | Osteoarthritis knee | High body-mass index | Rate | 2021 | 48.1793023 | 127.246088 | -4.9815031 |
| YLDs (Years Lived with Disability) | Russian Federation | Both | Age-standardized | Osteoarthritis knee | High body-mass index | Rate | 2021 | 48.1793023 | 127.246088 | -4.9815031 |
| DALYs (Disability-Adjusted Life Years) | Denmark | Both | Age-standardized | Osteoarthritis knee | High body-mass index | Rate | 2021 | 47.9753361 | 131.545137 | -4.7323089 |
| YLDs (Years Lived with Disability) | Denmark | Both | Age-standardized | Osteoarthritis knee | High body-mass index | Rate | 2021 | 47.9753361 | 131.545137 | -4.7323089 |
| DALYs (Disability-Adjusted Life Years) | Seychelles | Both | Age-standardized | Osteoarthritis knee | High body-mass index | Rate | 2021 | 47.7335484 | 125.120697 | -5.4582938 |
| YLDs (Years Lived with Disability) | Seychelles | Both | Age-standardized | Osteoarthritis knee | High body-mass index | Rate | 2021 | 47.7335484 | 125.120697 | -5.4582938 |
| DALYs (Disability-Adjusted Life Years) | Liberia | Both | Age-standardized | Osteoarthritis knee | High body-mass index | Rate | 2021 | 47.6869852 | 131.890408 | -4.5655888 |
| YLDs (Years Lived with Disability) | Liberia | Both | Age-standardized | Osteoarthritis knee | High body-mass index | Rate | 2021 | 47.6869852 | 131.890408 | -4.5655888 |
| DALYs (Disability-Adjusted Life Years) | Montenegro | Both | Age-standardized | Osteoarthritis knee | High body-mass index | Rate | 2021 | 47.4523325 | 124.54345 | -5.0823695 |
| YLDs (Years Lived with Disability) | Montenegro | Both | Age-standardized | Osteoarthritis knee | High body-mass index | Rate | 2021 | 47.4523325 | 124.54345 | -5.0823695 |
| DALYs (Disability-Adjusted Life Years) | Hungary | Both | Age-standardized | Osteoarthritis knee | High body-mass index | Rate | 2021 | 47.336843 | 127.027199 | -4.8528899 |
| YLDs (Years Lived with Disability) | Hungary | Both | Age-standardized | Osteoarthritis knee | High body-mass index | Rate | 2021 | 47.336843 | 127.027199 | -4.8528899 |
| DALYs (Disability-Adjusted Life Years) | Morocco | Both | Age-standardized | Osteoarthritis knee | High body-mass index | Rate | 2021 | 47.2210697 | 124.560817 | -4.9895414 |
| YLDs (Years Lived with Disability) | Morocco | Both | Age-standardized | Osteoarthritis knee | High body-mass index | Rate | 2021 | 47.2210697 | 124.560817 | -4.9895414 |
| DALYs (Disability-Adjusted Life Years) | Norway | Both | Age-standardized | Osteoarthritis knee | High body-mass index | Rate | 2021 | 47.142221 | 131.203141 | -4.3663185 |
| YLDs (Years Lived with Disability) | Norway | Both | Age-standardized | Osteoarthritis knee | High body-mass index | Rate | 2021 | 47.142221 | 131.203141 | -4.3663185 |
| DALYs (Disability-Adjusted Life Years) | Botswana | Both | Age-standardized | Osteoarthritis knee | High body-mass index | Rate | 2021 | 46.523574 | 132.38871 | -3.8150219 |
| YLDs (Years Lived with Disability) | Botswana | Both | Age-standardized | Osteoarthritis knee | High body-mass index | Rate | 2021 | 46.523574 | 132.38871 | -3.8150219 |
| DALYs (Disability-Adjusted Life Years) | Slovakia | Both | Age-standardized | Osteoarthritis knee | High body-mass index | Rate | 2021 | 46.3548581 | 123.266198 | -4.7345055 |
| YLDs (Years Lived with Disability) | Slovakia | Both | Age-standardized | Osteoarthritis knee | High body-mass index | Rate | 2021 | 46.3548581 | 123.266198 | -4.7345055 |
| DALYs (Disability-Adjusted Life Years) | Ukraine | Both | Age-standardized | Osteoarthritis knee | High body-mass index | Rate | 2021 | 46.2666059 | 123.146501 | -4.9134092 |
| YLDs (Years Lived with Disability) | Ukraine | Both | Age-standardized | Osteoarthritis knee | High body-mass index | Rate | 2021 | 46.2666059 | 123.146501 | -4.9134092 |
| DALYs (Disability-Adjusted Life Years) | Switzerland | Both | Age-standardized | Osteoarthritis knee | High body-mass index | Rate | 2021 | 46.1254495 | 127.58152 | -4.0319937 |
| YLDs (Years Lived with Disability) | Switzerland | Both | Age-standardized | Osteoarthritis knee | High body-mass index | Rate | 2021 | 46.1254495 | 127.58152 | -4.0319937 |
| DALYs (Disability-Adjusted Life Years) | Sao Tome and Principe | Both | Age-standardized | Osteoarthritis knee | High body-mass index | Rate | 2021 | 46.1092374 | 128.673039 | -4.4300775 |
| YLDs (Years Lived with Disability) | Sao Tome and Principe | Both | Age-standardized | Osteoarthritis knee | High body-mass index | Rate | 2021 | 46.1092374 | 128.673039 | -4.4300775 |
| DALYs (Disability-Adjusted Life Years) | Lesotho | Both | Age-standardized | Osteoarthritis knee | High body-mass index | Rate | 2021 | 45.9149741 | 124.954723 | -4.533573 |
| YLDs (Years Lived with Disability) | Lesotho | Both | Age-standardized | Osteoarthritis knee | High body-mass index | Rate | 2021 | 45.9149741 | 124.954723 | -4.533573 |
| DALYs (Disability-Adjusted Life Years) | Serbia | Both | Age-standardized | Osteoarthritis knee | High body-mass index | Rate | 2021 | 45.8594085 | 120.627535 | -4.9587611 |
| YLDs (Years Lived with Disability) | Serbia | Both | Age-standardized | Osteoarthritis knee | High body-mass index | Rate | 2021 | 45.8594085 | 120.627535 | -4.9587611 |
| DALYs (Disability-Adjusted Life Years) | Czechia | Both | Age-standardized | Osteoarthritis knee | High body-mass index | Rate | 2021 | 45.6433958 | 120.537354 | -5.0584946 |
| YLDs (Years Lived with Disability) | Czechia | Both | Age-standardized | Osteoarthritis knee | High body-mass index | Rate | 2021 | 45.6433958 | 120.537354 | -5.0584946 |
| DALYs (Disability-Adjusted Life Years) | Slovenia | Both | Age-standardized | Osteoarthritis knee | High body-mass index | Rate | 2021 | 45.5214851 | 120.243062 | -4.7008866 |
| YLDs (Years Lived with Disability) | Slovenia | Both | Age-standardized | Osteoarthritis knee | High body-mass index | Rate | 2021 | 45.5214851 | 120.243062 | -4.7008866 |
| DALYs (Disability-Adjusted Life Years) | Croatia | Both | Age-standardized | Osteoarthritis knee | High body-mass index | Rate | 2021 | 45.3374345 | 122.613415 | -4.9144259 |
| YLDs (Years Lived with Disability) | Croatia | Both | Age-standardized | Osteoarthritis knee | High body-mass index | Rate | 2021 | 45.3374345 | 122.613415 | -4.9144259 |
| DALYs (Disability-Adjusted Life Years) | Equatorial Guinea | Both | Age-standardized | Osteoarthritis knee | High body-mass index | Rate | 2021 | 45.3178488 | 126.665136 | -4.0425274 |
| YLDs (Years Lived with Disability) | Equatorial Guinea | Both | Age-standardized | Osteoarthritis knee | High body-mass index | Rate | 2021 | 45.3178488 | 126.665136 | -4.0425274 |
| DALYs (Disability-Adjusted Life Years) | Cabo Verde | Both | Age-standardized | Osteoarthritis knee | High body-mass index | Rate | 2021 | 44.8349146 | 125.942615 | -4.1295121 |
| YLDs (Years Lived with Disability) | Cabo Verde | Both | Age-standardized | Osteoarthritis knee | High body-mass index | Rate | 2021 | 44.8349146 | 125.942615 | -4.1295121 |
| DALYs (Disability-Adjusted Life Years) | Japan | Both | Age-standardized | Osteoarthritis knee | High body-mass index | Rate | 2021 | 44.273879 | 131.440193 | -3.5626141 |
| YLDs (Years Lived with Disability) | Japan | Both | Age-standardized | Osteoarthritis knee | High body-mass index | Rate | 2021 | 44.273879 | 131.440193 | -3.5626141 |
| DALYs (Disability-Adjusted Life Years) | Namibia | Both | Age-standardized | Osteoarthritis knee | High body-mass index | Rate | 2021 | 44.0258989 | 125.534072 | -3.5250089 |
| YLDs (Years Lived with Disability) | Namibia | Both | Age-standardized | Osteoarthritis knee | High body-mass index | Rate | 2021 | 44.0258989 | 125.534072 | -3.5250089 |
| DALYs (Disability-Adjusted Life Years) | Romania | Both | Age-standardized | Osteoarthritis knee | High body-mass index | Rate | 2021 | 43.5062646 | 116.516251 | -4.3011879 |
| YLDs (Years Lived with Disability) | Romania | Both | Age-standardized | Osteoarthritis knee | High body-mass index | Rate | 2021 | 43.5062646 | 116.516251 | -4.3011879 |
| DALYs (Disability-Adjusted Life Years) | North Macedonia | Both | Age-standardized | Osteoarthritis knee | High body-mass index | Rate | 2021 | 43.5009171 | 120.074238 | -4.4770568 |
| YLDs (Years Lived with Disability) | North Macedonia | Both | Age-standardized | Osteoarthritis knee | High body-mass index | Rate | 2021 | 43.5009171 | 120.074238 | -4.4770568 |
| DALYs (Disability-Adjusted Life Years) | Bulgaria | Both | Age-standardized | Osteoarthritis knee | High body-mass index | Rate | 2021 | 43.3229879 | 117.699675 | -4.3110583 |
| YLDs (Years Lived with Disability) | Bulgaria | Both | Age-standardized | Osteoarthritis knee | High body-mass index | Rate | 2021 | 43.3229879 | 117.699675 | -4.3110583 |
| DALYs (Disability-Adjusted Life Years) | Poland | Both | Age-standardized | Osteoarthritis knee | High body-mass index | Rate | 2021 | 43.0881291 | 115.817725 | -4.5603926 |
| YLDs (Years Lived with Disability) | Poland | Both | Age-standardized | Osteoarthritis knee | High body-mass index | Rate | 2021 | 43.0881291 | 115.817725 | -4.5603926 |
| DALYs (Disability-Adjusted Life Years) | Ghana | Both | Age-standardized | Osteoarthritis knee | High body-mass index | Rate | 2021 | 41.7856735 | 118.321259 | -3.4018521 |
| YLDs (Years Lived with Disability) | Ghana | Both | Age-standardized | Osteoarthritis knee | High body-mass index | Rate | 2021 | 41.7856735 | 118.321259 | -3.4018521 |
| DALYs (Disability-Adjusted Life Years) | Albania | Both | Age-standardized | Osteoarthritis knee | High body-mass index | Rate | 2021 | 41.5629349 | 108.330608 | -4.4539596 |
| YLDs (Years Lived with Disability) | Albania | Both | Age-standardized | Osteoarthritis knee | High body-mass index | Rate | 2021 | 41.5629349 | 108.330608 | -4.4539596 |
| DALYs (Disability-Adjusted Life Years) | Bosnia and Herzegovina | Both | Age-standardized | Osteoarthritis knee | High body-mass index | Rate | 2021 | 41.4695613 | 112.236073 | -4.4132108 |
| YLDs (Years Lived with Disability) | Bosnia and Herzegovina | Both | Age-standardized | Osteoarthritis knee | High body-mass index | Rate | 2021 | 41.4695613 | 112.236073 | -4.4132108 |
| DALYs (Disability-Adjusted Life Years) | Bhutan | Both | Age-standardized | Osteoarthritis knee | High body-mass index | Rate | 2021 | 41.2220611 | 114.552777 | -3.7615152 |
| YLDs (Years Lived with Disability) | Bhutan | Both | Age-standardized | Osteoarthritis knee | High body-mass index | Rate | 2021 | 41.2220611 | 114.552777 | -3.7615152 |
| DALYs (Disability-Adjusted Life Years) | Mauritius | Both | Age-standardized | Osteoarthritis knee | High body-mass index | Rate | 2021 | 41.1451367 | 109.741812 | -3.8186458 |
| YLDs (Years Lived with Disability) | Mauritius | Both | Age-standardized | Osteoarthritis knee | High body-mass index | Rate | 2021 | 41.1451367 | 109.741812 | -3.8186458 |
| DALYs (Disability-Adjusted Life Years) | Nigeria | Both | Age-standardized | Osteoarthritis knee | High body-mass index | Rate | 2021 | 41.1130928 | 115.135609 | -3.6007266 |
| YLDs (Years Lived with Disability) | Nigeria | Both | Age-standardized | Osteoarthritis knee | High body-mass index | Rate | 2021 | 41.1130928 | 115.135609 | -3.6007266 |
| DALYs (Disability-Adjusted Life Years) | Sweden | Both | Age-standardized | Osteoarthritis knee | High body-mass index | Rate | 2021 | 40.9106458 | 113.527179 | -4.1036695 |
| YLDs (Years Lived with Disability) | Sweden | Both | Age-standardized | Osteoarthritis knee | High body-mass index | Rate | 2021 | 40.9106458 | 113.527179 | -4.1036695 |
| DALYs (Disability-Adjusted Life Years) | Benin | Both | Age-standardized | Osteoarthritis knee | High body-mass index | Rate | 2021 | 40.4869714 | 115.339407 | -3.3182184 |
| YLDs (Years Lived with Disability) | Benin | Both | Age-standardized | Osteoarthritis knee | High body-mass index | Rate | 2021 | 40.4869714 | 115.339407 | -3.3182184 |
| DALYs (Disability-Adjusted Life Years) | C么te d'Ivoire | Both | Age-standardized | Osteoarthritis knee | High body-mass index | Rate | 2021 | 40.3767409 | 114.939113 | -3.3858574 |
| YLDs (Years Lived with Disability) | C么te d'Ivoire | Both | Age-standardized | Osteoarthritis knee | High body-mass index | Rate | 2021 | 40.3767409 | 114.939113 | -3.3858574 |
| DALYs (Disability-Adjusted Life Years) | Gambia | Both | Age-standardized | Osteoarthritis knee | High body-mass index | Rate | 2021 | 39.9191929 | 112.615519 | -3.5435306 |
| YLDs (Years Lived with Disability) | Gambia | Both | Age-standardized | Osteoarthritis knee | High body-mass index | Rate | 2021 | 39.9191929 | 112.615519 | -3.5435306 |
| DALYs (Disability-Adjusted Life Years) | Greenland | Both | Age-standardized | Osteoarthritis knee | High body-mass index | Rate | 2021 | 39.6090334 | 106.583354 | -3.9446885 |
| YLDs (Years Lived with Disability) | Greenland | Both | Age-standardized | Osteoarthritis knee | High body-mass index | Rate | 2021 | 39.6090334 | 106.583354 | -3.9446885 |
| DALYs (Disability-Adjusted Life Years) | Zimbabwe | Both | Age-standardized | Osteoarthritis knee | High body-mass index | Rate | 2021 | 39.3235734 | 108.290775 | -3.3527879 |
| YLDs (Years Lived with Disability) | Zimbabwe | Both | Age-standardized | Osteoarthritis knee | High body-mass index | Rate | 2021 | 39.3235734 | 108.290775 | -3.3527879 |
| DALYs (Disability-Adjusted Life Years) | Thailand | Both | Age-standardized | Osteoarthritis knee | High body-mass index | Rate | 2021 | 38.7452102 | 111.089526 | -3.4009501 |
| YLDs (Years Lived with Disability) | Thailand | Both | Age-standardized | Osteoarthritis knee | High body-mass index | Rate | 2021 | 38.7452102 | 111.089526 | -3.4009501 |
| DALYs (Disability-Adjusted Life Years) | Malaysia | Both | Age-standardized | Osteoarthritis knee | High body-mass index | Rate | 2021 | 38.4327166 | 104.576743 | -3.5246073 |
| YLDs (Years Lived with Disability) | Malaysia | Both | Age-standardized | Osteoarthritis knee | High body-mass index | Rate | 2021 | 38.4327166 | 104.576743 | -3.5246073 |
| DALYs (Disability-Adjusted Life Years) | Kazakhstan | Both | Age-standardized | Osteoarthritis knee | High body-mass index | Rate | 2021 | 37.8454131 | 101.193743 | -3.8148738 |
| YLDs (Years Lived with Disability) | Kazakhstan | Both | Age-standardized | Osteoarthritis knee | High body-mass index | Rate | 2021 | 37.8454131 | 101.193743 | -3.8148738 |
| DALYs (Disability-Adjusted Life Years) | Papua New Guinea | Both | Age-standardized | Osteoarthritis knee | High body-mass index | Rate | 2021 | 37.668305 | 105.048637 | -3.5219481 |
| YLDs (Years Lived with Disability) | Papua New Guinea | Both | Age-standardized | Osteoarthritis knee | High body-mass index | Rate | 2021 | 37.668305 | 105.048637 | -3.5219481 |
| DALYs (Disability-Adjusted Life Years) | Congo | Both | Age-standardized | Osteoarthritis knee | High body-mass index | Rate | 2021 | 37.5708404 | 107.192524 | -3.3087925 |
| YLDs (Years Lived with Disability) | Congo | Both | Age-standardized | Osteoarthritis knee | High body-mass index | Rate | 2021 | 37.5708404 | 107.192524 | -3.3087925 |
| DALYs (Disability-Adjusted Life Years) | Azerbaijan | Both | Age-standardized | Osteoarthritis knee | High body-mass index | Rate | 2021 | 37.5706768 | 100.620281 | -3.8678807 |
| YLDs (Years Lived with Disability) | Azerbaijan | Both | Age-standardized | Osteoarthritis knee | High body-mass index | Rate | 2021 | 37.5706768 | 100.620281 | -3.8678807 |
| DALYs (Disability-Adjusted Life Years) | Canada | Both | Age-standardized | Osteoarthritis knee | High body-mass index | Rate | 2021 | 37.3329334 | 99.7888003 | -3.6029902 |
| YLDs (Years Lived with Disability) | Canada | Both | Age-standardized | Osteoarthritis knee | High body-mass index | Rate | 2021 | 37.3329334 | 99.7888003 | -3.6029902 |
| DALYs (Disability-Adjusted Life Years) | Senegal | Both | Age-standardized | Osteoarthritis knee | High body-mass index | Rate | 2021 | 37.2870532 | 105.393889 | -3.1457658 |
| YLDs (Years Lived with Disability) | Senegal | Both | Age-standardized | Osteoarthritis knee | High body-mass index | Rate | 2021 | 37.2870532 | 105.393889 | -3.1457658 |
| DALYs (Disability-Adjusted Life Years) | Togo | Both | Age-standardized | Osteoarthritis knee | High body-mass index | Rate | 2021 | 37.1298007 | 105.967216 | -3.1428189 |
| YLDs (Years Lived with Disability) | Togo | Both | Age-standardized | Osteoarthritis knee | High body-mass index | Rate | 2021 | 37.1298007 | 105.967216 | -3.1428189 |
| DALYs (Disability-Adjusted Life Years) | Armenia | Both | Age-standardized | Osteoarthritis knee | High body-mass index | Rate | 2021 | 36.8163749 | 98.6035966 | -3.9415064 |
| YLDs (Years Lived with Disability) | Armenia | Both | Age-standardized | Osteoarthritis knee | High body-mass index | Rate | 2021 | 36.8163749 | 98.6035966 | -3.9415064 |
| DALYs (Disability-Adjusted Life Years) | Afghanistan | Both | Age-standardized | Osteoarthritis knee | High body-mass index | Rate | 2021 | 36.8079921 | 104.164368 | -3.1429826 |
| YLDs (Years Lived with Disability) | Afghanistan | Both | Age-standardized | Osteoarthritis knee | High body-mass index | Rate | 2021 | 36.8079921 | 104.164368 | -3.1429826 |
| DALYs (Disability-Adjusted Life Years) | Uzbekistan | Both | Age-standardized | Osteoarthritis knee | High body-mass index | Rate | 2021 | 35.9659595 | 95.2010184 | -3.9126375 |
| YLDs (Years Lived with Disability) | Uzbekistan | Both | Age-standardized | Osteoarthritis knee | High body-mass index | Rate | 2021 | 35.9659595 | 95.2010184 | -3.9126375 |
| DALYs (Disability-Adjusted Life Years) | United Republic of Tanzania | Both | Age-standardized | Osteoarthritis knee | High body-mass index | Rate | 2021 | 35.9354112 | 100.128286 | -2.8997633 |
| YLDs (Years Lived with Disability) | United Republic of Tanzania | Both | Age-standardized | Osteoarthritis knee | High body-mass index | Rate | 2021 | 35.9354112 | 100.128286 | -2.8997633 |
| DALYs (Disability-Adjusted Life Years) | Yemen | Both | Age-standardized | Osteoarthritis knee | High body-mass index | Rate | 2021 | 35.8047081 | 101.551899 | -2.9359856 |
| YLDs (Years Lived with Disability) | Yemen | Both | Age-standardized | Osteoarthritis knee | High body-mass index | Rate | 2021 | 35.8047081 | 101.551899 | -2.9359856 |
| DALYs (Disability-Adjusted Life Years) | Kyrgyzstan | Both | Age-standardized | Osteoarthritis knee | High body-mass index | Rate | 2021 | 35.7631206 | 93.9635269 | -3.7528233 |
| YLDs (Years Lived with Disability) | Kyrgyzstan | Both | Age-standardized | Osteoarthritis knee | High body-mass index | Rate | 2021 | 35.7631206 | 93.9635269 | -3.7528233 |
| DALYs (Disability-Adjusted Life Years) | Georgia | Both | Age-standardized | Osteoarthritis knee | High body-mass index | Rate | 2021 | 34.990576 | 94.2453592 | -3.5311226 |
| YLDs (Years Lived with Disability) | Georgia | Both | Age-standardized | Osteoarthritis knee | High body-mass index | Rate | 2021 | 34.990576 | 94.2453592 | -3.5311226 |
| DALYs (Disability-Adjusted Life Years) | Turkmenistan | Both | Age-standardized | Osteoarthritis knee | High body-mass index | Rate | 2021 | 34.7746104 | 93.6673561 | -3.6831148 |
| YLDs (Years Lived with Disability) | Turkmenistan | Both | Age-standardized | Osteoarthritis knee | High body-mass index | Rate | 2021 | 34.7746104 | 93.6673561 | -3.6831148 |
| DALYs (Disability-Adjusted Life Years) | Maldives | Both | Age-standardized | Osteoarthritis knee | High body-mass index | Rate | 2021 | 34.2100901 | 95.1355093 | -3.1302641 |
| YLDs (Years Lived with Disability) | Maldives | Both | Age-standardized | Osteoarthritis knee | High body-mass index | Rate | 2021 | 34.2100901 | 95.1355093 | -3.1302641 |
| DALYs (Disability-Adjusted Life Years) | Zambia | Both | Age-standardized | Osteoarthritis knee | High body-mass index | Rate | 2021 | 33.9030693 | 97.2495102 | -2.9167325 |
| YLDs (Years Lived with Disability) | Zambia | Both | Age-standardized | Osteoarthritis knee | High body-mass index | Rate | 2021 | 33.9030693 | 97.2495102 | -2.9167325 |
| DALYs (Disability-Adjusted Life Years) | Guinea-Bissau | Both | Age-standardized | Osteoarthritis knee | High body-mass index | Rate | 2021 | 33.6470957 | 98.0267018 | -2.7496047 |
| YLDs (Years Lived with Disability) | Guinea-Bissau | Both | Age-standardized | Osteoarthritis knee | High body-mass index | Rate | 2021 | 33.6470957 | 98.0267018 | -2.7496047 |
| DALYs (Disability-Adjusted Life Years) | Comoros | Both | Age-standardized | Osteoarthritis knee | High body-mass index | Rate | 2021 | 33.6285829 | 97.2325293 | -2.7234481 |
| YLDs (Years Lived with Disability) | Comoros | Both | Age-standardized | Osteoarthritis knee | High body-mass index | Rate | 2021 | 33.6285829 | 97.2325293 | -2.7234481 |
| DALYs (Disability-Adjusted Life Years) | Democratic People's Republic of Korea | Both | Age-standardized | Osteoarthritis knee | High body-mass index | Rate | 2021 | 32.7230796 | 93.9376748 | -2.771216 |
| YLDs (Years Lived with Disability) | Democratic People's Republic of Korea | Both | Age-standardized | Osteoarthritis knee | High body-mass index | Rate | 2021 | 32.7230796 | 93.9376748 | -2.771216 |
| DALYs (Disability-Adjusted Life Years) | Sierra Leone | Both | Age-standardized | Osteoarthritis knee | High body-mass index | Rate | 2021 | 32.5521146 | 95.2792044 | -2.5807475 |
| YLDs (Years Lived with Disability) | Sierra Leone | Both | Age-standardized | Osteoarthritis knee | High body-mass index | Rate | 2021 | 32.5521146 | 95.2792044 | -2.5807475 |
| DALYs (Disability-Adjusted Life Years) | Kenya | Both | Age-standardized | Osteoarthritis knee | High body-mass index | Rate | 2021 | 32.186117 | 93.0594894 | -2.6541622 |
| YLDs (Years Lived with Disability) | Kenya | Both | Age-standardized | Osteoarthritis knee | High body-mass index | Rate | 2021 | 32.186117 | 93.0594894 | -2.6541622 |
| DALYs (Disability-Adjusted Life Years) | Guinea | Both | Age-standardized | Osteoarthritis knee | High body-mass index | Rate | 2021 | 31.5867735 | 91.5907888 | -2.5317333 |
| YLDs (Years Lived with Disability) | Guinea | Both | Age-standardized | Osteoarthritis knee | High body-mass index | Rate | 2021 | 31.5867735 | 91.5907888 | -2.5317333 |
| DALYs (Disability-Adjusted Life Years) | Tajikistan | Both | Age-standardized | Osteoarthritis knee | High body-mass index | Rate | 2021 | 31.2526984 | 82.9662742 | -3.2578643 |
| YLDs (Years Lived with Disability) | Tajikistan | Both | Age-standardized | Osteoarthritis knee | High body-mass index | Rate | 2021 | 31.2526984 | 82.9662742 | -3.2578643 |
| DALYs (Disability-Adjusted Life Years) | Mongolia | Both | Age-standardized | Osteoarthritis knee | High body-mass index | Rate | 2021 | 31.0005661 | 85.3937332 | -3.0744687 |
| YLDs (Years Lived with Disability) | Mongolia | Both | Age-standardized | Osteoarthritis knee | High body-mass index | Rate | 2021 | 31.0005661 | 85.3937332 | -3.0744687 |
| DALYs (Disability-Adjusted Life Years) | Democratic Republic of the Congo | Both | Age-standardized | Osteoarthritis knee | High body-mass index | Rate | 2021 | 30.7640932 | 91.5485187 | -2.2817397 |
| YLDs (Years Lived with Disability) | Democratic Republic of the Congo | Both | Age-standardized | Osteoarthritis knee | High body-mass index | Rate | 2021 | 30.7640932 | 91.5485187 | -2.2817397 |
| DALYs (Disability-Adjusted Life Years) | Pakistan | Both | Age-standardized | Osteoarthritis knee | High body-mass index | Rate | 2021 | 30.1714311 | 89.4360152 | -2.5660875 |
| YLDs (Years Lived with Disability) | Pakistan | Both | Age-standardized | Osteoarthritis knee | High body-mass index | Rate | 2021 | 30.1714311 | 89.4360152 | -2.5660875 |
| DALYs (Disability-Adjusted Life Years) | Angola | Both | Age-standardized | Osteoarthritis knee | High body-mass index | Rate | 2021 | 29.5987241 | 87.1598448 | -2.2635749 |
| YLDs (Years Lived with Disability) | Angola | Both | Age-standardized | Osteoarthritis knee | High body-mass index | Rate | 2021 | 29.5987241 | 87.1598448 | -2.2635749 |
| DALYs (Disability-Adjusted Life Years) | Haiti | Both | Age-standardized | Osteoarthritis knee | High body-mass index | Rate | 2021 | 29.5574481 | 85.8073593 | -2.3618832 |
| YLDs (Years Lived with Disability) | Haiti | Both | Age-standardized | Osteoarthritis knee | High body-mass index | Rate | 2021 | 29.5574481 | 85.8073593 | -2.3618832 |
| DALYs (Disability-Adjusted Life Years) | Sri Lanka | Both | Age-standardized | Osteoarthritis knee | High body-mass index | Rate | 2021 | 29.2332352 | 85.0730456 | -2.1966728 |
| YLDs (Years Lived with Disability) | Sri Lanka | Both | Age-standardized | Osteoarthritis knee | High body-mass index | Rate | 2021 | 29.2332352 | 85.0730456 | -2.1966728 |
| DALYs (Disability-Adjusted Life Years) | Malawi | Both | Age-standardized | Osteoarthritis knee | High body-mass index | Rate | 2021 | 27.1415284 | 80.3435163 | -2.125272 |
| YLDs (Years Lived with Disability) | Malawi | Both | Age-standardized | Osteoarthritis knee | High body-mass index | Rate | 2021 | 27.1415284 | 80.3435163 | -2.125272 |
| DALYs (Disability-Adjusted Life Years) | Mali | Both | Age-standardized | Osteoarthritis knee | High body-mass index | Rate | 2021 | 27.0986451 | 79.2593279 | -2.0521741 |
| YLDs (Years Lived with Disability) | Mali | Both | Age-standardized | Osteoarthritis knee | High body-mass index | Rate | 2021 | 27.0986451 | 79.2593279 | -2.0521741 |
| DALYs (Disability-Adjusted Life Years) | Mozambique | Both | Age-standardized | Osteoarthritis knee | High body-mass index | Rate | 2021 | 27.0774599 | 78.2936553 | -1.9624129 |
| YLDs (Years Lived with Disability) | Mozambique | Both | Age-standardized | Osteoarthritis knee | High body-mass index | Rate | 2021 | 27.0774599 | 78.2936553 | -1.9624129 |
| DALYs (Disability-Adjusted Life Years) | Uganda | Both | Age-standardized | Osteoarthritis knee | High body-mass index | Rate | 2021 | 26.9653873 | 79.0804527 | -2.130563 |
| YLDs (Years Lived with Disability) | Uganda | Both | Age-standardized | Osteoarthritis knee | High body-mass index | Rate | 2021 | 26.9653873 | 79.0804527 | -2.130563 |
| DALYs (Disability-Adjusted Life Years) | Chad | Both | Age-standardized | Osteoarthritis knee | High body-mass index | Rate | 2021 | 26.0182153 | 74.8166917 | -2.0293066 |
| YLDs (Years Lived with Disability) | Chad | Both | Age-standardized | Osteoarthritis knee | High body-mass index | Rate | 2021 | 26.0182153 | 74.8166917 | -2.0293066 |
| DALYs (Disability-Adjusted Life Years) | India | Both | Age-standardized | Osteoarthritis knee | High body-mass index | Rate | 2021 | 25.8033923 | 75.0653199 | -2.0363028 |
| YLDs (Years Lived with Disability) | India | Both | Age-standardized | Osteoarthritis knee | High body-mass index | Rate | 2021 | 25.8033923 | 75.0653199 | -2.0363028 |
| DALYs (Disability-Adjusted Life Years) | Niger | Both | Age-standardized | Osteoarthritis knee | High body-mass index | Rate | 2021 | 25.7123902 | 74.4128794 | -1.8989067 |
| YLDs (Years Lived with Disability) | Niger | Both | Age-standardized | Osteoarthritis knee | High body-mass index | Rate | 2021 | 25.7123902 | 74.4128794 | -1.8989067 |
| DALYs (Disability-Adjusted Life Years) | Philippines | Both | Age-standardized | Osteoarthritis knee | High body-mass index | Rate | 2021 | 25.2833361 | 73.2933501 | -1.8955788 |
| YLDs (Years Lived with Disability) | Philippines | Both | Age-standardized | Osteoarthritis knee | High body-mass index | Rate | 2021 | 25.2833361 | 73.2933501 | -1.8955788 |
| DALYs (Disability-Adjusted Life Years) | Central African Republic | Both | Age-standardized | Osteoarthritis knee | High body-mass index | Rate | 2021 | 25.1845188 | 70.5701706 | -1.8750684 |
| YLDs (Years Lived with Disability) | Central African Republic | Both | Age-standardized | Osteoarthritis knee | High body-mass index | Rate | 2021 | 25.1845188 | 70.5701706 | -1.8750684 |
| DALYs (Disability-Adjusted Life Years) | Nepal | Both | Age-standardized | Osteoarthritis knee | High body-mass index | Rate | 2021 | 24.6006624 | 70.0746732 | -1.8904881 |
| YLDs (Years Lived with Disability) | Nepal | Both | Age-standardized | Osteoarthritis knee | High body-mass index | Rate | 2021 | 24.6006624 | 70.0746732 | -1.8904881 |
| DALYs (Disability-Adjusted Life Years) | Rwanda | Both | Age-standardized | Osteoarthritis knee | High body-mass index | Rate | 2021 | 24.411716 | 74.603331 | -2.0342916 |
| YLDs (Years Lived with Disability) | Rwanda | Both | Age-standardized | Osteoarthritis knee | High body-mass index | Rate | 2021 | 24.411716 | 74.603331 | -2.0342916 |
| DALYs (Disability-Adjusted Life Years) | Indonesia | Both | Age-standardized | Osteoarthritis knee | High body-mass index | Rate | 2021 | 23.5245247 | 68.0629699 | -1.8400728 |
| YLDs (Years Lived with Disability) | Indonesia | Both | Age-standardized | Osteoarthritis knee | High body-mass index | Rate | 2021 | 23.5245247 | 68.0629699 | -1.8400728 |
| DALYs (Disability-Adjusted Life Years) | Somalia | Both | Age-standardized | Osteoarthritis knee | High body-mass index | Rate | 2021 | 23.216284 | 65.3316937 | -1.7944068 |
| YLDs (Years Lived with Disability) | Somalia | Both | Age-standardized | Osteoarthritis knee | High body-mass index | Rate | 2021 | 23.216284 | 65.3316937 | -1.7944068 |
| DALYs (Disability-Adjusted Life Years) | Madagascar | Both | Age-standardized | Osteoarthritis knee | High body-mass index | Rate | 2021 | 22.681041 | 64.5080992 | -1.633486 |
| YLDs (Years Lived with Disability) | Madagascar | Both | Age-standardized | Osteoarthritis knee | High body-mass index | Rate | 2021 | 22.681041 | 64.5080992 | -1.633486 |
| DALYs (Disability-Adjusted Life Years) | Myanmar | Both | Age-standardized | Osteoarthritis knee | High body-mass index | Rate | 2021 | 22.6742123 | 64.4631562 | -1.7854414 |
| YLDs (Years Lived with Disability) | Myanmar | Both | Age-standardized | Osteoarthritis knee | High body-mass index | Rate | 2021 | 22.6742123 | 64.4631562 | -1.7854414 |
| DALYs (Disability-Adjusted Life Years) | Lao People's Democratic Republic | Both | Age-standardized | Osteoarthritis knee | High body-mass index | Rate | 2021 | 22.5799248 | 66.232567 | -1.5637871 |
| YLDs (Years Lived with Disability) | Lao People's Democratic Republic | Both | Age-standardized | Osteoarthritis knee | High body-mass index | Rate | 2021 | 22.5799248 | 66.232567 | -1.5637871 |
| DALYs (Disability-Adjusted Life Years) | Bangladesh | Both | Age-standardized | Osteoarthritis knee | High body-mass index | Rate | 2021 | 22.0041481 | 64.7094495 | -1.7225288 |
| YLDs (Years Lived with Disability) | Bangladesh | Both | Age-standardized | Osteoarthritis knee | High body-mass index | Rate | 2021 | 22.0041481 | 64.7094495 | -1.7225288 |
| DALYs (Disability-Adjusted Life Years) | Burkina Faso | Both | Age-standardized | Osteoarthritis knee | High body-mass index | Rate | 2021 | 21.7831965 | 65.6263526 | -1.7625802 |
| YLDs (Years Lived with Disability) | Burkina Faso | Both | Age-standardized | Osteoarthritis knee | High body-mass index | Rate | 2021 | 21.7831965 | 65.6263526 | -1.7625802 |
| DALYs (Disability-Adjusted Life Years) | Djibouti | Both | Age-standardized | Osteoarthritis knee | High body-mass index | Rate | 2021 | 20.581381 | 60.8508026 | -1.5315482 |
| YLDs (Years Lived with Disability) | Djibouti | Both | Age-standardized | Osteoarthritis knee | High body-mass index | Rate | 2021 | 20.581381 | 60.8508026 | -1.5315482 |
| DALYs (Disability-Adjusted Life Years) | Ethiopia | Both | Age-standardized | Osteoarthritis knee | High body-mass index | Rate | 2021 | 19.1337004 | 57.5844576 | -1.467639 |
| YLDs (Years Lived with Disability) | Ethiopia | Both | Age-standardized | Osteoarthritis knee | High body-mass index | Rate | 2021 | 19.1337004 | 57.5844576 | -1.467639 |
| DALYs (Disability-Adjusted Life Years) | Cambodia | Both | Age-standardized | Osteoarthritis knee | High body-mass index | Rate | 2021 | 18.2741664 | 55.1780056 | -1.3821291 |
| YLDs (Years Lived with Disability) | Cambodia | Both | Age-standardized | Osteoarthritis knee | High body-mass index | Rate | 2021 | 18.2741664 | 55.1780056 | -1.3821291 |
| DALYs (Disability-Adjusted Life Years) | Eritrea | Both | Age-standardized | Osteoarthritis knee | High body-mass index | Rate | 2021 | 17.2361683 | 52.1216968 | -1.2434398 |
| YLDs (Years Lived with Disability) | Eritrea | Both | Age-standardized | Osteoarthritis knee | High body-mass index | Rate | 2021 | 17.2361683 | 52.1216968 | -1.2434398 |
| DALYs (Disability-Adjusted Life Years) | South Sudan | Both | Age-standardized | Osteoarthritis knee | High body-mass index | Rate | 2021 | 17.118386 | 51.349043 | -1.2936355 |
| YLDs (Years Lived with Disability) | South Sudan | Both | Age-standardized | Osteoarthritis knee | High body-mass index | Rate | 2021 | 17.118386 | 51.349043 | -1.2936355 |
| DALYs (Disability-Adjusted Life Years) | Burundi | Both | Age-standardized | Osteoarthritis knee | High body-mass index | Rate | 2021 | 16.9653168 | 51.811576 | -1.2319123 |
| YLDs (Years Lived with Disability) | Burundi | Both | Age-standardized | Osteoarthritis knee | High body-mass index | Rate | 2021 | 16.9653168 | 51.811576 | -1.2319123 |
| DALYs (Disability-Adjusted Life Years) | Viet Nam | Both | Age-standardized | Osteoarthritis knee | High body-mass index | Rate | 2021 | 16.288187 | 49.1991348 | -1.1394476 |
| YLDs (Years Lived with Disability) | Viet Nam | Both | Age-standardized | Osteoarthritis knee | High body-mass index | Rate | 2021 | 16.288187 | 49.1991348 | -1.1394476 |
| DALYs (Disability-Adjusted Life Years) | Timor-Leste | Both | Age-standardized | Osteoarthritis knee | High body-mass index | Rate | 2021 | 12.7570576 | 39.3654477 | -0.8483256 |
| YLDs (Years Lived with Disability) | Timor-Leste | Both | Age-standardized | Osteoarthritis knee | High body-mass index | Rate | 2021 | 12.7570576 | 39.3654477 | -0.8483256 |

Appendix 9: The EAPC of age-standardized DALY rates and age-standardized YLD rates for knee osteoarthritis due to high BMI across 204 countries from 1990 to 2021

| location | EAPC | LCI | UCI | EAPC_CI |
| --- | --- | --- | --- | --- |
| Bangladesh | 2.95 | 2.36 | 3.55 | 2.95 (2.36 to 3.55) |
| Viet Nam | 2.8 | 2.19 | 3.41 | 2.80 (2.19 to 3.41) |
| India | 2.64 | 2.06 | 3.22 | 2.64 (2.06 to 3.22) |
| Nepal | 2.49 | 2.22 | 2.76 | 2.49 (2.22 to 2.76) |
| China | 2.45 | 1.82 | 3.09 | 2.45 (1.82 to 3.09) |
| Thailand | 2.41 | 1.74 | 3.09 | 2.41 (1.74 to 3.09) |
| Indonesia | 2.37 | 1.8 | 2.94 | 2.37 (1.80 to 2.94) |
| Equatorial Guinea | 2.33 | 2.02 | 2.64 | 2.33 (2.02 to 2.64) |
| Pakistan | 2.25 | 1.91 | 2.6 | 2.25 (1.91 to 2.60) |
| Lao People's Democratic Republic | 2.23 | 1.64 | 2.81 | 2.23 (1.64 to 2.81) |
| Maldives | 2.23 | 1.64 | 2.82 | 2.23 (1.64 to 2.82) |
| Oman | 2.11 | 1.84 | 2.39 | 2.11 (1.84 to 2.39) |
| Botswana | 2.09 | 1.37 | 2.82 | 2.09 (1.37 to 2.82) |
| Eritrea | 2.02 | 1.39 | 2.65 | 2.02 (1.39 to 2.65) |
| Timor-Leste | 1.99 | 1.52 | 2.47 | 1.99 (1.52 to 2.47) |
| Comoros | 1.98 | 1.63 | 2.33 | 1.98 (1.63 to 2.33) |
| Ghana | 1.88 | 1.45 | 2.31 | 1.88 (1.45 to 2.31) |
| Yemen | 1.87 | 1.54 | 2.2 | 1.87 (1.54 to 2.20) |
| Angola | 1.87 | 1.52 | 2.21 | 1.87 (1.52 to 2.21) |
| Democratic Republic of the Congo | 1.85 | 1.63 | 2.07 | 1.85 (1.63 to 2.07) |
| Zambia | 1.85 | 1.43 | 2.28 | 1.85 (1.43 to 2.28) |
| Central African Republic | 1.83 | 1.26 | 2.4 | 1.83 (1.26 to 2.40) |
| Taiwan (Province of China) | 1.8 | 1.25 | 2.36 | 1.80 (1.25 to 2.36) |
| Haiti | 1.78 | 1.27 | 2.3 | 1.78 (1.27 to 2.30) |
| Kenya | 1.78 | 1.36 | 2.2 | 1.78 (1.36 to 2.20) |
| Singapore | 1.76 | 1.32 | 2.2 | 1.76 (1.32 to 2.20) |
| Mozambique | 1.69 | 1.27 | 2.11 | 1.69 (1.27 to 2.11) |
| Malawi | 1.68 | 1.61 | 1.75 | 1.68 (1.61 to 1.75) |
| Madagascar | 1.66 | 1.1 | 2.23 | 1.66 (1.10 to 2.23) |
| Djibouti | 1.62 | 1.16 | 2.08 | 1.62 (1.16 to 2.08) |
| United Republic of Tanzania | 1.62 | 1.09 | 2.17 | 1.62 (1.09 to 2.17) |
| Uganda | 1.62 | 1.13 | 2.11 | 1.62 (1.13 to 2.11) |
| Sudan | 1.62 | 1.22 | 2.02 | 1.62 (1.22 to 2.02) |
| Cambodia | 1.61 | 0.96 | 2.26 | 1.61 (0.96 to 2.26) |
| Namibia | 1.61 | 1.37 | 1.85 | 1.61 (1.37 to 1.85) |
| Cabo Verde | 1.61 | 1.08 | 2.14 | 1.61 (1.08 to 2.14) |
| Iran (Islamic Republic of) | 1.59 | 1.27 | 1.9 | 1.59 (1.27 to 1.90) |
| Myanmar | 1.58 | 0.83 | 2.33 | 1.58 (0.83 to 2.33) |
| Philippines | 1.58 | 1.05 | 2.12 | 1.58 (1.05 to 2.12) |
| Brunei Darussalam | 1.58 | 1.01 | 2.15 | 1.58 (1.01 to 2.15) |
| Congo | 1.57 | 1.12 | 2.02 | 1.57 (1.12 to 2.02) |
| Sri Lanka | 1.56 | 0.9 | 2.22 | 1.56 (0.90 to 2.22) |
| United Arab Emirates | 1.56 | 1.34 | 1.77 | 1.56 (1.34 to 1.77) |
| Republic of Korea | 1.55 | 0.99 | 2.12 | 1.55 (0.99 to 2.12) |
| Algeria | 1.55 | 1.15 | 1.94 | 1.55 (1.15 to 1.94) |
| Democratic People's Republic of Korea | 1.52 | 0.89 | 2.15 | 1.52 (0.89 to 2.15) |
| Nigeria | 1.51 | 1.22 | 1.79 | 1.51 (1.22 to 1.79) |
| Zimbabwe | 1.49 | 1.01 | 1.97 | 1.49 (1.01 to 1.97) |
| Gabon | 1.46 | 1.01 | 1.9 | 1.46 (1.01 to 1.90) |
| Rwanda | 1.46 | 0.92 | 2.01 | 1.46 (0.92 to 2.01) |
| Saudi Arabia | 1.43 | 1.17 | 1.68 | 1.43 (1.17 to 1.68) |
| Mauritius | 1.43 | 0.88 | 1.98 | 1.43 (0.88 to 1.98) |
| Tunisia | 1.42 | 1.09 | 1.75 | 1.42 (1.09 to 1.75) |
| Malaysia | 1.39 | 0.73 | 2.06 | 1.39 (0.73 to 2.06) |
| Togo | 1.39 | 0.8 | 1.99 | 1.39 (0.80 to 1.99) |
| Dominican Republic | 1.37 | 1.11 | 1.64 | 1.37 (1.11 to 1.64) |
| Lesotho | 1.35 | 0.59 | 2.11 | 1.35 (0.59 to 2.11) |
| Morocco | 1.33 | 0.83 | 1.83 | 1.33 (0.83 to 1.83) |
| Bhutan | 1.33 | 0.94 | 1.73 | 1.33 (0.94 to 1.73) |
| Saint Vincent and the Grenadines | 1.32 | 0.89 | 1.75 | 1.32 (0.89 to 1.75) |
| Cuba | 1.31 | 0.89 | 1.73 | 1.31 (0.89 to 1.73) |
| Sao Tome and Principe | 1.29 | 0.69 | 1.89 | 1.29 (0.69 to 1.89) |
| Bolivia (Plurinational State of) | 1.28 | 0.89 | 1.67 | 1.28 (0.89 to 1.67) |
| Ecuador | 1.27 | 0.83 | 1.7 | 1.27 (0.83 to 1.70) |
| Benin | 1.25 | 0.68 | 1.81 | 1.25 (0.68 to 1.81) |
| Libya | 1.24 | 0.93 | 1.54 | 1.24 (0.93 to 1.54) |
| Seychelles | 1.24 | 0.6 | 1.87 | 1.24 (0.60 to 1.87) |
| Syrian Arab Republic | 1.23 | 0.96 | 1.51 | 1.23 (0.96 to 1.51) |
| Guinea-Bissau | 1.23 | 0.72 | 1.76 | 1.23 (0.72 to 1.76) |
| Burkina Faso | 1.22 | 1.17 | 1.26 | 1.22 (1.17 to 1.26) |
| C么te d'Ivoire | 1.2 | 0.83 | 1.58 | 1.20 (0.83 to 1.58) |
| Sierra Leone | 1.2 | 0.59 | 1.82 | 1.20 (0.59 to 1.82) |
| Jamaica | 1.19 | 0.74 | 1.65 | 1.19 (0.74 to 1.65) |
| Colombia | 1.19 | 0.83 | 1.56 | 1.19 (0.83 to 1.56) |
| Cyprus | 1.18 | 0.81 | 1.55 | 1.18 (0.81 to 1.55) |
| Grenada | 1.18 | 0.69 | 1.68 | 1.18 (0.69 to 1.68) |
| Suriname | 1.18 | 0.84 | 1.52 | 1.18 (0.84 to 1.52) |
| Somalia | 1.17 | 0.56 | 1.79 | 1.17 (0.56 to 1.79) |
| Liberia | 1.16 | 0.89 | 1.43 | 1.16 (0.89 to 1.43) |
| Bahrain | 1.15 | 0.87 | 1.43 | 1.15 (0.87 to 1.43) |
| Eswatini | 1.15 | 0.71 | 1.6 | 1.15 (0.71 to 1.60) |
| Guinea | 1.15 | 0.56 | 1.73 | 1.15 (0.56 to 1.73) |
| Saint Lucia | 1.13 | 0.75 | 1.51 | 1.13 (0.75 to 1.51) |
| Kuwait | 1.13 | 0.87 | 1.39 | 1.13 (0.87 to 1.39) |
| Egypt | 1.12 | 0.81 | 1.42 | 1.12 (0.81 to 1.42) |
| Ethiopia | 1.12 | 0.92 | 1.33 | 1.12 (0.92 to 1.33) |
| T眉rkiye | 1.1 | 0.72 | 1.48 | 1.10 (0.72 to 1.48) |
| Burundi | 1.1 | 0.55 | 1.66 | 1.10 (0.55 to 1.66) |
| Australia | 1.09 | 0.75 | 1.42 | 1.09 (0.75 to 1.42) |
| Gambia | 1.08 | 0.63 | 1.54 | 1.08 (0.63 to 1.54) |
| Saint Kitts and Nevis | 1.08 | 0.75 | 1.41 | 1.08 (0.75 to 1.41) |
| Panama | 1.07 | 0.76 | 1.39 | 1.07 (0.76 to 1.39) |
| Brazil | 1.07 | 0.78 | 1.37 | 1.07 (0.78 to 1.37) |
| Afghanistan | 1.07 | 0.76 | 1.38 | 1.07 (0.76 to 1.38) |
| Cameroon | 1.07 | 0.71 | 1.42 | 1.07 (0.71 to 1.42) |
| South Sudan | 1.07 | 0.62 | 1.53 | 1.07 (0.62 to 1.53) |
| Peru | 1.06 | 0.68 | 1.45 | 1.06 (0.68 to 1.45) |
| Lebanon | 1.04 | 0.85 | 1.23 | 1.04 (0.85 to 1.23) |
| Malta | 1.03 | 0.59 | 1.48 | 1.03 (0.59 to 1.48) |
| Qatar | 1.03 | 0.82 | 1.24 | 1.03 (0.82 to 1.24) |
| Mauritania | 1.03 | 0.72 | 1.35 | 1.03 (0.72 to 1.35) |
| Jordan | 1.02 | 0.73 | 1.32 | 1.02 (0.73 to 1.32) |
| Mali | 1.02 | 0.37 | 1.68 | 1.02 (0.37 to 1.68) |
| Guyana | 1 | 0.65 | 1.35 | 1.00 (0.65 to 1.35) |
| Antigua and Barbuda | 0.99 | 0.65 | 1.34 | 0.99 (0.65 to 1.34) |
| Guatemala | 0.99 | 0.55 | 1.43 | 0.99 (0.55 to 1.43) |
| Vanuatu | 0.98 | 0.45 | 1.5 | 0.98 (0.45 to 1.50) |
| France | 0.98 | 0.64 | 1.33 | 0.98 (0.64 to 1.33) |
| Senegal | 0.98 | 0.5 | 1.46 | 0.98 (0.50 to 1.46) |
| Tuvalu | 0.97 | 0.66 | 1.29 | 0.97 (0.66 to 1.29) |
| Tokelau | 0.96 | 0.64 | 1.28 | 0.96 (0.64 to 1.28) |
| Fiji | 0.95 | 0.47 | 1.43 | 0.95 (0.47 to 1.43) |
| Trinidad and Tobago | 0.95 | 0.58 | 1.31 | 0.95 (0.58 to 1.31) |
| Russian Federation | 0.94 | 0.73 | 1.15 | 0.94 (0.73 to 1.15) |
| Chad | 0.93 | 0.37 | 1.5 | 0.93 (0.37 to 1.50) |
| Solomon Islands | 0.92 | 0.42 | 1.41 | 0.92 (0.42 to 1.41) |
| Republic of Moldova | 0.92 | 0.7 | 1.14 | 0.92 (0.70 to 1.14) |
| Argentina | 0.92 | 0.55 | 1.29 | 0.92 (0.55 to 1.29) |
| Honduras | 0.92 | 0.58 | 1.27 | 0.92 (0.58 to 1.27) |
| South Africa | 0.92 | 0.78 | 1.06 | 0.92 (0.78 to 1.06) |
| El Salvador | 0.91 | 0.61 | 1.22 | 0.91 (0.61 to 1.22) |
| Costa Rica | 0.89 | 0.6 | 1.18 | 0.89 (0.60 to 1.18) |
| Paraguay | 0.89 | 0.59 | 1.19 | 0.89 (0.59 to 1.19) |
| Belarus | 0.88 | 0.66 | 1.1 | 0.88 (0.66 to 1.10) |
| Serbia | 0.87 | 0.62 | 1.12 | 0.87 (0.62 to 1.12) |
| Nicaragua | 0.87 | 0.49 | 1.26 | 0.87 (0.49 to 1.26) |
| Niue | 0.87 | 0.49 | 1.25 | 0.87 (0.49 to 1.25) |
| Papua New Guinea | 0.86 | 0.34 | 1.38 | 0.86 (0.34 to 1.38) |
| New Zealand | 0.86 | 0.6 | 1.12 | 0.86 (0.60 to 1.12) |
| Belize | 0.86 | 0.53 | 1.19 | 0.86 (0.53 to 1.19) |
| Bahamas | 0.86 | 0.55 | 1.18 | 0.86 (0.55 to 1.18) |
| Barbados | 0.86 | 0.52 | 1.2 | 0.86 (0.52 to 1.20) |
| Mexico | 0.85 | 0.6 | 1.11 | 0.85 (0.60 to 1.11) |
| Portugal | 0.84 | 0.39 | 1.3 | 0.84 (0.39 to 1.30) |
| Kiribati | 0.82 | 0.38 | 1.26 | 0.82 (0.38 to 1.26) |
| Belgium | 0.82 | 0.41 | 1.22 | 0.82 (0.41 to 1.22) |
| Greece | 0.82 | 0.47 | 1.17 | 0.82 (0.47 to 1.17) |
| Chile | 0.82 | 0.47 | 1.17 | 0.82 (0.47 to 1.17) |
| Uruguay | 0.82 | 0.49 | 1.15 | 0.82 (0.49 to 1.15) |
| Venezuela (Bolivarian Republic of) | 0.82 | 0.52 | 1.13 | 0.82 (0.52 to 1.13) |
| Palestine | 0.82 | 0.5 | 1.13 | 0.82 (0.50 to 1.13) |
| Ireland | 0.8 | 0.45 | 1.15 | 0.80 (0.45 to 1.15) |
| Niger | 0.8 | 0 | 1.61 | 0.80 (-0.00 to 1.61) |
| Bermuda | 0.79 | 0.53 | 1.05 | 0.79 (0.53 to 1.05) |
| Denmark | 0.78 | 0.5 | 1.05 | 0.78 (0.50 to 1.05) |
| Dominica | 0.78 | 0.47 | 1.09 | 0.78 (0.47 to 1.09) |
| Puerto Rico | 0.78 | 0.54 | 1.03 | 0.78 (0.54 to 1.03) |
| Bosnia and Herzegovina | 0.77 | 0.54 | 0.99 | 0.77 (0.54 to 0.99) |
| Azerbaijan | 0.76 | 0.55 | 0.97 | 0.76 (0.55 to 0.97) |
| Croatia | 0.75 | 0.61 | 0.9 | 0.75 (0.61 to 0.90) |
| Romania | 0.75 | 0.62 | 0.87 | 0.75 (0.62 to 0.87) |
| Micronesia (Federated States of) | 0.74 | 0.33 | 1.15 | 0.74 (0.33 to 1.15) |
| Lithuania | 0.72 | 0.55 | 0.89 | 0.72 (0.55 to 0.89) |
| Netherlands | 0.72 | 0.35 | 1.1 | 0.72 (0.35 to 1.10) |
| United Kingdom | 0.72 | 0.41 | 1.03 | 0.72 (0.41 to 1.03) |
| Kazakhstan | 0.71 | 0.32 | 1.1 | 0.71 (0.32 to 1.10) |
| Uzbekistan | 0.7 | 0.44 | 0.96 | 0.70 (0.44 to 0.96) |
| Estonia | 0.7 | 0.53 | 0.88 | 0.70 (0.53 to 0.88) |
| Ukraine | 0.7 | 0.53 | 0.87 | 0.70 (0.53 to 0.87) |
| Finland | 0.7 | 0.32 | 1.08 | 0.70 (0.32 to 1.08) |
| Luxembourg | 0.7 | 0.35 | 1.05 | 0.70 (0.35 to 1.05) |
| Marshall Islands | 0.69 | 0.24 | 1.15 | 0.69 (0.24 to 1.15) |
| Albania | 0.69 | 0.47 | 0.91 | 0.69 (0.47 to 0.91) |
| Spain | 0.69 | 0.18 | 1.2 | 0.69 (0.18 to 1.20) |
| Cook Islands | 0.69 | 0.31 | 1.08 | 0.69 (0.31 to 1.08) |
| Guam | 0.68 | 0.31 | 1.06 | 0.68 (0.31 to 1.06) |
| Armenia | 0.67 | 0.33 | 1.01 | 0.67 (0.33 to 1.01) |
| Canada | 0.66 | 0.39 | 0.92 | 0.66 (0.39 to 0.92) |
| Greenland | 0.66 | 0.35 | 0.97 | 0.66 (0.35 to 0.97) |
| Nauru | 0.66 | 0.26 | 1.07 | 0.66 (0.26 to 1.07) |
| Tonga | 0.65 | 0.23 | 1.07 | 0.65 (0.23 to 1.07) |
| Andorra | 0.65 | 0.28 | 1.02 | 0.65 (0.28 to 1.02) |
| Italy | 0.65 | 0.29 | 1 | 0.65 (0.29 to 1.00) |
| Palau | 0.65 | 0.28 | 1.02 | 0.65 (0.28 to 1.02) |
| Poland | 0.64 | 0.46 | 0.82 | 0.64 (0.46 to 0.82) |
| Sweden | 0.64 | 0.32 | 0.96 | 0.64 (0.32 to 0.96) |
| Northern Mariana Islands | 0.64 | 0.29 | 0.99 | 0.64 (0.29 to 0.99) |
| Kyrgyzstan | 0.63 | 0.34 | 0.91 | 0.63 (0.34 to 0.91) |
| Japan | 0.63 | 0.14 | 1.13 | 0.63 (0.14 to 1.13) |
| North Macedonia | 0.62 | 0.43 | 0.81 | 0.62 (0.43 to 0.81) |
| Austria | 0.61 | 0.26 | 0.95 | 0.61 (0.26 to 0.95) |
| Iceland | 0.61 | 0.27 | 0.96 | 0.61 (0.27 to 0.96) |
| Slovenia | 0.6 | 0.48 | 0.73 | 0.60 (0.48 to 0.73) |
| Norway | 0.6 | 0.22 | 0.99 | 0.60 (0.22 to 0.99) |
| San Marino | 0.6 | 0.26 | 0.94 | 0.60 (0.26 to 0.94) |
| Montenegro | 0.59 | 0.5 | 0.68 | 0.59 (0.50 to 0.68) |
| Latvia | 0.59 | 0.37 | 0.82 | 0.59 (0.37 to 0.82) |
| Turkmenistan | 0.57 | 0.37 | 0.77 | 0.57 (0.37 to 0.77) |
| Israel | 0.57 | 0.14 | 1 | 0.57 (0.14 to 1.00) |
| Iraq | 0.57 | 0.37 | 0.76 | 0.57 (0.37 to 0.76) |
| United States Virgin Islands | 0.57 | 0.28 | 0.87 | 0.57 (0.28 to 0.87) |
| Hungary | 0.56 | 0.42 | 0.69 | 0.56 (0.42 to 0.69) |
| Samoa | 0.52 | 0.11 | 0.92 | 0.52 (0.11 to 0.92) |
| Switzerland | 0.52 | 0.17 | 0.86 | 0.52 (0.17 to 0.86) |
| Tajikistan | 0.5 | 0.25 | 0.75 | 0.50 (0.25 to 0.75) |
| American Samoa | 0.5 | 0.17 | 0.84 | 0.50 (0.17 to 0.84) |
| Monaco | 0.5 | 0.16 | 0.84 | 0.50 (0.16 to 0.84) |
| Czechia | 0.46 | 0.34 | 0.57 | 0.46 (0.34 to 0.57) |
| Germany | 0.46 | 0.11 | 0.8 | 0.46 (0.11 to 0.80) |
| Slovakia | 0.44 | 0.28 | 0.61 | 0.44 (0.28 to 0.61) |
| Mongolia | 0.43 | 0.19 | 0.67 | 0.43 (0.19 to 0.67) |
| Bulgaria | 0.41 | 0.26 | 0.57 | 0.41 (0.26 to 0.57) |
| Georgia | 0.39 | 0.2 | 0.58 | 0.39 (0.20 to 0.58) |
| United States of America | 0.38 | 0.06 | 0.7 | 0.38 (0.06 to 0.70) |

Appendix 10: The global DALYs rate for knee osteoarthritis due to high BMI across all age groups in 1990

| measure | location | sex | age | cause | rei | metric | year | val | upper | lower |
| --- | --- | --- | --- | --- | --- | --- | --- | --- | --- | --- |
| DALYs (Disability-Adjusted Life Years) | Global | Both | 75-79 years | Osteoarthritis knee | High body-mass index | Rate | 1990 | 181.021618 | 507.527604 | -14.723438 |
| YLDs (Years Lived with Disability) | Global | Both | 75-79 years | Osteoarthritis knee | High body-mass index | Rate | 1990 | 181.021618 | 507.527604 | -14.723438 |
| DALYs (Disability-Adjusted Life Years) | Global | Both | 80-84 years | Osteoarthritis knee | High body-mass index | Rate | 1990 | 170.718821 | 492.124246 | -12.89197 |
| YLDs (Years Lived with Disability) | Global | Both | 80-84 years | Osteoarthritis knee | High body-mass index | Rate | 1990 | 170.718821 | 492.124246 | -12.89197 |
| DALYs (Disability-Adjusted Life Years) | Global | Both | 85-89 years | Osteoarthritis knee | High body-mass index | Rate | 1990 | 169.147536 | 495.853852 | -13.019632 |
| YLDs (Years Lived with Disability) | Global | Both | 85-89 years | Osteoarthritis knee | High body-mass index | Rate | 1990 | 169.147536 | 495.853852 | -13.019632 |
| DALYs (Disability-Adjusted Life Years) | Global | Both | 70-74 years | Osteoarthritis knee | High body-mass index | Rate | 1990 | 167.579561 | 473.956927 | -14.004462 |
| YLDs (Years Lived with Disability) | Global | Both | 70-74 years | Osteoarthritis knee | High body-mass index | Rate | 1990 | 167.579561 | 473.956927 | -14.004462 |
| DALYs (Disability-Adjusted Life Years) | Global | Both | 90-94 years | Osteoarthritis knee | High body-mass index | Rate | 1990 | 160.445057 | 466.041748 | -12.695401 |
| YLDs (Years Lived with Disability) | Global | Both | 90-94 years | Osteoarthritis knee | High body-mass index | Rate | 1990 | 160.445057 | 466.041748 | -12.695401 |
| DALYs (Disability-Adjusted Life Years) | Global | Both | 65-69 years | Osteoarthritis knee | High body-mass index | Rate | 1990 | 154.691885 | 448.716779 | -13.711152 |
| YLDs (Years Lived with Disability) | Global | Both | 65-69 years | Osteoarthritis knee | High body-mass index | Rate | 1990 | 154.691885 | 448.716779 | -13.711152 |
| DALYs (Disability-Adjusted Life Years) | Global | Both | 95+ years | Osteoarthritis knee | High body-mass index | Rate | 1990 | 148.582756 | 422.672775 | -12.099131 |
| YLDs (Years Lived with Disability) | Global | Both | 95+ years | Osteoarthritis knee | High body-mass index | Rate | 1990 | 148.582756 | 422.672775 | -12.099131 |
| DALYs (Disability-Adjusted Life Years) | Global | Both | 60-64 years | Osteoarthritis knee | High body-mass index | Rate | 1990 | 131.33978 | 377.615957 | -11.892695 |
| YLDs (Years Lived with Disability) | Global | Both | 60-64 years | Osteoarthritis knee | High body-mass index | Rate | 1990 | 131.33978 | 377.615957 | -11.892695 |
| DALYs (Disability-Adjusted Life Years) | Global | Both | 55-59 years | Osteoarthritis knee | High body-mass index | Rate | 1990 | 105.098526 | 286.410687 | -9.6971893 |
| YLDs (Years Lived with Disability) | Global | Both | 55-59 years | Osteoarthritis knee | High body-mass index | Rate | 1990 | 105.098526 | 286.410687 | -9.6971893 |
| DALYs (Disability-Adjusted Life Years) | Global | Both | 50-54 years | Osteoarthritis knee | High body-mass index | Rate | 1990 | 77.0158222 | 212.655095 | -7.044862 |
| YLDs (Years Lived with Disability) | Global | Both | 50-54 years | Osteoarthritis knee | High body-mass index | Rate | 1990 | 77.0158222 | 212.655095 | -7.044862 |
| DALYs (Disability-Adjusted Life Years) | Global | Both | 45-49 years | Osteoarthritis knee | High body-mass index | Rate | 1990 | 46.5086317 | 125.81392 | -4.1671465 |
| YLDs (Years Lived with Disability) | Global | Both | 45-49 years | Osteoarthritis knee | High body-mass index | Rate | 1990 | 46.5086317 | 125.81392 | -4.1671465 |
| DALYs (Disability-Adjusted Life Years) | Global | Both | 40-44 years | Osteoarthritis knee | High body-mass index | Rate | 1990 | 21.9585494 | 62.2915917 | -1.895472 |
| YLDs (Years Lived with Disability) | Global | Both | 40-44 years | Osteoarthritis knee | High body-mass index | Rate | 1990 | 21.9585494 | 62.2915917 | -1.895472 |
| DALYs (Disability-Adjusted Life Years) | Global | Both | 35-39 years | Osteoarthritis knee | High body-mass index | Rate | 1990 | 7.05498435 | 20.2177546 | -0.5853346 |
| YLDs (Years Lived with Disability) | Global | Both | 35-39 years | Osteoarthritis knee | High body-mass index | Rate | 1990 | 7.05498435 | 20.2177546 | -0.5853346 |
| DALYs (Disability-Adjusted Life Years) | Global | Both | 30-34 years | Osteoarthritis knee | High body-mass index | Rate | 1990 | 0.69577218 | 2.00956493 | -0.0581875 |
| YLDs (Years Lived with Disability) | Global | Both | 30-34 years | Osteoarthritis knee | High body-mass index | Rate | 1990 | 0.69577218 | 2.00956493 | -0.0581875 |

Appendix 11: The global DALYs cases for knee osteoarthritis due to high BMI across all age groups in 1990

| measure | location | sex | age | cause | rei | metric | year | val | upper | lower |
| --- | --- | --- | --- | --- | --- | --- | --- | --- | --- | --- |
| DALYs (Disability-Adjusted Life Years) | Global | Both | 60-64 years | Osteoarthritis knee | High body-mass index | Number | 1990 | 210943.818 | 606486.107 | -19100.767 |
| YLDs (Years Lived with Disability) | Global | Both | 60-64 years | Osteoarthritis knee | High body-mass index | Number | 1990 | 210943.818 | 606486.107 | -19100.767 |
| DALYs (Disability-Adjusted Life Years) | Global | Both | 55-59 years | Osteoarthritis knee | High body-mass index | Number | 1990 | 194642.426 | 530432.474 | -17959.191 |
| YLDs (Years Lived with Disability) | Global | Both | 55-59 years | Osteoarthritis knee | High body-mass index | Number | 1990 | 194642.426 | 530432.474 | -17959.191 |
| DALYs (Disability-Adjusted Life Years) | Global | Both | 65-69 years | Osteoarthritis knee | High body-mass index | Number | 1990 | 191213.799 | 554656.375 | -16948.28 |
| YLDs (Years Lived with Disability) | Global | Both | 65-69 years | Osteoarthritis knee | High body-mass index | Number | 1990 | 191213.799 | 554656.375 | -16948.28 |
| DALYs (Disability-Adjusted Life Years) | Global | Both | 50-54 years | Osteoarthritis knee | High body-mass index | Number | 1990 | 163713.663 | 452044.055 | -14975.367 |
| YLDs (Years Lived with Disability) | Global | Both | 50-54 years | Osteoarthritis knee | High body-mass index | Number | 1990 | 163713.663 | 452044.055 | -14975.367 |
| DALYs (Disability-Adjusted Life Years) | Global | Both | 70-74 years | Osteoarthritis knee | High body-mass index | Number | 1990 | 141874.791 | 401257.406 | -11856.339 |
| YLDs (Years Lived with Disability) | Global | Both | 70-74 years | Osteoarthritis knee | High body-mass index | Number | 1990 | 141874.791 | 401257.406 | -11856.339 |
| DALYs (Disability-Adjusted Life Years) | Global | Both | 75-79 years | Osteoarthritis knee | High body-mass index | Number | 1990 | 111428.788 | 312411.227 | -9063.0877 |
| YLDs (Years Lived with Disability) | Global | Both | 75-79 years | Osteoarthritis knee | High body-mass index | Number | 1990 | 111428.788 | 312411.227 | -9063.0877 |
| DALYs (Disability-Adjusted Life Years) | Global | Both | 45-49 years | Osteoarthritis knee | High body-mass index | Number | 1990 | 107990.946 | 292134.252 | -9675.9263 |
| YLDs (Years Lived with Disability) | Global | Both | 45-49 years | Osteoarthritis knee | High body-mass index | Number | 1990 | 107990.946 | 292134.252 | -9675.9263 |
| DALYs (Disability-Adjusted Life Years) | Global | Both | 40-44 years | Osteoarthritis knee | High body-mass index | Number | 1990 | 62907.2113 | 178453.97 | -5430.1791 |
| YLDs (Years Lived with Disability) | Global | Both | 40-44 years | Osteoarthritis knee | High body-mass index | Number | 1990 | 62907.2113 | 178453.97 | -5430.1791 |
| DALYs (Disability-Adjusted Life Years) | Global | Both | 80-84 years | Osteoarthritis knee | High body-mass index | Number | 1990 | 60393.366 | 174093.515 | -4560.6539 |
| YLDs (Years Lived with Disability) | Global | Both | 80-84 years | Osteoarthritis knee | High body-mass index | Number | 1990 | 60393.366 | 174093.515 | -4560.6539 |
| DALYs (Disability-Adjusted Life Years) | Global | Both | 85-89 years | Osteoarthritis knee | High body-mass index | Number | 1990 | 25560.0067 | 74928.8351 | -1967.4061 |
| YLDs (Years Lived with Disability) | Global | Both | 85-89 years | Osteoarthritis knee | High body-mass index | Number | 1990 | 25560.0067 | 74928.8351 | -1967.4061 |
| DALYs (Disability-Adjusted Life Years) | Global | Both | 35-39 years | Osteoarthritis knee | High body-mass index | Number | 1990 | 24850.7923 | 71215.9228 | -2061.8088 |
| YLDs (Years Lived with Disability) | Global | Both | 35-39 years | Osteoarthritis knee | High body-mass index | Number | 1990 | 24850.7923 | 71215.9228 | -2061.8088 |
| DALYs (Disability-Adjusted Life Years) | Global | Both | 90-94 years | Osteoarthritis knee | High body-mass index | Number | 1990 | 6875.3716 | 19970.763 | -544.02176 |
| YLDs (Years Lived with Disability) | Global | Both | 90-94 years | Osteoarthritis knee | High body-mass index | Number | 1990 | 6875.3716 | 19970.763 | -544.02176 |
| DALYs (Disability-Adjusted Life Years) | Global | Both | 30-34 years | Osteoarthritis knee | High body-mass index | Number | 1990 | 2681.66244 | 7745.31512 | -224.26785 |
| YLDs (Years Lived with Disability) | Global | Both | 30-34 years | Osteoarthritis knee | High body-mass index | Number | 1990 | 2681.66244 | 7745.31512 | -224.26785 |
| DALYs (Disability-Adjusted Life Years) | Global | Both | 95+ years | Osteoarthritis knee | High body-mass index | Number | 1990 | 1512.70099 | 4303.17449 | -123.17962 |
| YLDs (Years Lived with Disability) | Global | Both | 95+ years | Osteoarthritis knee | High body-mass index | Number | 1990 | 1512.70099 | 4303.17449 | -123.17962 |

Appendix 12: The global DALYs rate for knee osteoarthritis due to high BMI across all age groups in 2021

| measure | location | sex | age | cause | rei | metric | year | val | upper | lower |
| --- | --- | --- | --- | --- | --- | --- | --- | --- | --- | --- |
| DALYs (Disability-Adjusted Life Years) | Global | Both | 75-79 years | Osteoarthritis knee | High body-mass index | Rate | 2021 | 231.439752 | 650.503748 | -19.901641 |
| YLDs (Years Lived with Disability) | Global | Both | 75-79 years | Osteoarthritis knee | High body-mass index | Rate | 2021 | 231.439752 | 650.503748 | -19.901641 |
| DALYs (Disability-Adjusted Life Years) | Global | Both | 70-74 years | Osteoarthritis knee | High body-mass index | Rate | 2021 | 227.468031 | 629.935317 | -20.306821 |
| YLDs (Years Lived with Disability) | Global | Both | 70-74 years | Osteoarthritis knee | High body-mass index | Rate | 2021 | 227.468031 | 629.935317 | -20.306821 |
| DALYs (Disability-Adjusted Life Years) | Global | Both | 80-84 years | Osteoarthritis knee | High body-mass index | Rate | 2021 | 218.156505 | 640.839003 | -17.44881 |
| YLDs (Years Lived with Disability) | Global | Both | 80-84 years | Osteoarthritis knee | High body-mass index | Rate | 2021 | 218.156505 | 640.839003 | -17.44881 |
| DALYs (Disability-Adjusted Life Years) | Global | Both | 85-89 years | Osteoarthritis knee | High body-mass index | Rate | 2021 | 216.848102 | 625.491577 | -17.491193 |
| YLDs (Years Lived with Disability) | Global | Both | 85-89 years | Osteoarthritis knee | High body-mass index | Rate | 2021 | 216.848102 | 625.491577 | -17.491193 |
| DALYs (Disability-Adjusted Life Years) | Global | Both | 65-69 years | Osteoarthritis knee | High body-mass index | Rate | 2021 | 211.759151 | 601.564751 | -19.964837 |
| YLDs (Years Lived with Disability) | Global | Both | 65-69 years | Osteoarthritis knee | High body-mass index | Rate | 2021 | 211.759151 | 601.564751 | -19.964837 |
| DALYs (Disability-Adjusted Life Years) | Global | Both | 90-94 years | Osteoarthritis knee | High body-mass index | Rate | 2021 | 207.167329 | 586.146399 | -17.179031 |
| YLDs (Years Lived with Disability) | Global | Both | 90-94 years | Osteoarthritis knee | High body-mass index | Rate | 2021 | 207.167329 | 586.146399 | -17.179031 |
| DALYs (Disability-Adjusted Life Years) | Global | Both | 95+ years | Osteoarthritis knee | High body-mass index | Rate | 2021 | 192.136767 | 533.811594 | -16.245646 |
| YLDs (Years Lived with Disability) | Global | Both | 95+ years | Osteoarthritis knee | High body-mass index | Rate | 2021 | 192.136767 | 533.811594 | -16.245646 |
| DALYs (Disability-Adjusted Life Years) | Global | Both | 60-64 years | Osteoarthritis knee | High body-mass index | Rate | 2021 | 187.488736 | 520.716244 | -18.74818 |
| YLDs (Years Lived with Disability) | Global | Both | 60-64 years | Osteoarthritis knee | High body-mass index | Rate | 2021 | 187.488736 | 520.716244 | -18.74818 |
| DALYs (Disability-Adjusted Life Years) | Global | Both | 55-59 years | Osteoarthritis knee | High body-mass index | Rate | 2021 | 158.409081 | 422.199449 | -16.033403 |
| YLDs (Years Lived with Disability) | Global | Both | 55-59 years | Osteoarthritis knee | High body-mass index | Rate | 2021 | 158.409081 | 422.199449 | -16.033403 |
| DALYs (Disability-Adjusted Life Years) | Global | Both | 50-54 years | Osteoarthritis knee | High body-mass index | Rate | 2021 | 118.501176 | 319.347993 | -12.163676 |
| YLDs (Years Lived with Disability) | Global | Both | 50-54 years | Osteoarthritis knee | High body-mass index | Rate | 2021 | 118.501176 | 319.347993 | -12.163676 |
| DALYs (Disability-Adjusted Life Years) | Global | Both | 45-49 years | Osteoarthritis knee | High body-mass index | Rate | 2021 | 70.8156755 | 186.443857 | -6.929802 |
| YLDs (Years Lived with Disability) | Global | Both | 45-49 years | Osteoarthritis knee | High body-mass index | Rate | 2021 | 70.8156755 | 186.443857 | -6.929802 |
| DALYs (Disability-Adjusted Life Years) | Global | Both | 40-44 years | Osteoarthritis knee | High body-mass index | Rate | 2021 | 33.3090763 | 90.8956908 | -3.1030681 |
| YLDs (Years Lived with Disability) | Global | Both | 40-44 years | Osteoarthritis knee | High body-mass index | Rate | 2021 | 33.3090763 | 90.8956908 | -3.1030681 |
| DALYs (Disability-Adjusted Life Years) | Global | Both | 35-39 years | Osteoarthritis knee | High body-mass index | Rate | 2021 | 10.9720625 | 31.0476695 | -0.9715142 |
| YLDs (Years Lived with Disability) | Global | Both | 35-39 years | Osteoarthritis knee | High body-mass index | Rate | 2021 | 10.9720625 | 31.0476695 | -0.9715142 |
| DALYs (Disability-Adjusted Life Years) | Global | Both | 30-34 years | Osteoarthritis knee | High body-mass index | Rate | 2021 | 1.0901675 | 3.13501566 | -0.0960904 |
| YLDs (Years Lived with Disability) | Global | Both | 30-34 years | Osteoarthritis knee | High body-mass index | Rate | 2021 | 1.0901675 | 3.13501566 | -0.0960904 |

Appendix 13: The global DALYs cases for knee osteoarthritis due to high BMI across all age groups in 2021

| measure | location | sex | age | cause | rei | metric | year | val | upper | lower |
| --- | --- | --- | --- | --- | --- | --- | --- | --- | --- | --- |
| DALYs (Disability-Adjusted Life Years) | Global | Both | 55-59 years | Osteoarthritis knee | High body-mass index | Number | 2021 | 626869.096 | 1670761.45 | -63448.668 |
| YLDs (Years Lived with Disability) | Global | Both | 55-59 years | Osteoarthritis knee | High body-mass index | Number | 2021 | 626869.096 | 1670761.45 | -63448.668 |
| DALYs (Disability-Adjusted Life Years) | Global | Both | 60-64 years | Osteoarthritis knee | High body-mass index | Number | 2021 | 600053.673 | 1666541.16 | -60003.148 |
| YLDs (Years Lived with Disability) | Global | Both | 60-64 years | Osteoarthritis knee | High body-mass index | Number | 2021 | 600053.673 | 1666541.16 | -60003.148 |
| DALYs (Disability-Adjusted Life Years) | Global | Both | 65-69 years | Osteoarthritis knee | High body-mass index | Number | 2021 | 584121.013 | 1659369.19 | -55071.437 |
| YLDs (Years Lived with Disability) | Global | Both | 65-69 years | Osteoarthritis knee | High body-mass index | Number | 2021 | 584121.013 | 1659369.19 | -55071.437 |
| DALYs (Disability-Adjusted Life Years) | Global | Both | 50-54 years | Osteoarthritis knee | High body-mass index | Number | 2021 | 527238.969 | 1420852.61 | -54118.991 |
| YLDs (Years Lived with Disability) | Global | Both | 50-54 years | Osteoarthritis knee | High body-mass index | Number | 2021 | 527238.969 | 1420852.61 | -54118.991 |
| DALYs (Disability-Adjusted Life Years) | Global | Both | 70-74 years | Osteoarthritis knee | High body-mass index | Number | 2021 | 468218.422 | 1296653.95 | -41799.402 |
| YLDs (Years Lived with Disability) | Global | Both | 70-74 years | Osteoarthritis knee | High body-mass index | Number | 2021 | 468218.422 | 1296653.95 | -41799.402 |
| DALYs (Disability-Adjusted Life Years) | Global | Both | 45-49 years | Osteoarthritis knee | High body-mass index | Number | 2021 | 335315.499 | 882820.29 | -32812.933 |
| YLDs (Years Lived with Disability) | Global | Both | 45-49 years | Osteoarthritis knee | High body-mass index | Number | 2021 | 335315.499 | 882820.29 | -32812.933 |
| DALYs (Disability-Adjusted Life Years) | Global | Both | 75-79 years | Osteoarthritis knee | High body-mass index | Number | 2021 | 305232.939 | 857912.995 | -26247.16 |
| YLDs (Years Lived with Disability) | Global | Both | 75-79 years | Osteoarthritis knee | High body-mass index | Number | 2021 | 305232.939 | 857912.995 | -26247.16 |
| DALYs (Disability-Adjusted Life Years) | Global | Both | 80-84 years | Osteoarthritis knee | High body-mass index | Number | 2021 | 191068.136 | 561266.389 | -15282.201 |
| YLDs (Years Lived with Disability) | Global | Both | 80-84 years | Osteoarthritis knee | High body-mass index | Number | 2021 | 191068.136 | 561266.389 | -15282.201 |
| DALYs (Disability-Adjusted Life Years) | Global | Both | 40-44 years | Osteoarthritis knee | High body-mass index | Number | 2021 | 166628.919 | 454706.416 | -15523.123 |
| YLDs (Years Lived with Disability) | Global | Both | 40-44 years | Osteoarthritis knee | High body-mass index | Number | 2021 | 166628.919 | 454706.416 | -15523.123 |
| DALYs (Disability-Adjusted Life Years) | Global | Both | 85-89 years | Osteoarthritis knee | High body-mass index | Number | 2021 | 99146.8361 | 285985.952 | -7997.2867 |
| YLDs (Years Lived with Disability) | Global | Both | 85-89 years | Osteoarthritis knee | High body-mass index | Number | 2021 | 99146.8361 | 285985.952 | -7997.2867 |
| DALYs (Disability-Adjusted Life Years) | Global | Both | 35-39 years | Osteoarthritis knee | High body-mass index | Number | 2021 | 61538.5797 | 174135.855 | -5448.8936 |
| YLDs (Years Lived with Disability) | Global | Both | 35-39 years | Osteoarthritis knee | High body-mass index | Number | 2021 | 61538.5797 | 174135.855 | -5448.8936 |
| DALYs (Disability-Adjusted Life Years) | Global | Both | 90-94 years | Osteoarthritis knee | High body-mass index | Number | 2021 | 37060.9374 | 104857.919 | -3073.2211 |
| YLDs (Years Lived with Disability) | Global | Both | 90-94 years | Osteoarthritis knee | High body-mass index | Number | 2021 | 37060.9374 | 104857.919 | -3073.2211 |
| DALYs (Disability-Adjusted Life Years) | Global | Both | 95+ years | Osteoarthritis knee | High body-mass index | Number | 2021 | 10472.0469 | 29094.3796 | -885.43787 |
| YLDs (Years Lived with Disability) | Global | Both | 95+ years | Osteoarthritis knee | High body-mass index | Number | 2021 | 10472.0469 | 29094.3796 | -885.43787 |
| DALYs (Disability-Adjusted Life Years) | Global | Both | 30-34 years | Osteoarthritis knee | High body-mass index | Number | 2021 | 6589.84642 | 18950.5482 | -580.84712 |
| YLDs (Years Lived with Disability) | Global | Both | 30-34 years | Osteoarthritis knee | High body-mass index | Number | 2021 | 6589.84642 | 18950.5482 | -580.84712 |

Appendix 14: The global DALYs rate and DALYs cases for knee osteoarthritis due to high BMI across all age groups from 1990 to 2021

| measure | location | sex | age | cause | rei | metric | year | val | upper | lower |
| --- | --- | --- | --- | --- | --- | --- | --- | --- | --- | --- |
| DALYs (Disability-Adjusted Life Years) | Global | Both | 30-34 years | Osteoarthritis knee | High body-mass index | Number | 2021 | 6589.84642 | 18950.5482 | -580.84712 |
| DALYs (Disability-Adjusted Life Years) | Global | Both | 30-34 years | Osteoarthritis knee | High body-mass index | Rate | 2021 | 1.0901675 | 3.13501566 | -0.0960904 |
| DALYs (Disability-Adjusted Life Years) | Global | Both | 30-34 years | Osteoarthritis knee | High body-mass index | Number | 1990 | 2681.66244 | 7745.31512 | -224.26785 |
| YLDs (Years Lived with Disability) | Global | Both | 30-34 years | Osteoarthritis knee | High body-mass index | Number | 1990 | 2681.66244 | 7745.31512 | -224.26785 |
| YLDs (Years Lived with Disability) | Global | Both | 30-34 years | Osteoarthritis knee | High body-mass index | Number | 2021 | 6589.84642 | 18950.5482 | -580.84712 |
| YLDs (Years Lived with Disability) | Global | Both | 30-34 years | Osteoarthritis knee | High body-mass index | Rate | 2021 | 1.0901675 | 3.13501566 | -0.0960904 |
| DALYs (Disability-Adjusted Life Years) | Global | Both | 30-34 years | Osteoarthritis knee | High body-mass index | Rate | 1990 | 0.69577218 | 2.00956493 | -0.0581875 |
| YLDs (Years Lived with Disability) | Global | Both | 30-34 years | Osteoarthritis knee | High body-mass index | Rate | 1990 | 0.69577218 | 2.00956493 | -0.0581875 |
| DALYs (Disability-Adjusted Life Years) | Global | Both | 35-39 years | Osteoarthritis knee | High body-mass index | Number | 1990 | 24850.7923 | 71215.9228 | -2061.8088 |
| YLDs (Years Lived with Disability) | Global | Both | 35-39 years | Osteoarthritis knee | High body-mass index | Number | 1990 | 24850.7923 | 71215.9228 | -2061.8088 |
| DALYs (Disability-Adjusted Life Years) | Global | Both | 35-39 years | Osteoarthritis knee | High body-mass index | Number | 2021 | 61538.5797 | 174135.855 | -5448.8936 |
| YLDs (Years Lived with Disability) | Global | Both | 35-39 years | Osteoarthritis knee | High body-mass index | Number | 2021 | 61538.5797 | 174135.855 | -5448.8936 |
| DALYs (Disability-Adjusted Life Years) | Global | Both | 35-39 years | Osteoarthritis knee | High body-mass index | Rate | 2021 | 10.9720625 | 31.0476695 | -0.9715142 |
| YLDs (Years Lived with Disability) | Global | Both | 35-39 years | Osteoarthritis knee | High body-mass index | Rate | 2021 | 10.9720625 | 31.0476695 | -0.9715142 |
| DALYs (Disability-Adjusted Life Years) | Global | Both | 35-39 years | Osteoarthritis knee | High body-mass index | Rate | 1990 | 7.05498435 | 20.2177546 | -0.5853346 |
| YLDs (Years Lived with Disability) | Global | Both | 35-39 years | Osteoarthritis knee | High body-mass index | Rate | 1990 | 7.05498435 | 20.2177546 | -0.5853346 |
| DALYs (Disability-Adjusted Life Years) | Global | Both | 40-44 years | Osteoarthritis knee | High body-mass index | Number | 1990 | 62907.2113 | 178453.97 | -5430.1791 |
| YLDs (Years Lived with Disability) | Global | Both | 40-44 years | Osteoarthritis knee | High body-mass index | Number | 1990 | 62907.2113 | 178453.97 | -5430.1791 |
| DALYs (Disability-Adjusted Life Years) | Global | Both | 40-44 years | Osteoarthritis knee | High body-mass index | Number | 2021 | 166628.919 | 454706.416 | -15523.123 |
| YLDs (Years Lived with Disability) | Global | Both | 40-44 years | Osteoarthritis knee | High body-mass index | Number | 2021 | 166628.919 | 454706.416 | -15523.123 |
| DALYs (Disability-Adjusted Life Years) | Global | Both | 40-44 years | Osteoarthritis knee | High body-mass index | Rate | 2021 | 33.3090763 | 90.8956908 | -3.1030681 |
| YLDs (Years Lived with Disability) | Global | Both | 40-44 years | Osteoarthritis knee | High body-mass index | Rate | 2021 | 33.3090763 | 90.8956908 | -3.1030681 |
| DALYs (Disability-Adjusted Life Years) | Global | Both | 40-44 years | Osteoarthritis knee | High body-mass index | Rate | 1990 | 21.9585494 | 62.2915917 | -1.895472 |
| YLDs (Years Lived with Disability) | Global | Both | 40-44 years | Osteoarthritis knee | High body-mass index | Rate | 1990 | 21.9585494 | 62.2915917 | -1.895472 |
| DALYs (Disability-Adjusted Life Years) | Global | Both | 45-49 years | Osteoarthritis knee | High body-mass index | Number | 1990 | 107990.946 | 292134.252 | -9675.9263 |
| YLDs (Years Lived with Disability) | Global | Both | 45-49 years | Osteoarthritis knee | High body-mass index | Number | 1990 | 107990.946 | 292134.252 | -9675.9263 |
| DALYs (Disability-Adjusted Life Years) | Global | Both | 45-49 years | Osteoarthritis knee | High body-mass index | Number | 2021 | 335315.499 | 882820.29 | -32812.933 |
| YLDs (Years Lived with Disability) | Global | Both | 45-49 years | Osteoarthritis knee | High body-mass index | Number | 2021 | 335315.499 | 882820.29 | -32812.933 |
| DALYs (Disability-Adjusted Life Years) | Global | Both | 45-49 years | Osteoarthritis knee | High body-mass index | Rate | 2021 | 70.8156755 | 186.443857 | -6.929802 |
| YLDs (Years Lived with Disability) | Global | Both | 45-49 years | Osteoarthritis knee | High body-mass index | Rate | 2021 | 70.8156755 | 186.443857 | -6.929802 |
| DALYs (Disability-Adjusted Life Years) | Global | Both | 45-49 years | Osteoarthritis knee | High body-mass index | Rate | 1990 | 46.5086317 | 125.81392 | -4.1671465 |
| YLDs (Years Lived with Disability) | Global | Both | 45-49 years | Osteoarthritis knee | High body-mass index | Rate | 1990 | 46.5086317 | 125.81392 | -4.1671465 |
| DALYs (Disability-Adjusted Life Years) | Global | Both | 50-54 years | Osteoarthritis knee | High body-mass index | Number | 1990 | 163713.663 | 452044.055 | -14975.367 |
| YLDs (Years Lived with Disability) | Global | Both | 50-54 years | Osteoarthritis knee | High body-mass index | Number | 1990 | 163713.663 | 452044.055 | -14975.367 |
| DALYs (Disability-Adjusted Life Years) | Global | Both | 50-54 years | Osteoarthritis knee | High body-mass index | Number | 2021 | 527238.969 | 1420852.61 | -54118.991 |
| YLDs (Years Lived with Disability) | Global | Both | 50-54 years | Osteoarthritis knee | High body-mass index | Number | 2021 | 527238.969 | 1420852.61 | -54118.991 |
| DALYs (Disability-Adjusted Life Years) | Global | Both | 50-54 years | Osteoarthritis knee | High body-mass index | Rate | 1990 | 77.0158222 | 212.655095 | -7.044862 |
| YLDs (Years Lived with Disability) | Global | Both | 50-54 years | Osteoarthritis knee | High body-mass index | Rate | 1990 | 77.0158222 | 212.655095 | -7.044862 |
| DALYs (Disability-Adjusted Life Years) | Global | Both | 50-54 years | Osteoarthritis knee | High body-mass index | Rate | 2021 | 118.501176 | 319.347993 | -12.163676 |
| YLDs (Years Lived with Disability) | Global | Both | 50-54 years | Osteoarthritis knee | High body-mass index | Rate | 2021 | 118.501176 | 319.347993 | -12.163676 |
| DALYs (Disability-Adjusted Life Years) | Global | Both | 55-59 years | Osteoarthritis knee | High body-mass index | Number | 1990 | 194642.426 | 530432.474 | -17959.191 |
| YLDs (Years Lived with Disability) | Global | Both | 55-59 years | Osteoarthritis knee | High body-mass index | Number | 1990 | 194642.426 | 530432.474 | -17959.191 |
| DALYs (Disability-Adjusted Life Years) | Global | Both | 55-59 years | Osteoarthritis knee | High body-mass index | Number | 2021 | 626869.096 | 1670761.45 | -63448.668 |
| YLDs (Years Lived with Disability) | Global | Both | 55-59 years | Osteoarthritis knee | High body-mass index | Number | 2021 | 626869.096 | 1670761.45 | -63448.668 |
| DALYs (Disability-Adjusted Life Years) | Global | Both | 55-59 years | Osteoarthritis knee | High body-mass index | Rate | 1990 | 105.098526 | 286.410687 | -9.6971893 |
| YLDs (Years Lived with Disability) | Global | Both | 55-59 years | Osteoarthritis knee | High body-mass index | Rate | 1990 | 105.098526 | 286.410687 | -9.6971893 |
| DALYs (Disability-Adjusted Life Years) | Global | Both | 55-59 years | Osteoarthritis knee | High body-mass index | Rate | 2021 | 158.409081 | 422.199449 | -16.033403 |
| YLDs (Years Lived with Disability) | Global | Both | 55-59 years | Osteoarthritis knee | High body-mass index | Rate | 2021 | 158.409081 | 422.199449 | -16.033403 |
| DALYs (Disability-Adjusted Life Years) | Global | Both | 60-64 years | Osteoarthritis knee | High body-mass index | Number | 1990 | 210943.818 | 606486.107 | -19100.767 |
| YLDs (Years Lived with Disability) | Global | Both | 60-64 years | Osteoarthritis knee | High body-mass index | Number | 1990 | 210943.818 | 606486.107 | -19100.767 |
| DALYs (Disability-Adjusted Life Years) | Global | Both | 60-64 years | Osteoarthritis knee | High body-mass index | Number | 2021 | 600053.673 | 1666541.16 | -60003.148 |
| YLDs (Years Lived with Disability) | Global | Both | 60-64 years | Osteoarthritis knee | High body-mass index | Number | 2021 | 600053.673 | 1666541.16 | -60003.148 |
| DALYs (Disability-Adjusted Life Years) | Global | Both | 60-64 years | Osteoarthritis knee | High body-mass index | Rate | 1990 | 131.33978 | 377.615957 | -11.892695 |
| YLDs (Years Lived with Disability) | Global | Both | 60-64 years | Osteoarthritis knee | High body-mass index | Rate | 1990 | 131.33978 | 377.615957 | -11.892695 |
| DALYs (Disability-Adjusted Life Years) | Global | Both | 60-64 years | Osteoarthritis knee | High body-mass index | Rate | 2021 | 187.488736 | 520.716244 | -18.74818 |
| YLDs (Years Lived with Disability) | Global | Both | 60-64 years | Osteoarthritis knee | High body-mass index | Rate | 2021 | 187.488736 | 520.716244 | -18.74818 |
| DALYs (Disability-Adjusted Life Years) | Global | Both | 65-69 years | Osteoarthritis knee | High body-mass index | Number | 1990 | 191213.799 | 554656.375 | -16948.28 |
| YLDs (Years Lived with Disability) | Global | Both | 65-69 years | Osteoarthritis knee | High body-mass index | Number | 1990 | 191213.799 | 554656.375 | -16948.28 |
| DALYs (Disability-Adjusted Life Years) | Global | Both | 65-69 years | Osteoarthritis knee | High body-mass index | Number | 2021 | 584121.013 | 1659369.19 | -55071.437 |
| DALYs (Disability-Adjusted Life Years) | Global | Both | 65-69 years | Osteoarthritis knee | High body-mass index | Rate | 1990 | 154.691885 | 448.716779 | -13.711152 |
| YLDs (Years Lived with Disability) | Global | Both | 65-69 years | Osteoarthritis knee | High body-mass index | Number | 2021 | 584121.013 | 1659369.19 | -55071.437 |
| YLDs (Years Lived with Disability) | Global | Both | 65-69 years | Osteoarthritis knee | High body-mass index | Rate | 1990 | 154.691885 | 448.716779 | -13.711152 |
| DALYs (Disability-Adjusted Life Years) | Global | Both | 65-69 years | Osteoarthritis knee | High body-mass index | Rate | 2021 | 211.759151 | 601.564751 | -19.964837 |
| YLDs (Years Lived with Disability) | Global | Both | 65-69 years | Osteoarthritis knee | High body-mass index | Rate | 2021 | 211.759151 | 601.564751 | -19.964837 |
| DALYs (Disability-Adjusted Life Years) | Global | Both | 70-74 years | Osteoarthritis knee | High body-mass index | Number | 1990 | 141874.791 | 401257.406 | -11856.339 |
| YLDs (Years Lived with Disability) | Global | Both | 70-74 years | Osteoarthritis knee | High body-mass index | Number | 1990 | 141874.791 | 401257.406 | -11856.339 |
| DALYs (Disability-Adjusted Life Years) | Global | Both | 70-74 years | Osteoarthritis knee | High body-mass index | Rate | 1990 | 167.579561 | 473.956927 | -14.004462 |
| YLDs (Years Lived with Disability) | Global | Both | 70-74 years | Osteoarthritis knee | High body-mass index | Rate | 1990 | 167.579561 | 473.956927 | -14.004462 |
| DALYs (Disability-Adjusted Life Years) | Global | Both | 70-74 years | Osteoarthritis knee | High body-mass index | Rate | 2021 | 227.468031 | 629.935317 | -20.306821 |
| YLDs (Years Lived with Disability) | Global | Both | 70-74 years | Osteoarthritis knee | High body-mass index | Rate | 2021 | 227.468031 | 629.935317 | -20.306821 |
| DALYs (Disability-Adjusted Life Years) | Global | Both | 70-74 years | Osteoarthritis knee | High body-mass index | Number | 2021 | 468218.422 | 1296653.95 | -41799.402 |
| YLDs (Years Lived with Disability) | Global | Both | 70-74 years | Osteoarthritis knee | High body-mass index | Number | 2021 | 468218.422 | 1296653.95 | -41799.402 |
| DALYs (Disability-Adjusted Life Years) | Global | Both | 75-79 years | Osteoarthritis knee | High body-mass index | Number | 1990 | 111428.788 | 312411.227 | -9063.0877 |
| YLDs (Years Lived with Disability) | Global | Both | 75-79 years | Osteoarthritis knee | High body-mass index | Number | 1990 | 111428.788 | 312411.227 | -9063.0877 |
| DALYs (Disability-Adjusted Life Years) | Global | Both | 75-79 years | Osteoarthritis knee | High body-mass index | Rate | 1990 | 181.021618 | 507.527604 | -14.723438 |
| YLDs (Years Lived with Disability) | Global | Both | 75-79 years | Osteoarthritis knee | High body-mass index | Rate | 1990 | 181.021618 | 507.527604 | -14.723438 |
| DALYs (Disability-Adjusted Life Years) | Global | Both | 75-79 years | Osteoarthritis knee | High body-mass index | Rate | 2021 | 231.439752 | 650.503748 | -19.901641 |
| YLDs (Years Lived with Disability) | Global | Both | 75-79 years | Osteoarthritis knee | High body-mass index | Rate | 2021 | 231.439752 | 650.503748 | -19.901641 |
| DALYs (Disability-Adjusted Life Years) | Global | Both | 75-79 years | Osteoarthritis knee | High body-mass index | Number | 2021 | 305232.939 | 857912.995 | -26247.16 |
| YLDs (Years Lived with Disability) | Global | Both | 75-79 years | Osteoarthritis knee | High body-mass index | Number | 2021 | 305232.939 | 857912.995 | -26247.16 |
| DALYs (Disability-Adjusted Life Years) | Global | Both | 80-84 years | Osteoarthritis knee | High body-mass index | Number | 1990 | 60393.366 | 174093.515 | -4560.6539 |
| YLDs (Years Lived with Disability) | Global | Both | 80-84 years | Osteoarthritis knee | High body-mass index | Number | 1990 | 60393.366 | 174093.515 | -4560.6539 |
| DALYs (Disability-Adjusted Life Years) | Global | Both | 80-84 years | Osteoarthritis knee | High body-mass index | Rate | 1990 | 170.718821 | 492.124246 | -12.89197 |
| YLDs (Years Lived with Disability) | Global | Both | 80-84 years | Osteoarthritis knee | High body-mass index | Rate | 1990 | 170.718821 | 492.124246 | -12.89197 |
| DALYs (Disability-Adjusted Life Years) | Global | Both | 80-84 years | Osteoarthritis knee | High body-mass index | Rate | 2021 | 218.156505 | 640.839003 | -17.44881 |
| YLDs (Years Lived with Disability) | Global | Both | 80-84 years | Osteoarthritis knee | High body-mass index | Rate | 2021 | 218.156505 | 640.839003 | -17.44881 |
| DALYs (Disability-Adjusted Life Years) | Global | Both | 80-84 years | Osteoarthritis knee | High body-mass index | Number | 2021 | 191068.136 | 561266.389 | -15282.201 |
| YLDs (Years Lived with Disability) | Global | Both | 80-84 years | Osteoarthritis knee | High body-mass index | Number | 2021 | 191068.136 | 561266.389 | -15282.201 |
| DALYs (Disability-Adjusted Life Years) | Global | Both | 85-89 years | Osteoarthritis knee | High body-mass index | Rate | 1990 | 169.147536 | 495.853852 | -13.019632 |
| YLDs (Years Lived with Disability) | Global | Both | 85-89 years | Osteoarthritis knee | High body-mass index | Rate | 1990 | 169.147536 | 495.853852 | -13.019632 |
| DALYs (Disability-Adjusted Life Years) | Global | Both | 85-89 years | Osteoarthritis knee | High body-mass index | Rate | 2021 | 216.848102 | 625.491577 | -17.491193 |
| YLDs (Years Lived with Disability) | Global | Both | 85-89 years | Osteoarthritis knee | High body-mass index | Rate | 2021 | 216.848102 | 625.491577 | -17.491193 |
| DALYs (Disability-Adjusted Life Years) | Global | Both | 85-89 years | Osteoarthritis knee | High body-mass index | Number | 1990 | 25560.0067 | 74928.8351 | -1967.4061 |
| YLDs (Years Lived with Disability) | Global | Both | 85-89 years | Osteoarthritis knee | High body-mass index | Number | 1990 | 25560.0067 | 74928.8351 | -1967.4061 |
| DALYs (Disability-Adjusted Life Years) | Global | Both | 85-89 years | Osteoarthritis knee | High body-mass index | Number | 2021 | 99146.8361 | 285985.952 | -7997.2867 |
| YLDs (Years Lived with Disability) | Global | Both | 85-89 years | Osteoarthritis knee | High body-mass index | Number | 2021 | 99146.8361 | 285985.952 | -7997.2867 |
| DALYs (Disability-Adjusted Life Years) | Global | Both | 90-94 years | Osteoarthritis knee | High body-mass index | Rate | 1990 | 160.445057 | 466.041748 | -12.695401 |
| YLDs (Years Lived with Disability) | Global | Both | 90-94 years | Osteoarthritis knee | High body-mass index | Rate | 1990 | 160.445057 | 466.041748 | -12.695401 |
| DALYs (Disability-Adjusted Life Years) | Global | Both | 90-94 years | Osteoarthritis knee | High body-mass index | Rate | 2021 | 207.167329 | 586.146399 | -17.179031 |
| YLDs (Years Lived with Disability) | Global | Both | 90-94 years | Osteoarthritis knee | High body-mass index | Rate | 2021 | 207.167329 | 586.146399 | -17.179031 |
| DALYs (Disability-Adjusted Life Years) | Global | Both | 90-94 years | Osteoarthritis knee | High body-mass index | Number | 1990 | 6875.3716 | 19970.763 | -544.02176 |
| YLDs (Years Lived with Disability) | Global | Both | 90-94 years | Osteoarthritis knee | High body-mass index | Number | 1990 | 6875.3716 | 19970.763 | -544.02176 |
| DALYs (Disability-Adjusted Life Years) | Global | Both | 90-94 years | Osteoarthritis knee | High body-mass index | Number | 2021 | 37060.9374 | 104857.919 | -3073.2211 |
| YLDs (Years Lived with Disability) | Global | Both | 90-94 years | Osteoarthritis knee | High body-mass index | Number | 2021 | 37060.9374 | 104857.919 | -3073.2211 |
| DALYs (Disability-Adjusted Life Years) | Global | Both | 95+ years | Osteoarthritis knee | High body-mass index | Rate | 1990 | 148.582756 | 422.672775 | -12.099131 |
| YLDs (Years Lived with Disability) | Global | Both | 95+ years | Osteoarthritis knee | High body-mass index | Rate | 1990 | 148.582756 | 422.672775 | -12.099131 |
| DALYs (Disability-Adjusted Life Years) | Global | Both | 95+ years | Osteoarthritis knee | High body-mass index | Rate | 2021 | 192.136767 | 533.811594 | -16.245646 |
| YLDs (Years Lived with Disability) | Global | Both | 95+ years | Osteoarthritis knee | High body-mass index | Rate | 2021 | 192.136767 | 533.811594 | -16.245646 |
| DALYs (Disability-Adjusted Life Years) | Global | Both | 95+ years | Osteoarthritis knee | High body-mass index | Number | 2021 | 10472.0469 | 29094.3796 | -885.43787 |
| YLDs (Years Lived with Disability) | Global | Both | 95+ years | Osteoarthritis knee | High body-mass index | Number | 2021 | 10472.0469 | 29094.3796 | -885.43787 |
| DALYs (Disability-Adjusted Life Years) | Global | Both | 95+ years | Osteoarthritis knee | High body-mass index | Number | 1990 | 1512.70099 | 4303.17449 | -123.17962 |
| YLDs (Years Lived with Disability) | Global | Both | 95+ years | Osteoarthritis knee | High body-mass index | Number | 1990 | 1512.70099 | 4303.17449 | -123.17962 |

Appendix 15: The age-standardized DALYs rate for knee osteoarthritis due to high BMI in 27 regions in 2021

| measure | location | sex | age | cause | rei | metric | year | val | upper | lower |
| --- | --- | --- | --- | --- | --- | --- | --- | --- | --- | --- |
| DALYs (Disability-Adjusted Life Years) | High-income North America | Both | Age-standardized | Osteoarthritis knee | High body-mass index | Rate | 2021 | 69.00394084 | 180.4950986 | -7.686114119 |
| DALYs (Disability-Adjusted Life Years) | Australasia | Both | Age-standardized | Osteoarthritis knee | High body-mass index | Rate | 2021 | 68.37509103 | 183.9341616 | -7.070550606 |
| DALYs (Disability-Adjusted Life Years) | Southern Latin America | Both | Age-standardized | Osteoarthritis knee | High body-mass index | Rate | 2021 | 66.61944513 | 172.4993908 | -7.337480807 |
| DALYs (Disability-Adjusted Life Years) | Central Latin America | Both | Age-standardized | Osteoarthritis knee | High body-mass index | Rate | 2021 | 62.94026112 | 167.1804226 | -6.832121551 |
| DALYs (Disability-Adjusted Life Years) | Andean Latin America | Both | Age-standardized | Osteoarthritis knee | High body-mass index | Rate | 2021 | 61.02897822 | 162.7962031 | -6.480722139 |
| DALYs (Disability-Adjusted Life Years) | Tropical Latin America | Both | Age-standardized | Osteoarthritis knee | High body-mass index | Rate | 2021 | 58.02891473 | 155.3515024 | -5.871159845 |
| DALYs (Disability-Adjusted Life Years) | High SDI | Both | Age-standardized | Osteoarthritis knee | High body-mass index | Rate | 2021 | 57.84805564 | 155.7530708 | -5.903333742 |
| DALYs (Disability-Adjusted Life Years) | North Africa and Middle East | Both | Age-standardized | Osteoarthritis knee | High body-mass index | Rate | 2021 | 55.57431766 | 145.6449306 | -6.330611068 |
| DALYs (Disability-Adjusted Life Years) | Caribbean | Both | Age-standardized | Osteoarthritis knee | High body-mass index | Rate | 2021 | 55.34075183 | 149.4994557 | -5.514969125 |
| DALYs (Disability-Adjusted Life Years) | Western Europe | Both | Age-standardized | Osteoarthritis knee | High body-mass index | Rate | 2021 | 53.4491088 | 144.9943464 | -5.317175826 |
| DALYs (Disability-Adjusted Life Years) | Southern Sub-Saharan Africa | Both | Age-standardized | Osteoarthritis knee | High body-mass index | Rate | 2021 | 53.01572511 | 142.5412356 | -5.37678531 |
| DALYs (Disability-Adjusted Life Years) | High-middle SDI | Both | Age-standardized | Osteoarthritis knee | High body-mass index | Rate | 2021 | 50.83308659 | 139.9580485 | -4.982168884 |
| DALYs (Disability-Adjusted Life Years) | East Asia | Both | Age-standardized | Osteoarthritis knee | High body-mass index | Rate | 2021 | 50.36947517 | 143.9614451 | -4.520376449 |
| DALYs (Disability-Adjusted Life Years) | Eastern Europe | Both | Age-standardized | Osteoarthritis knee | High body-mass index | Rate | 2021 | 47.81464272 | 126.695648 | -4.990363532 |
| DALYs (Disability-Adjusted Life Years) | High-income Asia Pacific | Both | Age-standardized | Osteoarthritis knee | High body-mass index | Rate | 2021 | 47.50975538 | 142.0488395 | -3.830659474 |
| DALYs (Disability-Adjusted Life Years) | Global | Both | Age-standardized | Osteoarthritis knee | High body-mass index | Rate | 2021 | 45.92754267 | 128.3321004 | -4.360318848 |
| DALYs (Disability-Adjusted Life Years) | Oceania | Both | Age-standardized | Osteoarthritis knee | High body-mass index | Rate | 2021 | 45.65247554 | 124.8922164 | -4.660226578 |
| DALYs (Disability-Adjusted Life Years) | Middle SDI | Both | Age-standardized | Osteoarthritis knee | High body-mass index | Rate | 2021 | 45.12375865 | 128.3162868 | -4.145856054 |
| DALYs (Disability-Adjusted Life Years) | Central Europe | Both | Age-standardized | Osteoarthritis knee | High body-mass index | Rate | 2021 | 44.22976744 | 118.4566517 | -4.620645532 |
| DALYs (Disability-Adjusted Life Years) | Western Sub-Saharan Africa | Both | Age-standardized | Osteoarthritis knee | High body-mass index | Rate | 2021 | 38.75988884 | 110.0137027 | -3.324082475 |
| DALYs (Disability-Adjusted Life Years) | Central Asia | Both | Age-standardized | Osteoarthritis knee | High body-mass index | Rate | 2021 | 36.00493412 | 96.01574812 | -3.766759066 |
| DALYs (Disability-Adjusted Life Years) | Low-middle SDI | Both | Age-standardized | Osteoarthritis knee | High body-mass index | Rate | 2021 | 32.00339439 | 90.79871784 | -2.813901112 |
| DALYs (Disability-Adjusted Life Years) | Central Sub-Saharan Africa | Both | Age-standardized | Osteoarthritis knee | High body-mass index | Rate | 2021 | 31.18802168 | 91.839532 | -2.377954382 |
| DALYs (Disability-Adjusted Life Years) | Eastern Sub-Saharan Africa | Both | Age-standardized | Osteoarthritis knee | High body-mass index | Rate | 2021 | 26.13054012 | 76.79924935 | -2.029354923 |
| DALYs (Disability-Adjusted Life Years) | Southeast Asia | Both | Age-standardized | Osteoarthritis knee | High body-mass index | Rate | 2021 | 25.83858274 | 74.3322614 | -2.032275984 |
| DALYs (Disability-Adjusted Life Years) | South Asia | Both | Age-standardized | Osteoarthritis knee | High body-mass index | Rate | 2021 | 25.78560763 | 75.30920901 | -2.049144274 |
| DALYs (Disability-Adjusted Life Years) | Low SDI | Both | Age-standardized | Osteoarthritis knee | High body-mass index | Rate | 2021 | 24.63924639 | 73.61696997 | -1.935706264 |

Appendix 16: The projected age-standardized DALY rate for knee osteoarthritis due to high BMI across 27 regions by 2050

| **location** | **age** | **measure** | **metric** | **sex** | **year** | **val** | **lower** | **upper** |
| --- | --- | --- | --- | --- | --- | --- | --- | --- |
| Australasia | Age-standardized | DALYs (Disability-Adjusted Life Years) | Rate | Both | 2050 | 82.20692 | 73.6036 | 90.81024 |
| Central Latin America | Age-standardized | DALYs (Disability-Adjusted Life Years) | Rate | Both | 2050 | 77.23814 | 76.25639 | 78.21989 |
| Southern Latin America | Age-standardized | DALYs (Disability-Adjusted Life Years) | Rate | Both | 2050 | 76.61359 | 61.72066 | 91.50652 |
| Tropical Latin America | Age-standardized | DALYs (Disability-Adjusted Life Years) | Rate | Both | 2050 | 72.35865 | 71.09001 | 73.62729 |
| High-income North America | Age-standardized | DALYs (Disability-Adjusted Life Years) | Rate | Both | 2050 | 69.22475 | 43.74857 | 94.70093 |
| North Africa and Middle East | Age-standardized | DALYs (Disability-Adjusted Life Years) | Rate | Both | 2050 | 69.14232 | 59.3977 | 78.88693 |
| High SDI | Age-standardized | DALYs (Disability-Adjusted Life Years) | Rate | Both | 2050 | 68.87355 | 62.51924 | 75.22787 |
| Andean Latin America | Age-standardized | DALYs (Disability-Adjusted Life Years) | Rate | Both | 2050 | 67.90448 | 54.17055 | 81.63841 |
| Southern Sub-Saharan Africa | Age-standardized | DALYs (Disability-Adjusted Life Years) | Rate | Both | 2050 | 66.30278 | 65.81553 | 66.79003 |
| East Asia | Age-standardized | DALYs (Disability-Adjusted Life Years) | Rate | Both | 2050 | 64.61155 | 34.76935 | 94.45375 |
| Caribbean | Age-standardized | DALYs (Disability-Adjusted Life Years) | Rate | Both | 2050 | 63.02317 | 55.24517 | 70.80116 |
| High-middle SDI | Age-standardized | DALYs (Disability-Adjusted Life Years) | Rate | Both | 2050 | 62.96162 | 57.59663 | 68.32661 |
| Western Europe | Age-standardized | DALYs (Disability-Adjusted Life Years) | Rate | Both | 2050 | 62.87308 | 53.06951 | 72.67666 |
| Global | Age-standardized | DALYs (Disability-Adjusted Life Years) | Rate | Both | 2050 | 59.34784 | 50.59402 | 68.10165 |
| Middle SDI | Age-standardized | DALYs (Disability-Adjusted Life Years) | Rate | Both | 2050 | 58.9725 | 52.55561 | 65.38938 |
| High-income Asia Pacific | Age-standardized | DALYs (Disability-Adjusted Life Years) | Rate | Both | 2050 | 57.27506 | 55.69519 | 58.85493 |
| Central Sub-Saharan Africa | Age-standardized | DALYs (Disability-Adjusted Life Years) | Rate | Both | 2050 | 52.52889 | 45.03664 | 60.02113 |
| Western Sub-Saharan Africa | Age-standardized | DALYs (Disability-Adjusted Life Years) | Rate | Both | 2050 | 51.24614 | 49.37794 | 53.11435 |
| Oceania | Age-standardized | DALYs (Disability-Adjusted Life Years) | Rate | Both | 2050 | 50.95893 | 36.26837 | 65.64949 |
| Central Europe | Age-standardized | DALYs (Disability-Adjusted Life Years) | Rate | Both | 2050 | 43.15368 | 33.88054 | 52.42681 |
| Central Asia | Age-standardized | DALYs (Disability-Adjusted Life Years) | Rate | Both | 2050 | 41.58116 | 41.01514 | 42.14718 |
| Eastern Europe | Age-standardized | DALYs (Disability-Adjusted Life Years) | Rate | Both | 2050 | 41.18498 | 26.66821 | 55.70174 |
| Low-middle SDI | Age-standardized | DALYs (Disability-Adjusted Life Years) | Rate | Both | 2050 | 40.46692 | 32.34449 | 48.58935 |
| Eastern Sub-Saharan Africa | Age-standardized | DALYs (Disability-Adjusted Life Years) | Rate | Both | 2050 | 37.0298 | 33.21697 | 40.84263 |
| Southeast Asia | Age-standardized | DALYs (Disability-Adjusted Life Years) | Rate | Both | 2050 | 35.11643 | 32.11202 | 38.12084 |
| Low SDI | Age-standardized | DALYs (Disability-Adjusted Life Years) | Rate | Both | 2050 | 34.20333 | 29.45638 | 38.95029 |
| South Asia | Age-standardized | DALYs (Disability-Adjusted Life Years) | Rate | Both | 2050 | 33.81735 | 25.30984 | 42.32485 |

Appendix 17: Projected global DALYs for knee osteoarthritis due to high BMI across all age groups by 2050（Unit: million）

| **year** | **age** | **val** | **lower** | **upper** |
| --- | --- | --- | --- | --- |
| 2022 | 30-34 years | 0.006715917 | 0.00663938 | 0.006792 |
| 2023 | 30-34 years | 0.006841987 | 0.006733748 | 0.00695 |
| 2024 | 30-34 years | 0.006968058 | 0.006835493 | 0.007101 |
| 2025 | 30-34 years | 0.007094128 | 0.006941055 | 0.007247 |
| 2026 | 30-34 years | 0.007220199 | 0.007049058 | 0.007391 |
| 2027 | 30-34 years | 0.007346269 | 0.007158794 | 0.007534 |
| 2028 | 30-34 years | 0.00747234 | 0.007269843 | 0.007675 |
| 2029 | 30-34 years | 0.00759841 | 0.007381932 | 0.007815 |
| 2030 | 30-34 years | 0.00772448 | 0.007494871 | 0.007954 |
| 2031 | 30-34 years | 0.007850551 | 0.007608521 | 0.008093 |
| 2032 | 30-34 years | 0.007976621 | 0.007722779 | 0.00823 |
| 2033 | 30-34 years | 0.008102692 | 0.007837562 | 0.008368 |
| 2034 | 30-34 years | 0.008228762 | 0.007952806 | 0.008505 |
| 2035 | 30-34 years | 0.008354833 | 0.00806846 | 0.008641 |
| 2036 | 30-34 years | 0.008480903 | 0.008184479 | 0.008777 |
| 2037 | 30-34 years | 0.008606974 | 0.008300828 | 0.008913 |
| 2038 | 30-34 years | 0.008733044 | 0.008417476 | 0.009049 |
| 2039 | 30-34 years | 0.008859115 | 0.008534398 | 0.009184 |
| 2040 | 30-34 years | 0.008985185 | 0.00865157 | 0.009319 |
| 2041 | 30-34 years | 0.009111255 | 0.008768974 | 0.009454 |
| 2042 | 30-34 years | 0.009237326 | 0.008886592 | 0.009588 |
| 2043 | 30-34 years | 0.009363396 | 0.009004409 | 0.009722 |
| 2044 | 30-34 years | 0.009489467 | 0.009122411 | 0.009857 |
| 2045 | 30-34 years | 0.009615537 | 0.009240587 | 0.00999 |
| 2046 | 30-34 years | 0.009741608 | 0.009358925 | 0.010124 |
| 2047 | 30-34 years | 0.009867678 | 0.009477417 | 0.010258 |
| 2048 | 30-34 years | 0.009993749 | 0.009596054 | 0.010391 |
| 2049 | 30-34 years | 0.010119819 | 0.009714826 | 0.010525 |
| 2050 | 30-34 years | 0.01024589 | 0.009833728 | 0.010658 |
| 2022 | 35-39 years | 0.064033584 | 0.063589479 | 0.064478 |
| 2023 | 35-39 years | 0.066377574 | 0.065211401 | 0.067544 |
| 2024 | 35-39 years | 0.068249855 | 0.066230118 | 0.07027 |
| 2025 | 35-39 years | 0.069562402 | 0.066729906 | 0.072395 |
| 2026 | 35-39 years | 0.070431338 | 0.066942751 | 0.07392 |
| 2027 | 35-39 years | 0.071088872 | 0.067137891 | 0.07504 |
| 2028 | 35-39 years | 0.071779035 | 0.067532314 | 0.076026 |
| 2029 | 35-39 years | 0.072675208 | 0.068242941 | 0.077107 |
| 2030 | 35-39 years | 0.073840964 | 0.06927851 | 0.078403 |
| 2031 | 35-39 years | 0.075236343 | 0.070558522 | 0.079914 |
| 2032 | 35-39 years | 0.076756343 | 0.071950353 | 0.081562 |
| 2033 | 35-39 years | 0.078281152 | 0.073317785 | 0.083245 |
| 2034 | 35-39 years | 0.079718852 | 0.074566299 | 0.084871 |
| 2035 | 35-39 years | 0.081028533 | 0.075666361 | 0.086391 |
| 2036 | 35-39 years | 0.082221087 | 0.076647374 | 0.087795 |
| 2037 | 35-39 years | 0.083342916 | 0.077571776 | 0.089114 |
| 2038 | 35-39 years | 0.084452071 | 0.07850536 | 0.090399 |
| 2039 | 35-39 years | 0.085596461 | 0.079495364 | 0.091698 |
| 2040 | 35-39 years | 0.086800722 | 0.080560556 | 0.093041 |
| 2041 | 35-39 years | 0.088063914 | 0.081692513 | 0.094435 |
| 2042 | 35-39 years | 0.08936611 | 0.082864762 | 0.095867 |
| 2043 | 35-39 years | 0.090679569 | 0.084045386 | 0.097314 |
| 2044 | 35-39 years | 0.091979697 | 0.085208525 | 0.098751 |
| 2045 | 35-39 years | 0.093252297 | 0.086341293 | 0.100163 |
| 2046 | 35-39 years | 0.094495638 | 0.087444762 | 0.101547 |
| 2047 | 35-39 years | 0.095717954 | 0.08853008 | 0.102906 |
| 2048 | 35-39 years | 0.096932311 | 0.089612257 | 0.104252 |
| 2049 | 35-39 years | 0.098151155 | 0.090704291 | 0.105598 |
| 2050 | 35-39 years | 0.09938241 | 0.091813451 | 0.106951 |
| 2022 | 40-44 years | 0.171117043 | 0.169737867 | 0.172496 |
| 2023 | 40-44 years | 0.176086807 | 0.172354257 | 0.179819 |
| 2024 | 40-44 years | 0.18115506 | 0.174356467 | 0.187954 |
| 2025 | 40-44 years | 0.186040433 | 0.175846584 | 0.196234 |
| 2026 | 40-44 years | 0.190580868 | 0.177004003 | 0.204158 |
| 2027 | 40-44 years | 0.194724868 | 0.17802344 | 0.211426 |
| 2028 | 40-44 years | 0.198506157 | 0.179075426 | 0.217937 |
| 2029 | 40-44 years | 0.202011329 | 0.180286756 | 0.223736 |
| 2030 | 40-44 years | 0.205348303 | 0.181736142 | 0.22896 |
| 2031 | 40-44 years | 0.20862086 | 0.183459114 | 0.233783 |
| 2032 | 40-44 years | 0.211911802 | 0.185456735 | 0.238367 |
| 2033 | 40-44 years | 0.215275008 | 0.1877047 | 0.242845 |
| 2034 | 40-44 years | 0.218735006 | 0.190161653 | 0.247308 |
| 2035 | 40-44 years | 0.222291804 | 0.192776966 | 0.251807 |
| 2036 | 40-44 years | 0.225928503 | 0.195498251 | 0.256359 |
| 2037 | 40-44 years | 0.229619501 | 0.198278165 | 0.260961 |
| 2038 | 40-44 years | 0.23333766 | 0.201079519 | 0.265596 |
| 2039 | 40-44 years | 0.23705953 | 0.203877841 | 0.270241 |
| 2040 | 40-44 years | 0.240768292 | 0.206661238 | 0.274875 |
| 2041 | 40-44 years | 0.244454641 | 0.209428164 | 0.279481 |
| 2042 | 40-44 years | 0.248116075 | 0.212184076 | 0.284048 |
| 2043 | 40-44 years | 0.251755219 | 0.214937965 | 0.288572 |
| 2044 | 40-44 years | 0.255377776 | 0.217699445 | 0.293056 |
| 2045 | 40-44 years | 0.258990575 | 0.220476729 | 0.297504 |
| 2046 | 40-44 years | 0.262600027 | 0.223275525 | 0.301925 |
| 2047 | 40-44 years | 0.266211139 | 0.226098727 | 0.306324 |
| 2048 | 40-44 years | 0.269827071 | 0.228946687 | 0.310707 |
| 2049 | 40-44 years | 0.273449161 | 0.231817817 | 0.315081 |
| 2050 | 40-44 years | 0.277077257 | 0.234709345 | 0.319445 |
| 2022 | 45-49 years | 0.336292144 | 0.333397762 | 0.339187 |
| 2023 | 45-49 years | 0.335269348 | 0.327442479 | 0.343096 |
| 2024 | 45-49 years | 0.335794883 | 0.320922085 | 0.350668 |
| 2025 | 45-49 years | 0.335153511 | 0.312818082 | 0.357489 |
| 2026 | 45-49 years | 0.335421124 | 0.30465094 | 0.366191 |
| 2027 | 45-49 years | 0.335008604 | 0.295502107 | 0.374515 |
| 2028 | 45-49 years | 0.335130546 | 0.286364754 | 0.383896 |
| 2029 | 45-49 years | 0.33485685 | 0.276638454 | 0.393075 |
| 2030 | 45-49 years | 0.334898117 | 0.266941739 | 0.402854 |
| 2031 | 45-49 years | 0.334709915 | 0.256907107 | 0.412513 |
| 2032 | 45-49 years | 0.334707937 | 0.246914527 | 0.422501 |
| 2033 | 45-49 years | 0.334573461 | 0.236745561 | 0.432401 |
| 2034 | 45-49 years | 0.334549622 | 0.226632461 | 0.442467 |
| 2035 | 45-49 years | 0.334449793 | 0.216447762 | 0.452452 |
| 2036 | 45-49 years | 0.33441615 | 0.206333207 | 0.462499 |
| 2037 | 45-49 years | 0.334339383 | 0.196215456 | 0.472463 |
| 2038 | 45-49 years | 0.334302599 | 0.186180983 | 0.482424 |
| 2039 | 45-49 years | 0.334241745 | 0.176188061 | 0.492295 |
| 2040 | 45-49 years | 0.334205376 | 0.166289406 | 0.502121 |
| 2041 | 45-49 years | 0.334155935 | 0.156461441 | 0.51185 |
| 2042 | 45-49 years | 0.334121765 | 0.146736197 | 0.521507 |
| 2043 | 45-49 years | 0.334080825 | 0.137100334 | 0.531061 |
| 2044 | 45-49 years | 0.33404964 | 0.127573138 | 0.540526 |
| 2045 | 45-49 years | 0.334015258 | 0.118146914 | 0.549884 |
| 2046 | 45-49 years | 0.333987295 | 0.108833043 | 0.559142 |
| 2047 | 45-49 years | 0.333958124 | 0.099626851 | 0.568289 |
| 2048 | 45-49 years | 0.333933327 | 0.090534779 | 0.577332 |
| 2049 | 45-49 years | 0.333908399 | 0.081553699 | 0.586263 |
| 2050 | 45-49 years | 0.333886566 | 0.072686939 | 0.595086 |
| 2022 | 50-54 years | 0.536548556 | 0.530352431 | 0.542745 |
| 2023 | 50-54 years | 0.545858143 | 0.532003188 | 0.559713 |
| 2024 | 50-54 years | 0.55516773 | 0.531983956 | 0.578352 |
| 2025 | 50-54 years | 0.564477318 | 0.530539747 | 0.598415 |
| 2026 | 50-54 years | 0.573786905 | 0.527835216 | 0.619739 |
| 2027 | 50-54 years | 0.583096492 | 0.523989232 | 0.642204 |
| 2028 | 50-54 years | 0.592406079 | 0.519092547 | 0.66572 |
| 2029 | 50-54 years | 0.601715667 | 0.513217309 | 0.690214 |
| 2030 | 50-54 years | 0.611025254 | 0.506422635 | 0.715628 |
| 2031 | 50-54 years | 0.620334841 | 0.498758101 | 0.741912 |
| 2032 | 50-54 years | 0.629644429 | 0.490266056 | 0.769023 |
| 2033 | 50-54 years | 0.638954016 | 0.480983219 | 0.796925 |
| 2034 | 50-54 years | 0.648263603 | 0.470941824 | 0.825585 |
| 2035 | 50-54 years | 0.65757319 | 0.460170462 | 0.854976 |
| 2036 | 50-54 years | 0.666882778 | 0.448694722 | 0.885071 |
| 2037 | 50-54 years | 0.676192365 | 0.436537678 | 0.915847 |
| 2038 | 50-54 years | 0.685501952 | 0.423720278 | 0.947284 |
| 2039 | 50-54 years | 0.694811539 | 0.410261651 | 0.979361 |
| 2040 | 50-54 years | 0.704121127 | 0.396179353 | 1.012063 |
| 2041 | 50-54 years | 0.713430714 | 0.381489576 | 1.045372 |
| 2042 | 50-54 years | 0.722740301 | 0.366207312 | 1.079273 |
| 2043 | 50-54 years | 0.732049888 | 0.350346496 | 1.113753 |
| 2044 | 50-54 years | 0.741359476 | 0.333920124 | 1.148799 |
| 2045 | 50-54 years | 0.750669063 | 0.316940354 | 1.184398 |
| 2046 | 50-54 years | 0.75997865 | 0.299418593 | 1.220539 |
| 2047 | 50-54 years | 0.769288238 | 0.281365569 | 1.257211 |
| 2048 | 50-54 years | 0.778597825 | 0.2627914 | 1.294404 |
| 2049 | 50-54 years | 0.787907412 | 0.243705646 | 1.332109 |
| 2050 | 50-54 years | 0.797216999 | 0.224117358 | 1.370317 |
| 2022 | 55-59 years | 0.651858398 | 0.643996676 | 0.65972 |
| 2023 | 55-59 years | 0.676847701 | 0.659268355 | 0.694427 |
| 2024 | 55-59 years | 0.701837004 | 0.672421132 | 0.731253 |
| 2025 | 55-59 years | 0.726826307 | 0.683765879 | 0.769887 |
| 2026 | 55-59 years | 0.751815609 | 0.693511515 | 0.81012 |
| 2027 | 55-59 years | 0.776804912 | 0.701808859 | 0.851801 |
| 2028 | 55-59 years | 0.801794215 | 0.70877306 | 0.894815 |
| 2029 | 55-59 years | 0.826783518 | 0.71449566 | 0.939071 |
| 2030 | 55-59 years | 0.851772821 | 0.719051669 | 0.984494 |
| 2031 | 55-59 years | 0.876762123 | 0.722503988 | 1.03102 |
| 2032 | 55-59 years | 0.901751426 | 0.724906351 | 1.078597 |
| 2033 | 55-59 years | 0.926740729 | 0.726305346 | 1.127176 |
| 2034 | 55-59 years | 0.951730032 | 0.726741872 | 1.176718 |
| 2035 | 55-59 years | 0.976719334 | 0.726252208 | 1.227186 |
| 2036 | 55-59 years | 1.001708637 | 0.72486882 | 1.278548 |
| 2037 | 55-59 years | 1.02669794 | 0.722620984 | 1.330775 |
| 2038 | 55-59 years | 1.051687243 | 0.719535281 | 1.383839 |
| 2039 | 55-59 years | 1.076676545 | 0.715635979 | 1.437717 |
| 2040 | 55-59 years | 1.101665848 | 0.710945357 | 1.492386 |
| 2041 | 55-59 years | 1.126655151 | 0.705483957 | 1.547826 |
| 2042 | 55-59 years | 1.151644454 | 0.699270801 | 1.604018 |
| 2043 | 55-59 years | 1.176633757 | 0.692323572 | 1.660944 |
| 2044 | 55-59 years | 1.201623059 | 0.684658758 | 1.718587 |
| 2045 | 55-59 years | 1.226612362 | 0.676291785 | 1.776933 |
| 2046 | 55-59 years | 1.251601665 | 0.667237127 | 1.835966 |
| 2047 | 55-59 years | 1.276590968 | 0.657508397 | 1.895674 |
| 2048 | 55-59 years | 1.30158027 | 0.64711843 | 1.956042 |
| 2049 | 55-59 years | 1.326569573 | 0.636079357 | 2.01706 |
| 2050 | 55-59 years | 1.351558876 | 0.624402664 | 2.078715 |
| 2022 | 60-64 years | 0.615158272 | 0.609583755 | 0.620733 |
| 2023 | 60-64 years | 0.62814345 | 0.615224339 | 0.641063 |
| 2024 | 60-64 years | 0.642440621 | 0.61799851 | 0.666883 |
| 2025 | 60-64 years | 0.655925624 | 0.618775407 | 0.693076 |
| 2026 | 60-64 years | 0.669913386 | 0.617930397 | 0.721896 |
| 2027 | 60-64 years | 0.683589924 | 0.615466633 | 0.751713 |
| 2028 | 60-64 years | 0.69745912 | 0.61167239 | 0.783246 |
| 2029 | 60-64 years | 0.711209054 | 0.606535241 | 0.815883 |
| 2030 | 60-64 years | 0.725032815 | 0.600219411 | 0.849846 |
| 2031 | 60-64 years | 0.738810875 | 0.592739094 | 0.884883 |
| 2032 | 60-64 years | 0.752617225 | 0.584188863 | 0.921046 |
| 2033 | 60-64 years | 0.766406063 | 0.574595279 | 0.958217 |
| 2034 | 60-64 years | 0.780205741 | 0.564017444 | 0.996394 |
| 2035 | 60-64 years | 0.793998709 | 0.552484221 | 1.035513 |
| 2036 | 60-64 years | 0.807795831 | 0.54003634 | 1.075555 |
| 2037 | 60-64 years | 0.821590381 | 0.526700791 | 1.11648 |
| 2038 | 60-64 years | 0.835386523 | 0.512508128 | 1.158265 |
| 2039 | 60-64 years | 0.84918168 | 0.497482283 | 1.200881 |
| 2040 | 60-64 years | 0.862977447 | 0.481647639 | 1.244307 |
| 2041 | 60-64 years | 0.876772836 | 0.465025074 | 1.288521 |
| 2042 | 60-64 years | 0.890568459 | 0.447634869 | 1.333502 |
| 2043 | 60-64 years | 0.904363937 | 0.429495221 | 1.379233 |
| 2044 | 60-64 years | 0.918159505 | 0.410623469 | 1.425696 |
| 2045 | 60-64 years | 0.931955018 | 0.391035562 | 1.472874 |
| 2046 | 60-64 years | 0.945750564 | 0.370746601 | 1.520755 |
| 2047 | 60-64 years | 0.95954609 | 0.349770677 | 1.569322 |
| 2048 | 60-64 years | 0.973341628 | 0.328121121 | 1.618562 |
| 2049 | 60-64 years | 0.987137159 | 0.305810483 | 1.668464 |
| 2050 | 60-64 years | 1.000932694 | 0.282850656 | 1.719015 |
| 2022 | 65-69 years | 0.60997118 | 0.603737561 | 0.616205 |
| 2023 | 65-69 years | 0.627853746 | 0.615236956 | 0.640471 |
| 2024 | 65-69 years | 0.651698652 | 0.628334032 | 0.675063 |
| 2025 | 65-69 years | 0.669508987 | 0.634146438 | 0.704872 |
| 2026 | 65-69 years | 0.692213079 | 0.641644842 | 0.742781 |
| 2027 | 65-69 years | 0.710255834 | 0.64319431 | 0.777317 |
| 2028 | 65-69 years | 0.732245181 | 0.646189836 | 0.818301 |
| 2029 | 65-69 years | 0.750593929 | 0.644325363 | 0.856862 |
| 2030 | 65-69 years | 0.772096932 | 0.643584129 | 0.90061 |
| 2031 | 65-69 years | 0.790739279 | 0.638839549 | 0.942639 |
| 2032 | 65-69 years | 0.811890836 | 0.634896338 | 0.988885 |
| 2033 | 65-69 years | 0.830787208 | 0.627622236 | 1.033952 |
| 2034 | 65-69 years | 0.851674738 | 0.620861115 | 1.082488 |
| 2035 | 65-69 years | 0.870781274 | 0.611299608 | 1.130263 |
| 2036 | 65-69 years | 0.891465808 | 0.602006305 | 1.180925 |
| 2037 | 65-69 years | 0.910742506 | 0.590333666 | 1.231151 |
| 2038 | 65-69 years | 0.931268908 | 0.578728093 | 1.28381 |
| 2039 | 65-69 years | 0.950681886 | 0.565079241 | 1.336285 |
| 2040 | 65-69 years | 0.971084206 | 0.551335775 | 1.390833 |
| 2041 | 65-69 years | 0.990605712 | 0.535818595 | 1.445393 |
| 2042 | 65-69 years | 1.010910288 | 0.520079006 | 1.501742 |
| 2043 | 65-69 years | 1.030517963 | 0.502782927 | 1.558253 |
| 2044 | 65-69 years | 1.05074538 | 0.48516489 | 1.616326 |
| 2045 | 65-69 years | 1.070421365 | 0.466166184 | 1.674677 |
| 2046 | 65-69 years | 1.090587801 | 0.446769072 | 1.734407 |
| 2047 | 65-69 years | 1.110317894 | 0.426134235 | 1.794502 |
| 2048 | 65-69 years | 1.130436108 | 0.405043185 | 1.855829 |
| 2049 | 65-69 years | 1.150209039 | 0.382831167 | 1.917587 |
| 2050 | 65-69 years | 1.170289108 | 0.360120033 | 1.980458 |
| 2022 | 70-74 years | 0.493225273 | 0.488145364 | 0.498305 |
| 2023 | 70-74 years | 0.518093807 | 0.505411953 | 0.530776 |
| 2024 | 70-74 years | 0.542922584 | 0.520427319 | 0.565418 |
| 2025 | 70-74 years | 0.567739933 | 0.533615427 | 0.601864 |
| 2026 | 70-74 years | 0.592553998 | 0.545239086 | 0.639869 |
| 2027 | 70-74 years | 0.617367118 | 0.555474158 | 0.67926 |
| 2028 | 70-74 years | 0.642179967 | 0.564447109 | 0.719913 |
| 2029 | 70-74 years | 0.666992738 | 0.572254251 | 0.761731 |
| 2030 | 70-74 years | 0.691805486 | 0.578972171 | 0.804639 |
| 2031 | 70-74 years | 0.716618228 | 0.584663767 | 0.848573 |
| 2032 | 70-74 years | 0.741430968 | 0.589381965 | 0.89348 |
| 2033 | 70-74 years | 0.766243707 | 0.593172151 | 0.939315 |
| 2034 | 70-74 years | 0.791056447 | 0.596073829 | 0.986039 |
| 2035 | 70-74 years | 0.815869186 | 0.598121811 | 1.033617 |
| 2036 | 70-74 years | 0.840681925 | 0.599347083 | 1.082017 |
| 2037 | 70-74 years | 0.865494665 | 0.599777467 | 1.131212 |
| 2038 | 70-74 years | 0.890307404 | 0.599438128 | 1.181177 |
| 2039 | 70-74 years | 0.915120143 | 0.598351971 | 1.231888 |
| 2040 | 70-74 years | 0.939932883 | 0.596539969 | 1.283326 |
| 2041 | 70-74 years | 0.964745622 | 0.594021414 | 1.33547 |
| 2042 | 70-74 years | 0.989558361 | 0.590814134 | 1.388303 |
| 2043 | 70-74 years | 1.0143711 | 0.586934671 | 1.441808 |
| 2044 | 70-74 years | 1.03918384 | 0.582398426 | 1.495969 |
| 2045 | 70-74 years | 1.063996579 | 0.577219786 | 1.550773 |
| 2046 | 70-74 years | 1.088809318 | 0.571412234 | 1.606206 |
| 2047 | 70-74 years | 1.113622058 | 0.564988433 | 1.662256 |
| 2048 | 70-74 years | 1.138434797 | 0.557960313 | 1.718909 |
| 2049 | 70-74 years | 1.163247536 | 0.550339136 | 1.776156 |
| 2050 | 70-74 years | 1.188060276 | 0.542135555 | 1.833985 |
| 2022 | 75-79 years | 0.312649294 | 0.311625483 | 0.313673 |
| 2023 | 75-79 years | 0.319913143 | 0.316928661 | 0.322898 |
| 2024 | 75-79 years | 0.327278346 | 0.320959769 | 0.333597 |
| 2025 | 75-79 years | 0.33488225 | 0.323911428 | 0.345853 |
| 2026 | 75-79 years | 0.342700932 | 0.326147136 | 0.359255 |
| 2027 | 75-79 years | 0.350601598 | 0.328004827 | 0.373198 |
| 2028 | 75-79 years | 0.35844027 | 0.329671965 | 0.387209 |
| 2029 | 75-79 years | 0.366141461 | 0.331174601 | 0.401108 |
| 2030 | 75-79 years | 0.373721679 | 0.33244 | 0.415003 |
| 2031 | 75-79 years | 0.381258011 | 0.333371153 | 0.429145 |
| 2032 | 75-79 years | 0.388832052 | 0.333899965 | 0.443764 |
| 2033 | 75-79 years | 0.396485209 | 0.334012193 | 0.458958 |
| 2034 | 75-79 years | 0.404206447 | 0.333743104 | 0.47467 |
| 2035 | 75-79 years | 0.411951067 | 0.333150405 | 0.490752 |
| 2036 | 75-79 years | 0.419672859 | 0.332283455 | 0.507062 |
| 2037 | 75-79 years | 0.427349161 | 0.331166857 | 0.523531 |
| 2038 | 75-79 years | 0.434987181 | 0.329802144 | 0.540172 |
| 2039 | 75-79 years | 0.44261281 | 0.328179598 | 0.557046 |
| 2040 | 75-79 years | 0.450252199 | 0.326290583 | 0.574214 |
| 2041 | 75-79 years | 0.457917723 | 0.324134699 | 0.591701 |
| 2042 | 75-79 years | 0.465604755 | 0.321720373 | 0.609489 |
| 2043 | 75-79 years | 0.473298312 | 0.319060718 | 0.627536 |
| 2044 | 75-79 years | 0.480983606 | 0.31616823 | 0.645799 |
| 2045 | 75-79 years | 0.488653898 | 0.313051436 | 0.664256 |
| 2046 | 75-79 years | 0.496312116 | 0.309714529 | 0.68291 |
| 2047 | 75-79 years | 0.503966923 | 0.30615912 | 0.701775 |
| 2048 | 75-79 years | 0.511626675 | 0.302386474 | 0.720867 |
| 2049 | 75-79 years | 0.519295033 | 0.298399006 | 0.740191 |
| 2050 | 75-79 years | 0.526970161 | 0.294200596 | 0.75974 |
| 2022 | 80-84 years | 0.194367406 | 0.193140605 | 0.195594 |
| 2023 | 80-84 years | 0.196542842 | 0.193022724 | 0.200063 |
| 2024 | 80-84 years | 0.197943448 | 0.191025675 | 0.204861 |
| 2025 | 80-84 years | 0.198809847 | 0.187448061 | 0.210172 |
| 2026 | 80-84 years | 0.199307936 | 0.182528053 | 0.216088 |
| 2027 | 80-84 years | 0.199552094 | 0.1764533 | 0.222651 |
| 2028 | 80-84 years | 0.199621178 | 0.169371271 | 0.229871 |
| 2029 | 80-84 years | 0.199569557 | 0.161398177 | 0.237741 |
| 2030 | 80-84 years | 0.199434716 | 0.152626143 | 0.246243 |
| 2031 | 80-84 years | 0.199242499 | 0.143128812 | 0.255356 |
| 2032 | 80-84 years | 0.199010723 | 0.132965645 | 0.265056 |
| 2033 | 80-84 years | 0.198751675 | 0.122185178 | 0.275318 |
| 2034 | 80-84 years | 0.198473822 | 0.110827506 | 0.28612 |
| 2035 | 80-84 years | 0.198183006 | 0.098926149 | 0.29744 |
| 2036 | 80-84 years | 0.197883251 | 0.086509469 | 0.309257 |
| 2037 | 80-84 years | 0.197577334 | 0.073601751 | 0.321553 |
| 2038 | 80-84 years | 0.197267168 | 0.060224022 | 0.33431 |
| 2039 | 80-84 years | 0.196954073 | 0.046394681 | 0.347513 |
| 2040 | 80-84 years | 0.196638958 | 0.03212999 | 0.361148 |
| 2041 | 80-84 years | 0.196322451 | 0.017444447 | 0.3752 |
| 2042 | 80-84 years | 0.196004984 | 0.002351087 | 0.389659 |
| 2043 | 80-84 years | 0.195686855 | -0.013138286 | 0.404512 |
| 2044 | 80-84 years | 0.19536827 | -0.029012911 | 0.419749 |
| 2045 | 80-84 years | 0.19504937 | -0.045262919 | 0.435362 |
| 2046 | 80-84 years | 0.194730253 | -0.061879214 | 0.45134 |
| 2047 | 80-84 years | 0.194410986 | -0.07885337 | 0.467675 |
| 2048 | 80-84 years | 0.194091617 | -0.09617755 | 0.484361 |
| 2049 | 80-84 years | 0.193772176 | -0.113844436 | 0.501389 |
| 2050 | 80-84 years | 0.193452686 | -0.131847175 | 0.518753 |
| 2022 | 85-89 years | 0.101742987 | 0.101144463 | 0.102342 |
| 2023 | 85-89 years | 0.103811782 | 0.101942761 | 0.105681 |
| 2024 | 85-89 years | 0.105954077 | 0.10209661 | 0.109812 |
| 2025 | 85-89 years | 0.108002517 | 0.101752583 | 0.114252 |
| 2026 | 85-89 years | 0.110090642 | 0.101046226 | 0.119135 |
| 2027 | 85-89 years | 0.112153599 | 0.099995584 | 0.124312 |
| 2028 | 85-89 years | 0.114229867 | 0.098655806 | 0.129804 |
| 2029 | 85-89 years | 0.116298536 | 0.097039054 | 0.135558 |
| 2030 | 85-89 years | 0.118371402 | 0.095172055 | 0.141571 |
| 2031 | 85-89 years | 0.120441917 | 0.093066031 | 0.147818 |
| 2032 | 85-89 years | 0.12251374 | 0.090736419 | 0.154291 |
| 2033 | 85-89 years | 0.124584834 | 0.088192789 | 0.160977 |
| 2034 | 85-89 years | 0.126656334 | 0.085445462 | 0.167867 |
| 2035 | 85-89 years | 0.128727608 | 0.082502306 | 0.174953 |
| 2036 | 85-89 years | 0.130799008 | 0.079370962 | 0.182227 |
| 2037 | 85-89 years | 0.132870337 | 0.076057879 | 0.189683 |
| 2038 | 85-89 years | 0.134941706 | 0.072569087 | 0.197314 |
| 2039 | 85-89 years | 0.137013053 | 0.068909934 | 0.205116 |
| 2040 | 85-89 years | 0.139084412 | 0.065085375 | 0.213083 |
| 2041 | 85-89 years | 0.141155764 | 0.061099914 | 0.221212 |
| 2042 | 85-89 years | 0.14322712 | 0.056957729 | 0.229497 |
| 2043 | 85-89 years | 0.145298474 | 0.052662675 | 0.237934 |
| 2044 | 85-89 years | 0.147369829 | 0.048218343 | 0.246521 |
| 2045 | 85-89 years | 0.149441184 | 0.043628077 | 0.255254 |
| 2046 | 85-89 years | 0.151512538 | 0.038895008 | 0.26413 |
| 2047 | 85-89 years | 0.153583893 | 0.034022074 | 0.273146 |
| 2048 | 85-89 years | 0.155655248 | 0.029012037 | 0.282298 |
| 2049 | 85-89 years | 0.157726602 | 0.023867502 | 0.291586 |
| 2050 | 85-89 years | 0.159797957 | 0.018590931 | 0.301005 |
| 2022 | 90-94 years | 0.037859531 | 0.03763433 | 0.038085 |
| 2023 | 90-94 years | 0.038367388 | 0.037632674 | 0.039102 |
| 2024 | 90-94 years | 0.038812137 | 0.037292294 | 0.040332 |
| 2025 | 90-94 years | 0.039339766 | 0.036824326 | 0.041855 |
| 2026 | 90-94 years | 0.039992148 | 0.036343318 | 0.043641 |
| 2027 | 90-94 years | 0.040738923 | 0.035871962 | 0.045606 |
| 2028 | 90-94 years | 0.041524546 | 0.035381046 | 0.047668 |
| 2029 | 90-94 years | 0.042303554 | 0.034830445 | 0.049777 |
| 2030 | 90-94 years | 0.043054854 | 0.034193424 | 0.051916 |
| 2031 | 90-94 years | 0.043778997 | 0.033462634 | 0.054095 |
| 2032 | 90-94 years | 0.044487667 | 0.032644694 | 0.056331 |
| 2033 | 90-94 years | 0.045193491 | 0.031751469 | 0.058636 |
| 2034 | 90-94 years | 0.045904292 | 0.030793379 | 0.061015 |
| 2035 | 90-94 years | 0.046622089 | 0.029776557 | 0.063468 |
| 2036 | 90-94 years | 0.047345007 | 0.028703181 | 0.065987 |
| 2037 | 90-94 years | 0.048069913 | 0.027573286 | 0.068567 |
| 2038 | 90-94 years | 0.048794328 | 0.02638656 | 0.071202 |
| 2039 | 90-94 years | 0.049517155 | 0.025143368 | 0.073891 |
| 2040 | 90-94 years | 0.050238487 | 0.023844926 | 0.076632 |
| 2041 | 90-94 years | 0.050958998 | 0.02249298 | 0.079425 |
| 2042 | 90-94 years | 0.051679387 | 0.021089359 | 0.082269 |
| 2043 | 90-94 years | 0.052400072 | 0.019635669 | 0.085164 |
| 2044 | 90-94 years | 0.053121149 | 0.018133195 | 0.088109 |
| 2045 | 90-94 years | 0.053842503 | 0.016582953 | 0.091102 |
| 2046 | 90-94 years | 0.054563958 | 0.014985807 | 0.094142 |
| 2047 | 90-94 years | 0.055285379 | 0.01334257 | 0.097228 |
| 2048 | 90-94 years | 0.056006709 | 0.011654056 | 0.100359 |
| 2049 | 90-94 years | 0.056727957 | 0.009921085 | 0.103535 |
| 2050 | 90-94 years | 0.057449162 | 0.008144469 | 0.106754 |
| 2022 | 95+ years | 0.010960836 | 0.010880324 | 0.011041 |
| 2023 | 95+ years | 0.011417958 | 0.011200661 | 0.011635 |
| 2024 | 95+ years | 0.011859031 | 0.011452291 | 0.012266 |
| 2025 | 95+ years | 0.012291969 | 0.011650282 | 0.012934 |
| 2026 | 95+ years | 0.012720784 | 0.011804479 | 0.013637 |
| 2027 | 95+ years | 0.013147511 | 0.011921471 | 0.014374 |
| 2028 | 95+ years | 0.013573178 | 0.01200582 | 0.015141 |
| 2029 | 95+ years | 0.013998308 | 0.012060812 | 0.015936 |
| 2030 | 95+ years | 0.014423166 | 0.012088906 | 0.016757 |
| 2031 | 95+ years | 0.014847887 | 0.012092014 | 0.017604 |
| 2032 | 95+ years | 0.015272538 | 0.012071669 | 0.018473 |
| 2033 | 95+ years | 0.015697153 | 0.012029135 | 0.019365 |
| 2034 | 95+ years | 0.01612175 | 0.01196548 | 0.020278 |
| 2035 | 95+ years | 0.016546338 | 0.011881623 | 0.021211 |
| 2036 | 95+ years | 0.016970922 | 0.011778364 | 0.022163 |
| 2037 | 95+ years | 0.017395503 | 0.011656414 | 0.023135 |
| 2038 | 95+ years | 0.017820083 | 0.011516407 | 0.024124 |
| 2039 | 95+ years | 0.018244663 | 0.011358913 | 0.02513 |
| 2040 | 95+ years | 0.018669242 | 0.011184451 | 0.026154 |
| 2041 | 95+ years | 0.019093821 | 0.010993497 | 0.027194 |
| 2042 | 95+ years | 0.0195184 | 0.010786484 | 0.02825 |
| 2043 | 95+ years | 0.019942978 | 0.010563817 | 0.029322 |
| 2044 | 95+ years | 0.020367557 | 0.010325868 | 0.030409 |
| 2045 | 95+ years | 0.020792136 | 0.010072984 | 0.031511 |
| 2046 | 95+ years | 0.021216715 | 0.009805489 | 0.032628 |
| 2047 | 95+ years | 0.021641294 | 0.009523689 | 0.033759 |
| 2048 | 95+ years | 0.022065873 | 0.009227867 | 0.034904 |
| 2049 | 95+ years | 0.022490451 | 0.008918294 | 0.036063 |
| 2050 | 95+ years | 0.02291503 | 0.008595222 | 0.037235 |
